# Tandem aza-Heck Suzuki and carbonylation reactions of *O*-phenyl hydroxamic ethers: complex lactams *via* carboamination†

**DOI:** 10.1039/d1sc02075g

**Published:** 2021-05-27

**Authors:** Run-Duo Gao, Scott A. Shuler, Donald A. Watson

**Affiliations:** Department of Chemistry and Biochemistry, University of Delaware Newark DE 19716 USA dawatson@udel.edu

## Abstract

The palladium-catalysed tandem aza-Heck–Suzuki and aza-Heck–carbonylation reactions of *O*-phenyl hydroxamic ethers are reported. These formal alkene carboamination reactions provide highly versatile access to wide range complex, stereogenic secondary lactams and exhibit outstanding functional group tolerance and high diastereoselectivity.

## Abstract 2

The palladium-catalysed tandem aza-Heck–Suzuki and aza-Heck–carbonylation reactions of *O*-phenyl hydroxamic ethers are reported.

## Graphical abstract

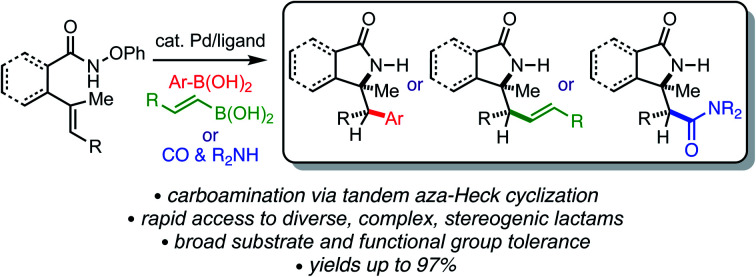

## Introduction

Nitrogen-containing heterocycles are versatile moieties for the synthesis of natural products and bioactive molecules.^1^ In particular, γ-lactams are both highly prevalent and also extremely valuable intermediates for the synthesis of a variety of other heterocycles.^2^ Simultaneously, the incorporation of stereocenters into drug-like compounds has been shown important for improvement of selectivity and other properties.^3^ These facts have combined to create high demand for new methods that can rapidly access stereogenic γ-lactams and other nitrogen heterocycles.

Aza-Heck cyclizations,^4,5^ which employ an umpolung strategy involving palladium-catalysed activation of an electrophilic nitrogen followed by cyclization onto a pendant alkene, have proven to be a powerful method for construction of stereogenic nitrogen heterocycles. When sequenced with other cross-coupling steps,^6^ tandem aza-Heck cyclizations present a powerful opportunity to prepare complex heterocycles in a highly convergent and modular fashion. While several of these formal carboamination reactions have been reported,^7^ all have been limited to the use of oxime esters as the nitrogen electrophile and only deliver cyclic ketimines.^8,9^ The feasibility of using other nitrogen electrophiles in tandem aza-Heck reactions has not yet been explored.

**Fig. 1:**
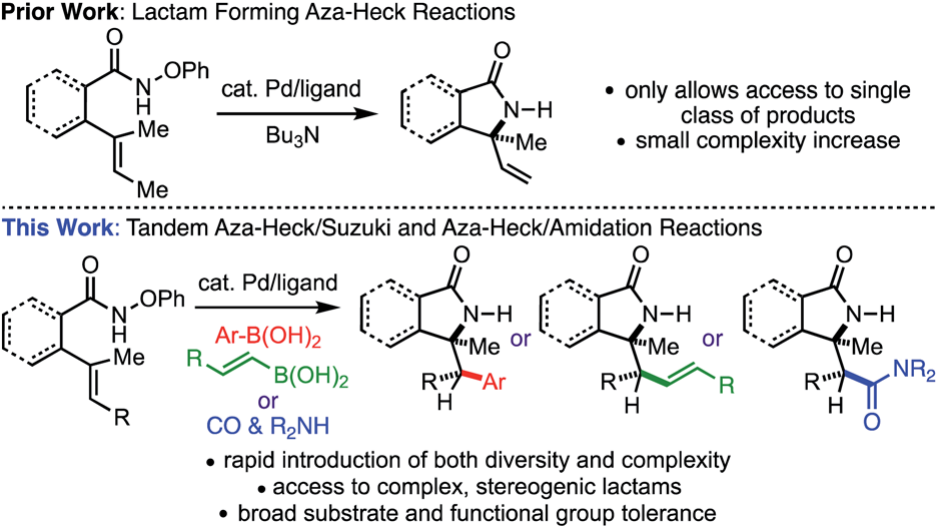
Carboamination *via* tandem aza-Heck cyclization of *O*-phenyl hydroxamic ethers.

We now report that aza-Heck cyclizations of *O*-phenyl hydroxamic ethers^5*r*^ can be sequenced with both palladium-catalysed Suzuki reactions and palladium-catalysed carbonylative amidation reactions to effect formal alkene carboamination. These reactions provide rapid access to complex, stereogenic γ-lactams with a variety of pendant functional groups (Fig. 1). In some cases, we also demonstrate that, as a result of suprafacial aminopalladation of the alkene, two stereogenic centers can arise from the process. These tandem reactions result in rapid increases of molecular complexity, providing highly valuable lactams that are rich in heteroatom content and stereogenic complexity. These reactions also open new vistas for the possibility of other classes of tandem aza-Heck reactions that are not reliant on oxime electrophiles and the myriad of heterocycles that can result.

## Results and discussion

At the outset, the tandem aza-Heck/Suzuki reaction involving *N*-phenoxy-benzamide (**1**) and phenylboronic acid (**2**) was chosen as the model system for condition development (Table 1). Starting with conditions from our prior work on *O*-phenyl hydroxamic ether cyclization [(COD)Pd(CH_2_SiMe_3_)_2_, tris(2,2,2-trifluoroethyl) phosphite, Bu_3_N in MeCN],^5*r*^ we were pleased to see small a amount of desired product **3**, however the yield was low (18%) and significant a amount of uncyclized primary amide **4** was also observed (Table 1, entry 1). Gratifyingly, omission of the base, which was critical for good yields in prior aza-Heck reactions of this class of electrophiles,^5*r*^ resulted in significantly higher yield of **3** (74%) and less byproduct **4** (entry 2). The use of other solvents was explored, but none proved superior to MeCN.^10,11^ Based on data presented below, it is particularly notable that dioxane was far inferior in this regard (entry 3). Addition of 4 Å molecular sieves (4 Å MS) further decreased the production of **4** and improved the yield of **3** (entry 4). Other desiccants were less effective.^10^ Finally, as (COD)Pd(CH_2_SiMe_3_)_2_ has limitations with respect to commercial supply and thermal stability, we sought to identify a more general Pd-precatalyst. While several were found to work,^10^ Pd(OAc)_2_ proved the most effective (entry 5). Under these conditions, catalyst loading could be halved, and the reaction concentration increased, without negative impact (entry 6). We believe that the reason that Pd(OAc)_2_ is effective in this transformation, and was not in the earlier aza-Heck reaction is due to the presence of phenyl boronic acid **2**. Aryl boronic acids have been shown to be an effective reductant for Pd(ii) precatalysts.^12^

**Table 1:** Optimization of Aza-Heck–Suzuki reaction^a^

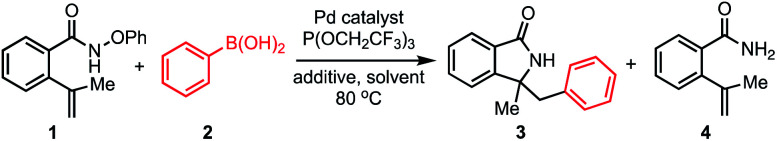				
Entry	Pd catalyst [mol%]	Solvent	Additive	Yield **3**^b^ [%]
1	(COD)Pd(CH_2_SiMe_3_)_2_ [10]	MeCN	Bu_3_N	18
2	(COD)Pd(CH_2_SiMe_3_)_2_ [10]	MeCN	—	74
3	(COD)Pd(CH_2_SiMe_3_)_2_ [10]	Dioxane	—	15
4	(COD)Pd(CH_2_SiMe_3_)_2_ [10]	MeCN	4 Å MS	81
5	Pd(OAc)_2_ [10]	MeCN	4 Å MS	81
6^c^	Pd(OAc)_2_ [5]	MeCN	4 Å MS	81

a Unless otherwise noted, reactions run with 0.2 mmol **1**, 0.6 mmol **2**, 10 mmol% Pd catalyst and 30 mmol% P(OCH_2_CF_3_)_3_ at 0.05 M.

b Yield calculated by ^1^H NMR with 1,3,5-trimethoxybenzene as internal standard.

c 5 mol% Pd(OAc)_2_ and 15 mmol% P(OCH_2_CF_3_)_3_ at 0.1 M.

**Scheme 1:**
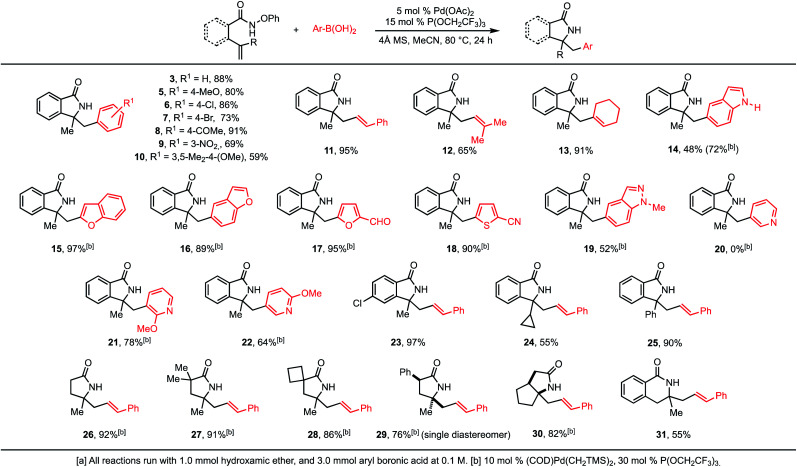
Substrate scope of Aza-Heck–Suzuki reaction.^a^

The scope of reaction was next explored, and the model substrate **3** was obtained in 88% isolated yield (Scheme 1). High yields were observed with a wide range of functional groups on the phenylboronic acid (**5–10**). Notably, this includes aryl bromides and chlorides, as well as nitroarenes,^13^ all of which are also competent electrophiles in cross-coupling reactions. Vinylboronic acids also can be used, leading to products with a variety of alkene substitutions (**11–13**). It is also notable that these alkenyl products are one carbon homologs of the alkenes that can be prepared by direct aza-Heck reaction. Heterocyclic boronic esters were also well tolerated. With the exception of unencumbered pyridines, a wide variety of such compounds could be coupled in good yields, leading to polyheterocyclic compounds (**14–22**). These include protic groups (**14**). Also as exemplified by **14**, the use of (COD)Pd(CH_2_SiMe_3_)_2_ as precatalyst was preferred in these cases.

The nature of the hydroxamic ether could also be varied. This includes substitution on the aromatic ring (**23**), and modulation of the alkene substituents (**24–25**), as well as more dramatic changes (**26–31**). In the cases of **29** and **30**, single diastereomers of product were observed. NMR studies confirmed the relative stereochemistry as shown in Scheme 1.^10^ Finally, in some cases, 6-*exo* aza-Heck cyclizations can also be included in the tandem sequence (**31**).

In general, stability of the putative alkyl palladium intermediate dictates which substrates are compatible with the tandem aza-Heck/Suzuki reaction. In substrates where the initial cyclization results in an alkyl palladium complex that can rapidly undergo β-hydride elimination, the tandem sequence was not successful. For example, when substrate **32** was subjected to the tandem aza-Heck/Suzuki conditions, the only cyclization product that was observed was alkene **33** (eqn (1)).^14^
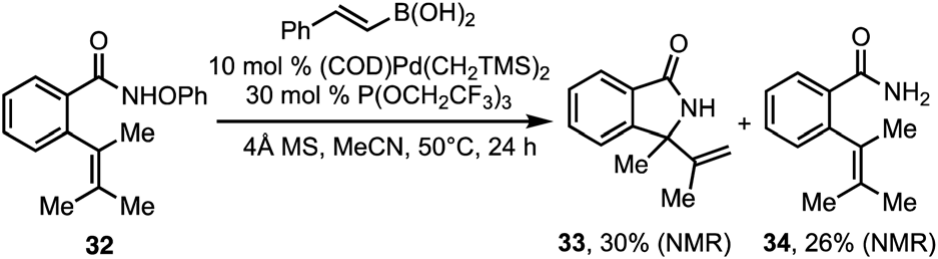


**Scheme 2:**
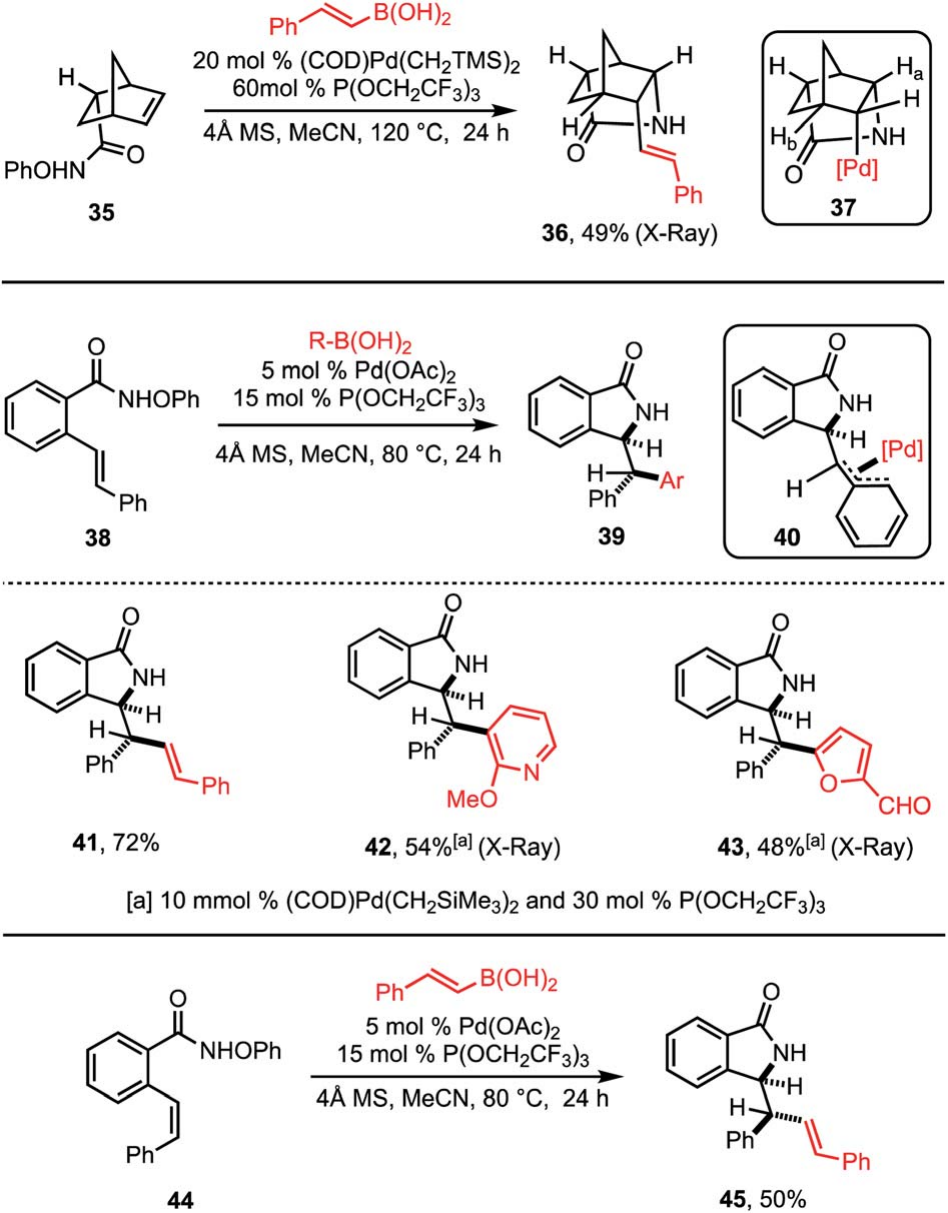
Formation of vicinal stereocenters *via* Aza-Heck–Suzuki reaction.

In the cases shown in Table 1, β-hydride elimination is prevented by the formation of neopentylic palladium alkyl intermediates that lack β-hydrogen atoms. However, success can also be realized in cases where β-hydride elimination is possible but the alkyl palladium intermediate is stabilized against it. Examples are shown in Scheme 2. First, in the case of the cyclization of norbornene **35**, a single diastereomer of the polycyclic product **36** was observed (Scheme 2, top). The stereochemistry of the product (established by X-ray crystallographic analysis) indicates that the reaction proceeds *via* suprafacial aminopalladation followed by stereoretentive cross-coupling, which is consistent with an aza-Heck/Suzuki pathway. In this case, β-hydride elimination from alkyl palladium intermediate **37** is disfavored, as elimination of H_a_ would be forced to proceed *via* an antarafacial pathway and elimination of H_b_ would result in an anti-Bredt olefin.

In the second example, styrenyl substate **38** was also found to participate in a tandem aza-Heck–Suzuki reaction without competing β-hydride elimination to provide a single diastereoisomer of **39** in which two new stereogenic centers are formed (Scheme 2, middle). As in the previous case, the stereochemistry of the observed products (also established by X-ray crystallographic analysis) is consistent with suprafacial aminopalladation followed by stereoretentive cross-coupling. In this case, the stabilization of the alkyl palladium intermediate afforded by the benzallyl fragment in intermediate **40** appears sufficient to allow transmetallation by the aryl boronic acid to out compete β-hydride elimination. It is notable that both alkenyl and (hetero)aryl boronic acids were capable of participating in this transformation (**41–43**). Finally, it is likewise notable that substrate **44**, which bears the opposite alkene configuration, led to the diastereomeric product **45**. This result is also consistent with the proposed aza-Heck–Suzuki pathway.

In addition to tandem aza-Heck–Suzuki reactions, we were interested in exploring other classes of tandem reactivity. Tandem aza-Heck–carbonylation reactions were very attractive in this regard due to the versatility and variety of products that such a reaction could afford. To explore the possibility, the reaction of model substrate **1** and a variety of simple amines under an atmosphere of CO was explored (Table 2). We were initially pleased to find that when morpholine was used as the nucleophile, the conditions developed for tandem Suzuki reactions provided high yields of the amidolactam product **46** (entry 1). However, the reaction yield proved to be highly dependent on the nucleophile employed; other simple amines provided unacceptably low yields under these conditions (entries 2–3). Fortunately, we found that by switching the solvent from MeCN to dioxane, uniformly good yields of products were observed (entries 4–6). This is notable, as dioxane was a particularly poor solvent for the tandem Suzuki reactions (see Table 1).^15^ In addition, when using dioxane, molecular sieves proved both unnecessary and slightly deleterious. As such, they were omitted from the tandem carbonylation reactions.

**Table 2:** Optimization of Aza-Heck–carbonylation reaction^a^

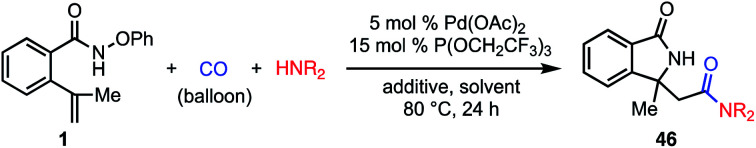				
Entry	Solvent	Amine	Additive	Yield **46**^b^ [%]
1	MeCN	Morpholine	4 Å MS	81
2	MeCN	*n*-Bu_2_NH	4 Å MS	36
3	MeCN	BnNH_2_	4 Å MS	12
4	Dioxane	Morpholine	—	87
5	Dioxane	*n*-Bu_2_NH	—	82
6	Dioxane	BnNH_2_	—	80

a Unless otherwise noted, reactions run with 0.2 mmol **1** and 0.3 mmol amine.

b Yields calculated by ^1^H NMR with 1,3,5-trimethoxybenzene as internal standard.

**Scheme 3:**
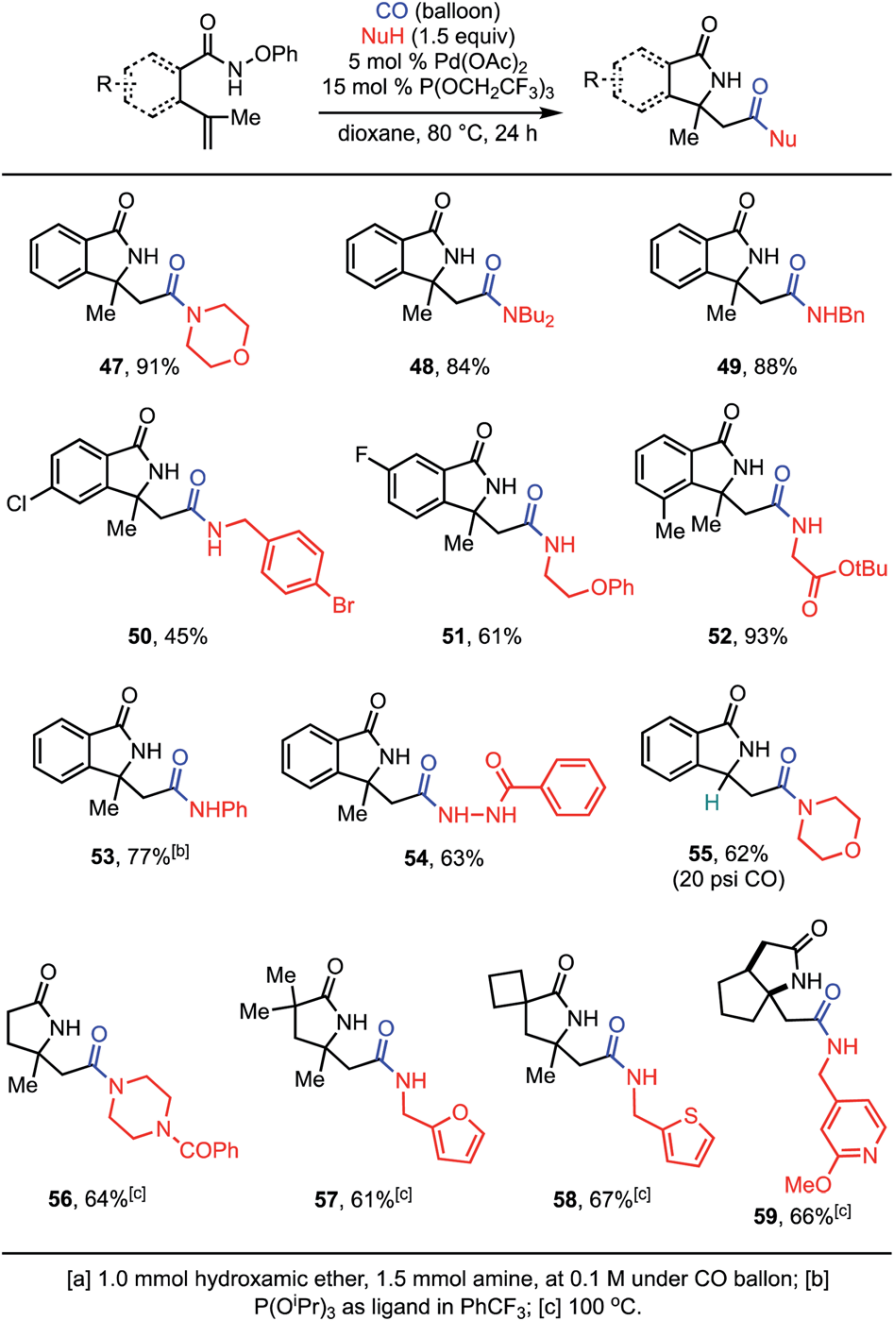
Scope of Aza-Heck–carbonylation reaction.^a^

With optimized conditions in hand, we then explored the scope of the tandem aza-Heck–carbonylation reaction (Scheme 3). A wide variety of primary, cyclic secondary, and acyclic secondary amines participate in the reaction and afford amidolactams in good to excellent yields. Aryl fluorides, chlorides, and bromides are all tolerated (**50** and **51**), as are ethers and esters (**51** and **52**). Heterocycles were also highly compatible (**47**, **55–59**). Aniline could be used as the nucleophile (**53**), but in this case specialized conditions were required.^10^ Unfortunately, the attempted use of various alcohols led to mixtures of products containing both the desired esters and phenolic esters resulting from the incorporation of the co-generated PhOH in poor yield (not shown). However, acyl hydrazines did prove effective alternatives to amines (**54**).^16^ In some cases, substates that could potentially undergo β-hydride elimination after the aza-Heck step could be utilized (**55**). In these cases, increased pressure of CO was required for migratory insertion to outcompete alkene formation. Finally, as with the tandem Suzuki reactions above, the structure of the hydroxamic ether could be varied (**56–59**), although slightly elevated temperatures were required. Overall, this provides rapid and efficient entry to diverse amidolactams.

**Fig. 2:**
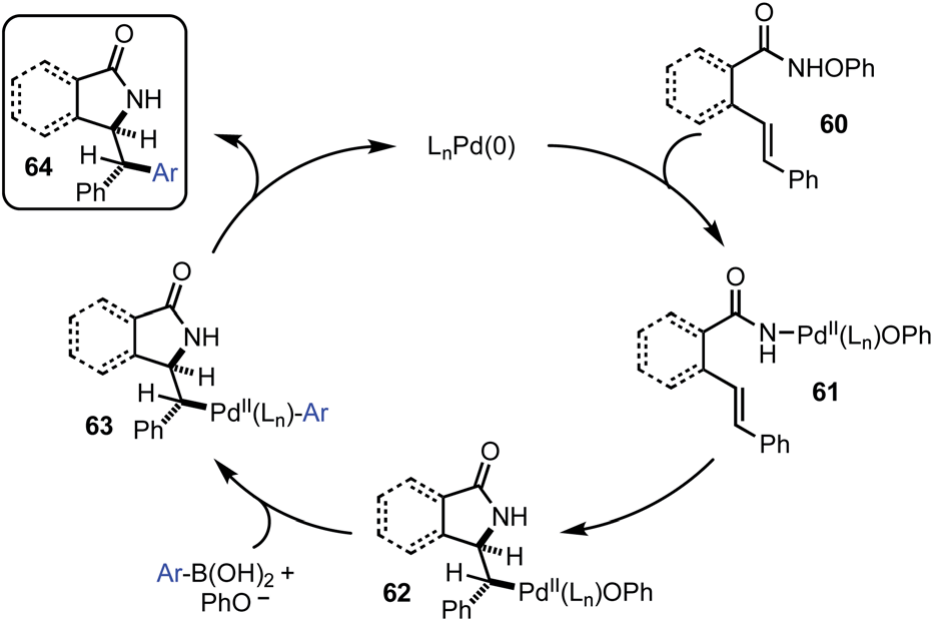
Proposed mechanism.

Based upon the data presented herein, we believe these reactions proceed *via* the mechanism outlined in Fig. 2. Initial oxidative addition by an active Pd(0) complex results in Pd(ii) complex **61**. Capture of this intermediate, boronic acid (Schemes 1 and 2) or CO/amine (Scheme 3), and subsequent reductive elimination then results in the product. Notably, in cases where two stereocenters are formed, diastereoselectivity is consist with suprafacial aminopalladation and stereoretentive transmetallation, consistent with a Heck-type mechanism.

## Conclusions

In summary, we have developed effective conditions for lactam-forming tandem aza-Heck–Suzuki and aza-Heck–carbonylation reactions. The scope for both of these formal carboamination reaction is large, and the reactions lead to a variety of complex stereogenic γ-lactams that are relevant to the preparation of bioactive molecules. All observations in this study, including the relative stereochemistry in cases where multiple stereocenters were formed, are consistent with a Heck-type mechanism in which the alkyl palladium intermediate is captured in a subsequent cross-coupling reaction. These transformations also demonstrate the feasibility of non-oxime aza-Heck reactions to participate in tandem processes. Further studies in our laboratory are underway to expand these processes to other cases of complex hetereocycles.

## Author contributions

R.-D. G. and S. A. S. contributed to the experimental work. All authors contributed to ideation and writing of the paper.

## Conflicts of interest

There are no conflicts to declare.

## Supplementary Material

SC-012-D1SC02075G-s001

SC-012-D1SC02075G-s002

## References

[cit1] (b) Joule J. A., in Advances in Heterocyclic Chemistry, ed. E. F. V. Scriven and C. A. Ramsden, Academic Press, 2016, vol. 119, pp. 81–106

[cit2] Caruano J., Muccioli G. G., Robiette R. (2016). Org. Biomol. Chem..

[cit3] Lovering F., Bikker J., Humblet C. (2009). J. Med. Chem..

[cit4] Narasaka K., Kitamura M. (2005). Eur. J. Org. Chem..

[cit5] Tsutsui H., Narasaka K. (1999). Chem. Lett..

[cit6] (b) Tietze L. F. and Levy L. M., in The Mizoroki–Heck Reaction, 2009, pp. 281–344

[cit7] (a) Wolfe J. P., in Synthesis of Heterocycles via Metal-Catalyzed Reactions that Generate One or More Carbon-Heteroatom Bonds, 2013, ch. 98, pp. 1–37

[cit8] Kitamura M., Zaman S., Narasaka K. (2001). Synlett.

[cit9] Bingham M., Moutrille C., Zard S. Z. (2014). Heterocycles.

[cit10] See ESI†

[cit11] Other ligands were also explored but none prove effective

[cit12] Moreno-Mañas M., Pérez M., Pleixats R. (1996). J. Org. Chem..

[cit13] Inoue F., Kashihara M., Yadav M. R., Nakao Y. (2017). Angew. Chem., Int. Ed..

[cit14] Tokunaga Y., Ueno H., Shimomura Y., Seo T. (2002). Heterocycles.

[cit15] Veleckis E., Hacker D. S. (1984). J. Chem. Eng. Data.

[cit16] It is notable that N–N bond cleavage was not observed under these conditions

