# Supplementary material for: Tandem aza-Heck Suzuki and carbonylation reactions of O-phenyl hydroxamic ethers: complex lactams via carboamination
Source: Chem Sci. 2021 May 27;12(25):8859–64. doi: 10.1039/d1sc02075g (PMC8246078; doi:10.1039/d1sc02075g)

Electronic Supplementary Material (ESI) for Chemical Science.
This journal is © The Royal Society of Chemistry 2021

Tandem Aza-Heck Suzuki and Carbonylation Reactions of *O*-Phenyl Hydroxamic Ethers: Complex Lactams via Carboamination

Run-Duo Gao, Scott A. Shuler, and Donald A. Watson*

Department of Chemistry and Biochemistry, University of Delaware,

Newark, Delaware 19716, United States

Supporting Information

Table of Contents

1. General Experimental Details S3

2. Instrumentation and Chromatography S3

3. Synthesis of the *O*-Phenyl Hydroxamates S4

4. Synthesis of Lactams *via* Aza-Heck-Suzuki Reaction S9

5. Synthesis of Lactams *via* Aza-Heck-Carbonylation Reaction S23

6. Crystallographic Details S30

7. References S31

8. Spectroscopic Data S32

1. General Experimental Details. Toluene, dioxane, and acetonitrile were dried on alumina according to a published procedure.[1] Tris(2,2,2-trifluoroethyl) phosphite were purchased from Sigma-Aldrich, distilled from P_2O_5, sparged with N_2, and stored under N_2 in a sealed vessel. (1,5-cyclooctadiene)bis[(trimethylsilyl)methyl]palladium [(COD)Pd(CH_2SiMe_3)$_2$] was prepared according to literature procedure,[2] and stored in a sealed container of DrieRite at –30 °C when not in use. 4Å MS powder was activated at 150 °C under vacuum for 1d and stored in the oven when not in use. All hot glassware was oven dried for a minimum of two hours or flame-dried under vacuum prior to use. The following substrates were synthesized according to published procedures: 2-(prop-1-en-2-yl)benzoic acid;[3] 2-(1-phenylvinyl)benzoic acid;[3] 4-chloro-2-(prop-1-en-2-yl)benzoic acid;[3] 4-methylpent-4-enoic acid;[3] 2,2,4-trimethylpent-4-enoic acid;[3] 4-methyl-2-phenylpent-4-enoic acid;[3] 2-(1-cyclopropylvinyl)benzoic acid;[3] 2-(2'-methylallyl)benzoic acid;[3] 1-(2-methylallyl)cyclobutanecarboxylic acid;[4] 2-(2-methylenecyclopentyl)acetic acid;[5] (*Z*)-N-phenoxy-2-styrylbenzamide;[6] (*E*)-N-phenoxy-2-styrylbenzamide.[6] All other substrates and reagents were purchased in highest analytical purity from commercial suppliers and used as received. Reaction optimization was conducted in a glovebox (N_2 atmosphere) on a 0.2 mmol scale in 2-dram vials with Teflon lined caps and heated in an aluminum block with stirring. All NMR chemical yields are reported using 1,3,5-trimethoxybenzene as an internal standard. All other reactions were set up using standard Schlenk technique and heated with stirring in temperature-controlled oil baths.

2. Instrumentation and Chromatography

400 MHz ^{1}H, 100 MHz ^{13}C and 376 MHz ^{19}F spectra were obtained on a 400 MHz FT-NMR spectrometer equipped with a Bruker CryoPlatform. 600 MHz ^{1}H, 150 MHz ^{13}C, and 564 MHz ^{19}F spectra were obtained on a 600 MHz FT-NMR spectrometer equipped with a Bruker SMART probe. All samples were analyzed in the indicated deutero-solvent and were recorded at ambient temperatures. All chemical shifts are reported in ppm. ^{1}H NMR spectra were calibrated using the residual protio-signal in deutero-solvents as a standard. ^{13}C NMR spectra were calibrated using the deutero-solvent as a standard. IR spectra were recorded on a Nicolet Magma-IR 560 FT-IR spectrometer as thin films on KBr plates. High resolution MS data was obtained on a Thermo Q-Exactive Orbitrap using electrospray ionization (ESI). Unless otherwise noted, column chromatography was performed either by hand or by use of Isolera 4 Biotage unit with 40-63 μm silica gel, and the eluent reported in parentheses. Analytical thin-layer chromatography (TLC) was performed on precoated glass plates and visualized by UV or by staining ($KMnO_4$ or I_2).

3. Synthesis of the *O*-Phenyl Hydroxamates

Note: All yields in this section are unoptimized

General Protocol: A flame-dried round-bottom flask, equipped with magnetic stir bar and rubber septum was purged with a stream of nitrogen until cool. The septum was quickly removed, and the carboxylic acid (1.0 equiv) was added. Subsequent addition of anhydrous DMF (0.3 M) and diisopropylethyl amine (DIEA, 3.3 equiv) gave a clear-to-pale yellow solution, followed by replacement of the septa. The flask was cooled to 0 °C in an ice bath, and the septa was then briefly removed to allow for solid addition of *O*-(7-azabenzotriazol-1-yl)-*N,N,N',N'*-tetramethyluronium hexafluorophosphate (HATU, 1.1 equiv). The reaction was stirred at 0 °C for 30 minutes, and *O*-phenylhydroxylamine hydrochloride was added. The reaction was stirred at 0 °C for 4 hours, and then warm up to room temperature upon completion (as monitored by TLC), the reaction was quenched with brine (4X reaction volume) and diluted with EtOAc (4X reaction volume). The resulting biphasic mixture was poured into a separatory funnel and the layers were separated. The organic layer was washed with brine, dried with $MgSO_4$, filtered through a glass frit, and concentrated in vacuo to give crude product. The product was purified by flash column chromatography on silica gel (5-20 μm particle size), recrystallization, or both to yield pure product.

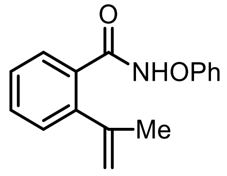

(**1**) According to the general protocol: 2-(prop-1-en-2-yl)benzoic acid (2.44 g, 15 mmol) and DIEA (8.2 mL, 49.5 mmol) were dissolved in DMF (45 mL) and stirred at 0 °C for 10 minutes. HATU (6.28 g, 16.5 mmol) was added, stirred at 0 °C for 30 minutes, and then *O*-phenylhydroxylamine hydrochloride (2.40 g, 16.5 mmol) was added. The reaction stirred for 4 hours, slowly warming to room temperature for overnight, and was worked up according to the general procedure. Purification by chromatography (linear gradient, 5:95 acetone:hexanes to 10:90 acetone:hexanes) and recrystallization in EtOAc afforded **1** (2.72 g, 72%) as a white solid. ^{1}H NMR (600 MHz, DMSO-*d*$_6$) δ 12.10 (s, 1H), 7.54-7.49 (m, 2H), 7.42-7.35 (m, 4H), 7.12 (d, *J* = 7.8 Hz, 2H), 7.06-7.04 (m, 1H), 5.18 (s, 1H), 4.97 (s, 1H), 2.09 (s, 3H); ^{13}C NMR (150 MHz, DMSO-*d*$_6$) δ 166.8, 159.6, 143.9, 142.0, 132.4, 130.0, 129.4, 128.1, 127.9, 127.0, 122.3, 115.4, 112.9, 23.8; FTIR (cm^{-1}): 3148, 2968, 1661, 1591, 1489, 1201, 901, 751; mp = 90-92 °C (acetone/hexanes); HRMS (ESI) m/z, calculated for [$C_{16}H_{16}NO_2^+$]: 254.1176; found: 254.1180.

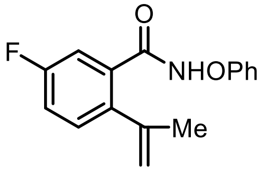

(**S1**) According to the general protocol: 5-fluoro-2-(prop-1-en-2-yl)benzoic acid (1.80 g, 10 mmol) and DIEA (5.5 mL, 33 mmol) were dissolved in DMF (30 mL) and stirred at 0 °C for 10 minutes. HATU (4.18 g, 11 mmol) was added, stirred at 0 °C for 30 minutes, and then *O*-phenylhydroxylamine hydrochloride (1.60 g, 11 mmol) was added. The reaction stirred for 4 hours, slowly warming to room temperature for overnight, and was worked up according to the general procedure. Purification by chromatography (linear gradient, 5:95 EtOAc:hexanes to 40:60 EtOAc:hexanes) afforded **S1** (2.50 g, 92%) as a white solid. ^{1}H NMR (600 MHz, DMSO-*d*$_6$) δ 12.21 (s, 1H), 7.45-7.41 (m, 2H), 7.38-7.33 (m, 3H), 7.14 (d, *J* = 8.4 Hz, 2H), 7.07-7.05 (m, 1H), 5.19 (s, 1H), 4.97 (s, 1H), 2.08 (s, 3H); ^{19}F NMR (565 MHz, DMSO-*d*$_6$) δ -114.9 (m); ^{13}C NMR (150 MHz, DMSO-*d*$_6$) δ 165.4, 160.5 (d, *J* = 243.9 Hz), 159.5, 143.0, 138.4, 134.2 (d, *J* = 6.6 Hz), 130.3 (d, *J* = 7.8 Hz), 129.4, 122.4, 116.9 (d, *J* = 20.7 Hz), 115.8, 114.8 (d, *J* = 30.0 Hz), 113.0, 23.9;

(FTIR cm^{-1}): 3144, 2970, 1662, 1591, 1490, 1215, 1161, 751, 688; mp = 93-95 °C (EtOAc:hexanes); HRMS (ESI) m/z, calculated for [$C_{16}H_{15}FNO_2^+$]: 272.1081; found: 272.1074.

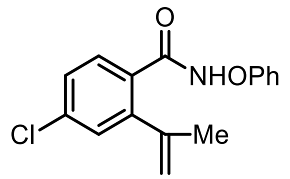

(**S2**) According to the general protocol: 4-chloro-2-(prop-1-en-2-yl)benzoic acid (1.47 g, 7.5 mmol) and DIEA (4.1 mL, 24.8 mmol) were dissolved in DMF (20 mL) and stirred at 0 °C for 10 minutes. HATU (3.14 g, 8.25 mmol) was added, stirred at 0 °C for 30 minutes, and then *O*-phenylhydroxylamine hydrochloride (1.21 g, 8.25 mmol) was added. The reaction stirred for 4 hours, slowly warming to room temperature for overnight, and was worked up according to the general procedure. Purification by chromatography (linear gradient, 4:96 EtOAc:hexanes to 30:70 EtOAc:hexanes) afforded **S2** (1.34 g, 63%) as a white solid. ^{1}H NMR (600 MHz, DMSO-d_6) δ 12.18 (s, 1H), 7.58 (d, J = 7.8 Hz, 1H), 7.49-7.45 (m, 2H), 7.37-7.35 (m, 2H), 7.11 (d, J = 7.8 Hz, 2H), 7.07-7.04 (m, 1H), 5.22 (s, 1H), 5.01 (s, 1H), 2.09 (s, 3H); ^{13}C NMR (150 MHz, DMSO-d_6) δ 165.8, 159.5, 144.1, 142.8, 134.7, 131.2, 129.9, 129.4, 127.9, 127.0, 122.4, 116.3, 112.9, 23.5; FTIR (cm^{-1}): 3141, 2949, 1661, 1590, 1489, 1200, 901, 751; mp = 130-133 °C (EtOAc:hexanes); HRMS (ESI) m/z, calculated for [$C_{16}H_{15}NClO_2^+$]: 288.0786; found: 288.0791.

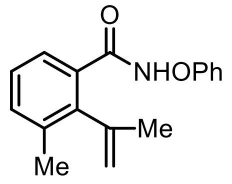

(**S3**) According to the general protocol: 3-methyl-2-(prop-1-en-2-yl)benzoic acid (1.76 g, 10 mmol) and DIEA (5.5 mL, 33 mmol) were dissolved in DMF (30 mL) and stirred at 0 °C for 10 minutes. HATU (4.18 g, 11 mmol) was added, stirred at 0 °C for 30 minutes, and then *O*-phenylhydroxylamine hydrochloride (1.60 g, 11 mmol) was added. The reaction stirred for 4 hours, slowly warming to room temperature for overnight, and was worked up according to the general procedure. Purification by chromatography (linear gradient, 3:97 EtOAc:hexanes to 30:70 EtOAc:hexanes) afforded **S3** (2.23 g, 83%) as a white solid. ^{1}H NMR (600 MHz, DMSO-d_6) δ 12.02 (s, 1H), 7.37-7.34 (m, 4H), 7.30-7.28 (m, 1H), 7.10 (d, J = 6.6 Hz, 2H), 7.06-7.03 (m, 1H), 5.26 (s, 1H), 4.82 (s, 1H), 2.28 (s, 3H), 2.03 (s, 3H); ^{13}C NMR (150 MHz, DMSO-d_6) δ 166.7, 159.6, 144.0, 141.4, 135.2, 133.2, 131.5, 129.4, 126.6, 124.8, 122.2, 115.3, 112.8, 24.2, 18.9; (FTIR cm^{-1}): 3152, 2952, 1662, 1591, 1489, 1208, 751, 688; mp = 115-117 °C (EtOAc:hexanes); HRMS (ESI) m/z, calculated for [$C_{17}H_{18}NO_2^+$]: 268.1332; found: 268.1337.

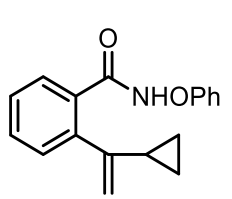

(**S4**) According to the general protocol: 2-(1-cyclopropylvinyl)benzoic acid (1.19 g, 6 mmol) and DIEA (3.3 mL, 19.8 mmol) were dissolved in DMF (20 mL) and stirred at 0 °C for 10 minutes. HATU (2.51 g, 6.6 mmol) was added, stirred at 0 °C for 30 minutes, and then *O*-phenylhydroxylamine hydrochloride (0.96 g, 6.6 mmol) was added. The reaction stirred for 4 hours, slowly warming to room temperature for overnight, and was worked up according to the general procedure. Purification by chromatography (linear gradient, 5:95 EtOAc:hexanes to 35:65 EtOAc:hexanes) afforded **S4** (1.55 g, 93%) as a white solid. ^{1}H NMR (600 MHz, DMSO-d_6) δ 12.05 (s, 1H), 7.54-7.48 (m, 2H), 7.42-7.34 (m, 4H), 7.12 (d, J = 7.8 Hz, 2H), 7.06-7.03 (m, 1H), 5.06 (s, 1H),

4.93 (s, 1H), 1.69-1.65 (m, 1H), 0.74-0.70 (m, 2H), 0.58-0.55 (m, 2H); ^{13}C NMR (150 MHz, DMSO-d_6) δ 166.6, 159.6, 149.0, 141.1, 132.9, 129.8, 129.4, 128.7, 127.8, 127.0, 122.3, 113.0, 111.9, 17.1, 6.7; FTIR (cm^{-1}): 3151, 3007, 1662, 1592, 1489, 1202, 902, 751, 688; mp = 85-87 °C (EtOAc:hexanes); HRMS (ESI) m/z, calculated for [$C_{18}H_{18}NO_2^+$]: 280.1332; found: 280.1337.

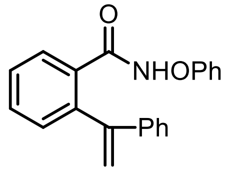

(**S5**) According to the general protocol: 2-(1-phenylvinyl)benzoic acid (2.24 g, 10 mmol) and DIEA (5.5 mL, 33 mmol) were dissolved in DMF (30 mL) and stirred at 0 °C for 10 minutes. HATU (4.18 g, 11 mmol) was added, stirred at 0 °C for 30 minutes, and then *O*-phenylhydroxylamine hydrochloride (1.60 g, 11 mmol) was added. The reaction stirred for 4 hours, slowly warming to room temperature for overnight, and was worked up according to the general procedure. Purification by chromatography (linear gradient, 6:94 EtOAc:hexanes to 50:50 EtOAc:hexanes) afforded **S5** (2.60 g, 83%) as a white solid. ^{1}H NMR (600 MHz, DMSO-d_6) δ 12.02 (d, *J* = 7.2 Hz, 1H), 7.62-7.20 (m, 11H), 6.96-6.74 (m, 3H), 5.77 (d, *J* = 14.4 Hz, 1H), 5.26 (d, *J* = 12.0 Hz, 1H); ^{13}C NMR (150 MHz, DMSO-d_6) δ 166.1, 159.4, 147.4, 140.6, 139.9, 133.4, 130.5, 130.3, 129.3, 128.2, 128.0, 127.8, 127.6, 126.8, 122.1, 115.6, 112.7; FTIR (cm^{-1}): 3163, 2945, 1663, 1591, 1489, 1201, 902, 771; mp = 115-118 °C (EtOAc:hexanes); HRMS (ESI) m/z, calculated for [$C_{21}H_{18}NO_2^+$]: 316.1332; found: 316.1338.

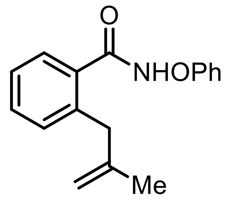

(**S6**) According to the general protocol: 2-(2-methylallyl)benzoic acid (1.76 g, 10 mmol) and DIEA (5.5 mL, 33 mmol) were dissolved in DMF (30 mL) and stirred at 0 °C for 10 minutes. HATU (4.18 g, 11 mmol) was added, stirred at 0 °C for 30 minutes, and then *O*-phenylhydroxylamine hydrochloride (1.60 g, 11 mmol) was added. The reaction stirred for 4 hours, slowly warming to room temperature for overnight, and was worked up according to the general procedure. Purification by chromatography (linear gradient, 5:95 EtOAc:hexanes to 35:65 EtOAc:hexanes) afforded **S6** (1.91 g, 72%) as a white solid. ^{1}H NMR (600 MHz, DMSO-d_6) δ 12.21 (brs, 1H), 7.56-7.46 (m, 2H), 7.36-7.31 (m, 4H), 7.12-7.03 (m, 3H), 4.83 (s, 1H), 4.55 (s, 1H), 1.67 (s, 3H); ^{13}C NMR (150 MHz, DMSO-d_6) δ 159.6, 144.5, 138.0, 133.2, 130.5, 130.3, 129.4, 127.9, 126.2, 122.3, 112.9, 112.1, 40.0, 22.4; FTIR (cm^{-1}): 3151, 2968, 1659, 1591, 1489, 1201, 898, 749; mp = 93-96 °C (EtOAc:hexanes); HRMS (ESI) m/z, calculated for [$C_{17}H_{18}NO_2^+$]: 268.1332; found: 268.1337.

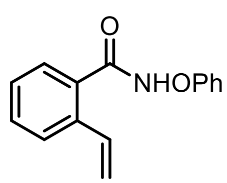

(**S7**) According to the general protocol: 2-vinylbenzoic acid (1.48 g, 10 mmol) and DIEA (5.5 mL, 33 mmol) were dissolved in DMF (30 mL) and stirred at 0 °C for 10 minutes. HATU (4.18 g, 11 mmol) was added, stirred at 0 °C for 30 minutes, and then *O*-phenylhydroxylamine hydrochloride (1.60 g, 11 mmol) was added. The reaction stirred for 4 hours, slowly warming to room temperature for overnight, and was worked up according to the general procedure. Purification by chromatography (linear gradient, 6:94 EtOAc:hexanes to 42:58 EtOAc:hexanes) afforded **S7** (2.07 g, 87%) as a white solid. ^{1}H NMR (600 MHz, DMSO-d_6) δ 12.30 (s, 1H), 7.78 (d, *J* = 7.8 Hz, 1H), 7.58 (d, *J* = 7.2 Hz, 1H), 7.55-7.53 (m, 1H), 7.43-7.37 (m, 3H), 7.14 (d, *J* = 7.2 Hz, 2H), 7.08-

7.06 (m, 1H), 7.02-6.97 (m, 1H), 5,89 (d, *J* = 17.4 Hz), 5.40 (d, *J* = 10.8 Hz); ^{13}C NMR (150 MHz, DMSO-d_6) δ 166.1, 159.5, 135.6, 133.4, 132.2, 130.5, 129.5, 127.9, 127.7, 125.5, 122.4, 116.9, 112.9; FTIR (cm^{-1}): 3145, 2970, 1674, 1595, 1202, 904, 770, 689; mp = 111-114 °C (EtOAc:hexanes); HRMS (ESI) m/z, calculated for [$C_{15}H_{14}NO_2^+$]: 240.1019; found: 240.1023.

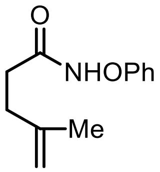

(**S8**) According to the general protocol: 4-methylpent-4-enoic acid (2.28 g, 20 mmol) and DIEA (11.0 mL, 66 mmol) were dissolved in DMF (60 mL) and stirred at 0 °C for 10 minutes. HATU (8.37 g, 22 mmol) was added, stirred at 0 °C for 30 minutes, and then *O*-phenylhydroxylamine hydrochloride (3.2 g, 22 mmol) was added. The reaction stirred for 4 hours, slowly warming to room temperature for overnight, and was worked up according to the general procedure. Purification by chromatography (linear gradient, 6:94 EtOAc:hexanes to 60:40 EtOAc:hexanes) and recrystallization in EtOAc afforded **S8** (1.76 g, 43%) as a white solid. ^{1}H NMR (600 MHz, DMSO-d_6) δ 11.71 (s, 1H), 7.32-7.30 (m, 2H), 7.02-6.99 (m, 3H), 4.76 (s, 1H), 4.73 (s, 1H), 2.32-2.30 (m, 4H), 1.73 (s, 3H); ^{13}C NMR (150 MHz, DMSO-d_6) δ 170.0, 160.0, 144.7, 129.8, 122.7, 113.3, 111.0, 33.0, 31.0, 22.7; FTIR (cm^{-1}): 3112, 2904, 1659, 1527, 880, 744, 688; mp = 133-135 °C (EtOAc:hexanes); HRMS (ESI) m/z, calculated for [$C_{12}H_{16}NO_2^+$]: 206.1176; found: 206.1181.

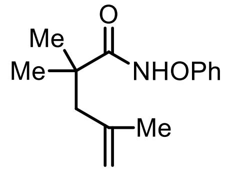

(**S9**) According to the general protocol: 2,2,4-trimethylpent-4-enoic acid (1.42 g, 10 mmol) and DIEA (5.5 mL, 33 mmol) were dissolved in DMF (30 mL) and stirred at 0 °C for 10 minutes. HATU (4.18 g, 11 mmol) was added, stirred at 0 °C for 30 minutes, and then *O*-phenylhydroxylamine hydrochloride (1.60 g, 11 mmol) was added. The reaction stirred for 4 hours, slowly warming to room temperature for overnight, and was worked up according to the general procedure. Purification by chromatography (linear gradient, 5:95 EtOAc:hexanes to 35:65 EtOAc:hexanes) afforded **S9** (2.12 g, 91%) as a white solid. ^{1}H NMR (600 MHz, DMSO-d_6) δ 11.59 (s, 1H), 7.33-7.30 (m, 2H), 7.02-6.98 (m, 3H), 4.83 (s, 1H), 4.70 (s, 1H), 2.30 (s, 2H), 1.67 (s, 3H), 1.18 (s, 6H); ^{13}C NMR (150 MHz, DMSO-d_6) δ 174.6, 159.7, 142.2, 129.3, 122.0, 114.2, 112.8, 47.4, 40.8, 25.3, 23.7; FTIR (cm^{-1}): 3155, 2970, 1656, 1589, 1499, 1205, 755; mp = 84-86 °C (EtOAc:hexanes); HRMS (ESI) m/z, calculated for [$C_{14}H_{20}NO_2^+$]: 234.1489; found: 234.1494.

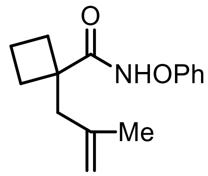

(**S10**) According to the general protocol: 4-methyl-2-phenylpent-4-enoic acid (1.52 g, 10 mmol) and DIEA (5.5 mL, 33 mmol) were dissolved in DMF (30 mL) and stirred at 0 °C for 10 minutes. HATU (4.18 g, 11 mmol) was added, stirred at 0 °C for 30 minutes, and then *O*-phenylhydroxylamine hydrochloride (1.60 g, 11 mmol) was added. The reaction stirred for 4 hours, slowly warming to room temperature for overnight, and was worked up according to the general procedure. Purification by chromatography (linear gradient, 5:95 EtOAc:hexanes to 40:60 EtOAc:hexanes) afforded **S10** (2.11 g, 90%) as a white solid. ^{1}H NMR (600 MHz, DMSO-d_6) δ 11.62 (s, 1H), 7.32-7.30 (m, 2H), 7.01-7.00 (m, 3H), 4.77 (s, 1H), 4.63 (s, 1H), 2.57 (s, 2H), 2.44-2.42 (m, 2H), 1.96-1.82 (m, 4H), 1.67 (s, 3H); ^{13}C NMR (150 MHz, DMSO-d_6) δ 173.6, 159.8, 142.3, 129.3, 122.0, 112.8, 112.3, 46.0, 45.1, 30.3, 23.2, 15.7; FTIR (cm^{-1}): 3192, 2969, 1657, 1592, 1488, 1197, 750, 688; mp = 73-75 °C (EtOAc:hexanes); HRMS (ESI) m/z,

calculated for [$C_{15}H_{20}NO_2^+$]: 246.1489; found: 246.1493.

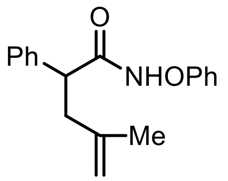

(**S11**) According to the general protocol: 4-methyl-2-phenylpent-4-enoic acid (1.90 g, 10 mmol) and DIEA (5.5 mL, 33 mmol) were dissolved in DMF (30 mL) and stirred at 0 °C for 10 minutes. HATU (4.18 g, 11 mmol) was added, stirred at 0 °C for 30 minutes, and then *O*-phenylhydroxylamine hydrochloride (1.60 g, 11 mmol) was added. The reaction stirred for 4 hours, slowly warming to room temperature for overnight, and was worked up according to the general procedure. Purification by chromatography (linear gradient, 3:97 EtOAc:hexanes to 30:70 EtOAc:hexanes) and recrystallization in EtOAc afforded **S11** (1.85 g, 66%) as a white solid. ^{1}H NMR (600 MHz, DMSO-d_6) δ 12.03 (s, 1H), 7.40-7.34 (m, 4H), 7.29-723 (m, 3H), 6.99-6.97 (m, 1H), 6.85 (d, *J* = 7.8 Hz, 2H), 4.79 (s, 1H), 4.74 (s, 1H), 3.75-3.72 (m, 1H), 2.79 (dd, *J* = 13.8, 9.6 Hz, 1H), 2.39 (dd, *J* = 14.4, 5.4 Hz, 1H), 1.74 (s, 3H); ^{13}C NMR (150 MHz, DMSO-d_6) δ 169.6, 159.4, 142.5, 139.4, 129.3, 128.3, 127.6, 127.0, 122.3, 112.6, 112.3, 46.6, 40.1, 22.3; FTIR (cm^{-1}): 3158, 2969, 1665, 1591, 1488, 1161, 752, 698; mp = 124-127 °C (EtOAc:hexanes); HRMS (ESI) m/z, calculated for [$C_{18}H_{20}NO_2^+$]: 282.1489; found: 282.1493.

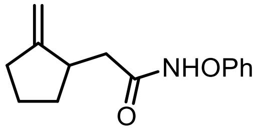

(**S12**) According to the general protocol: 2-(2-methylenecyclopentyl)acetic acid (1.20 g, 8.55 mmol) and DIEA (4.7 mL, 28.2 mmol) were dissolved in DMF (25 mL) and stirred at 0 °C for 10 minutes. HATU (3.58 g, 9.4 mmol) was added, stirred at 0 °C for 30 minutes, and then *O*-phenylhydroxylamine hydrochloride (1.37 g, 9.4 mmol) was added. The reaction stirred for 4 hours, slowly warming to room temperature for overnight, and was worked up according to the general procedure. Purification by chromatography (linear gradient, 6:94 EtOAc:hexanes to 50:50 EtOAc:hexanes) afforded **S12** (1.67 g, 86%) as a white solid. ^{1}H NMR (600 MHz, DMSO-d_6) δ 11.71 (s, 1H), 7.34-7.31 (m, 2H), 7.03-6.99 (m, 3H), 4.90 (s, 1H), 4.86 (s, 1H), 2.78-2.67 (m, 1H), 2.46 (dd, *J* = 14.4, 5.4 Hz, 1H), 2.32-2.78 (m, 2H), 2.09 (dd, *J* = 14.4, 9.0 Hz, 1H), 1.92-1.87 (m, 1H), 1.70-1.68 (m, 1H), 1.56-1.50 (m, 1H), 1.38-1.32 (m, 1H); ^{13}C NMR (150 MHz, DMSO-d_6) δ 169.1, 159.5, 154.9, 129.4, 122.2, 112.8, 105.1, 40.0, 37.0, 32.5, 32.3, 23.5; FTIR (cm^{-1}): 3146, 2954, 1659, 1592, 1488, 1207, 1158, 748, 688; mp = 98-100 °C (EtOAc:hexanes); HRMS (ESI) m/z, calculated for [$C_{14}H_{18}NO_2^+$]: 232.1332; found: 232.1337.

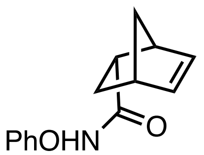

(**35**) According to the general protocol: 4-methyl-2-phenylpent-4-enoic acid (1.52 g, 10 mmol) and DIEA (5.5 mL, 33 mmol) were dissolved in DMF (30 mL) and stirred at 0 °C for 10 minutes. HATU (4.18 g, 11 mmol) was added, stirred at 0 °C for 30 minutes, and then *O*-phenylhydroxylamine hydrochloride (1.60 g, 11 mmol) was added. The reaction stirred for 4 hours, slowly warming to room temperature for overnight, and was worked up according to the general procedure. Purification by chromatography (linear gradient, 6:94 EtOAc:hexanes to 70:30 EtOAc:hexanes) and recrystallization in EtOAc afforded afforded **35** (1.15 g, 50%) as a white solid. ^{1}H NMR (600 MHz, DMSO-d_6) δ 11.59 (s, 1H), 7.33-7.30 (m, 2H), 7.01-6.95 (m, 3H), 6.18 (s, 1H), 5.93 (s, 1H), 3.24 (s, 1H),

2.93-2.87 (m, 2H), 1.87-1.82 (m, 1H), 1.36-1.31 (m, 3H); ^{13}C NMR (150 MHz, DMSO-d_6) δ 171.3, 159.7, 137.3, 132.0, 129.3, 122.0, 112.7, 49.4, 45.6, 41.9, 40.8, 28.2; mp = 170-172 °C (EtOAc:hexanes); FTIR (cm^{-1}): 3164, 2970, 1668, 1513, 1189, 750, 689; HRMS (ESI) m/z, calculated for [$C_{14}H_{16}NO_2^+$]: 230.1176; found: 230.1179.

4. Synthesis of Lactams *via* Aza-Heck-Suzuki Reaction

4.1. Reaction Optimization [a]

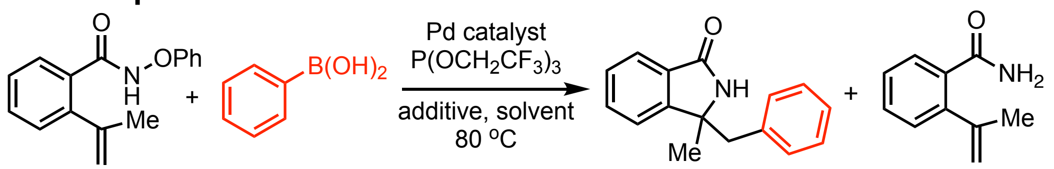

1 2 3 4

Entry	Pd Catalyst [mol %]	Solvent	Additive	Yield [%] [b]
1	(COD)Pd(CH_2SiMe_3)$_2$ [10]	PhMe	--	18
2	(COD)Pd(CH_2SiMe_3)$_2$ [10]	Dioxane	--	15
3	(COD)Pd(CH_2SiMe_3)$_2$ [10]	*t*-BuOH	--	55
4	(COD)Pd(CH_2SiMe_3)$_2$ [10]	MeCN	--	74
5	(COD)Pd(CH_2SiMe_3)$_2$ [10]	MeCN	4Å MS	81
6	(COD)Pd(CH_2SiMe_3)$_2$ [10]	MeCN	5Å MS	59
7	(COD)Pd(CH_2SiMe_3)$_2$ [10]	MeCN	$MgSO_4$	35
8	Pd(acac)$_2$ [10]	MeCN	4Å MS	72
9	Pd(MeCN)Cl_2 [10]	MeCN	4Å MS	54
10	Pd(OAc)$_2$ [10]	MeCN	4Å MS	81
11[c]	Pd(OAc)$_2$ [5]	MeCN	4Å MS	81

[a] Unless otherwise noted, reactions run with 0.2 mmol **1** and 0.6 mmol **2** at 0.05 M. [b] Yield calculated by ^1H NMR with 1,3,5-trimethoxybenzene as internal standard. [c] 5 mol% Pd(OAc)$_2$ at 0.1 M.

4.2. Synthesis of Lactams *via* Aza-Heck-Suzuki Reaction

General Protocol: A flame-dried Schlenk flask equipped with a magnetic stir bar and rubber septum was attached to a double manifold and cooled under vacuum. The flask was backfilled with N_2, the septum was removed and Pd(OAc)$_2$ or (COD)Pd(CH_2SiMe_3)$_2$ (0.05-0.20 equiv), hydroxamate (1.0 mmol, 1.0 equiv), boronic acid (3.0 mmol, 3.0 equiv) and 4Å MS (500 mg) were added to the flask. The septum was replaced, and the flask was evacuated and backfilled with nitrogen four times. P(OCH_2CF_3)$_3$ (0.15-0.6 equiv), and anhydrous MeCN (10.0 mL) were added sequentially to the flask via syringe. The resulting solution was heated in an oil bath with rapid stirring at the indicated temperature for 24 h. Upon completion, the reaction was cooled to room temperature, opened to air, and the reaction mixture was then diluted with EtOAc, filtered through celite. The

suspension was adsorbed onto Celite via rotary evaporation. The resulting powder was directly chromatographed on silica gel (5-20 µm particle size) to yield the desired product.

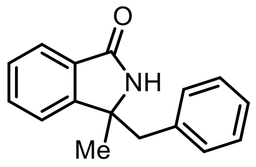

(**3**) According to the general protocol: $Pd(OAc)_2$ (11.3 mg, 0.05 mmol, 0.05 equiv), N-phenoxy-2-(prop-1-en-2-yl)benzamide **1** (253.3 mg, 1.00 mmol), phenylboronic acid (366 mg, 3.0 mmol, 3.0 equiv), 4Å MS (500 mg), $P(OCH_2CF_3)_3$ (49.2 mg, 0.15 mmol, 0.15 equiv), and anhydrous MeCN (10.0 mL) were combined under N_2. The reaction was stirred at 80 °C for 24 h under N_2 environment. The reaction was worked up according to the general procedure and purified by chromatography (linear gradient, 12:88 EtOAc:hexanes to 100:0 EtOAc:hexanes) to afford amide **3** (209.8 mg, 88%) as a white solid. ^1H NMR (600 MHz, $CDCl_3$) δ 7.76 (d, *J* = 7.8 Hz, 1H), 7.59-7.57 (m, 1H), 7.45-7.41 (m, 2H), 7.22-7.21 (m, 3H), 7.08-7.07 (m, 2H), 6.72 (brs, 1H), 3.12 (d, *J* = 13.8 Hz, 1H), 2.97 (d, *J* = 13.8 Hz, 1H), 1.53 (s, 3H); ^{13}C NMR (150 MHz, $CDCl_3$) δ 169.4, 151.7, 135.8, 131.9, 131.1, 130.3, 128.2, 128.1, 127.0, 123.9, 121.4, 61.8, 46.7, 25.4; FTIR (cm^{-1}): 3212, 2917, 1694, 1469, 1455, 1352, 700; mp = 148-150 °C (EtOAc:hexanes); HRMS (ESI) m/z, calculated for $[C_{16}H_{16}NO^+]$: 238.1226; found: 238.1220.

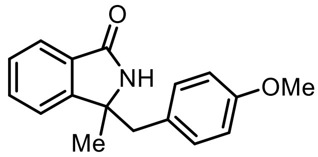

(**5**) According to the general protocol: $Pd(OAc)_2$ (11.3 mg, 0.05 mmol, 0.05 equiv), N-phenoxy-2-(prop-1-en-2-yl)benzamide **1** (253.3 mg, 1.00 mmol), 4-methoxyphenylboronic acid (366 mg, 3.0 mmol, 3.0 equiv), 4Å MS (500 mg), $P(OCH_2CF_3)_3$ (49.2 mg, 0.15 mmol, 0.15 equiv), and anhydrous MeCN (10.0 mL) were combined under N_2. The reaction was stirred at 80 °C for 24 h under N_2 environment. The reaction was worked up according to the general procedure and purified by chromatography (linear gradient, 12:88 EtOAc:hexanes to 100:0 EtOAc:hexanes) to afford amide **5** (212.8 mg, 80%) as a white solid. ^1H NMR (600 MHz, $CDCl_3$) δ 7.98 (s, 1H), 7.63 (d, *J* = 7.2 Hz, 1H), 7.46-7.43 (m, 1H), 7.31-7.27 (m, 2H), 6.80 (d, *J* = 8.4 Hz, 2H), 6.54 (d, *J* = 8.4 Hz, 2H), 3.57 (s, 3H), 2.97 (d, *J* = 13.8 Hz, 1H), 2.90 (d, *J* = 13.8 Hz, 1H), 1.46 (s, 3H); ^{13}C NMR (150 MHz, $CDCl_3$) δ 170.2, 158.4, 151.6, 131.7, 131.6, 131.3, 128.0, 127.9, 123.7, 121.6, 113.4, 62.5, 55.1, 45.7, 25.7; FTIR (cm^{-1}): 3230, 1693, 1612, 1513, 1249, 1179, 756; mp = 143-145 °C (EtOAc:hexanes); HRMS (ESI) m/z, calculated for $[C_{17}H_{18}NO_2^+]$: 268.1332; found: 268.1336.

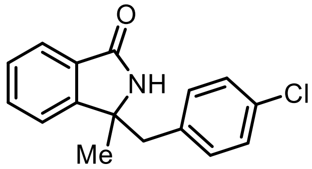

(**6**) According to the general protocol: $Pd(OAc)_2$ (11.3 mg, 0.05 mmol, 0.05 equiv), N-phenoxy-2-(prop-1-en-2-yl)benzamide **1** (253.3 mg, 1.00 mmol), 4-chlorophenylboronic acid (366 mg, 3.0 mmol, 3.0 equiv), 4Å MS (500 mg), $P(OCH_2CF_3)_3$ (49.2 mg, 0.15 mmol, 0.15 equiv), and anhydrous MeCN (10.0 mL) were combined under N_2. The reaction was stirred at 80 °C for 24 h under N_2 environment. The reaction was worked up according to the general procedure and purified by chromatography (linear gradient, 12:88 EtOAc:hexanes to 100:0 EtOAc:hexanes) to afford amide **6** (233 mg, 86%) as a white solid. ^1H NMR (600 MHz, $CDCl_3$) δ 8.18 (s, 1H), 7.71 (d, *J* = 7.5 Hz, 1H), 7.58-7.56 (t, *J* = 7.8 Hz, 1H), 7.43-7.40 (m, 2H), 7.04 (d, *J* = 8.4 Hz, 2H), 6.88 (d, *J* = 8.4 Hz, 2H), 3.12 (d, *J* = 13.8 Hz, 1H), 3.04 (d, *J* = 13.8 Hz, 1H), 1.58 (s, 3H); ^{13}C NMR (150 MHz, $CDCl_3$) δ 170.2, 151.0, 134.2, 132.7, 131.8, 131.6, 128.2,

128.1, 123.8, 121.5, 62.3, 45.8, 25.9; FTIR (cm^{-1}): 3215, 1693, 1492, 1352, 1319, 1147, 1016, 762; mp = 155-158 °C (EtOAc:hexanes); HRMS (ESI) m/z, calculated for [$C_{16}H_{15}ClNO^+$]: 272.0837; found: 272.0840.

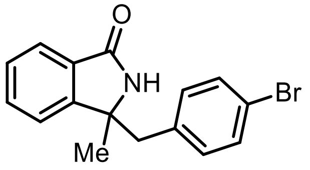

(**7**) According to the general protocol: $Pd(OAc)_2$ (11.3 mg, 0.05 mmol, 0.05 equiv), N-phenoxy-2-(prop-1-en-2-yl)benzamide **1** (253.3 mg, 1.00 mmol), 4-bromophenylboronic acid (600 mg, 3.0 mmol, 3.0 equiv), 4Å MS (500 mg), $P(OCH_2CF_3)_3$ (49.2 mg, 0.15 mmol, 0.15 equiv), and anhydrous MeCN (10.0 mL) were combined under N_2. The reaction was stirred at 80 °C for 24 h under N_2 environment. The reaction was worked up according to the general procedure and purified by chromatography (linear gradient, 12:88 EtOAc:hexanes to 100:0 EtOAc:hexanes) to afford amide **7** (230 mg, 73%) as a white solid. ^{1}H NMR (600 MHz, $CDCl_3$) δ 7.82 (s, 1H), 7.72 (d, *J* = 7.2 Hz, 1H), 7.59-7.57 (m, 1H), 7.44-7.40 (m, 2H), 7.23 (d, *J* = 8.2 Hz, 2H), 6.84 (d, *J* = 8.2 Hz, 2H), 3.10 (d, *J* = 13.2 Hz, 1H), 3.01 (d, *J* = 13.2 Hz, 1H), 1.57 (s, 3H); ^{13}C NMR (150 MHz, $CDCl_3$) δ 170.0, 151.1, 134.7, 131.94, 131.90, 131.4, 131.1, 128.3, 123.9, 121.5, 120.9, 62.1, 45.9, 25.9; FTIR (cm^{-1}): 3217, 2972, 1694, 1592, 1405, 1351, 762, 716; mp = 171-173 °C (EtOAc:hexanes); HRMS (ESI) m/z, calculated for [$C_{16}H_{15}BrNO^+$]: 316.0332; found: 316.0338.

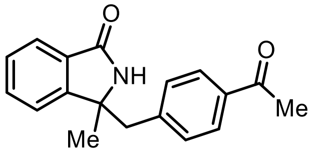

(**8**) According to the general protocol: $Pd(OAc)_2$ (11.3 mg, 0.05 mmol, 0.05 equiv), N-phenoxy-2-(prop-1-en-2-yl)benzamide **1** (253.3 mg, 1.00 mmol), 4-acetylphenylboronic acid (492 mg, 3.0 mmol, 3.0 equiv), 4Å MS (500 mg), $P(OCH_2CF_3)_3$ (49.2 mg, 0.15 mmol, 0.15 equiv), and anhydrous MeCN (10.0 mL) were combined under N_2. The reaction was stirred at 80 °C for 24 h under N_2 environment. The reaction was worked up according to the general procedure and purified by chromatography (linear gradient, 12:88 EtOAc:hexanes to 100:0 EtOAc:hexanes) to afford amide **8** (254.5 mg, 91%) as a white solid. ^{1}H NMR (400 MHz, $CDCl_3$) δ 8.73 (s, 1H), 7.64 (d, *J* = 7.6 Hz, 1H), 7.59 (d, *J* = 8.0 Hz, 2H), 7.56-7.52 (m, 1H), 7.43 (d, *J* = 7.6 Hz, 1H), 7.39-7.35 (m, 1H), 6.98 (d, *J* = 8.0 Hz, 2H), 3.21 (d, *J* = 13.6 Hz, 1H), 3.13 (d, *J* = 13.2 Hz, 1H), 2.40 (s, 3H), 1.58 (s, 3H); ^{13}C NMR (100 MHz, $CDCl_3$) δ 197.9, 170.3, 150.8, 141.4, 135.4, 131.9, 131.6, 130.5, 128.2, 127.8, 123.6, 121.6, 62.4, 46.1, 26.5, 26.2; FTIR (cm^{-1}): 3222, 2973, 1696, 1607, 1357, 1269, 766, 699; mp = 173-175 °C (EtOAc:hexanes); HRMS (ESI) m/z, calculated for [$C_{18}H_{18}NO_2^+$]: 280.1332; found: 280.1336.

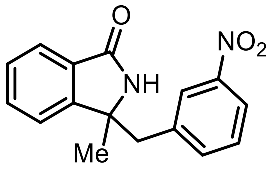

(**9**) According to the general protocol: $Pd(OAc)_2$ (11.3 mg, 0.05 mmol, 0.05 equiv), N-phenoxy-2-(prop-1-en-2-yl)benzamide **1** (253.3 mg, 1.00 mmol), 3-nitrophenylboronic acid (501 mg, 3.0 mmol, 3.0 equiv), 4Å MS (500 mg), $P(OCH_2CF_3)_3$ (49.2 mg, 0.15 mmol, 0.15 equiv), and anhydrous MeCN (10.0 mL) were combined under N_2. The reaction was stirred at 80 °C for 24 h under N_2 environment. The reaction was worked up according to the general procedure and purified by chromatography (linear gradient, 12:88 EtOAc:hexanes to 100:0 EtOAc:hexanes) to afford amide **9** (195.2 mg, 69%) as a white solid. ^{1}H NMR (600 MHz, $CDCl_3$) δ 8.71 (s,

1H), 7.87 (d, J = 7.8 Hz, 1H), 7.76 (s, 1H), 7.57-7.52 (m, 2H), 7.42 (d, J = 7.2 Hz, 1H), 7.35-7.33 (m, 1H), 7.20 (d, J = 7.2 Hz, 1H), 7.16-7.13 (m, 1H), 3.23 (d, J = 13.8 Hz, 1H), 3.14 (d, J = 13.8 Hz, 1H), 1.58 (s, 3H); ^{13}C NMR (150 MHz, $CDCl_3$) δ 170.5, 150.4, 147.7, 137.6, 136.4, 132.1, 131.5, 128.6, 128.5, 125.2, 123.8, 121.8, 121.4, 62.4, 45.8, 26.0; FTIR (cm^{-1}): 3209, 2974, 1694, 1526, 1350, 764, 700; mp = 165-167 °C (EtOAc:hexanes); HRMS (ESI) m/z, calculated for [$C_{16}H_{15}N_2O_3^+$]: 283.1077; found: 283.1080.

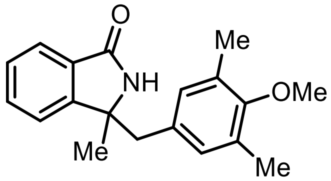

(**10**) According to the general protocol: $Pd(OAc)_2$ (11.3 mg, 0.05 mmol, 0.05 equiv), N-phenoxy-2-(prop-1-en-2-yl)benzamide **1** (253.3 mg, 1.00 mmol), (4-methoxy-3,5-dimethylphenyl)boronic acid (540 mg, 3.0 mmol, 3.0 equiv), 4Å MS (500 mg), $P(OCH_2CF_3)_3$ (49.2 mg, 0.15 mmol, 0.15 equiv), and anhydrous MeCN (10.0 mL) were combined under N_2. The reaction was stirred at 80 °C for 24 h under N_2 environment. The reaction was worked up according to the general procedure and purified by chromatography (linear gradient, 12:88 EtOAc:hexanes to 100:0 EtOAc:hexanes) to afford amide **10** (173.5 mg, 59%) as a white solid. ^{1}H NMR (600 MHz, $CDCl_3$) δ 7.73 (d, J = 7.8 Hz, 1H), 7.53-7.51 (m, 1H), 7.40-7.38 (m, 1H), 7.35 (d, J = 7.2 Hz, 1H), 6.71 (s, 2H), 6.15 (brs, 1H), 3.63 (s, 3H), 2.93 (d, J = 13.2 Hz, 1H), 2.69 (d, J = 13.2 Hz, 1H), 2.17 (s, 6H); ^{13}C NMR (150 MHz, $CDCl_3$) δ 169.5, 156.0, 152.2, 131.8, 131.3, 131.1, 130.7, 130.6, 128.1, 123.9, 121.4, 61.9, 59.7, 46.1, 25.2, 16.0; FTIR (cm^{-1}): 3204, 2925, 1695, 1469, 1225, 1144, 736; mp = 163-165 °C (EtOAc:hexanes); HRMS (ESI) m/z, calculated for [$C_{19}H_{22}NO_2^+$]: 296.1645; found: 296.1650.

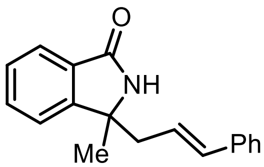

(**11**) According to the general protocol: $Pd(OAc)_2$ (11.3 mg, 0.05 mmol, 0.05 equiv), N-phenoxy-2-(prop-1-en-2-yl)benzamide **1** (253.3 mg, 1.00 mmol), (*E*)-styrylboronic acid (444 mg, 3.0 mmol, 3.0 equiv), 4Å MS (500 mg), $P(OCH_2CF_3)_3$ (49.2 mg, 0.15 mmol, 0.15 equiv), and anhydrous MeCN (10.0 mL) were combined under N_2. The reaction was stirred at 80 °C for 24 h under N_2 environment. The reaction was worked up according to the general procedure and purified by chromatography (linear gradient, 10:90 EtOAc:hexanes to 70:30 EtOAc:hexanes) to afford amide **11** (250.0 mg, 95%) as a colorless oil. ^{1}H NMR (600 MHz, $CDCl_3$) δ 7.75 (d, J = 7.2 Hz, 1H), 7.65-7.61 (m, 1H), 7.50-7.48 (m, 1H), 7.39-7.36 (m, 1H), 7.33 (d, J = 7.8 Hz, 1H), 7.18-7.16 (m, 4H), 7.12-7.10 (m, 1H), 6.31 (d, J = 15.7 Hz, 1H), 5.98 (dt, J = 15.4, 7.4 Hz, 1H), 2.65 (dd, J = 13.8, 7.8 Hz, 1H), 2.53 (dd, J = 13.8, 7.2 Hz, 1H), 1.49 (s, 3H); ^{13}C NMR (150 MHz, $CDCl_3$) δ 170.3, 151.8, 137.0, 134.6, 132.0, 131.4, 128.5, 128.1, 127.4, 126.3, 123.9, 123.9, 121.4, 62.0, 44.1, 25.6; FTIR (cm^{-1}): 3223, 2973, 1692, 1469, 1350, 749, 696; HRMS (ESI) m/z, calculated for [$C_{18}H_{18}NO^+$]: 264.1383; found: 264.1387.

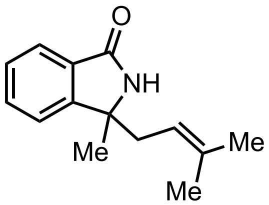

(**12**) According to the general protocol: $Pd(OAc)_2$ (11.3 mg, 0.05 mmol, 0.05 equiv), N-phenoxy-2-(prop-1-en-2-yl)benzamide **1** (253.3 mg, 1.00 mmol), (2-methylprop-1-en-1-yl)boronic acid (300 mg, 3.0 mmol, 3.0 equiv), 4Å MS (500 mg), $P(OCH_2CF_3)_3$ (49.2 mg, 0.15 mmol, 0.15 equiv), and anhydrous MeCN (10.0 mL) were combined under N_2. The reaction was stirred at 80 °C for 24 h under

N_2 environment. The reaction was worked up according to the general procedure and purified by chromatography (linear gradient, 10:90 EtOAc:hexanes to 80:20 EtOAc:hexanes) to afford amide **12** (146.1 mg, 65%) as a white solid. ^{1}H NMR (600 MHz, $CDCl_3$) δ 7.80 (d, *J* = 7.8 Hz, 1H), 7.54-7.51 (m, 1H), 7.43-7.41(m, 1H), 7.37 (d, *J* = 7.2 Hz, 1H), 7.28 (brs, 1H), 5.03-5.01 (m, 1H), 2.52 (dd, *J* = 14.4, 7.8 Hz, 1H), 2.41 (dd, *J* = 14.4, 7.8 Hz, 1H), 1.64 (s, 3H), 1.53 (s, 3H), 1.52 (s, 3H); ^{13}C NMR (150 MHz, $CDCl_3$) δ 169.8, 152.1, 136.0, 131.7, 131.2, 127.9, 123.7, 121.1, 118.0, 62.0, 38.8, 25.9, 25.4, 17.9; FTIR (cm^{-1}): 3208, 2971, 2927, 1695, 1452, 1319, 760, 699; mp = 78-80 °C (EtOAc:hexanes); HRMS (ESI) m/z, calculated for [$C_{14}H_{18}NO^+$]: 216.1383; found: 216.1387.

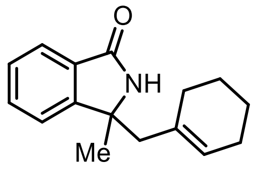

(**13**) According to the general protocol: $Pd(OAc)_2$ (11.3 mg, 0.05 mmol, 0.05 equiv), N-phenoxy-2-(prop-1-en-2-yl)benzamide **1** (253.3 mg, 1.00 mmol), cyclohex-1-en-1-ylboronic acid (378 mg, 3.0 mmol, 3.0 equiv), 4Å MS (500 mg), $P(OCH_2CF_3)_3$ (49.2 mg, 0.15 mmol, 0.15 equiv), and anhydrous MeCN (10.0 mL) were combined under N_2. The reaction was stirred at 80 °C for 24 h under N_2 environment. The reaction was worked up according to the general procedure and purified by chromatography (linear gradient, 10:90 EtOAc:hexanes to 80:20 EtOAc:hexanes) to afford amide **13** (220 mg, 91%) as a white solid. ^{1}H NMR (600 MHz, $CDCl_3$) δ 7.75-7.72 (m, 2H), 7.46-7.44 m, 1H), 7.35-7.33 (m, 1H), 7.30 (d, *J* = 7.2 Hz, 1H), 5.28 (s, 1H), 2.46 (d, *J* = 13.2 Hz, 1H), 2.32 (d, *J* = 13.8 Hz, 1H), 1.83-1.70 (m, 3H), 1.52-1,49 (m, 4H), 1.36-1.32 (m, 4H); ^{13}C NMR (150 MHz, $CDCl_3$) δ 170.1, 152.2, 132.7, 131.6, 131.5, 127.8, 127.3, 123.6, 121.5, 62.2, 48.5, 30.5, 26.4, 25.3, 22.9, 21.9; FTIR (cm^{-1}): 3206, 2926, 2835, 1695, 1469, 1372, 761, 700; mp = 112-114 °C (EtOAc:hexanes); HRMS (ESI) m/z, calculated for [$C_{16}H_{20}NO^+$]: 242.1539; found: 242.1542.

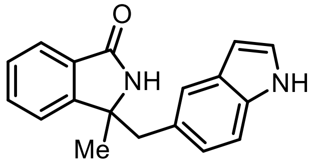

(**14**) According to the general protocol: $(COD)Pd(CH_2SiMe_3)_2$ (38.9 mg, 0.1 mmol, 0.1 equiv), N-phenoxy-2-(prop-1-en-2-yl)benzamide **1** (253.3 mg, 1.00 mmol), (1H-indol-5-yl)boronic acid acid (483 mg, 3.0 mmol, 3.0 equiv), 4Å MS (500 mg), $P(OCH_2CF_3)_3$ (98.4 mg, 0.3 mmol, 0.3 equiv), and anhydrous MeCN (10.0 mL) were combined under N_2. The reaction was stirred at 80 °C for 24 h under N_2 environment. The reaction was worked up according to the general procedure and purified by chromatography (linear gradient, 15:85 EtOAc:hexanes to 100:0 EtOAc:hexanes) to afford amide **14** (198.7 mg, 72%) as a white solid. ^{1}H NMR (600 MHz, $CDCl_3$) δ 8.32 (s, 1H), 7.80 (d, *J* = 7.2 Hz, 1H), 7.61-7.58 (m, 1H), 7.47 -7.43 (m, 3H), 7.31 (d, *J* = 7.8 Hz, 1H), 7.22-7.21 (m, 1H), 6.97 (dd, *J* = 8.4, 1.2 Hz, 1H), 6.50-6.49 (m, 1H), 6.17 (brs, 1H), 3.21 (d, *J* =13.8 Hz, 1H), 2.96 (d, *J* = 13.2 Hz, 1H), 1.49 (s, 3H); ^{13}C NMR (150 MHz, $CDCl_3$) δ 169.2, 152.6, 135.0, 131.9, 130.9, 128.1, 128.1, 127.2, 124.7, 124.5, 124.0, 122.2, 121.4, 110.9, 102.5, 62.0, 46.9, 25.0; FTIR (cm^{-1}): 3287, 1685, 1639, 1412, 1354, 1095, 755, 732; mp = > 200 °C Decompose (EtOAc:hexanes); HRMS (ESI) m/z, calculated for [$C_{18}H_{17}N_2O^+$]: 277.1335; found: 277.1339.

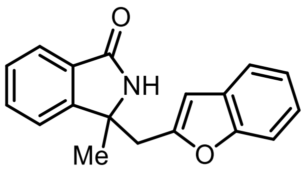

(**15**) According to the general protocol: (COD)Pd(CH_2SiMe_3)$_2$ (38.9 mg, 0.1 mmol, 0.1 equiv), N-phenoxy-2-(prop-1-en-2-yl)benzamide **1** (253.3 mg, 1.00 mmol), benzofuran-2-ylboronic acid (486 mg, 3.0 mmol, 3.0 equiv), 4Å MS (500 mg), P(OCH_2CF_3)$_3$ (98.4 mg, 0.3 mmol, 0.3 equiv), and anhydrous MeCN (10.0 mL) were combined under N_2. The reaction was stirred at 80 °C for 24 h under N_2 environment. The reaction was worked up according to the general procedure and purified by chromatography (linear gradient, 12:88 EtOAc:hexanes to 100:0 EtOAc:hexanes) to afford amide **15** (269.0 mg, 97%) as a white solid. ^{1}H NMR (600 MHz, $CDCl_3$) δ 7.73 (s, 1H), 7.70 (d, *J* = 7.8 Hz, 1H), 7.46-7.44 (m, 1H), 7.35-7.31 (m, 2H), 7.28 (d, *J* = 7.2 Hz, 1H), 7.24 (d, *J* = 8.4 Hz, 1H), 7.01-7.03 (m, 2H), 6.30 (s, 1H), 3.19 (d, *J* = 15.0 Hz, 1H), 3.02 (d, *J* = 14.4 Hz, 1H), 1.45 (s, 3H); ^{13}C NMR (150 MHz, $CDCl_3$) δ 169.8, 154.8, 153.9, 151.3, 132.1, 131.2, 128.4, 128.3, 124.0, 123.9, 122.8, 121.4, 120.7, 111.0, 105.7, 61.3, 39.7, 25.5; FTIR (cm^{-1}): 3218, 3070, 1697, 1615, 1469, 1315, 752, 739, 697; mp = 121-123 °C (EtOAc:hexanes); HRMS (ESI) m/z, calculated for [$C_{18}H_{16}NO_2^+$]: 278.1176; found: 278.1180.

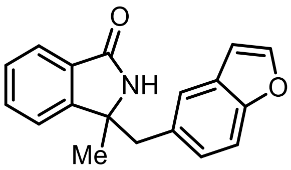

(**16**) According to the general protocol: (COD)Pd(CH_2SiMe_3)$_2$ (38.9 mg, 0.1 mmol, 0.1 equiv), N-phenoxy-2-(prop-1-en-2-yl)benzamide **1** (253.3 mg, 1.00 mmol), benzofuran-5-ylboronic acid (486 mg, 3.0 mmol, 3.0 equiv), 4Å MS (500 mg), P(OCH_2CF_3)$_3$ (98.4 mg, 0.3 mmol, 0.3 equiv), and anhydrous MeCN (10.0 mL) were combined under N_2. The reaction was stirred at 80 °C for 24 h under N_2 environment. The reaction was worked up according to the general procedure and purified by chromatography (linear gradient, 12:88 EtOAc:hexanes to 100:0 EtOAc:hexanes) to afford amide **16** (245.9 mg, 89%) as a white solid. ^{1}H NMR (600 MHz, $CDCl_3$) δ 7.66 (d, *J* = 7.8 Hz, 1H), 7.51-7.49 (m, 1H), 7.45 (d, *J* = 1.2 Hz, 1H), 7.36-7.34 (m, 3H), 7.21-7.19 (m, 2H), 6.86 (d, *J* = 8.4 Hz, 1H), 6.47 (d, *J* = 1.2 Hz, 1H), 3.13 (d, *J* = 13.2 Hz, 1H), 2.99 (d, *J* = 13.8 Hz, 1H), 1.46 (s, 3H); ^{13}C NMR (150 MHz, $CDCl_3$) δ 169.8, 154.1, 151.8, 145.2, 131.9, 131.4, 130.3, 128.1, 127.4, 126.6, 123.9, 122.8, 121.5, 110.9, 106.5, 62.3, 46.5, 25.5; FTIR (cm^{-1}): 3208, 1692, 1469, 1412, 1354, 1263, 1200, 759, 737; mp = 162-164 °C (EtOAc:hexanes); HRMS (ESI) m/z, calculated for [$C_{18}H_{16}NO_2^+$]: 278.1176; found: 278.1180.

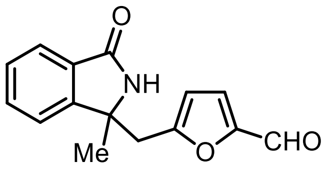

(**17**) According to the general protocol: (COD)Pd(CH_2SiMe_3)$_2$ (38.9 mg, 0.1 mmol, 0.1 equiv), N-phenoxy-2-(prop-1-en-2-yl)benzamide **1** (253.3 mg, 1.00 mmol), (5-formylfuran-2-yl)boronic acid (420 mg, 3.0 mmol, 3.0 equiv), 4Å MS (500 mg), P(OCH_2CF_3)$_3$ (98.4 mg, 0.3 mmol, 0.3 equiv), and anhydrous MeCN (10.0 mL) were combined under N_2. The reaction was stirred at 80 °C for 24 h under N_2 environment. The reaction was worked up according to the general procedure and purified by chromatography (linear gradient, 15:85 EtOAc:hexanes to 100:0 EtOAc:hexanes) to afford amide **17** (244.5 mg, 95%) as a white solid. ^{1}H NMR (600 MHz, $CDCl_3$) δ 9.35 (s, 1H), 8.15 (s, 1H), 7.68 (d, *J* = 7.2 Hz, 1H), 7.52-7.49 (m, 1H), 7.38-7.33 (m, 2H), 6.99 (d, *J* = 3.6 Hz, 1H), 6.04 (s, 1H), 3.29 (d, *J* = 15.0 Hz, 1H), 3.15 (d, *J* = 15.0 Hz, 1H), 1.56 (s, 3H); ^{13}C NMR (150 MHz, $CDCl_3$) δ 177.2,

169.9, 157.8, 152.2, 150.6, 132.2, 131.3, 128.5, 123.8, 121.3, 111.5, 61.3, 39.3, 25.8; FTIR (cm^{-1}): 3240, 2795, 1700, 1516, 1469, 1389, 1024, 736, 698; mp = 116-118 °C (EtOAc:hexanes); HRMS (ESI) m/z, calculated for [$C_{15}H_{14}NO_3^+$]: 256.0968; found: 256.0971.

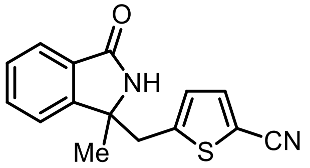

(**18**) According to the general protocol: (COD)Pd(CH_2SiMe_3)$_2$ (38.9 mg, 0.1 mmol, 0.1 equiv), N-phenoxy-2-(prop-1-en-2-yl)benzamide **1** (253.3 mg, 1.00 mmol), (5-cyanothiophen-2-yl)boronic acid (459 mg, 3.0 mmol, 3.0 equiv), 4Å MS (500 mg), P(OCH_2CF_3)$_3$ (98.4 mg, 0.3 mmol, 0.3 equiv), and anhydrous MeCN (10.0 mL) were combined under N_2. The reaction was stirred at 80 °C for 24 h under N_2 environment. The reaction was worked up according to the general procedure and purified by chromatography (linear gradient, 11:89 EtOAc:hexanes to 90:10 EtOAc:hexanes) to afford amide **18** (241.2 mg, 90%) as a white solid. ^{1}H NMR (600 MHz, $CDCl_3$) δ 8.38 (s, 1H), 7.72 (d, *J* = 7.8 Hz, 1H), 7.62-7.60 (m, 1H), 7.48-7.43 (m, 2H), 7.25 (d, *J* = 3.6 Hz, 1H), 6.62 (d, *J* = 3.8 Hz, 1H), 3.48 (d, *J* = 15.0 Hz, 1H), 3.38 (d, *J* = 15.0 Hz, 1H), 1.68 (s, 3H); ^{13}C NMR (150 MHz, $CDCl_3$) δ 170.4, 149.8, 145.5, 136.8, 132.3, 131.7, 128.8, 128.0, 124.0, 121.1, 114.1, 108.5, 61.8, 40.6, 26.3; FTIR (cm^{-1}): 3221, 2975, 2218, 1697, 1469, 1451, 699; mp = 188-190 °C (EtOAc:hexanes); HRMS (ESI) m/z, calculated for [$C_{15}H_{13}N_2OS^+$]: 269.0743; found: 269.0747.

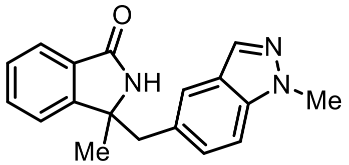

(**19**) According to the general protocol: (COD)Pd(CH_2SiMe_3)$_2$ (38.9 mg, 0.1 mmol, 0.1 equiv), N-phenoxy-2-(prop-1-en-2-yl)benzamide **1** (253.3 mg, 1.00 mmol), (1-methyl-1H-indazol-5-yl)boronic acid (528 mg, 3.0 mmol, 3.0 equiv), 4Å MS (500 mg), P(OCH_2CF_3)$_3$ (98.4 mg, 0.3 mmol, 0.3 equiv), and anhydrous MeCN (10.0 mL) were combined under N_2. The reaction was stirred at 80 °C for 24 h under N_2 environment. The reaction was worked up according to the general procedure and purified by chromatography (linear gradient, 12:88 EtOAc:hexanes to 100:0 EtOAc:hexanes) to afford amide **19** (149.4 mg, 52%) as a white solid. ^{1}H NMR (600 MHz, $CDCl_3$) δ 7.81 (s, 1H), 7.69 (d, *J* = 7.2 Hz, 1H), 7.54-7.52 (m, 1H), 7.39-7.36 (m, 3H), 7.21-7.19 (m, 3H), 7.05 (d, *J* = 8.4 Hz, 1H), 6.13 (brs, 1H), 3.98 (s, 3H), 3.15 (d, *J* = 13.8 Hz, 1H), 2.98 (d, *J* = 13.8 Hz, 1H), 1.46 (s, 3H); ^{13}C NMR (150 MHz, $CDCl_3$) δ 169.9, 151.7, 139.1, 132.4, 131.9, 131.5, 128.9, 128.1, 127.9, 124.0, 123.8, 122.3, 121.6, 108.4, 62.5, 46.4, 35.5, 25.6; FTIR (cm^{-1}): 3287, 1685, 1639, 1412, 1095, 755, 732; mp = > 200 °C Decompose (EtOAc:hexanes); HRMS (ESI) m/z, calculated for [$C_{18}H_{18}N_3O^+$]: 292.1444; found: 292.1448.

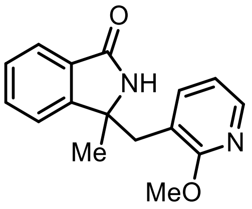

(**21**) According to the general protocol: (COD)Pd(CH_2SiMe_3)$_2$ (38.9 mg, 0.1 mmol, 0.1 equiv), N-phenoxy-2-(prop-1-en-2-yl)benzamide **1** (253.3 mg, 1.00 mmol), (2-methoxypyridin-3-yl)boronic acid acid (459 mg, 3.0 mmol, 3.0 equiv), 4Å MS (500 mg), P(OCH_2CF_3)$_3$ (98.4 mg, 0.3 mmol, 0.3 equiv), and anhydrous MeCN (10.0 mL) were combined under N_2. The reaction was stirred at 80 °C for 24 h under N_2 environment. The reaction was worked up according to the general

procedure and purified by chromatography (linear gradient, 12:88 EtOAc:hexanes to 100:0 EtOAc:hexanes) to afford amide **21** (207.8 mg, 78%) as a white solid. ^{1}H NMR (600 MHz, $CDCl_3$) δ 8.29 (s, 1H), 7.80 (d, *J* = 3.6 Hz, 1H), 7.57 (d, *J* = 7.2 Hz, 1H), 7.43-7.41 (m, 1H), 7.35 (d, *J* = 7.2 Hz, 1H), 7.27-7.25 (m, 1H), 7.00 (d, *J* = 7.2 Hz, 1H), 6.47-6.45 (m, 1H), 3.67 (s, 3H), 3.14 (d, *J* = 13.8 Hz, 1H), 3.01 (d, *J* = 13.8 Hz, 1H), 1.51 (s, 3H); ^{13}C NMR (150 MHz, $CDCl_3$) δ 170.1, 162.2, 150.9, 145.4, 139.8, 131.6, 131.3, 128.0, 123.3, 121.9, 118.5, 116.3, 62.7, 53.0, 39.0, 26.2; FTIR (cm^{-1}): 3214, 3074, 2975, 1696, 1615, 1586, 1467, 1413, 783, 735; mp = 115-118 °C (EtOAc:hexanes); HRMS (ESI) m/z, calculated for [$C_{16}H_{17}N_2O_2^+$]: 269.1285; found: 269.1289.

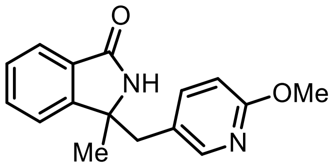

(**22**) According to the general protocol: (COD)Pd(CH_2SiMe_3)$_2$ (38.9 mg, 0.1 mmol, 0.1 equiv), N-phenoxy-2-(prop-1-en-2-yl)benzamide **1** (253.3 mg, 1.00 mmol), (6-methoxypyridin-3-yl)boronic acid (459 mg, 3.0 mmol, 3.0 equiv), 4Å MS (500 mg), P(OCH_2CF_3)$_3$ (98.4 mg, 0.3 mmol, 0.3 equiv), and anhydrous MeCN (10.0 mL) were combined under N_2. The reaction was stirred at 80 °C for 24 h under N_2 environment. The reaction was worked up according to the general procedure and purified by chromatography (linear gradient, 12:88 EtOAc:hexanes to 100:0 EtOAc:hexanes) to afford amide **22** (172.1 mg, 64%) as a white solid. ^{1}H NMR (600 MHz, $CDCl_3$) δ 7.79-7.64 (m, 3H), 7.53-7.50 (m, 1H), 7.36-7.35 (m, 2H), 7.11 (d, *J* = 8.4 Hz, 1H), 6.45 (d, *J* = 8.4 Hz, 1H), 3.75 (s, 3H), 3.01 (d, *J* = 13.8 Hz, 1H), 2.94 (d, *J* = 13.8 Hz, 1H), 1.54 (s, 3H); ^{13}C NMR (150 MHz, $CDCl_3$) δ 170.5, 163.0, 150.7, 147.8, 140.4, 131.9, 131.8, 128.2, 123.8, 123.7, 121.4, 109.8, 62.5, 53.2, 42.6, 26.0; FTIR (cm^{-1}): 3219, 2975, 1695, 1609, 1493, 1392, 1028, 762; mp = 165-167 °C (EtOAc:hexanes); HRMS (ESI) m/z, calculated for [$C_{16}H_{17}N_2O_2^+$]: 269.1285; found: 269.1289.

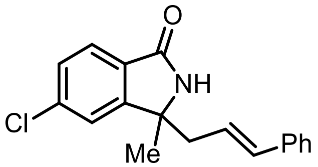

(**23**) According to the general protocol: $Pd(OAc)_2$ (11.3 mg, 0.05 mmol, 0.05 equiv), 4-chloro-N-phenoxy-2-(prop-1-en-2-yl)benzamide **S2** (287.8 mg, 1.00 mmol), (*E*)-styrylboronic acid (444 mg, 3.0 mmol, 3.0 equiv), 4Å MS (500 mg), P(OCH_2CF_3)$_3$ (49.2 mg, 0.15 mmol, 0.15 equiv), and anhydrous MeCN (10.0 mL) were combined under N_2. The reaction was stirred at 80 °C for 24 h under N_2 environment. The reaction was worked up according to the general procedure and purified by chromatography (linear gradient, 10:90 EtOAc:hexanes to 80:20 EtOAc:hexanes) to afford amide **23** (288.6 mg, 97%) as a white solid. ^{1}H NMR (600 MHz, $CDCl_3$) δ 7.96 (s, 1H), 7.73 (d, *J* = 7.8 Hz, 1H), 7.43 (dd, *J* = 8.4, 1.2 Hz, 1H), 7.40 (s, 1H), 7.25-7.24 (m, 4H), 7.21-7.18 (m, 1H), 6.39 (d, *J* = 16.2 Hz, 1H), 6.04-5.94 (m, 1H), 2.72 (dd, *J* = 13.8, 7.8 Hz, 1H), 2.58 (dd, *J* = 13.8, 7.2 Hz, 1H), 1.56 (s, 3H); ^{13}C NMR (150 MHz, $CDCl_3$) δ 169.0, 153.3, 138.3, 136.8, 135.0, 129.8, 128.7, 128.4, 127.5, 126.2, 125.1, 123.1, 121.8, 61.8, 43.8, 25.4; FTIR (cm^{-1}): 3202, 3060, 1696, 1611, 1450, 1421, 742, 693; mp = 177-179 °C (EtOAc:hexanes); HRMS (ESI) m/z, calculated for [$C_{18}H_{17}ClNO^+$]: 298.0993; found: 298.0998.

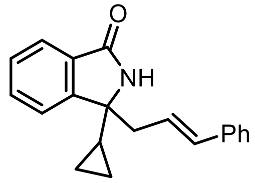

(**24**) According to the general protocol: $Pd(OAc)_2$ (11.3 mg, 0.05 mmol, 0.05 equiv), 2-(1-cyclopropylvinyl)-N-phenoxybenzamide **S4** (279.3 mg, 1.00 mmol), (*E*)-styrylboronic acid (444 mg, 3.0 mmol, 3.0 equiv), 4Å MS (500 mg), $P(OCH_2CF_3)_3$ (49.2 mg, 0.15 mmol, 0.15 equiv), and anhydrous MeCN (10.0 mL) were combined under N_2. The reaction was stirred at 80 °C for 24 h under N_2 environment. The reaction was worked up according to the general procedure and purified by chromatography (linear gradient, 5:95 EtOAc:hexanes to 70:30 EtOAc:hexanes) to afford amide **24** (158.9 mg, 55%) as a white solid. ^1H NMR (600 MHz, $CDCl_3$) δ 7.64 (d, *J* = 7.2 Hz, 1H), 7.59 (s, 1H), 7.42-7.40 (m, 1H), 7.31-7.28 (m, 2H), 7.11-7.02 (m, 5H), 6.22 (d, *J* = 15.6 Hz, 1H), 5.97-5.92 (m, 1H), 2.71 (dd, *J* = 13.8, 7.2 Hz, 1H), 2.58 (dd, *J* = 13.8, 7.2 Hz, 1H), 1.26-1.21 (m, 1H), 0.45-0.41 (m, 1H), 0.26-0.21 (m, 1H), 0.14-0.09 (m, 1H), 0.02--0.03 (m, 1H); ^{13}C NMR (150 MHz, $CDCl_3$) δ 168.6, 148.3, 134.7, 131.7, 129.5, 129.1, 126.0, 125.7, 124.8, 123.7, 121.5, 121.3, 119.2, 61.8, 40.8, 16.0, 0.0, -2.3; FTIR (cm^{-1}): 3199, 3078, 1693, 1468, 1344, 747, 693; mp = 151-153 °C (EtOAc:hexanes); HRMS (ESI) m/z, calculated for [$C_{20}H_{20}NO^+$]: 290.1539; found: 290.1543.

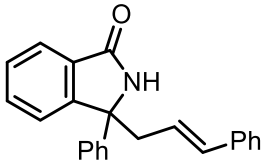

(**25**) According to the general protocol: $Pd(OAc)_2$ (11.3 mg, 0.05 mmol, 0.05 equiv), N-phenoxy-2-(1-phenylvinyl)benzamide **S5** (316 mg, 1.00 mmol), (*E*)-styrylboronic acid (444 mg, 3.0 mmol, 3.0 equiv), 4Å MS (500 mg), $P(OCH_2CF_3)_3$ (49.2 mg, 0.15 mmol, 0.15 equiv), and anhydrous MeCN (10.0 mL) were combined under N_2. The reaction was stirred at 80 °C for 24 h under N_2 environment. The reaction was worked up according to the general procedure and purified by chromatography (linear gradient, 10:90 EtOAc:hexanes to 80:20 EtOAc:hexanes) to afford amide **25** (289.7 mg, 90%) as a white solid. ^1H NMR (600 MHz, $CDCl_3$) δ 7.81 (d, *J* = 7.2 Hz, 1H), 7.54-7.51 (m, 3H), 7.44-7.42 (m, 2H), 7.37-7.35 (m, 3H), 7.30-7.28 (m, 1H), 7.23-7.17 (m, 5H), 6.44 (d, *J* = 15.6 Hz, 1H), 5.96-5.91 (m, 1H), 3.44 (dd, *J* = 13.8, 6.0 Hz, 1H), 2.94 (dd, *J* = 13.8, 7.8 Hz, 1H); ^{13}C NMR (150 MHz, $CDCl_3$) δ 170.4, 151.1, 140.9, 136.7, 135.0, 132.3, 130.3, 129.0, 128.4, 128.3, 127.8, 127.5, 126.2, 125.5, 124.0, 123.3, 122.3, 66.8, 43.0; FTIR (cm^{-1}): 3196, 3059, 1695, 1612, 1447, 966, 764, 695; mp = 190-192 °C (EtOAc:hexanes); HRMS (ESI) m/z, calculated for [$C_{23}H_{20}NO^+$]: 326.1539; found: 326.1544.

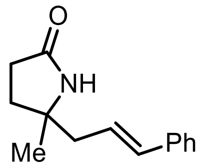

(**26**) According to the general protocol: $(COD)Pd(CH_2SiMe_3)_2$ (38.9 mg, 0.1 mmol, 0.1 equiv), 4-methyl-N-phenoxypent-4-enamide **S8** (205.2 mg, 1.00 mmol), (*E*)-styrylboronic acid (444 mg, 3.0 mmol, 3.0 equiv), 4Å MS (500 mg), $P(OCH_2CF_3)_3$ (98.4 mg, 0.3 mmol, 0.3 equiv), and anhydrous MeCN (10.0 mL) were combined under N_2. The reaction was stirred at 100 °C for 24 h in sealed tube. The reaction was worked up according to the general procedure and purified by chromatography (linear gradient, 16:84 EtOAc:hexanes to 100:0 EtOAc:hexanes) to afford amide **26** (197.5 mg, 92%) as a white solid. ^1H NMR (600 MHz, $CDCl_3$) δ 7.35 (d, *J* = 7.8 Hz, 2H), 7.30-7.28 (m, 2H), 7.22-7.20 m, 1H), 7.02 (s, 1H), 6.45 (d, *J* = 15.6 Hz, 1H), 6.20-6.15 (m, 1H), 2.43-2.36 (m, 4H), 2.06-2.01 (m, 1H), 1.87-1.82 (m, 1H), 1.30 (s, 3H); ^{13}C NMR (150 MHz, $CDCl_3$) δ 177.3, 137.0, 134.0, 128.4, 127.3, 126.1, 124.4, 59.3, 45.5, 32.9, 30.5, 27.4; FTIR (cm^{-1}): 3207, 2966,

1692, 969, 744, 694; mp = 74-77 °C (EtOAc:hexanes); HRMS (ESI) m/z, calculated for [$C_{16}H_{17}N_2O_2^+$]: 216.1383; found: 216.1388.

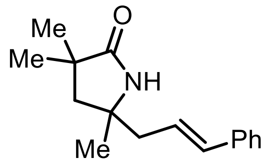

(**27**) According to the general protocol: (COD)Pd(CH_2SiMe_3)$_2$ (38.9 mg, 0.1 mmol, 0.1 equiv), 2,2,4-trimethyl-N-phenoxypent-4-enamide **S9** (233.3 mg, 1.00 mmol), (*E*)-styrylboronic acid (444 mg, 3.0 mmol, 3.0 equiv), 4Å MS (500 mg), P(OCH_2CF_3)$_3$ (98.4 mg, 0.3 mmol, 0.3 equiv), and anhydrous MeCN (10.0 mL) were combined under N_2. The reaction was stirred at 100 °C for 24 h in sealed tube. The reaction was worked up according to the general procedure and purified by chromatography (linear gradient, 12:88 EtOAc:hexanes to 100:0 EtOAc:hexanes) to afford amide **27** (222.5 mg, 91%) as a white solid. ^{1}H NMR (600 MHz, $CDCl_3$) δ 7.36 (d, *J* = 7.2 Hz, 2H), 7.32-7.30 (m, 2H), 7.24-7.22 (m, 1H), 6.46 (d, *J* = 15.6 Hz, 1H), 6.19-6.14 (m, 1H), 6.00 (s, 1H), 2.45-2.42 (m, 1H), 2.39-2.36 (m, 1H), 2.04 (d, *J* = 13.2 Hz, 1H), 1.85 (d, *J* = 13.2 Hz, 1H), 1.34 (s, 3H), 1.26 (s, 3H), 1.23 (s, 3H); ^{13}C NMR (150 MHz, $CDCl_3$) δ 181.9, 137.0, 134.0, 128.4, 127.3, 126.1, 124.8, 55.7, 48.0, 47.0, 40.8, 29.2, 27.7, 27.1; FTIR (cm^{-1}): 3196, 2965, 1687, 1468, 1449, 1362, 974, 745, 694; mp = 153-155 °C (EtOAc:hexanes); HRMS (ESI) m/z, calculated for [$C_{16}H_{22}NO^+$]: 244.1696; found: 244.1699.

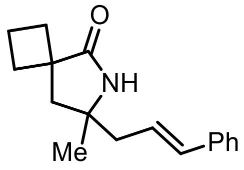

(**28**) According to the general protocol: (COD)Pd(CH_2SiMe_3)$_2$ (38.9 mg, 0.1 mmol, 0.1 equiv), 1-(2-methylallyl)-N-phenoxycyclobutanecarboxamide **S10** (245.3 mg, 1.00 mmol), (*E*)-styrylboronic acid (444 mg, 3.0 mmol, 3.0 equiv), 4Å MS (500 mg), P(OCH_2CF_3)$_3$ (98.4 mg, 0.3 mmol, 0.3 equiv), and anhydrous MeCN (10.0 mL) were combined under N_2. The reaction was stirred at 100 °C for 24 h in sealed tube. The reaction was worked up according to the general procedure and purified by chromatography (linear gradient, 12:88 EtOAc:hexanes to 100:0 EtOAc:hexanes) to afford amide **28** (218.2 mg, 86%) as a white solid. ^{1}H NMR (600 MHz, $CDCl_3$) δ 7.36 (d, *J* = 7.2 Hz, 2H), 7.31-7.28 (m, 2H), 7.22-7.20 (m, 1H), 6.65 (s, 1H), 6.43 (d, *J* = 15.6 Hz, 1H), 6.20-6.15 (m, 1H), 2.54-2.49 (m, 2H), 2.39-2.31 (m, 2H), 2.20 (d, *J* = 12.6 Hz, 1H), 2.11-2.06 (m, 1H), 2.03 (d, *J* = 13.2 Hz, 1H), 1.97-1.90 (mz, 3H), 1.26 (s, 3H); ^{13}C NMR (150 MHz, $CDCl_3$) δ 180.6, 137.0, 134.0, 128.5, 127.3, 126.1, 124.7, 56.9, 48.1, 46.1, 46.0, 32.1, 31.7, 27.9, 16.6; FTIR (cm^{-1}): 3192, 2927, 1696, 1369, 972, 746, 694; mp = 128-130 °C (EtOAc:hexanes); HRMS (ESI) m/z, calculated for [$C_{17}H_{22}NO^+$]: 256.1696; found: 256.1699.

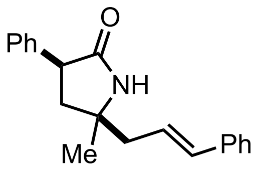

(**29**) According to the general protocol: (COD)Pd(CH_2SiMe_3)$_2$ (38.9 mg, 0.1 mmol, 0.1 equiv), 4-methyl-N-phenoxy-2-phenylpent-4-enamide **S11** (281.4 mg, 1.00 mmol), (*E*)-styrylboronic acid (444 mg, 3.0 mmol, 3.0 equiv), 4Å MS (500 mg), P(OCH_2CF_3)$_3$ (98.4 mg, 0.3 mmol, 0.3 equiv), and anhydrous MeCN (10.0 mL) were combined under N_2. The reaction was stirred at 100 °C for 24 h in sealed tube. The reaction was worked up according to the general procedure and purified by chromatography (linear gradient, 12:88 EtOAc:hexanes to 100:0 EtOAc:hexanes) to afford amide **29** (220 mg, 76%) as a white solid. ^{1}H NMR (600 MHz, $CDCl_3$) δ 7.52 (s, 1H), 7.22-7.08 (m, 10H), 6.31

(d, J = 15.6 Hz, 1H), 6.14-609 (m, 1H), 3.74 (t, J = 9.6 Hz, 1H), 2.38-2.30 (m, 2H), 2.25-2.21 (m, 1H), 2.05-2.01 m, 1H), 1.22 (s, 3H); ^{13}C NMR (150 MHz, $CDCl_3$) δ 177.0, 139.5, 136.9, 134.0, 128.5, 128.4, 128.1, 127.2, 126.8, 126.0, 124.4, 57.1, 47.6, 45.8, 43.2, 26.8; FTIR (cm^{-1}): 3195, 3028, 2966, 1693, 1497, 1449, 971, 748, 695; mp = 149-151 °C (EtOAc:hexanes); HRMS (ESI) m/z, calculated for [$C_{20}H_{22}NO^+$]: 292.1696; found: 292.1699.

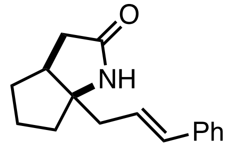

(**30**) According to the general protocol: (COD)Pd(CH_2SiMe_3)$_2$ (38.9 mg, 0.1 mmol, 0.1 equiv), 2-(2-methylenecyclopentyl)-N-phenoxyacetamide **S12** (231.3 mg, 1.00 mmol), (*E*)-styrylboronic acid (444 mg, 3.0 mmol, 3.0 equiv), 4Å MS (500 mg), P(OCH_2CF_3)$_3$ (98.4 mg, 0.3 mmol, 0.3 equiv), and anhydrous MeCN (10.0 mL) were combined under N_2. The reaction was stirred at 100 °C for 24 h in sealed tube. The reaction was worked up according to the general procedure and purified by chromatography (linear gradient, 12:88 EtOAc:hexanes to 100:0 EtOAc:hexanes) to afford amide **30** (197.7 mg, 82%) as a white solid. ^{1}H NMR (600 MHz, $CDCl_3$) δ 7.27-7.19 (m, 4H), 7.14-7.12 (m, 1H), 6.86 (d, J = 6.0 Hz, 1H), 6.39 (d, J = 15.6 Hz, 1H), 6.13-6.08 (m, 1H), 2.58-2.54 (m, 1H), 2.45 (dd, J = 13.8, 6.0 Hz, 1H), 2.39-2.34 (m, 2H), 1.97 (dd, J = 17.4, 2.4 Hz, 1H), 1.83-1.77 (m, 1H), 1.70-1.63 (m, 2H), 1.57-1.52 (m, 2H), 1.43-1.41 (m, 1H); ^{13}C NMR (150 MHz, $CDCl_3$) δ 177.7, 137.2, 134.0, 128.6, 127.4, 126.2, 124.8, 70.6, 44.4, 42.2, 39.2, 38.6, 34.9, 24.5; FTIR (cm^{-1}): 3197, 2951, 2866, 1689, 1422, 968, 748, 695; mp = 117-120 °C (EtOAc:hexanes); HRMS (ESI) m/z, calculated for [$C_{16}H_{20}NO^+$]: 242.1539; found: 242.1543.

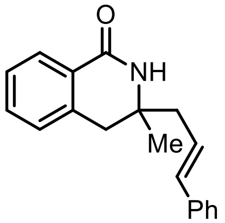

(**31**) According to the general protocol: Pd(OAc)$_2$ (11.3 mg, 0.05 mmol, 0.05 equiv), 2-(2-methylallyl)-N-phenoxybenzamide **S6** (267.4 mg, 1.00 mmol), (*E*)-styrylboronic acid (444 mg, 3.0 mmol, 3.0 equiv), 4Å MS (500 mg), P(OCH_2CF_3)$_3$ (49.2 mg, 0.15 mmol, 0.15 equiv), and anhydrous MeCN (10.0 mL) were combined under N_2. The reaction was stirred at 80 °C for 24 h under N_2 environment. The reaction was worked up according to the general procedure and purified by chromatography (linear gradient, 10:90 EtOAc:hexanes to 80:20 EtOAc:hexanes) to afford amide **31** (157.1 mg, 55%) as a white solid. ^{1}H NMR (600 MHz, $CDCl_3$) δ 8.00 (d, J = 7.8 Hz, 1H), 7.38 (m, 1H), 7.29-7.11 (m, 7H), 6.34 (d, J = 15.6 Hz, 1H), 6.14-6.09 (m, 2H), 2.96 (d, J = 15.6 Hz, 1H), 2.85 (d, J = 15.6 Hz, 1H), 2.40-2.39 (m, 2H), 1.27 (s, 3H); ^{13}C NMR (150 MHz, $CDCl_3$) δ 165.5, 137.3, 136.9, 134.7, 132.5, 128.6, 128.1, 128.0, 127.9, 127.6, 127.1, 126.2, 123.8, 54.7, 44.9, 40.0, 26.7; FTIR (cm^{-1}): 3193, 3060, 1664, 1578, 1462, 1392, 742; mp = 120-122 °C (EtOAc:hexanes); HRMS (ESI) m/z, calculated for [$C_{19}H_{20}NO^+$]: 278.1539; found: 278.1544.

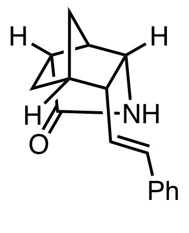

(**36**) According to the general protocol: (COD)Pd(CH_2SiMe_3)$_2$ (77.8 mg, 0.2 mmol, 0.2 equiv), N-phenoxybicyclo[2.2.1]hept-5-ene-2-carboxamide **35** (229.3 mg, 1.00 mmol), (*E*)-styrylboronic acid (444 mg, 3.0 mmol, 3.0 equiv), 4Å MS (500 mg), P(OCH_2CF_3)$_3$ (196.8 mg, 0.6 mmol, 0.6 equiv), and anhydrous MeCN (10.0 mL) were combined under N_2. The reaction was stirred at 120 °C for 24 h in sealed tube. The reaction was worked up

according to the general procedure and purified by chromatography (linear gradient, 18:82 EtOAc:hexanes to 100:0 EtOAc:hexanes) to afford amide **36** (118.4 mg, 49%) as a white solid. ^{1}H NMR (600 MHz, $CDCl_3$) δ 7.26 (d, *J* = 7.2 Hz, 2H), 7.20-7.18 (m, 2H), 7.12-7.10 (m, 1H), 6.39-6.36 (m, 2H), 6.17 (dd, *J* = 15.6, 8.4 Hz, 1H), 3.60 (t, *J* = 6.0 Hz, 1H), 3.05 (t, *J* = 4.2 Hz, 1H), 2.36-2.33 (m, 1H), 2.28 (s, 1H), 2.20 (dd, *J* = 10.8, 4.2 Hz, 1H), 1.89 (d, *J* = 13.2 Hz, 1H), 1.67-1.65 (m, 1H), 1.55 (d, *J* = 10.2 Hz, 1H), 1.47 (d, *J* = 10.8 Hz, 1H); ^{13}C NMR (150 MHz, $CDCl_3$) δ 182.9, 137.1, 132.8, 128.3, 127.2, 126.5, 126.1, 57.1, 48.4, 47.7, 42.9, 41.5, 37.1, 28.6; FTIR (cm^{-1}): 3218, 2956, 2875, 1698, 1449, 1257, 967, 747, 694; mp = 145-148 °C (EtOAc:hexanes); HRMS (ESI) m/z, calculated for [$C_{16}H_{18}NO^+$]: 240.1383; found: 240.1386.

A small sample of compound **36** was dissolved in EtOAc under air and recrystallized *via* slow vapor diffusion of hexanes at room temperature to give an X-ray quality crystal. See below for full crystallographic details.

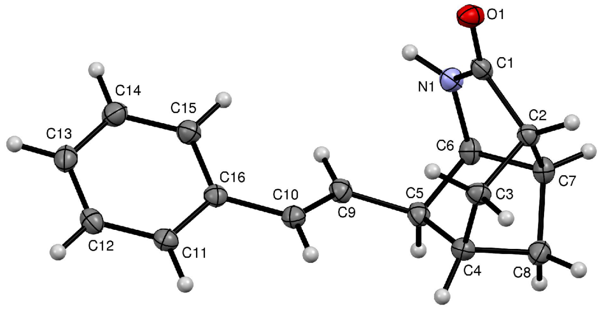

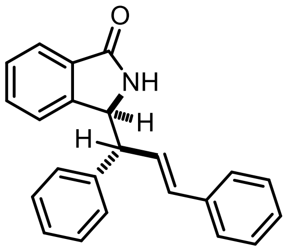

(**41**) According to the general protocol: $Pd(OAc)_2$ (11.3 mg, 0.05 mmol, 0.05 equiv), (*E*)-N-phenoxy-2-styrylbenzamide (315.4 mg, 1.00 mmol), (*E*)-styrylboronic acid (444 mg, 3.0 mmol, 3.0 equiv), 4Å MS (500 mg), $P(OCH_2CF_3)_3$ (49.2 mg, 0.15 mmol, 0.15 equiv), and anhydrous MeCN (10.0 mL) were combined under N_2. The reaction was stirred at 80 °C for 24 h under N_2 environment. The reaction was worked up according to the general procedure and purified by chromatography (linear gradient, 7:93 EtOAc:hexanes to 60:40 EtOAc:hexanes) to afford amide **41** (234.1 mg, 72%) as a colorless oil. ^{1}H NMR (600 MHz, $CDCl_3$) δ 7.73 (d, *J* = 7.2 Hz, 1H), 7.34-7.32 (m, 3H), 7.29-7.18 (m, 8H), 7.15-7.13 (m, 1H), 6.71 (brs, 1H), 6.46-6.24 (m, 2H), 6.35 (dd, *J* = 15.6, 8.4 Hz, 1H), 4.84 (d, *J* = 8.4 Hz, 1H), 3.47 (t, *J* = 8.4 Hz, 1H); ^{13}C NMR (150 MHz, $CDCl_3$) δ 170.3, 145.4, 140.2, 136.4, 133.3, 132.1, 131.4, 129.0, 128.5, 128.4, 128.3, 128.2, 127.8, 127.6, 126.4, 123.7, 123.6, 60.6, 54.6; FTIR (cm^{-1}): 3208, 1696, 1468, 1028, 744, 695; HRMS (ESI) m/z, calculated for [$C_{23}H_{20}NO^+$]: 326.1539; found: 326.1543.

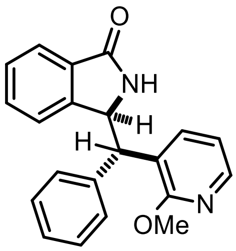

(**42**) According to the general protocol: $(COD)Pd(CH_2SiMe_3)_2$ (38.9 mg, 0.1 mmol, 0.1 equiv), (*E*)-N-phenoxy-2-styrylbenzamide (315.4 mg, 1.00 mmol), (2-methoxypyridin-3-yl)boronic acid acid (459 mg, 3.0 mmol, 3.0 equiv), 4Å MS (500 mg), $P(OCH_2CF_3)_3$ (98.4 mg, 0.3 mmol, 0.3 equiv), and anhydrous MeCN (10.0 mL) were combined under N_2. The reaction was stirred at 80 °C for 24 h under N_2 environment. The reaction was worked up according to the general procedure and

purified by chromatography (linear gradient, 12:88 EtOAc:hexanes to 100:0 EtOAc:hexanes) to afford amide **42** (179.8 mg, 54%) as a white solid. ^{1}H NMR (600 MHz, $CDCl_3$) δ 7.96 (d, *J* = 3.6 Hz, 1H), 7.70 (d, *J* = 7.2 Hz, 1H), 7.61 (d, *J* = 7.2 Hz, 1H), 7.32-7.17 (m, 7H), 6.80-6.78 (m, 1H), 6.60 (s, 1H), 6.31 (d, *J* = 7.8 Hz, 1H), 5.40 (d, *J* = 10.2 Hz, 1H), 4.17 (d, *J* = 10.2 Hz, 1H), 3.81 (s, 3H); ^{13}C NMR (150 MHz, $CDCl_3$) δ 170.4, 161.4, 146.0, 145.6, 139.9, 136.2, 132.0, 131.3, 128.84, 128.81, 128.4, 127.5, 124.0, 123.8, 123.7, 117.0, 58.7, 53.6, 50.1; FTIR (cm^{-1}): 3203, 3061, 1697, 1585, 1463, 1260, 748, 700; mp = 198-200 °C (EtOAc:hexanes); HRMS (ESI) m/z, calculated for [$C_{21}H_{19}N_2O_2^+$]: 331.1441; found: 331.1446.

A small sample of compound **42** was dissolved in EtOAc under air and recrystallized *via* slow vapor diffusion of hexanes at room temperature to give an X-ray quality crystal (See below for full crystallographic details).

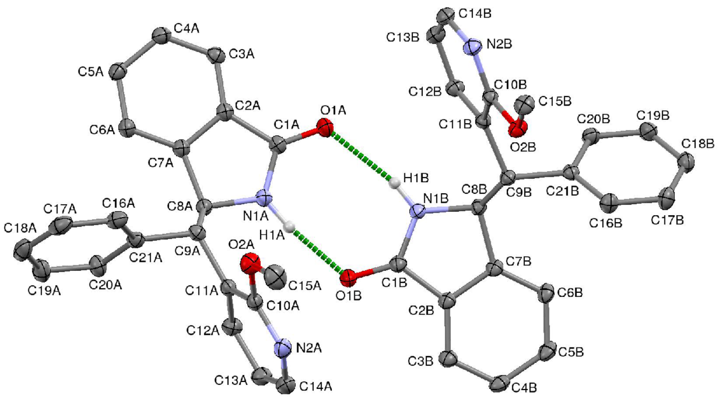

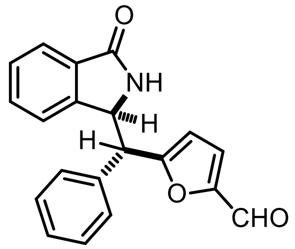

(**43**) According to the general protocol: (COD)Pd(CH_2SiMe_3)$_2$ (38.9 mg, 0.1 mmol, 0.1 equiv), (*E*)-N-phenoxy-2-styrylbenzamide (315.4 mg, 1.00 mmol), (5-formylfuran-2-yl)boronic acid (420 mg, 3.0 mmol, 3.0 equiv), 4Å MS (500 mg), P(OCH_2CF_3)$_3$ (98.4 mg, 0.3 mmol, 0.3 equiv), and anhydrous MeCN (10.0 mL) were combined under N_2. The reaction was stirred at 80 °C for 24 h under N_2 environment. The reaction was worked up according to the general procedure and purified by chromatography (linear gradient, 12:88 EtOAc:hexanes to 100:0 EtOAc:hexanes) to afford amide **43** (152.2 mg, 48%) as a white solid. ^{1}H NMR (600 MHz, $CDCl_3$) δ 9.51 (s, 1H), 7.71 (d, *J* = 7.8 Hz, 1H), 7.36-7.22 (m, 7H), 7.13-7.09 (m, 2H), 6.33 (d, *J* = 7.8 Hz, 1H), 6.30 (d, *J* = 3.6 Hz, 1H), 5.24 (d, *J* = 9.0 Hz, 1H), 4.10 (d, *J* = 9.0 Hz, 1H); ^{13}C NMR (150 MHz, $CDCl_3$) δ 177.5, 170.6, 160.6, 152.5, 144.7, 136.8, 132.2, 131.5, 129.1, 128.9, 128.7, 128.4, 123.8, 123.7, 110.7, 59.4, 51.1; FTIR (cm-1): 3205, 1698, 1678, 1513, 1469, 759, 702; mp = 182-184 °C (EtOAc:hexanes); HRMS (ESI) m/z, calculated for [$C_{20}H_{16}NO_3^+$]: 318.1125; found: 29318.1129.

A small sample of compound **43** was dissolved in EtOAc under air and recrystallized *via* slow vapor diffusion of hexanes at room temperature to give an X-ray quality crystal (See below for full crystallographic details).

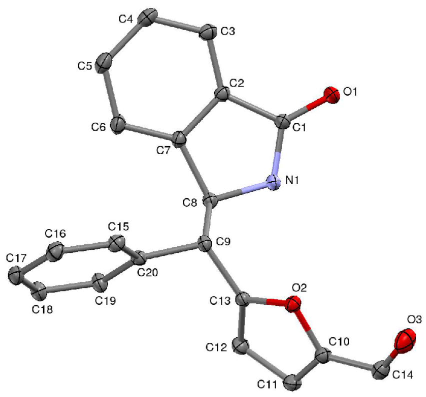

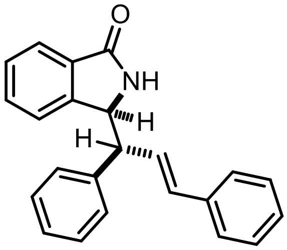

(**45**) According to the general protocol: $Pd(OAc)_2$ (11.3 mg, 0.05 mmol, 0.05 equiv), (*Z*)-N-phenoxy-2-styrylbenzamide (315.4 mg, 1.00 mmol), (*E*)-styrylboronic acid (444 mg, 3.0 mmol, 3.0 equiv), 4Å MS (500 mg), $P(OCH_2CF_3)_3$ (49.2 mg, 0.15 mmol, 0.15 equiv), and anhydrous MeCN (10.0 mL) were combined under N_2. The reaction was stirred at 80 °C for 24 h under N_2 environment. The reaction was worked up according to the general procedure and purified by chromatography (linear gradient, 7:93 EtOAc:hexanes to 60:40 EtOAc:hexanes) to afford amide **45** (162.6 mg, 50%) as a white solid. ^1H NMR (600 MHz, $CDCl_3$) δ 7.74 (d, *J* = 6.0 Hz, 1H), 7.39-7.18 (m, 13H), 6.48-6.41 (m, 2H), 6.36 (s, 1H), 4.89 (d, *J* = 8.4 Hz, 1H), 3.52 (t, *J* = 8.4 Hz, 1H); ^{13}C NMR (150 MHz, $CDCl_3$) δ 170.1, 145.6, 140.2, 136.6, 133.3, 132.2, 131.3, 129.1, 128.6, 128.4, 128.3, 127.9, 127.8, 127.4, 126.4, 124.1, 123.8, 60.3, 54.6; FTIR (cm^{-1}): 2923, 1669, 1494, 1340, 1261, 697; mp = 153-155 °C (EtOAc:hexanes); HRMS (ESI) m/z, calculated for $[C_{23}H_{20}NO^+]$: 326.1539; found: 326.1543.

5. Synthesis of Lactams *via* Aza-Heck-Carbonylation Reaction

5.1. Reaction Optimization of Aniline [a]

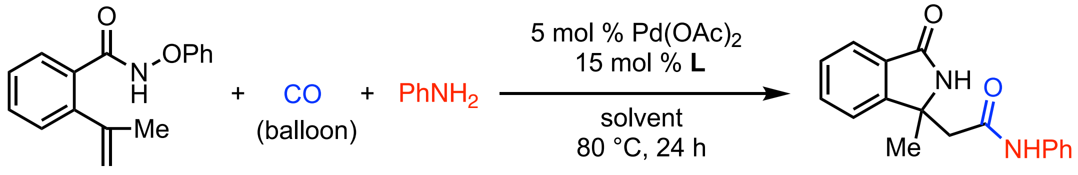

Entry	L	Solvent	Yield [%] [b]
1	$P(OCH_2CF_3)_3$	MeCN	40
2	$P(OCH_2CF_3)_3$	dioxane	2
3	$P(OCH_2CF_3)_3$	DCE	15
4	$P(OCH_2CF_3)_3$	*t*-BuOH	21
5	$P(OCH_2CF_3)_3$	PhMe	43
6	$P(OCH_2CF_3)_3$	Ph-F	27
7	$P(OCH_2CF_3)_3$	Ph-CF_3	58
8	P(O*i*-Pr)$_3$	Ph-CF_3	68

[a] Unless otherwise noted, reactions run with 0.2 mmol **1** and 0.3 mmol $PhNH_2$. [b] Yield calculated by ^{1}H NMR with 1,3,5-trimethoxybenzene as internal standard.

5.2. Synthesis of Lactams *via* Aza-Heck-Carbonylation Reaction

NOTE: CARBON MONOXIDE IS A HIGHLY TOXIC GAS. THESE PROCEDURES SHOULD ONLY BE CARRIED OUT BY A HIGHLY TRAINED EXPERIMENTALIST WITH EXPERTISE IN HANDLING TOXIC GASES. ADDITIONALLY, PROPER PERSONAL PROTECTIVE GEAR AND CARBON MONOXIDE MONITORS SHOULD BE EMPLOYED WHEN PREFORMING THESE PROCESSES. PLEASE CONSULT WITH YOUR LOCAL SAFETY OFFICALS BEFORE ATTEMPTING.

General Protocol: A flame-dried Schlenk flask equipped with a magnetic stir bar and rubber septum was attached to a double manifold and cooled under vacuum. The flask was backfilled with N_2, the septum was removed and $Pd(OAc)_2$ (0.05 equiv), hydroxamate (1.0 mmol, 1.0 equiv) were added to the flask. The septum was replaced, and the flask was evacuated and backfilled with nitrogen four times. Then anhydrous solvent (10.0 mL), $P(OCH_2CF_3)_3$ (0.15 equiv), and amine (1.5 mmol, 1.5 equiv) were added sequentially to the flask via syringe. The resulting solution was purged with CO which was introduced *via* a needle attached balloon for 30 s, then heated in an oil bath with rapid stirring at the indicated temperature for 24 h under CO which was introduced *via* a needle attached balloon. Upon completion, the reaction was cooled to room temperature, opened to air, and the reaction mixture was then diluted with EtOAc, filtered through celite. The suspension was adsorbed onto Celite via rotary evaporation. The resulting powder was

directly chromatographed on silica gel (5-20 μm particle size) to yield the desired product.

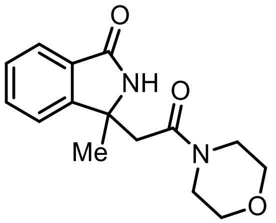

(**47**) According to the general protocol: $Pd(OAc)_2$ (11.3 mg, 0.05 mmol, 0.05 equiv), N-phenoxy-2-(prop-1-en-2-yl)benzamide **1** (253.3 mg, 1.00 mmol), morpholine (131 mg, 1.5 mmol, 1.5 equiv), $P(OCH_2CF_3)_3$ (49.2 mg, 0.15 mmol, 0.15 equiv), and anhydrous dioxane (10.0 mL) were combined under N_2. The resulting solution was purged with a CO balloon for 30 s, then heated in an oil bath with rapid stirring at 80 °C for 24 h under a CO balloon. The reaction was worked up according to the general procedure and purified by chromatography (linear gradient, 50:50 EtOAc:hexanes to 100:0 EtOAc:hexanes) to afford amide **47** (250.2 mg, 91%) as a white solid. ^1H NMR (600 MHz, $CDCl_3$) δ 7.74 (d, *J* = 7.2 Hz, 1H), 7.57 (s, 1H), 7.50-7.48 (m, 1H), 7.39-7.35 (m, 2H), 3.64-3.55 (m, 4H), 3.53-3.49 (m, 2H), 3.39-3.34 (m, 1H), 3.32-3.28 (m, 1H), 2.95 (d, *J* = 16.2 Hz, 1H), 2.32 (d, *J* = 16.2 Hz, 1H), 1.59 (s, 3H); ^{13}C NMR (150 MHz, $CDCl_3$) δ 168.5, 168.4, 151.9, 131.8, 130.7, 128.2, 123.9, 120.9, 66.5, 66.1, 59.3, 45.7, 41.9, 41.6, 24.8; FTIR (cm^{-1}): 3424, 3305, 2970, 2858, 1698, 1637, 1467, 1232, 1115, 1038, 734, 699; mp = 153-155 °C (EtOAc:hexanes); HRMS (ESI) m/z, calculated for [$C_{15}H_{19}N_2O_3^+$]: 275.1390; found: 275.1393.

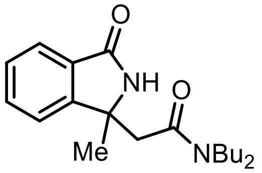

(**48**) According to the general protocol: $Pd(OAc)_2$ (11.3 mg, 0.05 mmol, 0.05 equiv), N-phenoxy-2-(prop-1-en-2-yl)benzamide **1** (253.3 mg, 1.00 mmol), dibutylamine (194 mg, 1.5 mmol, 1.5 equiv), $P(OCH_2CF_3)_3$ (49.2 mg, 0.15 mmol, 0.15 equiv), and anhydrous dioxane (10.0 mL) were combined under N_2. The resulting solution was purged with a CO balloon for 30 s, then heated in an oil bath with rapid stirring at 80 °C for 24 h under a CO balloon. The reaction was worked up according to the general procedure and purified by chromatography (linear gradient, 25:75 EtOAc:hexanes to 100:0 EtOAc:hexanes) to afford amide **48** (264.3 mg, 84%) as a white solid. ^1H NMR (600 MHz, $CDCl_3$) δ 7.79 (d, *J* = 7.8 Hz, 1H), 7.59 (s, 1H), 7.54-7.51 (m, 1H), 7.43-4.40 (m, 1H), 7.36 (d, *J* = 7.8 Hz, 1H), 3.33-3.25 (m, 2H), 3.14-3.11 (m, 2H), 2.96 (d, *J* = 16.2 Hz, 1H), 2.28 (d, *J* = 15.6 Hz, 1H), 1.59 (s, 3H), 1.51-1.44 (m, 4H), 1.31-1.23 (m, 4H), 0.91-0.87 (m, 6H); ^{13}C NMR (150 MHz, $CDCl_3$) δ 169.3, 168.4, 152.2, 131.8, 131.1, 128.2, 124.0, 120.9, 59.5, 47.6, 45.8, 42.1, 31.0, 29.7, 24.7, 20.2, 19.9, 13.7, 13.6. FTIR (cm^{-1}): 3425, 3290, 2959, 2932, 2873, 1703, 1631, 1468, 1375, 1216, 1140, 764, 697; mp = 82-84 °C (EtOAc:hexanes); HRMS (ESI) m/z, calculated for [$C_{19}H_{29}N_2O_2^+$]: 317.2224; found: 317.2227.

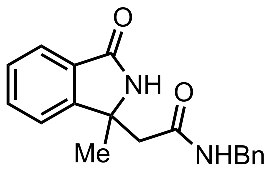

(**49**) According to the general protocol: $Pd(OAc)_2$ (11.3 mg, 0.05 mmol, 0.05 equiv), N-phenoxy-2-(prop-1-en-2-yl)benzamide **1** (253.3 mg, 1.00 mmol), phenylmethanamine (161 mg, 1.5 mmol, 1.5 equiv), $P(OCH_2CF_3)_3$ (49.2 mg, 0.15 mmol, 0.15 equiv), and anhydrous dioxane (10.0 mL) were combined under N_2. The resulting solution was purged with a CO balloon for 30 s, then heated in an oil bath with rapid stirring at 80 °C for 24 h under a CO balloon. The reaction was worked up according to the general procedure and purified by chromatography (linear gradient, 7:93 EtOAc:hexanes to 40:60 EtOAc:hexanes) to afford amide **49** (258.2 mg, 88%) as a

colorless oil. ^{1}H NMR (600 MHz, $CDCl_3$) δ 7.64 (d, *J* = 7.2 Hz, 1H), 7.58 (s, 1H), 7.44 (td, *J* = 7.8, 1.2 Hz, 1H), 7.32 (td, *J* = 7.2, 0.6 Hz, 1H), 7.27 (d, *J* = 7.2 Hz, 1H), 7.21-7.19 (m, 2H), 7.17-7.14 (m, 1H), 7.11 (d, *J* = 7.2 Hz, 2H), 6.53 (t, *J* = 4.8 Hz, 1H), 4.34 (dd, *J* = 15.0, 6.0 Hz, 1H), 4.27 (dd, *J* = 14.0, 5.4 Hz, 1H), 2.73 (d, *J* = 15.0 Hz, 1H), 2.36 (d, *J* = 14.4 Hz, 1H), 1.50 (s, 3H); ^{13}C NMR (150 MHz, $CDCl_3$) δ 169.6, 169.1, 151.4, 138.0, 132.0, 130.7, 128.4, 128.2, 127.5, 127.2, 123.7, 121.2, 59.9, 45.5, 43.3, 25.1; FTIR (cm^{-1}): 3289, 3064, 2975, 1692, 1615, 1551, 1469, 1349, 735, 698; HRMS (ESI) m/z, calculated for [$C_{18}H_{19}N_2O_2^+$]: 295.1441; found: 295.1445.

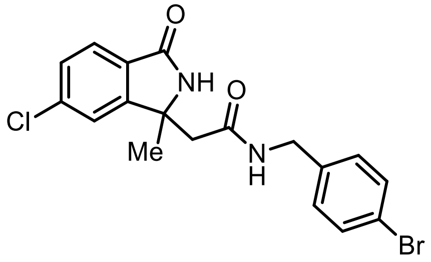

(**50**) According to the general protocol: $Pd(OAc)_2$ (11.3 mg, 0.05 mmol, 0.05 equiv), 4-chloro-N-phenoxy-2-(prop-1-en-2-yl)benzamide **S2** (287.8 mg, 1.00 mmol), (4-bromophenyl)methanamine (279.2 mg, 1.5 mmol, 1.5 equiv), $P(OCH_2CF_3)_3$ (49.2 mg, 0.15 mmol, 0.15 equiv), and anhydrous dioxane (10.0 mL) were combined under N_2. The resulting solution was purged with a CO balloon for 30 s, then heated in an oil bath with rapid stirring at 80 ^{o}C for 24 h under a CO balloon. The reaction was worked up according to the general procedure and purified by chromatography (linear gradient, 12:88 EtOAc:hexanes to 90:10 EtOAc:hexanes) to afford amide **50** (184.1mg, 45%) as a white solid. ^{1}H NMR (600 MHz, CD_3OD) δ 7.67 (d, *J* = 7.8 Hz, 1H), 7.60 (d, *J* = 1.2 Hz, 1H), 7.49 (dd, *J* = 7.8, 1.8 Hz, 1H), 7.43-7.41 (m, 2H), 7.04 (d, *J* = 8.4 Hz, 2H), 4.24 (d, *J* = 15.0 Hz, 1H), 4.20 (d, *J* = 15.0 Hz, 1H), 2.81 (d, *J* = 14.4 Hz, 1H), 2.70 (d, *J* = 14.4 Hz, 1H), 1.59 (s, 3H); ^{13}C NMR (150 MHz, CD_3OD) δ 171.0, 170.3, 154.4, 139.6, 139.1, 132.6, 131.0, 130.5, 130.1, 126.0, 123.8, 121.9, 61.6, 45.8, 43.3, 26.3; FTIR (cm^{-1}): 3262, 1691, 1650, 1553, 1406, 834, 784; mp = > 200 °C Decompose (EtOAc:hexanes); HRMS (ESI) m/z, calculated for [$C_{18}H_{17}ClBrN_2O_2^+$]: 407.0156; found: 407.0165.

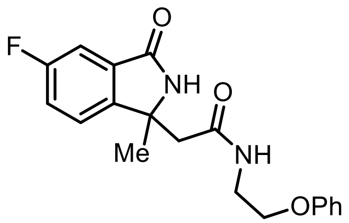

(**51**) According to the general protocol: $Pd(OAc)_2$ (11.3 mg, 0.05 mmol, 0.05 equiv), 5-fluoro-N-phenoxy-2-(prop-1-en-2-yl)benzamide **S1** (271.3 mg, 1.00 mmol), 2-phenoxyethanamine (205.8 mg, 1.5 mmol, 1.5 equiv), $P(OCH_2CF_3)_3$ (49.2 mg, 0.15 mmol, 0.15 equiv), and anhydrous dioxane (10.0 mL) were combined under N_2. The resulting solution was purged with a CO balloon for 30 s, then heated in an oil bath with rapid stirring at 80 ^{o}C for 24 h under a CO balloon. The reaction was worked up according to the general procedure and purified by chromatography (linear gradient, 8:92 EtOAc:hexanes to 70:30 EtOAc:hexanes) to afford amide **51** (210.1mg, 61%) as a white solid. ^{1}H NMR (600 MHz, $CDCl_3$) δ 7.81 (s, 1H), 7.41 (dd, *J* = 7.8, 2.4 Hz, 1H), 7.34 (dd, *J* = 8.4, 4.2 Hz, 1H), 7.27-7.24 (m, 2H), 7.20 (td, *J* = 9.0, 2.4 Hz, 1H), 6.96-6.93 (m, 1H), 6.84 (d, *J* = 7.8 Hz, 2H), 6.51-6.49 (m, 1H), 4.01-3.95 (m, 2H), 3.68-3.60 (m, 2H), 2.79 (d, *J* = 14.4 Hz, 1H), 2.44 (d, *J* = 14.4 Hz, 1H), 1.57 (s, 3H); ^{13}C NMR (150 MHz, $CDCl_3$) δ 169.5, 167.8 (d, *J* = 3.3 Hz), 162.9 (d, *J* = 246.5 Hz), 158.3, 146. 9 (d, *J* = 2.2 Hz), 133.2 (d, *J* = 8.4 Hz), 129.5, 122.7 (d, *J* = 8.4 Hz), 121.3, 119.5 (d, *J* = 23.6 Hz), 114.4, 110.7(d, *J* = 23.3 Hz), 66.4, 59.6, 45.8, 39.0, 25.2; ^{19}F NMR (565 MHz, $CDCl_3$) δ -112.6 (m); FTIR (cm^{-1}): 3300, 3069, 2935, 1698, 1657, 1487, 1243, 1223,

755, 692; mp = 134-136 °C (EtOAc:hexanes); HRMS (ESI) m/z, calculated for [$C_{19}H_{20}FN_2O_3^+$]: 343.1452; found: 343.1455.

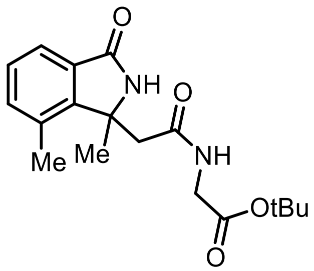

(**52**) According to the general protocol: $Pd(OAc)_2$ (11.3 mg, 0.05 mmol, 0.05 equiv), 3-methyl-N-phenoxy-2-(prop-1-en-2-yl)benzamide **S3** (267.3 mg, 1.00 mmol), tert-butyl-2-aminoacetate (196.8 mg, 1.5 mmol, 1.5 equiv), $P(OCH_2CF_3)_3$ (49.2 mg, 0.15 mmol, 0.15 equiv), and anhydrous dioxane (10.0 mL) were combined under N_2. The resulting solution was purged with a CO balloon for 30 s, then heated in an oil bath with rapid stirring at 80 °C for 24 h under a CO balloon. The reaction was worked up according to the general procedure and purified by chromatography (linear gradient, 8:92 EtOAc:hexanes to 70:30 EtOAc:hexanes) to afford amide **52** (310.1mg, 93%) as a white solid. ^{1}H NMR (600 MHz, $CDCl_3$) δ 7.92 (s, 1H), 7.50-7.48 (m, 1H), 7.33-7.30 (m, 1H), 7.25-7.24 (m, 2H), 3.97 (dd, *J* = 18.0, 6.0 Hz, 1H), 3.73 (dd, *J* = 18.0, 5.4 Hz, 1H), 3.08 (d, *J* = 13.8 Hz, 1H), 2.45 (d, *J* = 13.8 Hz, 1H), 2.43 (s, 3H), 1.63 (s, 3H), 1.38 (s, 9H); ^{13}C NMR (150 MHz, $CDCl_3$) δ 170.0, 169.5, 169.0, 148.6, 134.4, 132.2, 131.4, 128.4, 121.5, 82.2, 60.7, 43.7, 42.1, 27.9, 23.0, 18.6; FTIR (cm^{-1}): 3299, 2980, 2935, 1743, 1695, 1553, 1368, 1226, 1156, 735; mp = 143-145 °C (EtOAc:hexanes); HRMS (ESI) m/z, calculated for [$C_{18}H_{25}FNO_4^+$]: 333.1809; found: 333.1811.

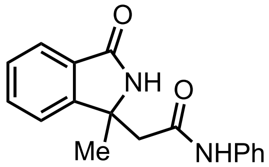

(**53**) According to the general protocol: $Pd(OAc)_2$ (11.3 mg, 0.05 mmol, 0.05 equiv), N-phenoxy-2-(prop-1-en-2-yl)benzamide **1** (253.3 mg, 1.00 mmol), aniline (140 mg, 1.5 mmol, 1.5 equiv), P(O*i*-Pr)$_3$ (31.3 mg, 0.15 mmol, 0.15 equiv), and anhydrous Ph-CF_3 (10.0 mL) were combined under N_2. The resulting solution was purged with a CO balloon for 30 s, then heated in an oil bath with rapid stirring at 80 °C for 24 h under a CO balloon. The reaction was worked up according to the general procedure and purified by chromatography (linear gradient, 20:80 EtOAc:hexanes to 100:0 EtOAc:hexanes) to afford amide **53** (207.2 mg, 77%) as a white solid. ^{1}H NMR (600 MHz, $CDCl_3$) δ 8.30 (s, 1H), 7.75 (s, 1H), 7.66 (d, *J* = 7.2 Hz, 1H), 7.47-7.45 (m, 1H), 7.39 (d, *J* = 7.8 Hz, 2H), 7.34-7.30 (m, 2H), 7.18-7.15 (m, 2H), 7.00-6.98 (m, 1H), 2.89 (d, *J* = 14.4 Hz, 1H), 2.50 (d, *J* = 15.0 Hz, 1H), 1.55 (s, 3H); ^{13}C NMR (150 MHz, $CDCl_3$) δ 169.4, 168.1, 151.5, 137.6, 132.3, 130.7, 128.9, 128.5, 124.5, 124.0, 121.3, 120.2, 60.1, 46.6, 25.2; FTIR (cm^{-1}): 3293, 3057, 1669, 1600, 1548, 1443, 1308, 755, 696; mp = 159-161 °C (EtOAc:hexanes); HRMS (ESI) m/z, calculated for [$C_{17}H_{17}N_2O_2^+$]: 281.1285; found: 281.1289.

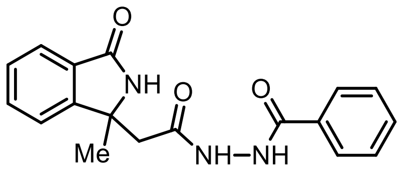

(**54**) According to the general protocol: $Pd(OAc)_2$ (11.3 mg, 0.05 mmol, 0.05 equiv), N-phenoxy-2-(prop-1-en-2-yl)benzamide **1** (253.3 mg, 1.00 mmol), benzohydrazide (204.3 mg, 1.5 mmol, 1.5 equiv), $P(OCH_2CF_3)_3$ (49.2 mg, 0.15 mmol, 0.15 equiv), and anhydrous dioxane (10.0 mL) were combined under N_2. The resulting solution was purged with a CO balloon for 30 s, then heated in an oil bath with rapid stirring at 80 °C for 24 h under a CO balloon. The reaction was worked up according to the general

procedure and purified by chromatography (linear gradient, 10:90 EtOAc:hexanes to 90:10 EtOAc:hexanes) to afford amide **54** (202.6 mg, 63%) as a white solid. ^{1}H NMR (600 MHz, CD_3OD) δ 7.89-7.88 (m, 2H), 7.76 (d, *J* = 7.8 Hz, 1H), 7.67-7.64 (m, 2H), 7.59-7.56 (m, 1H), 7.53-7.47 (m, 3H), 2.93 (d, *J* = 14.4 Hz, 1H), 2.60 (d, *J* = 14.4 Hz, 1H), 1.68 (s, 3H); ^{13}C NMR (150 MHz, CD_3OD) δ 171.4, 171.2, 169.3, 153.2, 133.7, 133.4, 131.8, 129.7, 128.7, 124.6, 123.1, 61.5, 44.5, 25.6; FTIR (cm^{-1}): 3240, 1690, 1605, 1310, 695; mp = > 200 °C Decompose (EtOAc:hexanes); HRMS (ESI) m/z, calculated for [$C_{18}H_{18}N_3O_3^+$]: 324.1343; found: 324.1346.

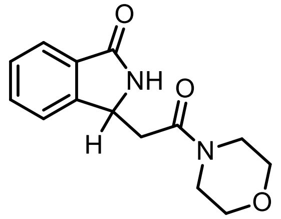

(**55**) According to the general protocol: $Pd(OAc)_2$ (11.3 mg, 0.05 mmol, 0.05 equiv), N-phenoxy-2-vinylbenzamide **S7** (240 mg, 1.00 mmol), morpholine (131 mg, 1.5 mmol, 1.5 equiv), $P(OCH_2CF_3)_3$ (49.2 mg, 0.15 mmol, 0.15 equiv), and anhydrous dioxane (10.0 mL) were combined under N_2. The resulting solution was purged with CO for 30 s, then heated in an oil bath with rapid stirring at 80 °C for 24 h under 20 psi CO. The reaction was worked up according to the general procedure and purified by chromatography (linear gradient, 80:20 EtOAc:hexanes to 100:0 EtOAc:hexanes) to afford amide **55** (160.8 mg, 62%) as a colorless oil. ^{1}H NMR (600 MHz, $CDCl_3$) δ 7.83 (d, *J* = 7.8 Hz, 1H), 7.55-7.53 (m, 1H), 7.47-7.44 (m, 1H), 7.41 (d, *J* = 7.2 Hz, 1H), 7.11 (s, 1H), 5.01 (dd, *J* = 10.8, 3.0 Hz, 1H), 3.71-3.59 (m, 6H), 3.42-3.35 (m, 2H), 3.00 (dd, *J* = 16.2, 3.0 Hz, 1H), 2.38 (dd, *J* = 16.2, 10.2 Hz, 1H); ^{13}C NMR (150 MHz, $CDCl_3$) δ 169.7, 168.7, 146.3, 132.2, 131.7, 128.4, 124.0, 122.2, 66.7, 66.3, 53.1, 45.7, 42.0, 38.8. FTIR (cm^{-1}): 3428, 3290, 2859, 1702, 1640, 1467, 1273, 1115, 1031, 733, 697; HRMS (ESI) m/z, calculated for [$C_{14}H_{17}N_2O_3^+$]: 261.1234; found: 261.1237.

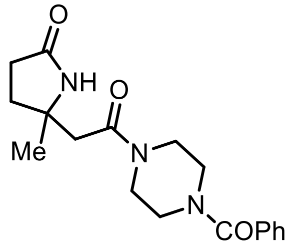

(**56**) According to the general protocol: $Pd(OAc)_2$ (11.3 mg, 0.05 mmol, 0.05 equiv), 4-methyl-N-phenoxypent-4-enamide **S8** (205.3 mg, 1.00 mmol), phenyl(piperazin-1-yl)methanone (286 mg, 1.5 mmol, 1.5 equiv), $P(OCH_2CF_3)_3$ (49.2 mg, 0.15 mmol, 0.15 equiv), and anhydrous dioxane (10.0 mL) were combined under N_2. The resulting solution was purged with a CO balloon for 30 s, then heated in an oil bath with rapid stirring at 100 °C for 24 h under a CO balloon. The reaction was worked up according to the general procedure and purified by chromatography (linear gradient, 2:98 acetone:hexanes to 16:84 acetone:hexanes) to afford amide **56** (212.1mg, 64%) as a white solid. ^{1}H NMR (600 MHz, $CDCl_3$) δ 7.42-7.35 (m, 5H), 6.87 (s, 1H), 3.70-3.31 (m, 8H), 2.59 (brs, 1H), 2.45 (d, *J* = 15.6 Hz, 1H), 2.39-2.26 (m, 2H), 2.00-1.93 (m, 2H), 1.36 (s, 3H); ^{13}C NMR (150 MHz, $CDCl_3$) δ 176.2, 170.5, 168.9, 134.9, 130.0, 128.5, 126.9, 57.4, 43.6, 35.0, 29.4, 26.9; FTIR (cm^{-1}): 3413, 3269, 2969, 1694, 1633, 1429, 1008, 732, 710; mp = > 200 °C Decompose (acetone:hexanes); HRMS (ESI) m/z, calculated for [$C_{18}H_{24}N_3O_3^+$]: 330.1812; found: 330.1815.

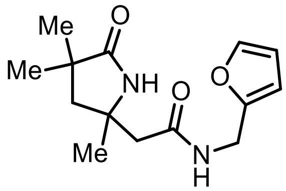

(**57**) According to the general protocol: $Pd(OAc)_2$ (11.3 mg, 0.05 mmol, 0.05 equiv), 2,2,4-trimethyl-N-phenoxypent-4-enamide **S9** (233.4 mg, 1.00 mmol), furan-2-ylmethanamine (145.8 mg, 1.5 mmol, 1.5 equiv), $P(OCH_2CF_3)_3$ (49.2 mg, 0.15 mmol, 0.15 equiv), and anhydrous dioxane (10.0 mL) were combined under N_2. The resulting solution was purged with a CO balloon for 30 s, then heated in an oil bath with rapid stirring at 100 °C for 24 h under a CO balloon. The reaction was worked up according to the general procedure and purified by chromatography (linear gradient, 80:20 EtOAc:hexanes to 100:0 EtOAc:hexanes) to afford amide **57** (160.6, 61%) as a white solid. ^1H NMR (600 MHz, $CDCl_3$) δ 7.32 (d, *J* = 1.2 Hz, 1H), 6.90 (s, 1H), 6.60 (s, 1H), 6.28 (dd, *J* = 3.0, 1.8 Hz, 1H), 6.19 (d, *J* = 3.0 Hz, 1H), 4.42-4.35 (m, 2H), 2.40 (s, 2H), 2.02 (d, *J* = 13.2 Hz, 1H), 1.88 (d, *J* = 13.2 Hz, 1H), 1.34 (s, 3H), 1.20 (s, 3H), 1.14 (s, 3H); ^{13}C NMR (150 MHz, $CDCl_3$) δ 181.2, 169.9, 151.1, 142.1, 110.4, 107.4, 54.2, 49.2, 48.9, 40.2, 36.3, 28.9, 27.5, 27.1; FTIR (cm^{-1}): 3279, 2967, 2870, 1656, 1549, 1410, 1254, 733; mp = 175-178 °C (EtOAc:hexanes); HRMS (ESI) m/z, calculated for [$C_{14}H_{21}N_2O_3^+$]: 265.1547; found: 265.1550.

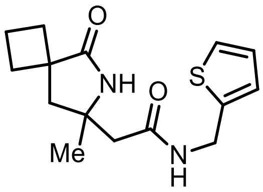

(**58**) According to the general protocol: $Pd(OAc)_2$ (11.3 mg, 0.05 mmol, 0.05 equiv), 1-(2-methylallyl)-N-phenoxycyclobutanecarboxamide **S10** (245.3mg, 1.00 mmol), thiophen-2-ylmethanamine (169.8 mg, 1.5 mmol, 1.5 equiv), $P(OCH_2CF_3)_3$ (49.2 mg, 0.15 mmol, 0.15 equiv), and anhydrous dioxane (10.0 mL) were combined under N_2. The resulting solution was purged with a CO balloon for 30 s, then heated in an oil bath with rapid stirring at 100 °C for 24 h under a CO balloon. The reaction was worked up according to the general procedure and purified by chromatography (linear gradient, 80:20 EtOAc:hexanes to 100:0 EtOAc:hexanes) to afford amide **58** (195.4, 67%) as a white solid. ^1H NMR (600 MHz, $CDCl_3$) δ 7.16 (dd, *J* = 4.8, 1.2 Hz, 1H), 6.93-6-89 (m, 3H), 6.84 (s, 1H), 4.57 (dd, *J* = 15.6, 6.0 Hz, 1H), 4.51 (dd, *J* = 15.6, 6.0 Hz, 1H), 2.44-2.39 (m, 3H), 2.31 (d, *J* = 14.4 Hz, 1H), 2.15 (d, *J* = 12.6 Hz, 1H), 2.10 (d, *J* = 12.6 Hz, 1H), 2.03-1.98 (m, 1H), 1.96-1.83 (m, 3H), 1.25 (s, 3H); ^{13}C NMR (150 MHz, $CDCl_3$) δ 180.0, 169.8, 141.0, 126.8, 125.8, 125.0, 55.4, 48.8, 47.9, 45.3, 38.1, 32.5, 30.8, 27.6, 16.6. FTIR (cm^{-1}): 3281, 3072, 2930, 1650, 1549, 1264, 1161, 700; mp = 101-103 °C (EtOAc:hexanes); HRMS (ESI) m/z, calculated for [$C_{15}H_{21}SN_2O^+$]: 293.1318; found: 293.1321.

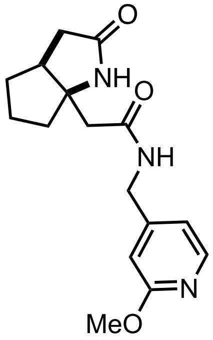

(**59**) According to the general protocol: $Pd(OAc)_2$ (11.3 mg, 0.05 mmol, 0.05 equiv), 2-(2-methylenecyclopentyl)-N-phenoxyacetamide **S12** (231.3 mg, 1.00 mmol), (2-methoxypyridin-4-yl)methanamine (207.3 mg, 1.5 mmol, 1.5 equiv), $P(OCH_2CF_3)_3$ (49.2 mg, 0.15 mmol, 0.15 equiv), and anhydrous dioxane (10.0 mL) were combined under N_2. The resulting solution was purged with a CO balloon for 30 s, then heated in an oil bath with rapid stirring at 100 °C for 24 h under a CO balloon. The reaction was worked up according to the general procedure and purified by chromatography (linear gradient, 25:75 EtOAc:hexanes to 100:0 EtOAc:hexanes) to afford amide **59** (212.1 mg, 66%) as a colorless oil. ^1H NMR (600 MHz, $CDCl_3$) δ 8.01 (d, *J* = 4.8 Hz, 1H), 7.31-7.29 (m, 1H), 6.91 (s, 1H),

6.68 (d, J = 4.8 Hz, 1H), 6.53 (s, 1H), 4.30-4.22 (m, 2H), 3.84 (s, 3H), 2.57-2.49 (m, 4H), 1.89 (d, J = 15.0 Hz, 1H), 1.81-1.78 (m, 2H), 1.60-1.57 (m, 2H), 1.52-1.47 (m, 1H), 1.42-1.40 (m, 1H); ^{13}C NMR (150 MHz, $CDCl_3$) δ 177.1, 170.7, 164.5, 150.2, 147.0, 115.5, 108.7, 68.4, 53.3, 46.2, 42.6, 42.1, 39.4, 38.0, 34.4, 24.1; FTIR (cm^{-1}): 3278, 3061, 2950, 2868, 1665, 1614, 1565, 1399, 1225, 1046, 734; HRMS (ESI) m/z, calculated for [$C_{16}H_{22}N_3O_3^+$]: 304.1656; found: 304.1659.

6. Crystallographic Details

X-ray structural analysis for **36**: Crystals were mounted using viscous oil onto a plastic mesh and cooled to the data collection temperature (100K). Data were collected on a Bruker-AXS APEX II DUO CCD diffractometer with Cu-Kα radiation (λ = 1.54178 Å) focused Mo-Kα radiation (λ = 0.71073 Å). Unit cell parameters were obtained from 36 to 48 data frames, 0.5° ω, from different sections of the Ewald sphere. The unit-cell dimensions, equivalent reflections and systematic absences in the diffraction data are consistent, uniquely, with $P2_1/c$. The data were treated with multi-scan absorption corrections.[7] Structures were solved using intrinsic phasing methods[8] and refined with full-matrix, least-squares procedures on F^2.[9] The structures have been deposited at the Cambridge Structural Database under the following CCDC deposition numbers: 2071160.

X-ray structural analysis for **42**: Crystals were mounted using viscous oil onto a plastic mesh and cooled to the data collection temperature (150K). Data were collected on a Bruker-AXS APEX II DUO CCD diffractometer with Cu-Kα radiation (λ = 1.54178 Å) focused with Goebel mirrors. Unit cell parameters were obtained from 36 to 48 data frames, 0.5° ω, from different sections of the Ewald sphere. No symmetry higher than triclinic was observed and refinement in the centrosymmetric space group option, *P*-1, yielded chemically reasonable and computationally stable results of refinement. The data were treated with multi-scan absorption corrections.[7] Structures were solved using intrinsic phasing methods[8] and refined with full-matrix, least-squares procedures on F^2.[9] Two symmetry-unique but chemically identical compound molecules were found in the asymmetric unit (i.e. Z = 2 and Z' = 2) together with a chloroform solvent molecule, disordered in two positions, with chemically equivalent atoms in non-crystallographical symmetry restrained disordered contributions treated with equal atomic displacement parameter restraints, having 88/12 refined site occupancy ratio. Non-hydrogen atoms were refined with anisotropic displacement parameters. Other than the H-atom on each of the disordered chloroform solvent molecule locations, treated as idealized contributions with geometrically calculated positions and with U_{iso} equal to 1.2 U_{eq} of the attached carbon atom, all other H-atoms were located from the difference map and refined independently with isotropic parameters. Atomic scattering factors are contained in the SHELXTL program library.[9] The structures have been deposited at the Cambridge Structural Database under the following CCDC deposition numbers: 2071161.

X-ray structural analysis for **43**: Crystals were mounted using viscous oil onto a plastic mesh and cooled to the data collection temperature (150K). Data were collected on a Bruker-AXS APEX II DUO CCD diffractometer with Cu-Kα radiation (λ = 1.54178 Å) focused Mo-Kα radiation (λ = 0.71073 Å). Unit cell parameters were obtained from 36 to 48 data frames, 0.5° ω, from different sections of the Ewald sphere. The unit-cell dimensions, equivalent reflections and systematic absences in the diffraction data are consistent, uniquely, with $P2_1/n$. The data were treated with multi-scan absorption corrections.[7] Structures were solved using intrinsic phasing methods[8] and refined with full-matrix, least-squares procedures on F^2.[9] The structures have been deposited at the Cambridge Structural Database under the following CCDC deposition numbers: 2071162.

7. References

(1) A. B. Pangborn, M. A. Giardello, R. H. Grubbs, R. K. Rosen and F. J. Timmers, *Organometallics*, 1996, **15**, 1518
(2) J. R. McAtee, S. E. S. Martin, D. T. Ahneman, K. A. Johnson and D. A. Watson, *Angew. Chem. Int. Ed.*, 2012, **51**, 3663
(3) B. N. Hemric, K. Shen and Q. Wang, *J. Am. Chem. Soc.*, 2016, **138**, 5813
(4) Z. Pan, S. Wang, J. T. Brethorst and C. J. Douglas, *J. Am. Chem. Soc.,* 2018, **140**, 3331
(5) A. B. Smith III, B. H. Toder, S. J. Branca and R. K. Dieter, *J. Am. Chem. Soc.*, 1981, **103**, 1996
(6) S. A. Shuler, G. Yin, S. B. Krause, C. M. Vesper and D. A. Watson, *J. Am. Chem. Soc*., 2016, **138**, 13830
(7) Apex3, Bruker AXS Inc.: Madison, WI, 2015
(8) G. M. Sheldrick, *Acta. Cryst*., **2015**, *A71*, 3
(9) G. M. Sheldrick, *Acta. Cryst*., **2015**, *C71*, 3

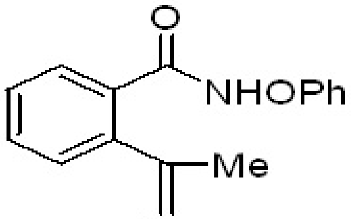

1

	Parameter	Value
1	Title	grd-1-143.1.fid
2	Solvent	DMSO
3	Temperature	300.0
4	Number of Scans	16
5	Receiver Gain	57.0
6	Relaxation Delay	1.0000
7	Pulse Width	10.6100
8	Spectrometer Frequency	600.32
9	Nucleus	1H

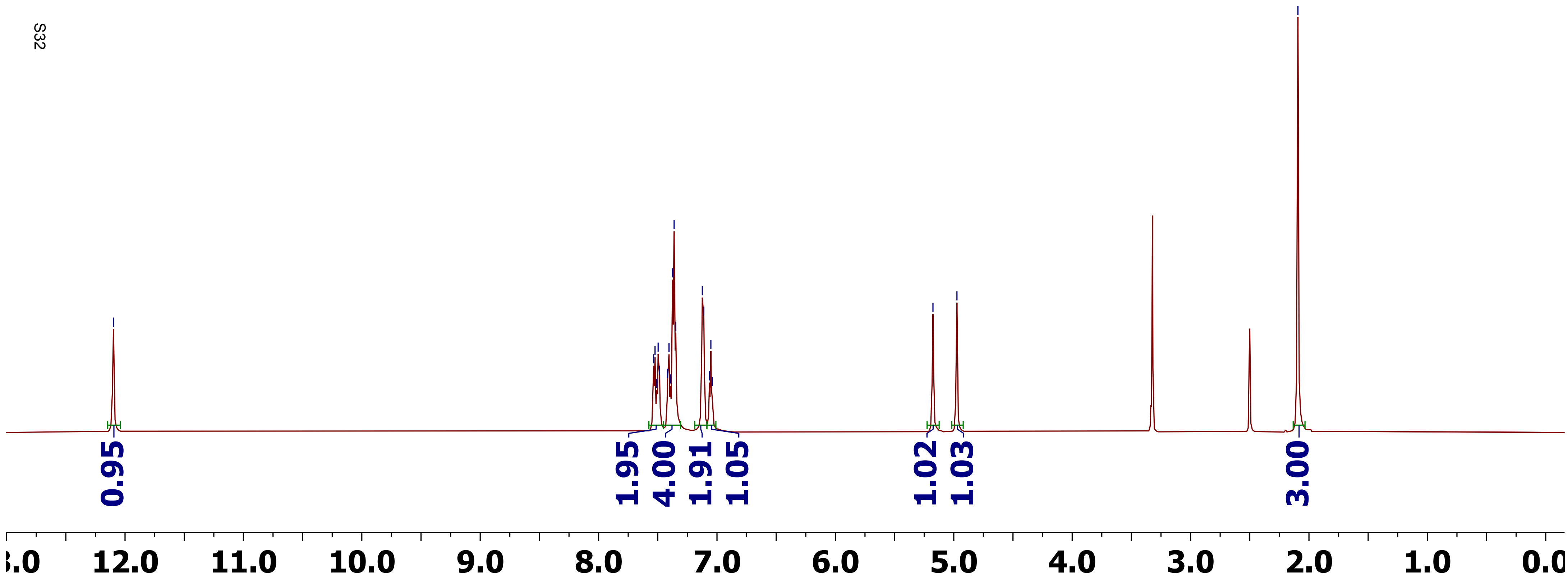

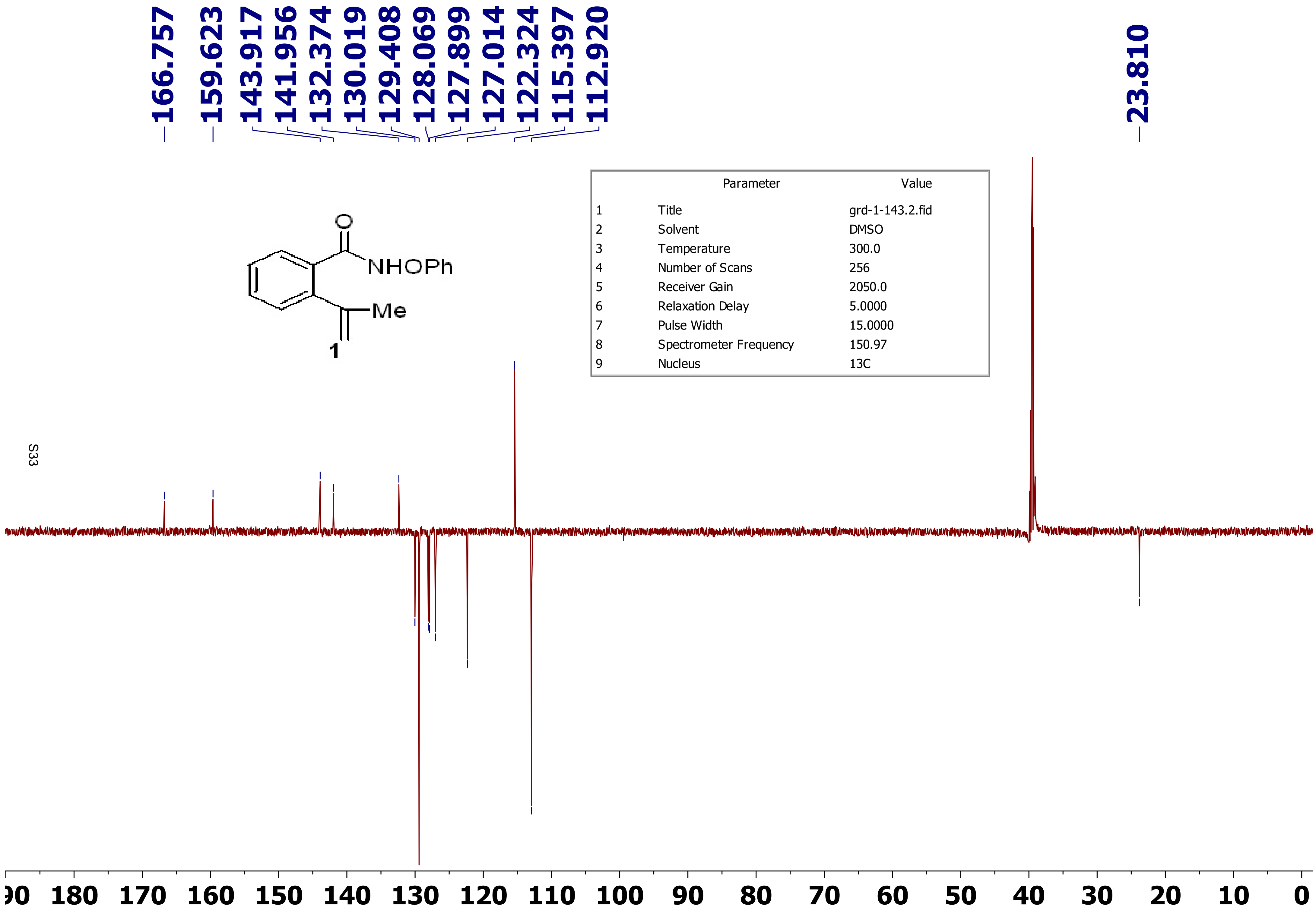

	Parameter	Value
1	Title	grd-1-143.2.fid
2	Solvent	DMSO
3	Temperature	300.0
4	Number of Scans	256
5	Receiver Gain	2050.0
6	Relaxation Delay	5.0000
7	Pulse Width	15.0000
8	Spectrometer Frequency	150.97
9	Nucleus	13C

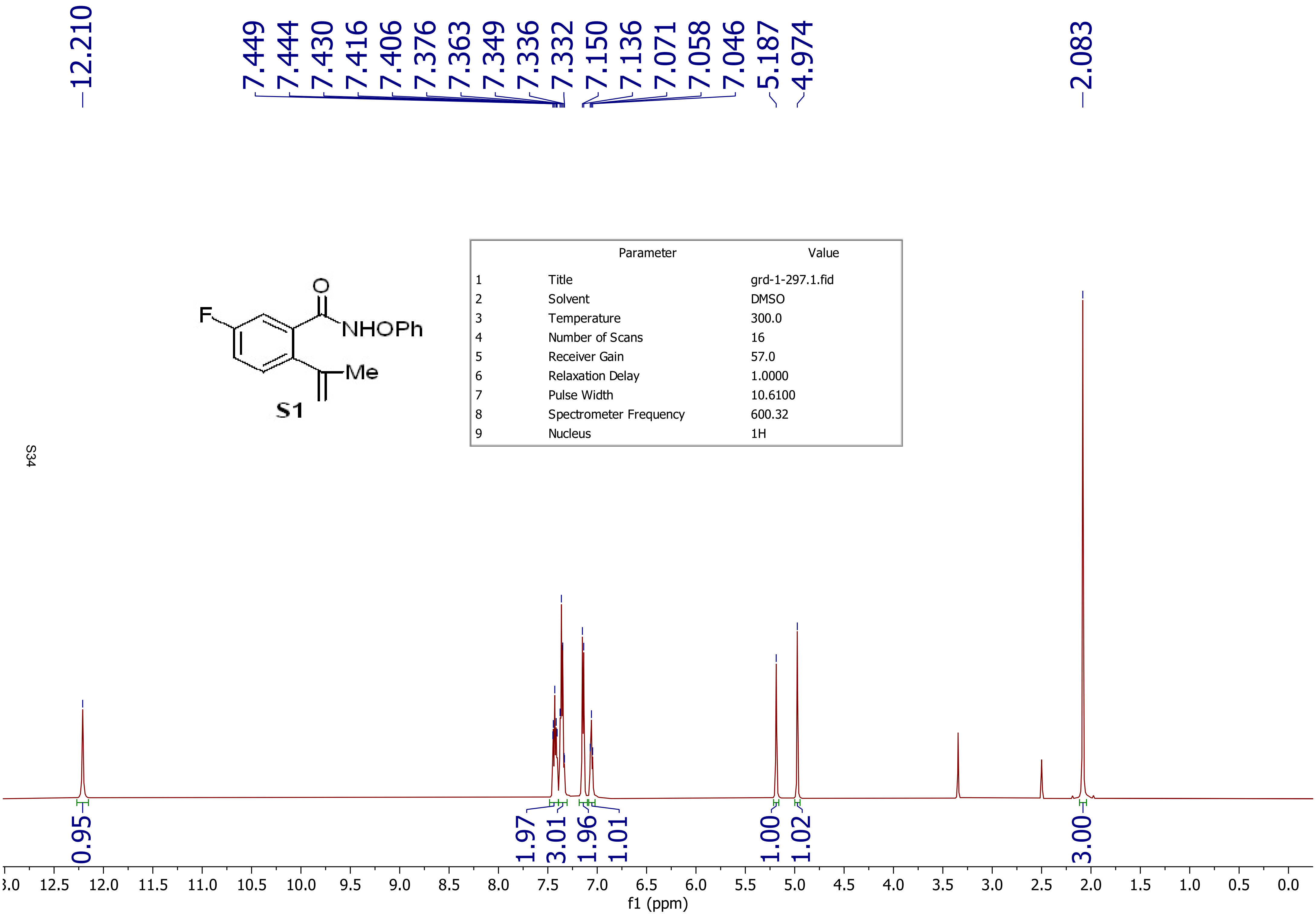

S1

	Parameter	Value
1	Title	grd-1-297.1.fid
2	Solvent	DMSO
3	Temperature	300.0
4	Number of Scans	16
5	Receiver Gain	57.0
6	Relaxation Delay	1.0000
7	Pulse Width	10.6100
8	Spectrometer Frequency	600.32
9	Nucleus	1H

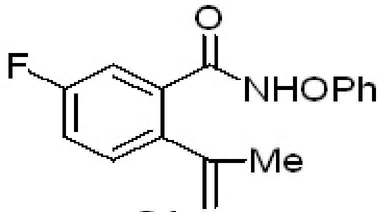

S1

	Parameter	Value
1	Title	grd-1-297.2.fid
2	Solvent	DMSO
3	Temperature	300.0
4	Number of Scans	256
5	Receiver Gain	2050.0
6	Relaxation Delay	5.0000
7	Pulse Width	15.0000
8	Spectrometer Frequency	150.97
9	Nucleus	13C

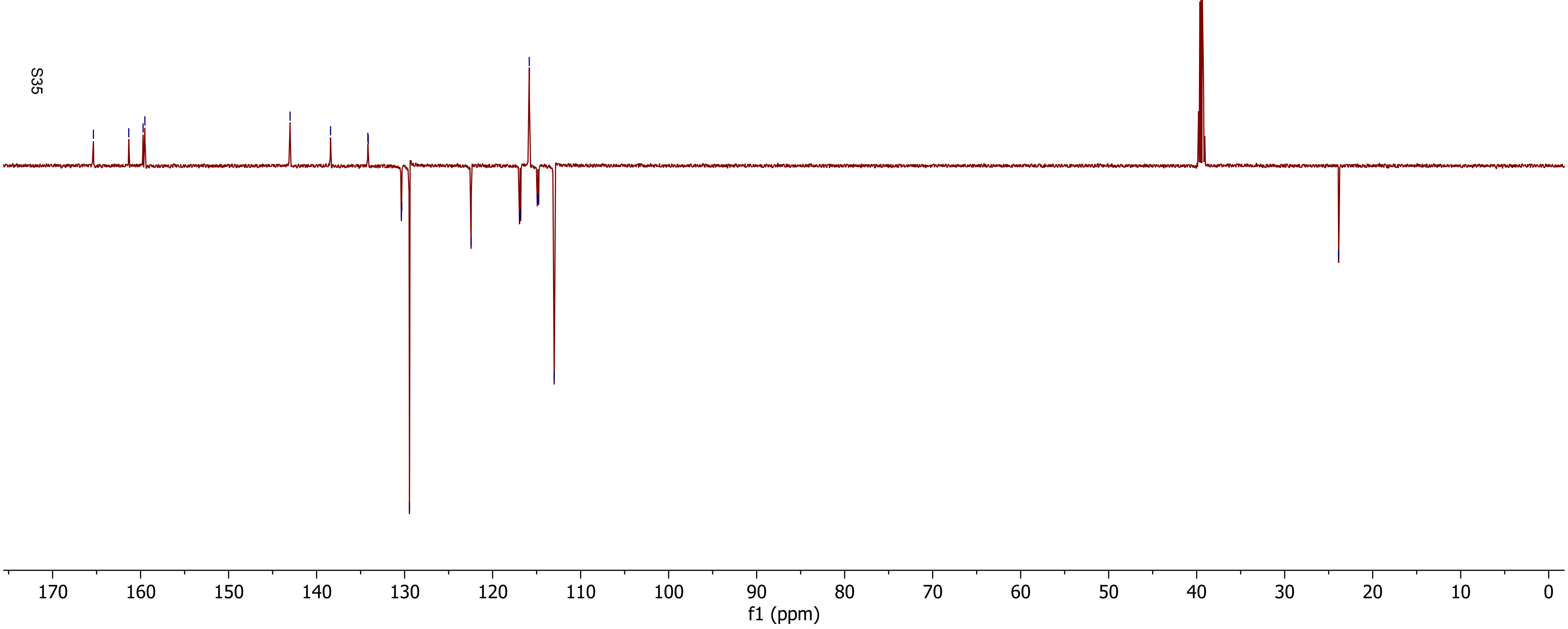

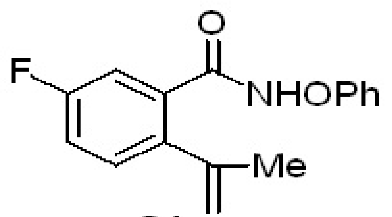

S1

	Parameter	Value
1	Title	grd-1-297.3.fid
2	Solvent	DMSO
3	Temperature	300.0
4	Number of Scans	32
5	Receiver Gain	144.0
6	Relaxation Delay	3.0000
7	Pulse Width	11.4000
8	Spectrometer Frequency	564.81
9	Nucleus	19F

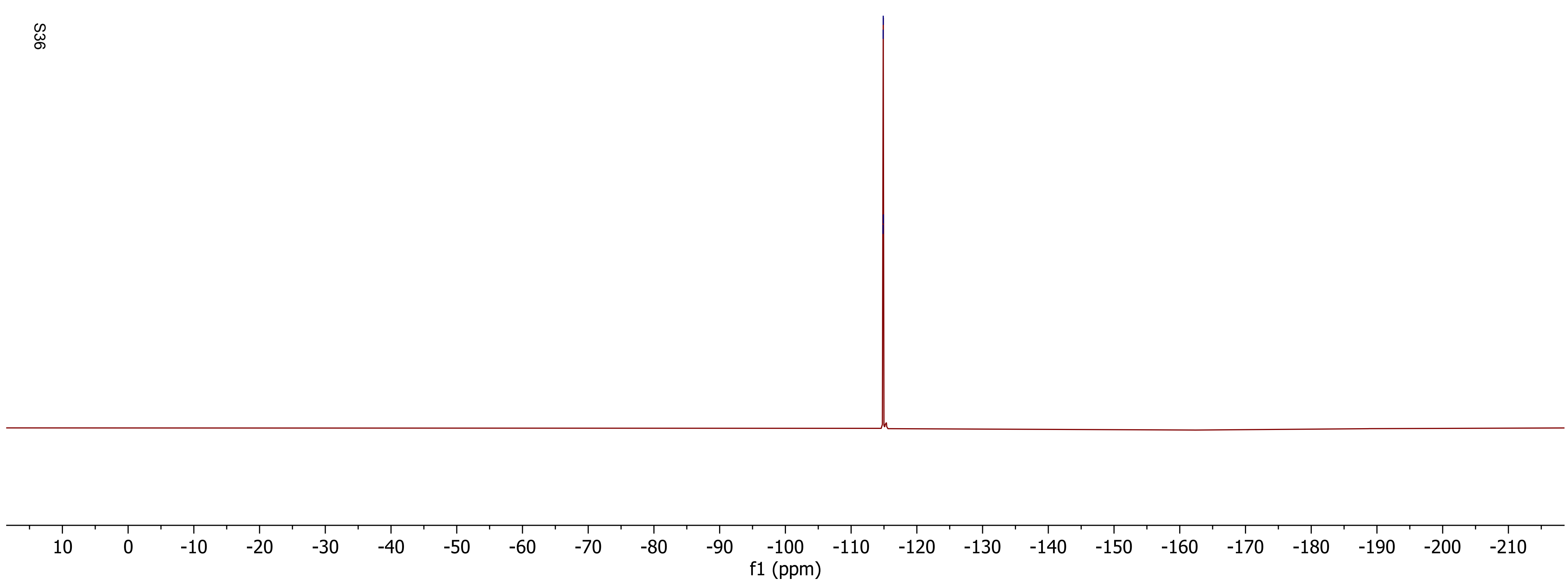

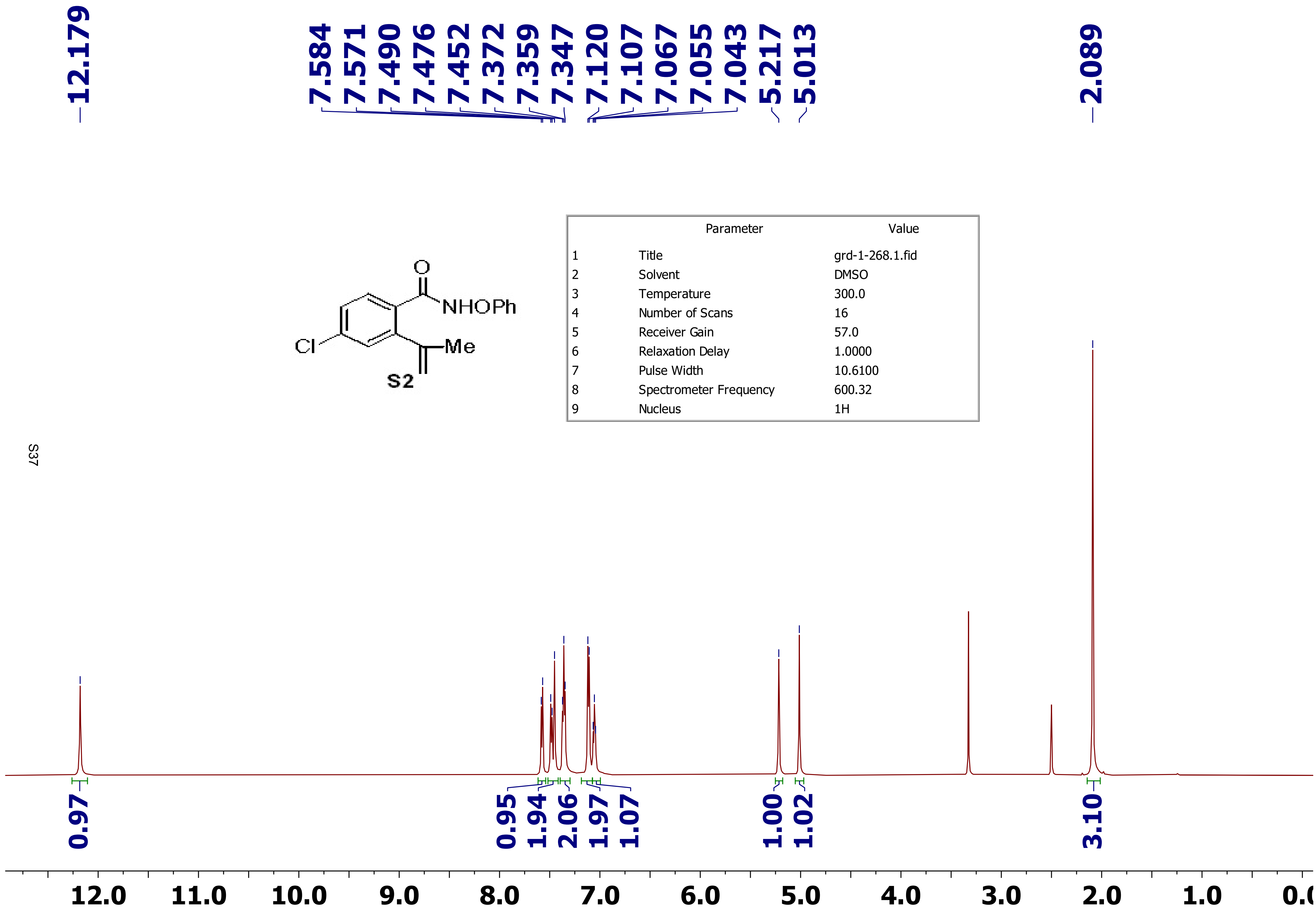

S2

	Parameter	Value
1	Title	grd-1-268.1.fid
2	Solvent	DMSO
3	Temperature	300.0
4	Number of Scans	16
5	Receiver Gain	57.0
6	Relaxation Delay	1.0000
7	Pulse Width	10.6100
8	Spectrometer Frequency	600.32
9	Nucleus	1H

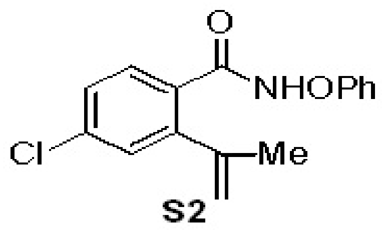

S2

	Parameter	Value
1	Title	grd-1-268.2.fid
2	Solvent	DMSO
3	Temperature	300.0
4	Number of Scans	256
5	Receiver Gain	2050.0
6	Relaxation Delay	5.0000
7	Pulse Width	15.0000
8	Spectrometer Frequency	150.97
9	Nucleus	13C

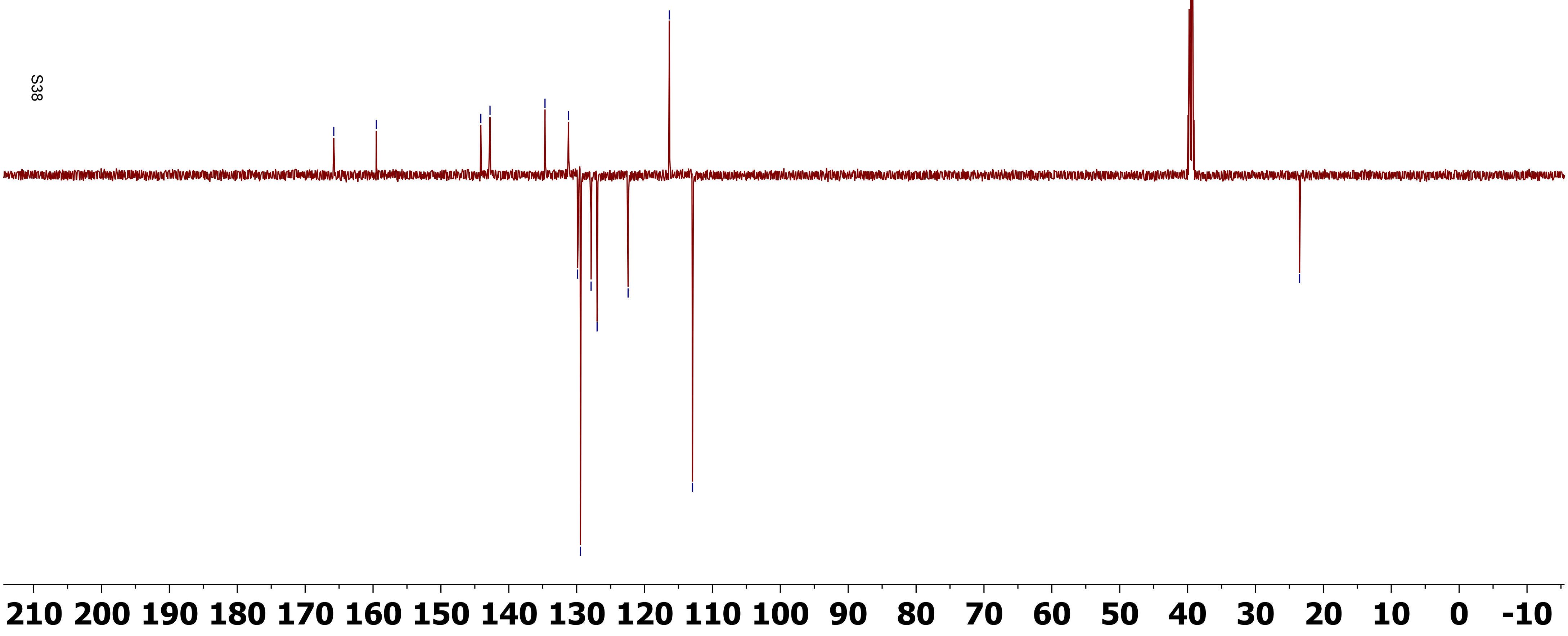

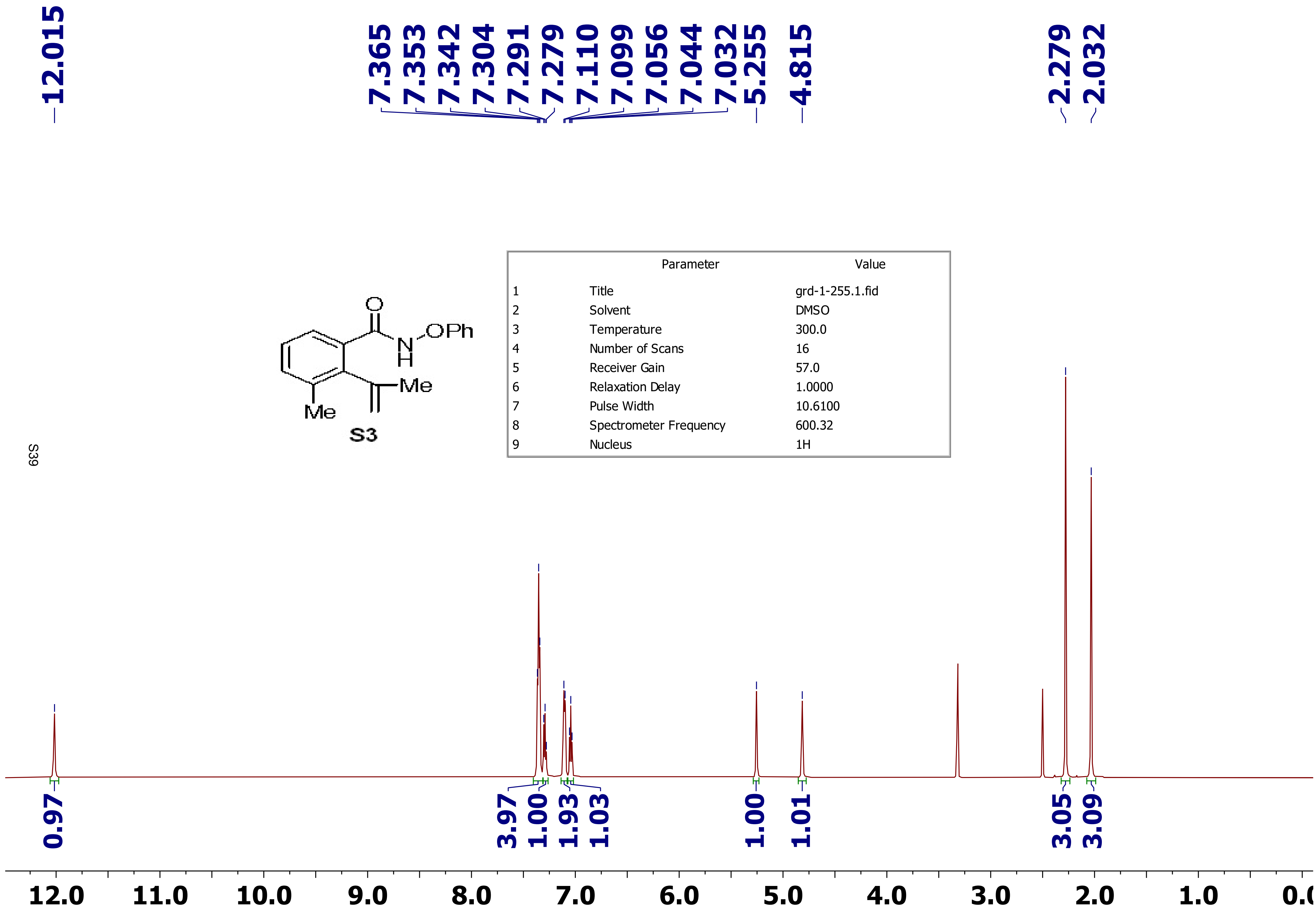

S3

	Parameter	Value
1	Title	grd-1-255.1.fid
2	Solvent	DMSO
3	Temperature	300.0
4	Number of Scans	16
5	Receiver Gain	57.0
6	Relaxation Delay	1.0000
7	Pulse Width	10.6100
8	Spectrometer Frequency	600.32
9	Nucleus	1H

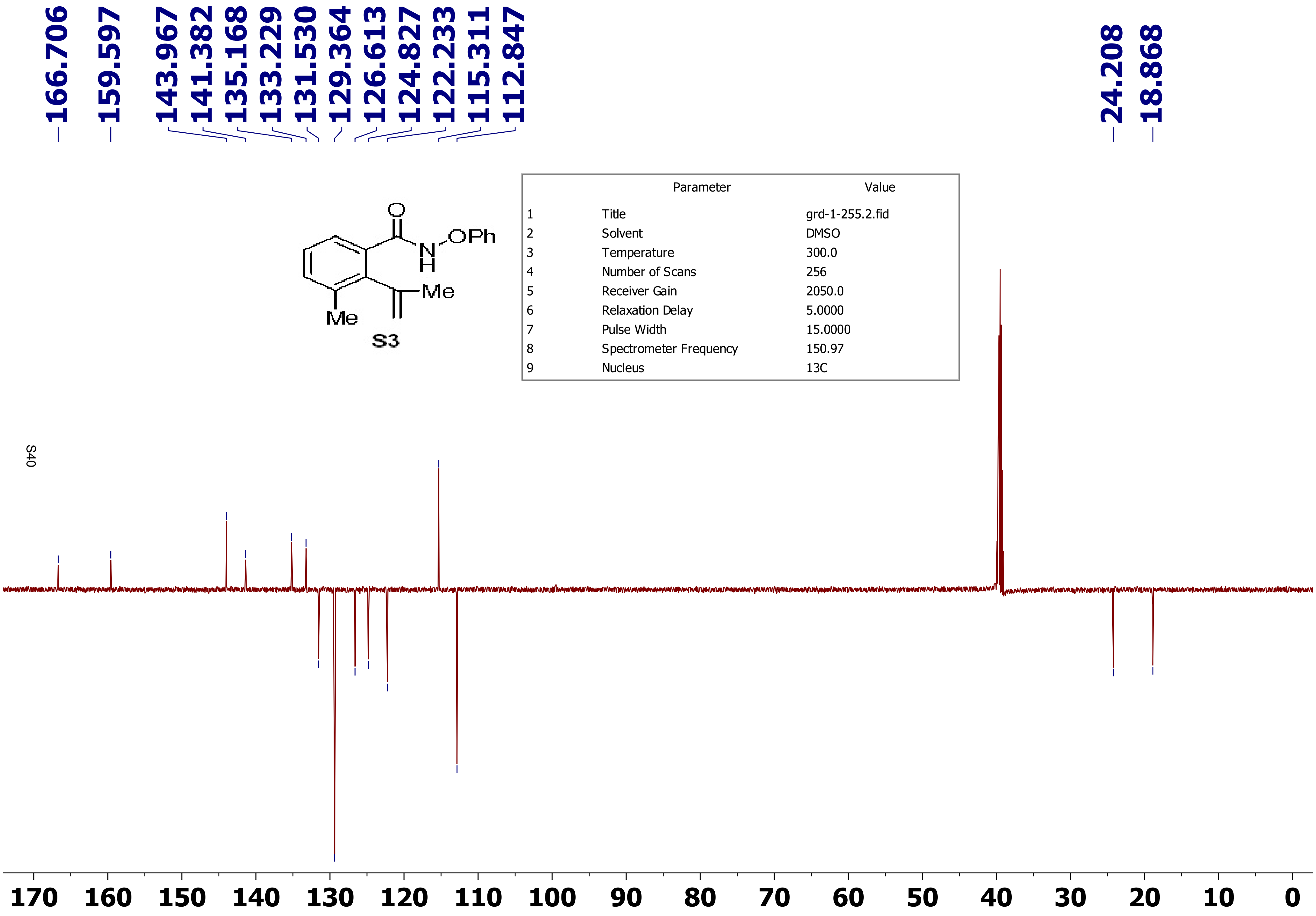

S3

	Parameter	Value
1	Title	grd-1-255.2.fid
2	Solvent	DMSO
3	Temperature	300.0
4	Number of Scans	256
5	Receiver Gain	2050.0
6	Relaxation Delay	5.0000
7	Pulse Width	15.0000
8	Spectrometer Frequency	150.97
9	Nucleus	13C

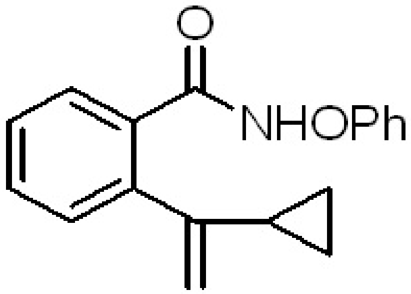

	Parameter	Value
1	Title	grd-1-232.1.fid
2	Solvent	DMSO
3	Temperature	300.0
4	Number of Scans	16
5	Receiver Gain	57.0
6	Relaxation Delay	1.0000
7	Pulse Width	10.6100
8	Spectrometer Frequency	600.32
9	Nucleus	1H

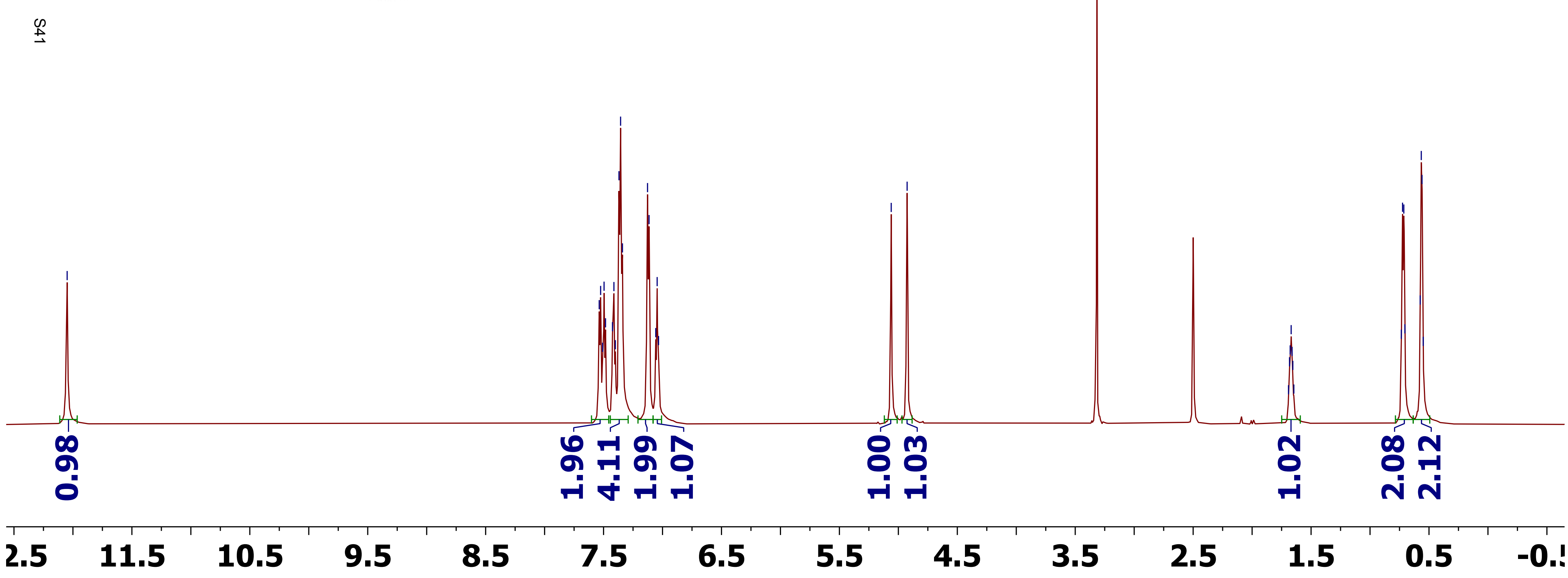

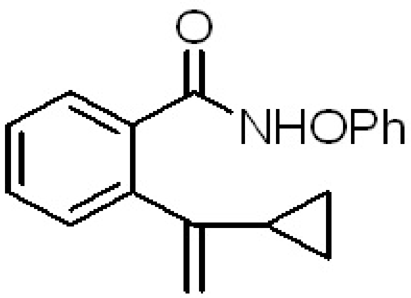

S4

	Parameter	Value
1	Title	grd-1-232.2.fid
2	Solvent	DMSO
3	Temperature	300.0
4	Number of Scans	256
5	Receiver Gain	2050.0
6	Relaxation Delay	5.0000
7	Pulse Width	15.0000
8	Spectrometer Frequency	150.97
9	Nucleus	13C

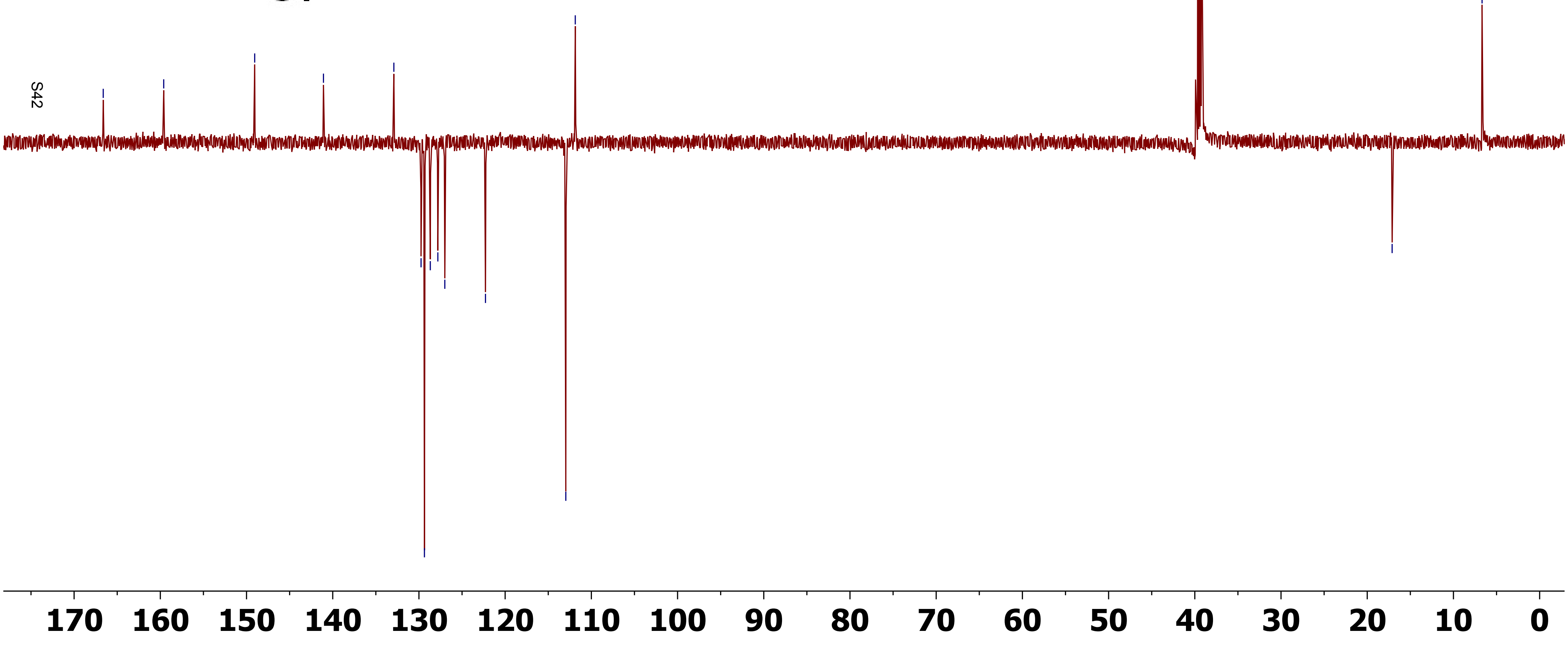

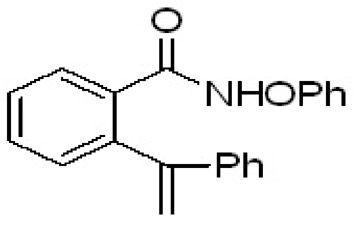

S5

	Parameter	Value
1	Title	grd-1-207.1.fid
2	Solvent	DMSO
3	Temperature	300.0
4	Number of Scans	16
5	Receiver Gain	57.0
6	Relaxation Delay	1.0000
7	Pulse Width	10.6100
8	Spectrometer Frequency	600.32
9	Nucleus	1H

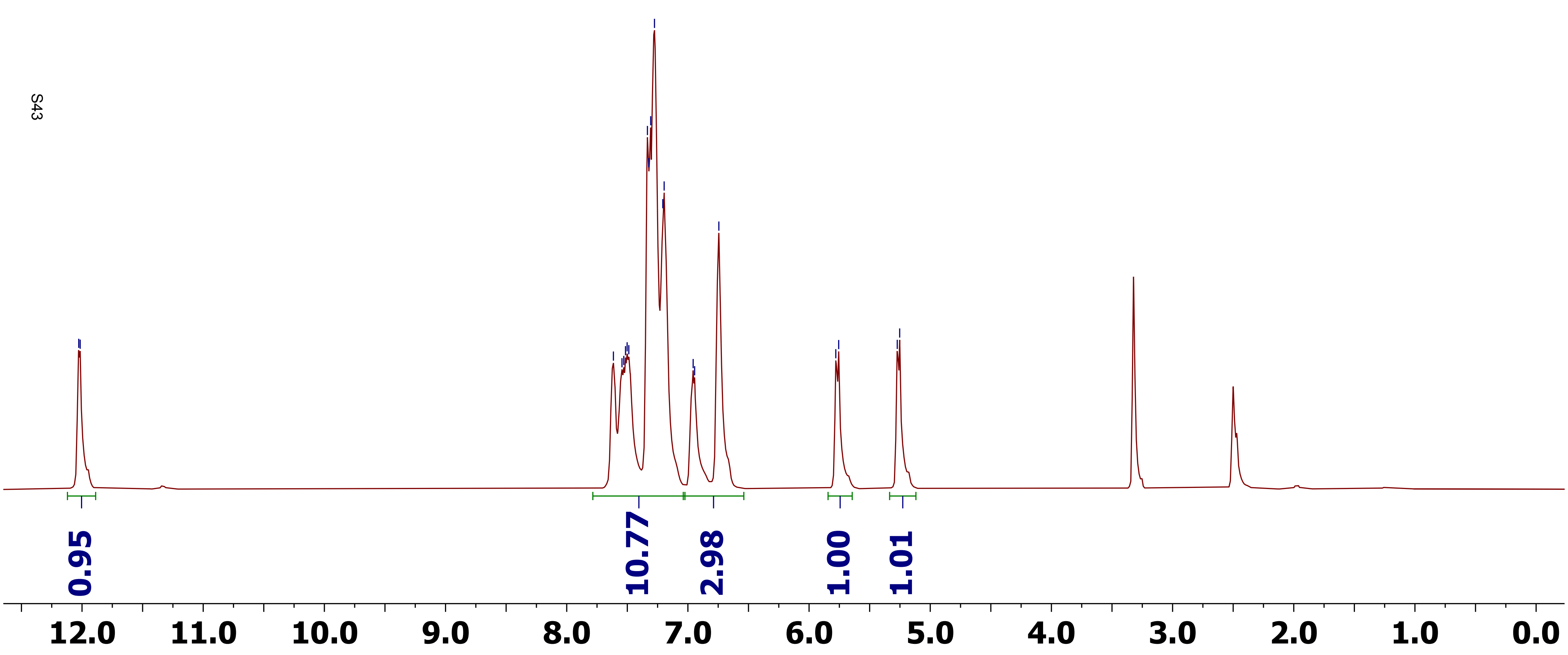

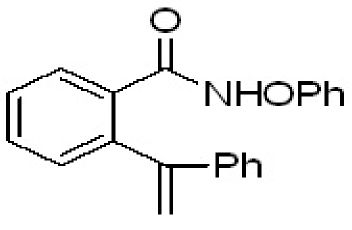

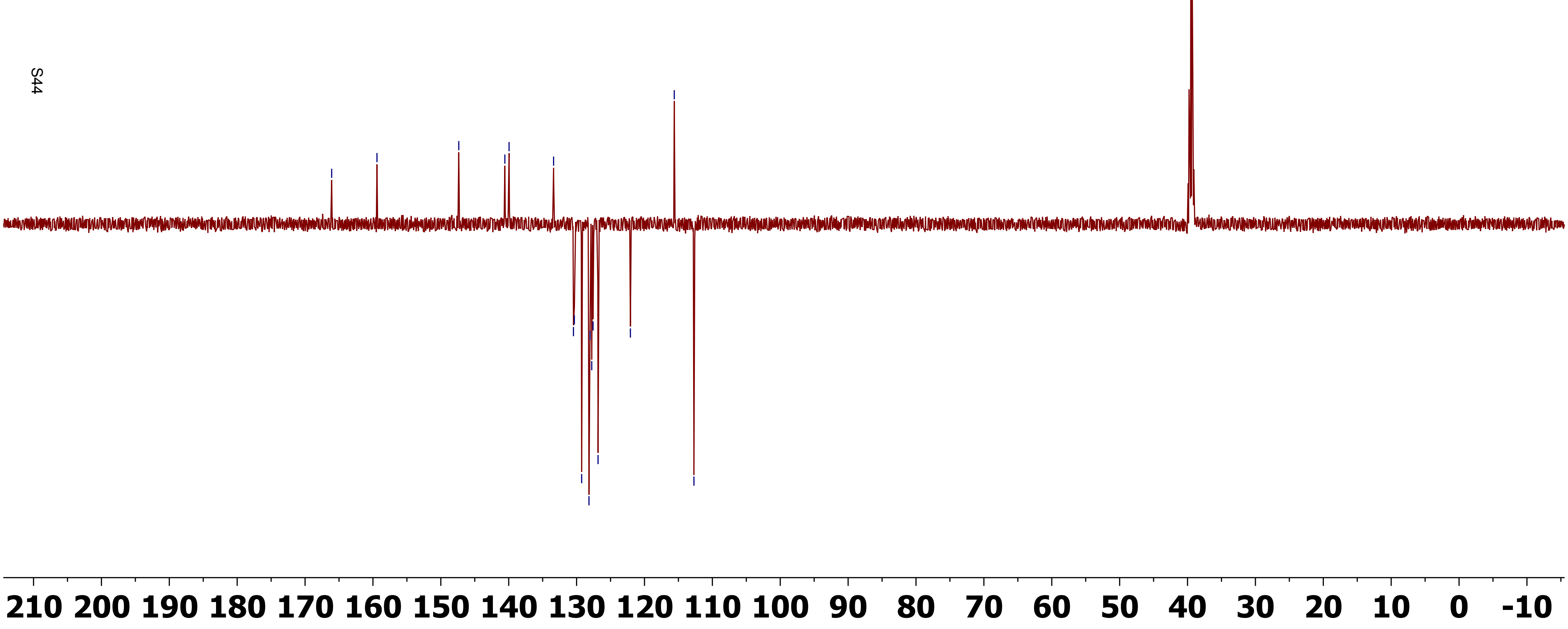

S5

	Parameter	Value
1	Title	grd-1-207.2.fid
2	Solvent	DMSO
3	Temperature	300.0
4	Number of Scans	256
5	Receiver Gain	2050.0
6	Relaxation Delay	5.0000
7	Pulse Width	15.0000
8	Spectrometer Frequency	150.97
9	Nucleus	13C

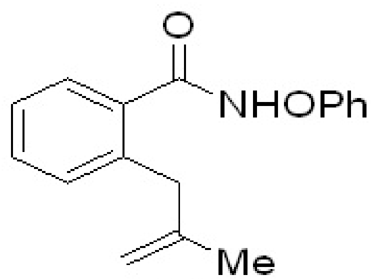

S6

	Parameter	Value
1	Title	grd-1-229.1.fid
2	Solvent	DMSO
3	Temperature	300.0
4	Number of Scans	16
5	Receiver Gain	57.0
6	Relaxation Delay	1.0000
7	Pulse Width	10.6100
8	Spectrometer Frequency	600.32
9	Nucleus	1H

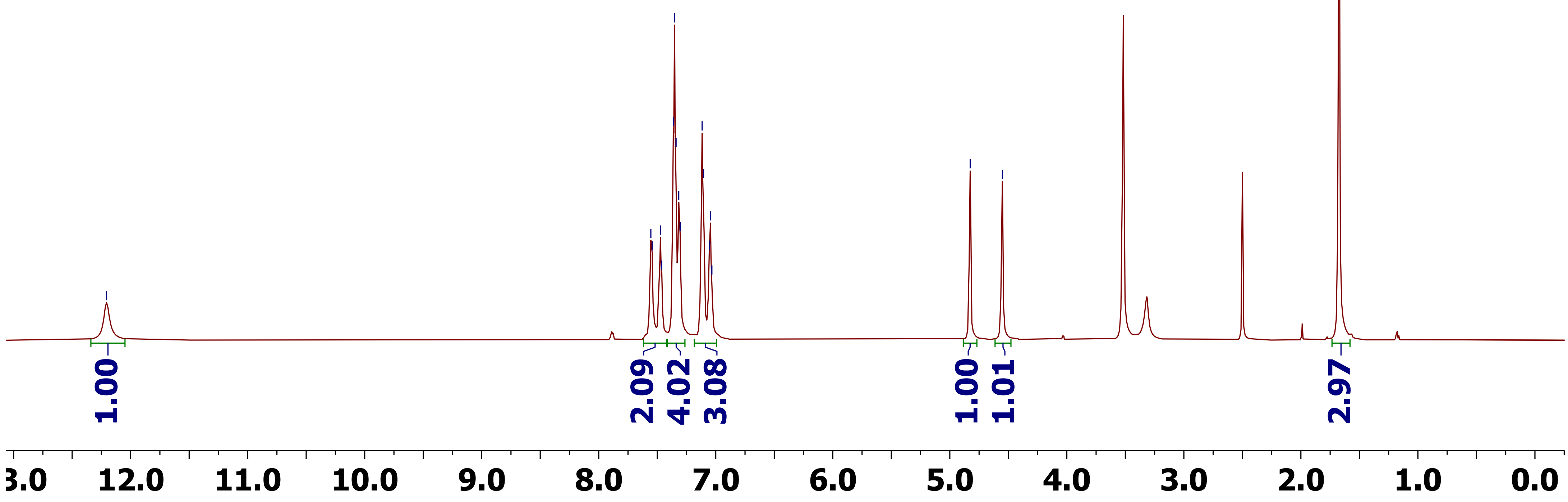

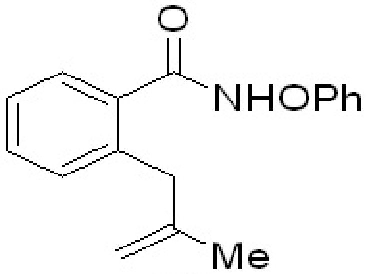

S6

	Parameter	Value
1	Title	grd-1-229.2.fid
2	Solvent	DMSO
3	Temperature	300.0
4	Number of Scans	256
5	Receiver Gain	2050.0
6	Relaxation Delay	5.0000
7	Pulse Width	15.0000
8	Spectrometer Frequency	150.97
9	Nucleus	13C

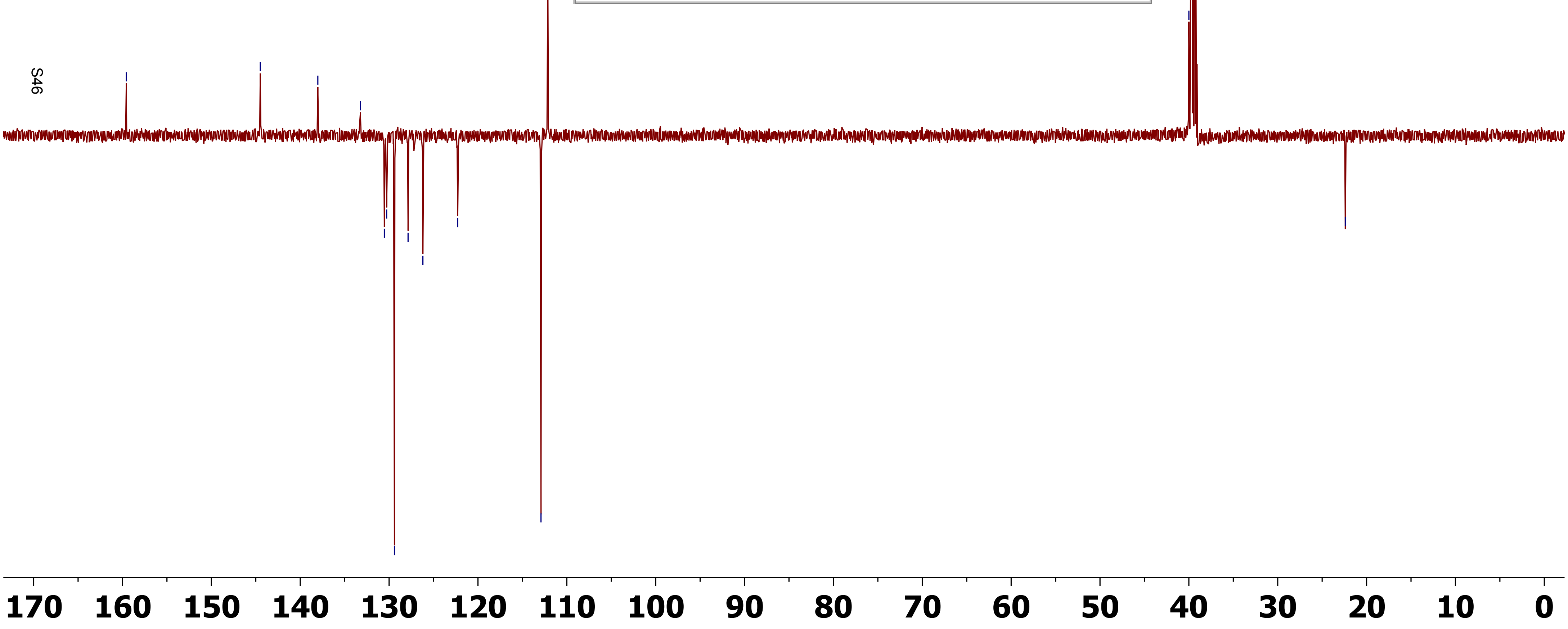

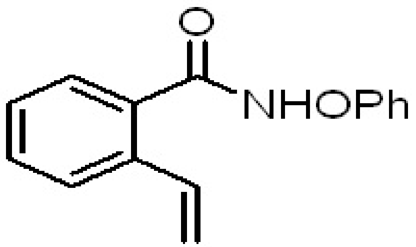

S7

	Parameter	Value
1	Title	grd-1-146.1.fid
2	Solvent	DMSO
3	Temperature	300.0
4	Number of Scans	16
5	Receiver Gain	57.0
6	Relaxation Delay	1.0000
7	Pulse Width	10.6100
8	Spectrometer Frequency	600.32
9	Nucleus	1H

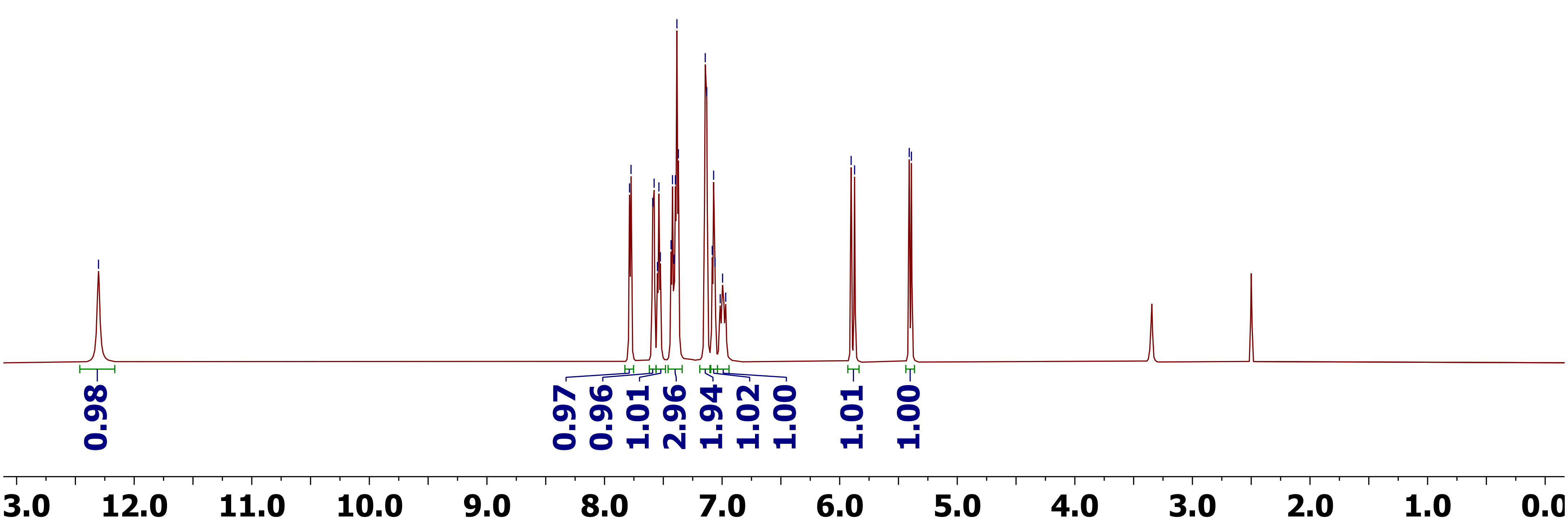

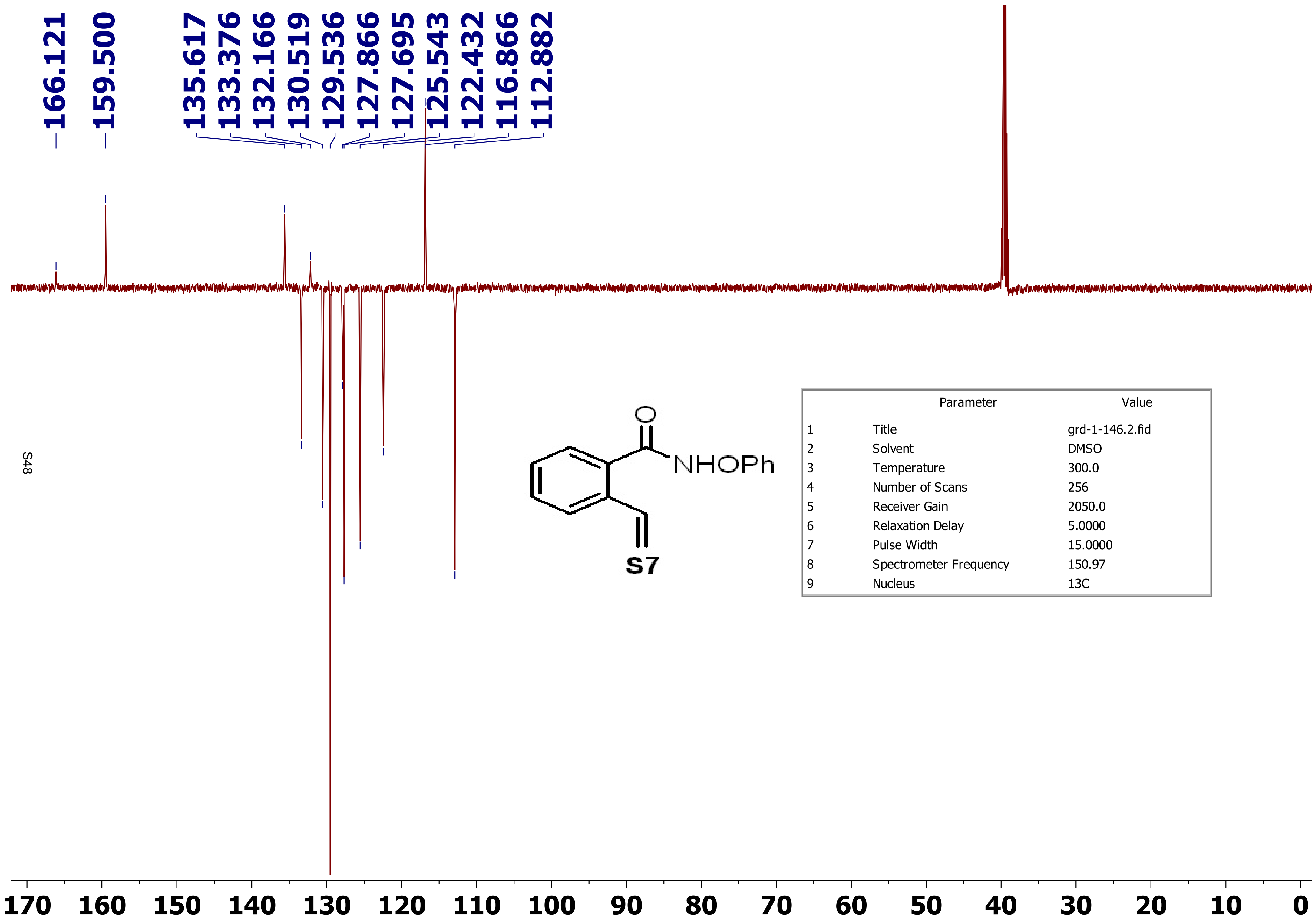

S7

	Parameter	Value
1	Title	grd-1-146.2.fid
2	Solvent	DMSO
3	Temperature	300.0
4	Number of Scans	256
5	Receiver Gain	2050.0
6	Relaxation Delay	5.0000
7	Pulse Width	15.0000
8	Spectrometer Frequency	150.97
9	Nucleus	13C

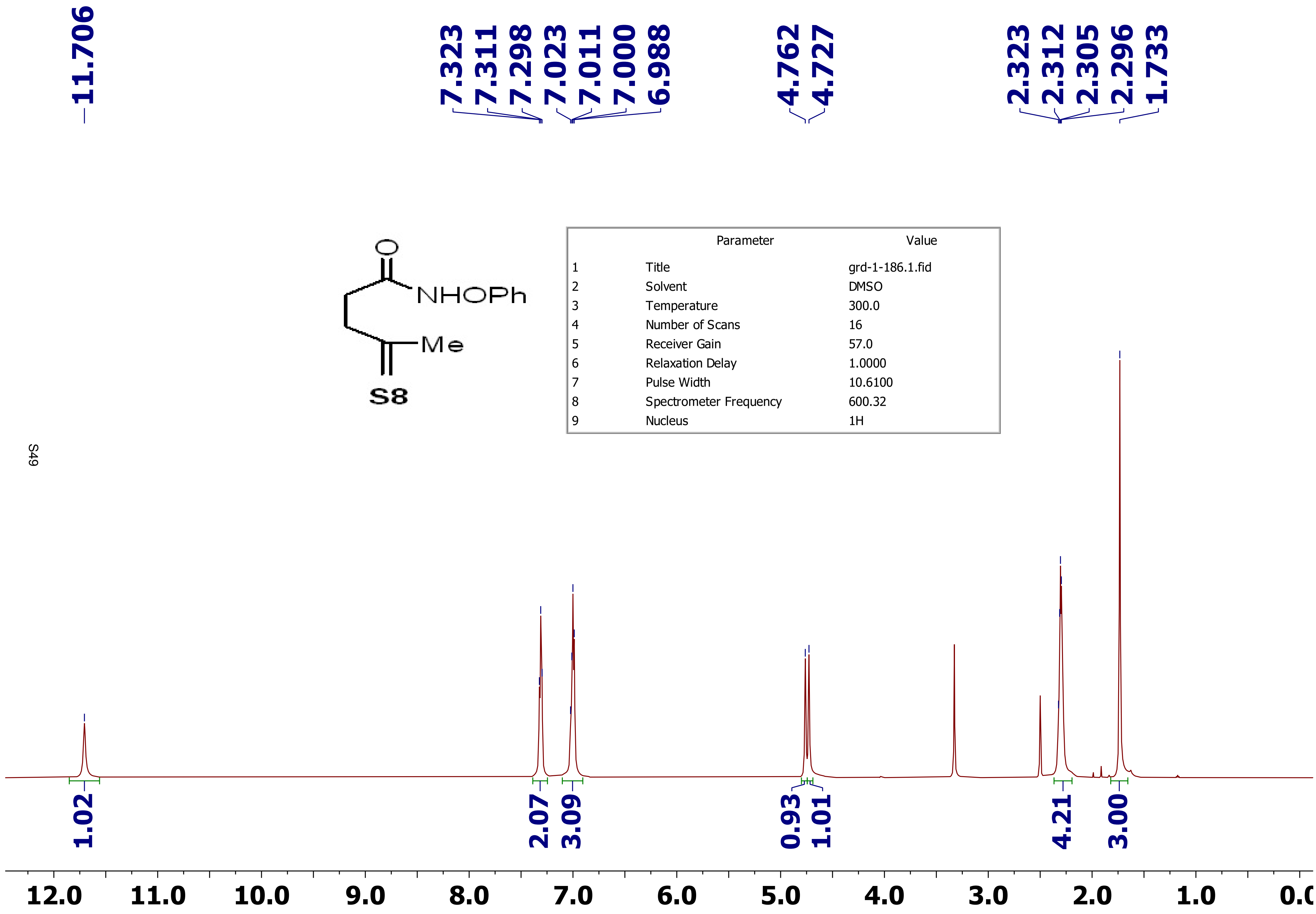

S8

	Parameter	Value
1	Title	grd-1-186.1.fid
2	Solvent	DMSO
3	Temperature	300.0
4	Number of Scans	16
5	Receiver Gain	57.0
6	Relaxation Delay	1.0000
7	Pulse Width	10.6100
8	Spectrometer Frequency	600.32
9	Nucleus	1H

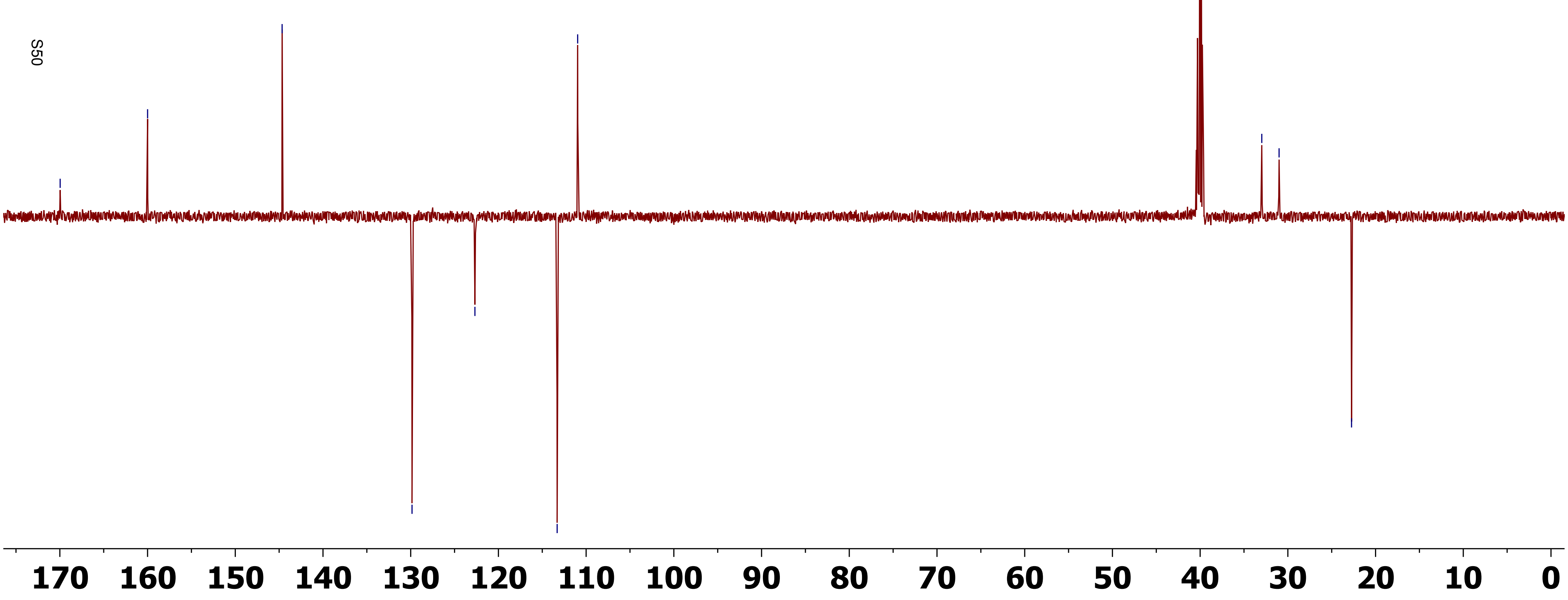

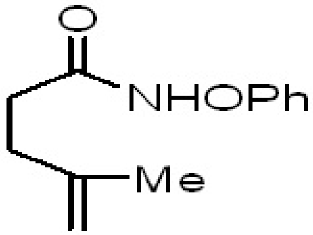

S8

	Parameter	Value
1	Title	grd-1-186.2.fid
2	Solvent	DMSO
3	Temperature	300.0
4	Number of Scans	256
5	Receiver Gain	2050.0
6	Relaxation Delay	5.0000
7	Pulse Width	15.0000
8	Spectrometer Frequency	150.97
9	Nucleus	13C

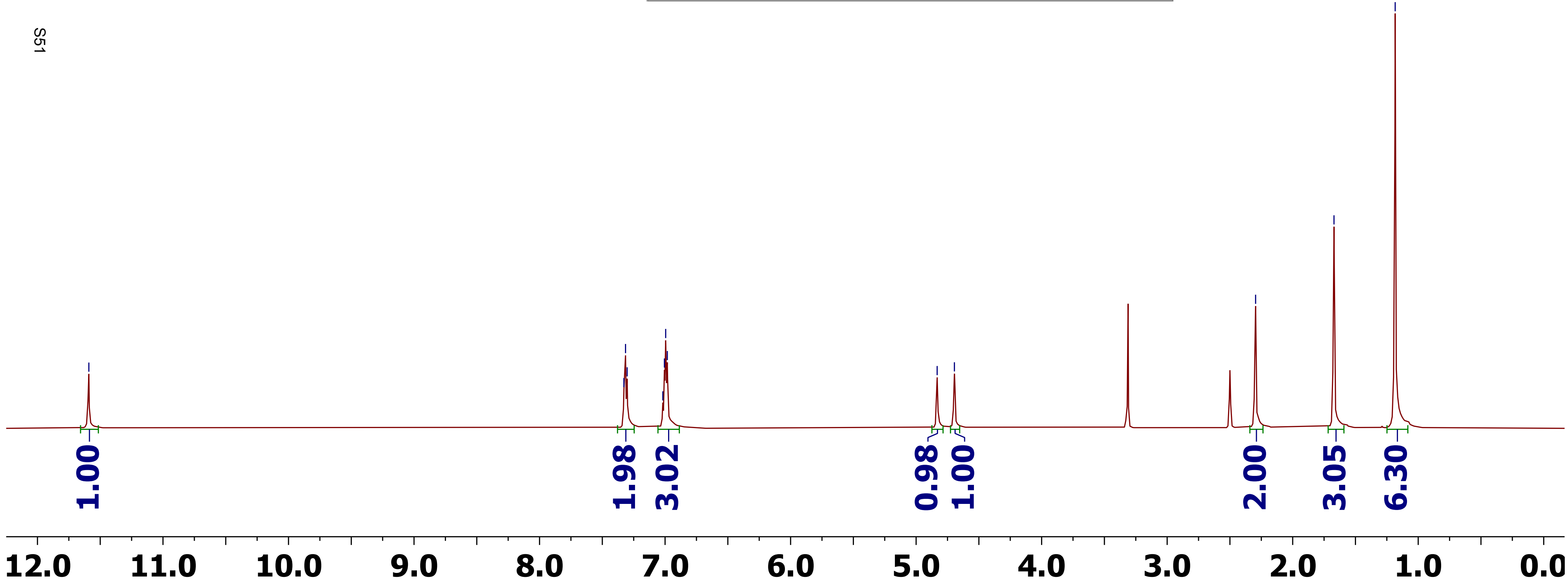

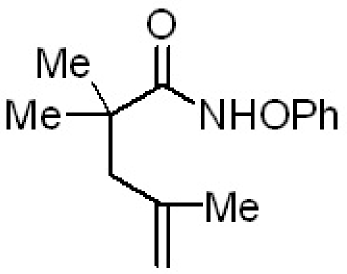

S9

	Parameter	Value
1	Title	grd-1-200.1.fid
2	Solvent	DMSO
3	Temperature	300.0
4	Number of Scans	16
5	Receiver Gain	57.0
6	Relaxation Delay	1.0000
7	Pulse Width	10.6100
8	Spectrometer Frequency	600.32
9	Nucleus	1H

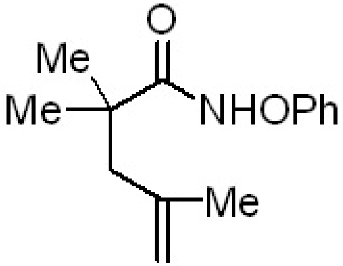

S9

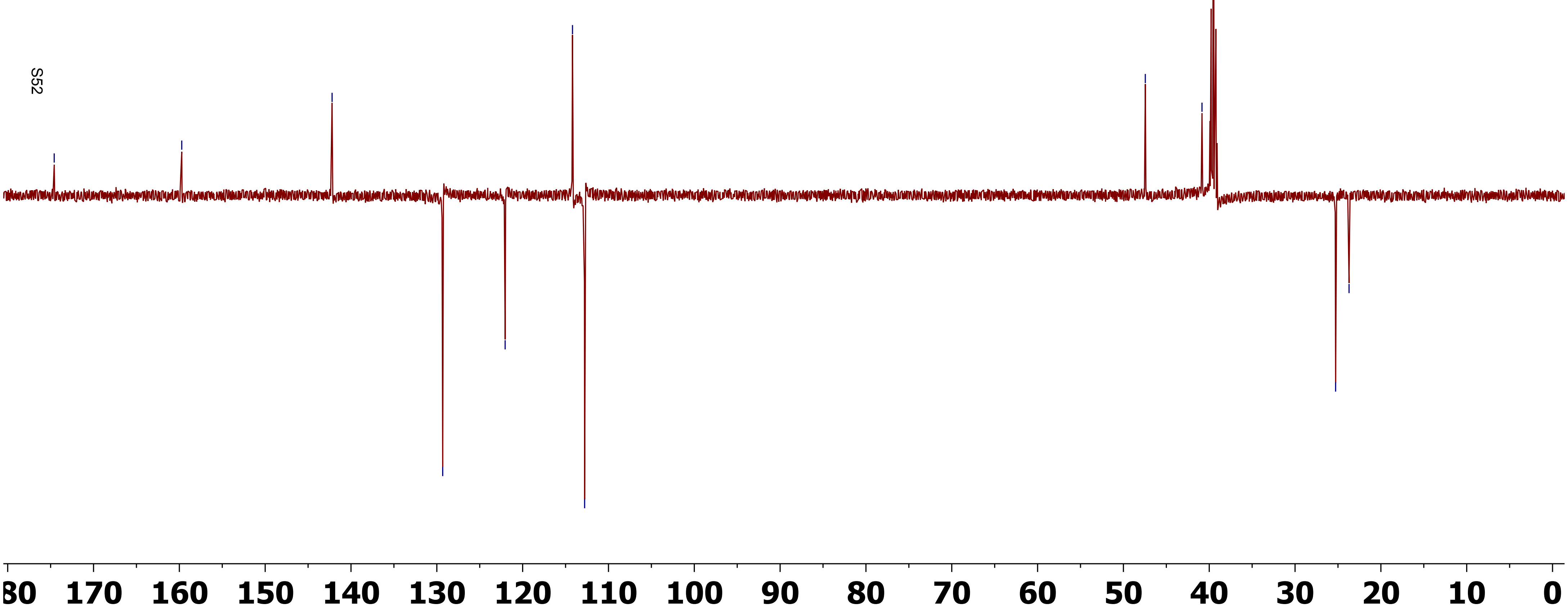

	Parameter	Value
1	Title	grd-1-200.2.fid
2	Solvent	DMSO
3	Temperature	300.0
4	Number of Scans	256
5	Receiver Gain	2050.0
6	Relaxation Delay	5.0000
7	Pulse Width	15.0000
8	Spectrometer Frequency	150.97
9	Nucleus	13C

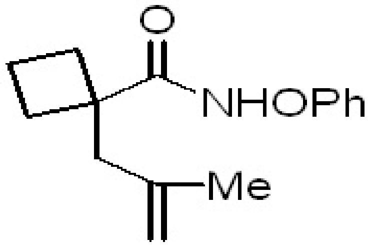

S10

	Parameter	Value
1	Title	grd-1-205.1.fid
2	Solvent	DMSO
3	Temperature	300.0
4	Number of Scans	16
5	Receiver Gain	57.0
6	Relaxation Delay	1.0000
7	Pulse Width	10.6100
8	Spectrometer Frequency	600.32
9	Nucleus	1H

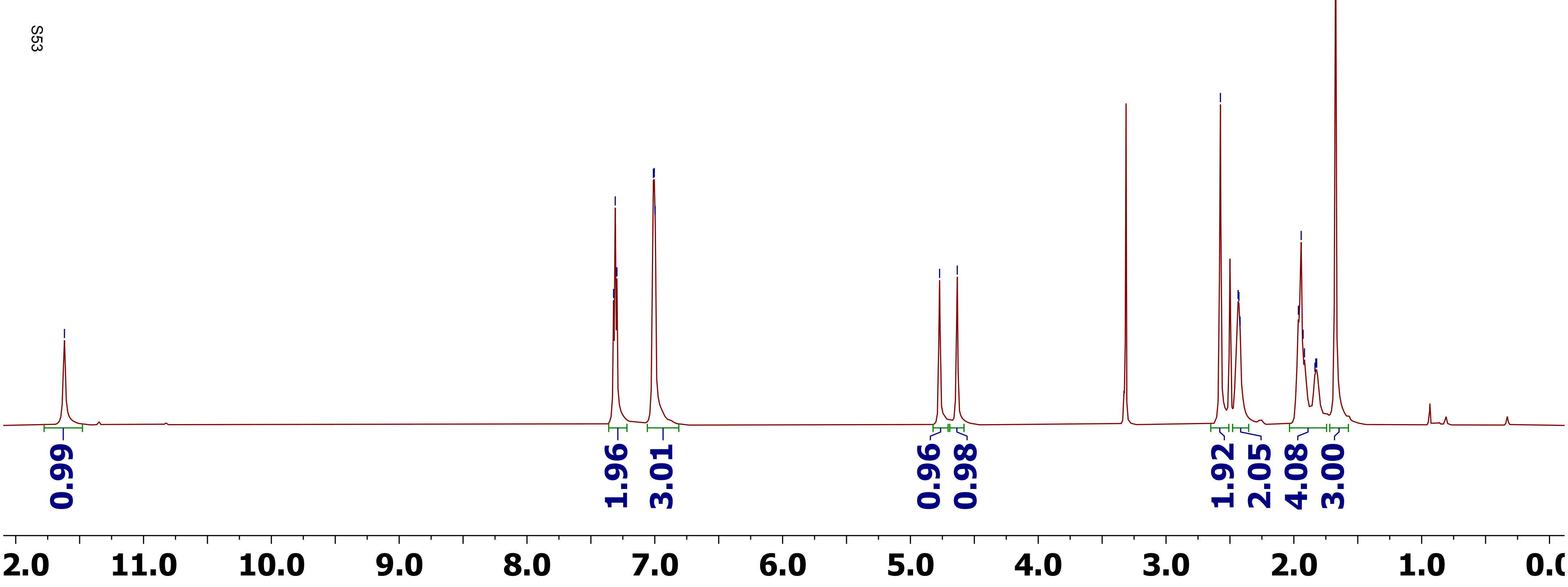

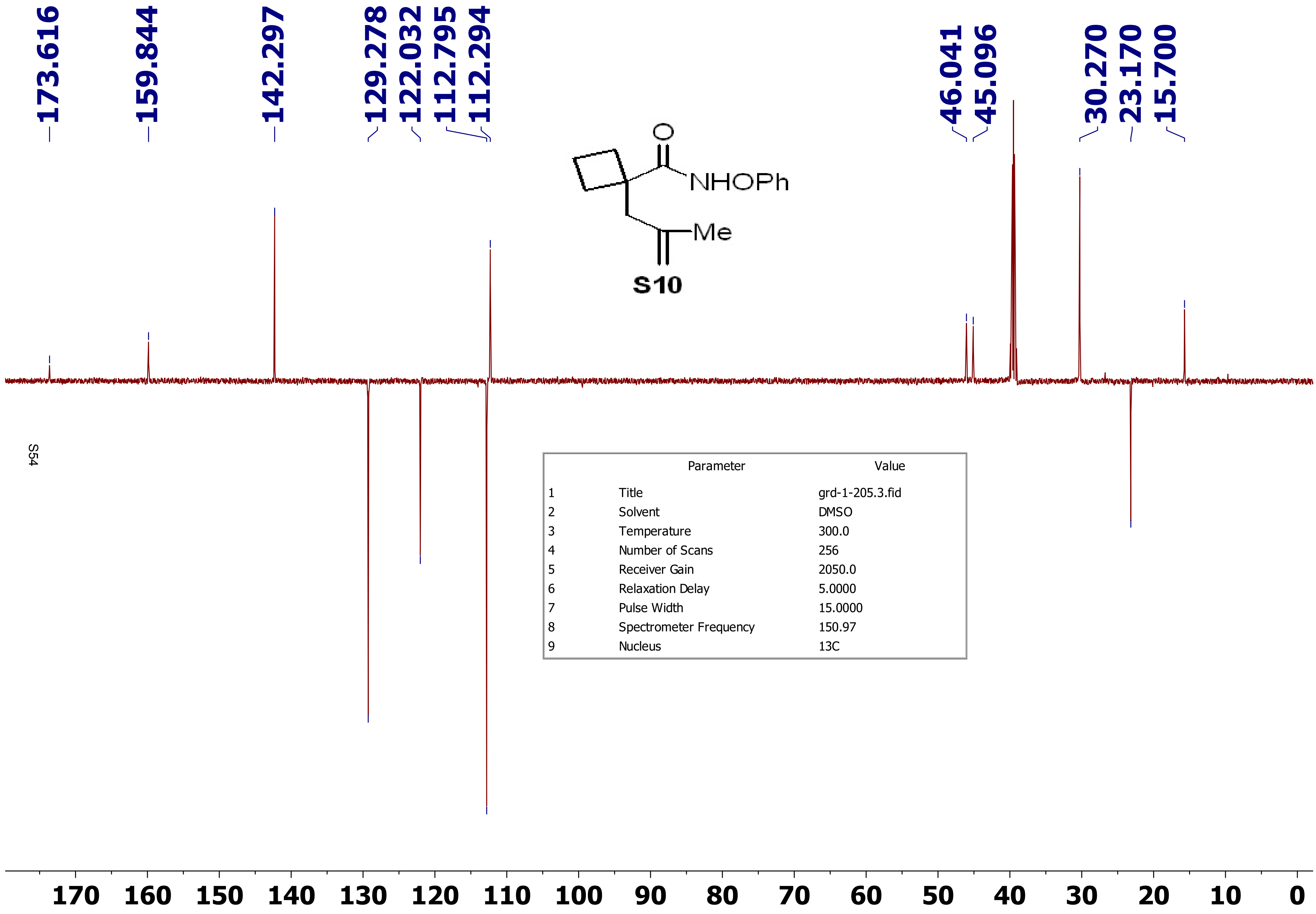

	Parameter	Value
1	Title	grd-1-205.3.fid
2	Solvent	DMSO
3	Temperature	300.0
4	Number of Scans	256
5	Receiver Gain	2050.0
6	Relaxation Delay	5.0000
7	Pulse Width	15.0000
8	Spectrometer Frequency	150.97
9	Nucleus	13C

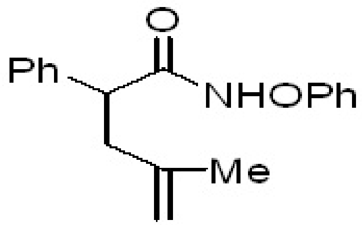

S11

	Parameter	Value
1	Title	grd-1-201.3.fid
2	Solvent	DMSO
3	Temperature	300.0
4	Number of Scans	16
5	Receiver Gain	57.0
6	Relaxation Delay	1.0000
7	Pulse Width	10.6100
8	Spectrometer Frequency	600.32
9	Nucleus	1H

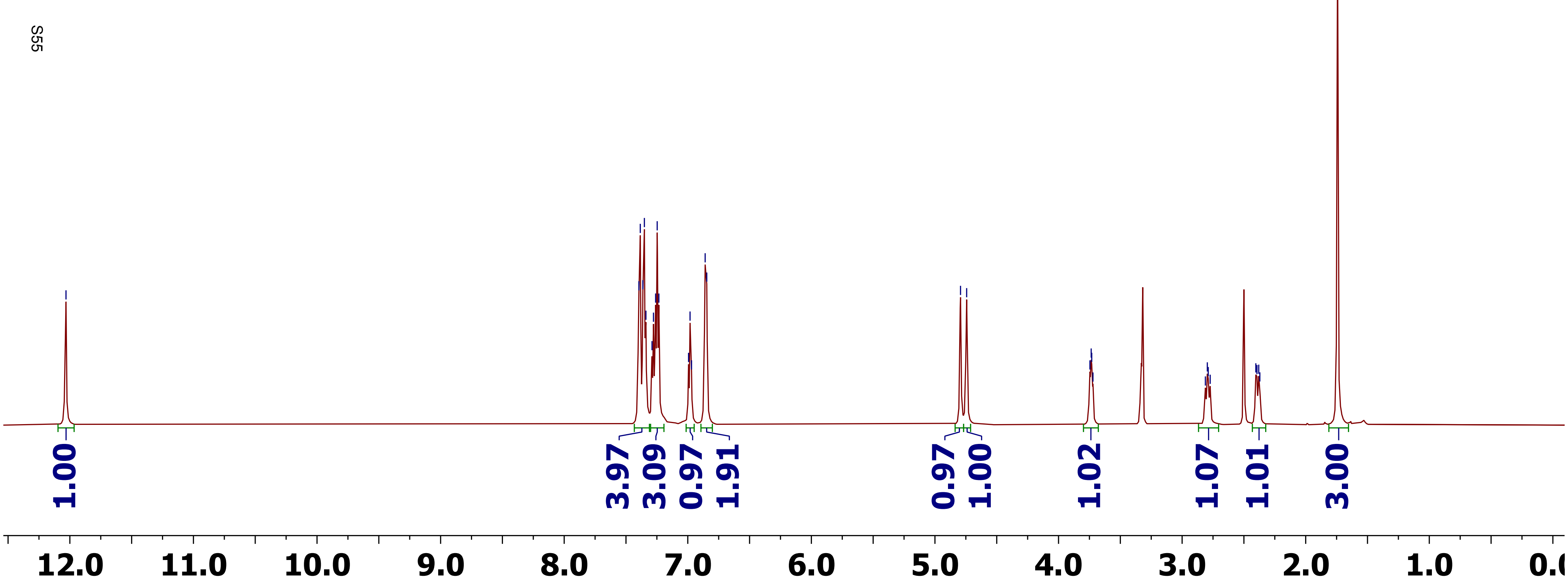

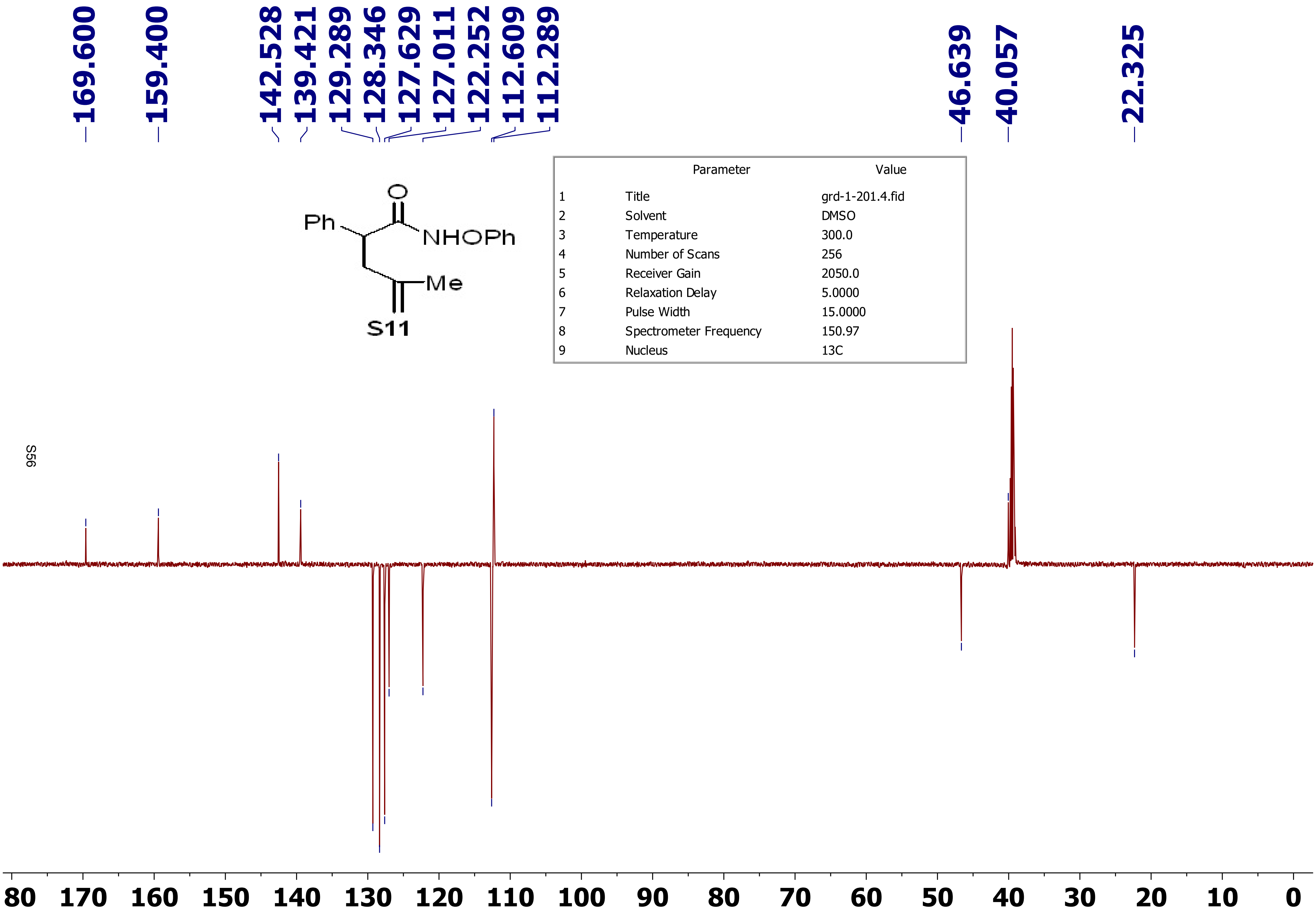

S11

	Parameter	Value
1	Title	grd-1-201.4.fid
2	Solvent	DMSO
3	Temperature	300.0
4	Number of Scans	256
5	Receiver Gain	2050.0
6	Relaxation Delay	5.0000
7	Pulse Width	15.0000
8	Spectrometer Frequency	150.97
9	Nucleus	13C

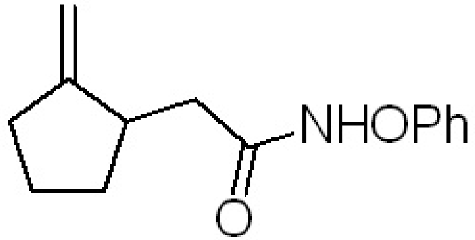

S12

	Parameter	Value
1	Title	grd-2-119.1.fid
2	Solvent	DMSO
3	Temperature	300.0
4	Number of Scans	16
5	Receiver Gain	90.5
6	Relaxation Delay	1.0000
7	Pulse Width	10.6100
8	Spectrometer Frequency	600.32
9	Nucleus	1H

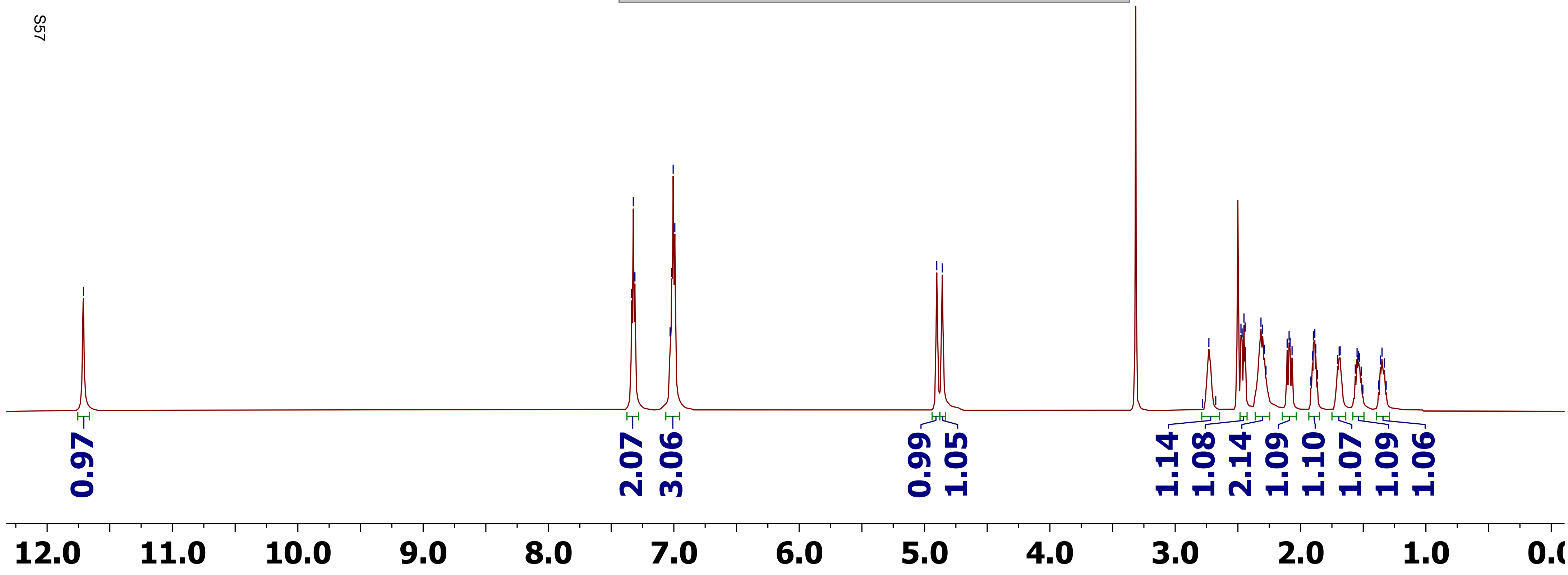

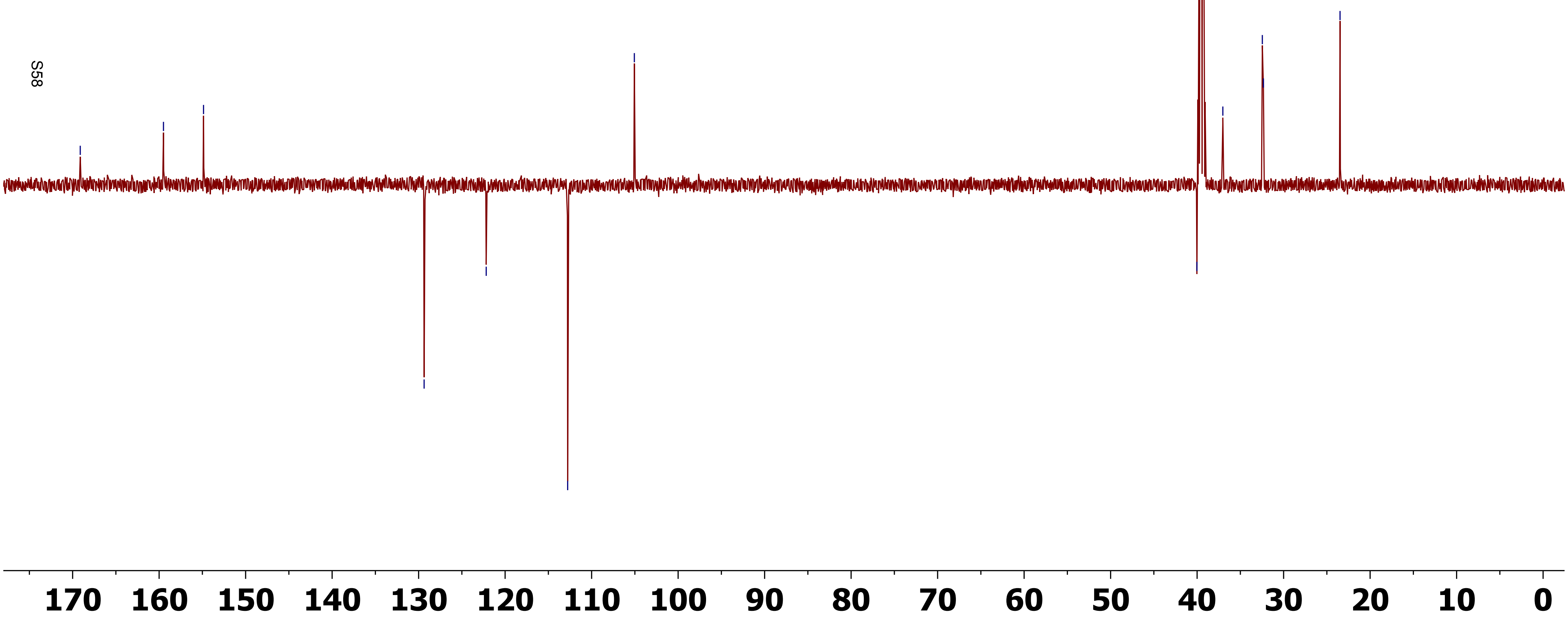

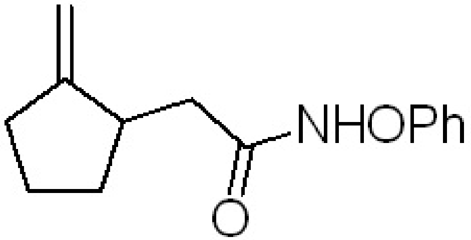

S12

	Parameter	Value
1	Title	grd-2-119.2.fid
2	Solvent	DMSO
3	Temperature	300.0
4	Number of Scans	256
5	Receiver Gain	2050.0
6	Relaxation Delay	5.0000
7	Pulse Width	15.0000
8	Spectrometer Frequency	150.97
9	Nucleus	13C

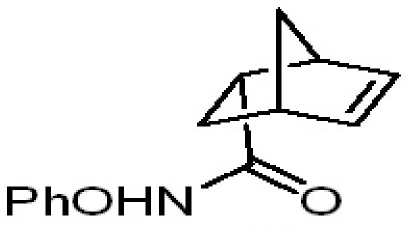

35

	Parameter	Value
1	Title	grd-1-278.3.fid
2	Solvent	DMSO
3	Temperature	300.0
4	Number of Scans	16
5	Receiver Gain	57.0
6	Relaxation Delay	1.0000
7	Pulse Width	10.6100
8	Spectrometer Frequency	600.32
9	Nucleus	1H

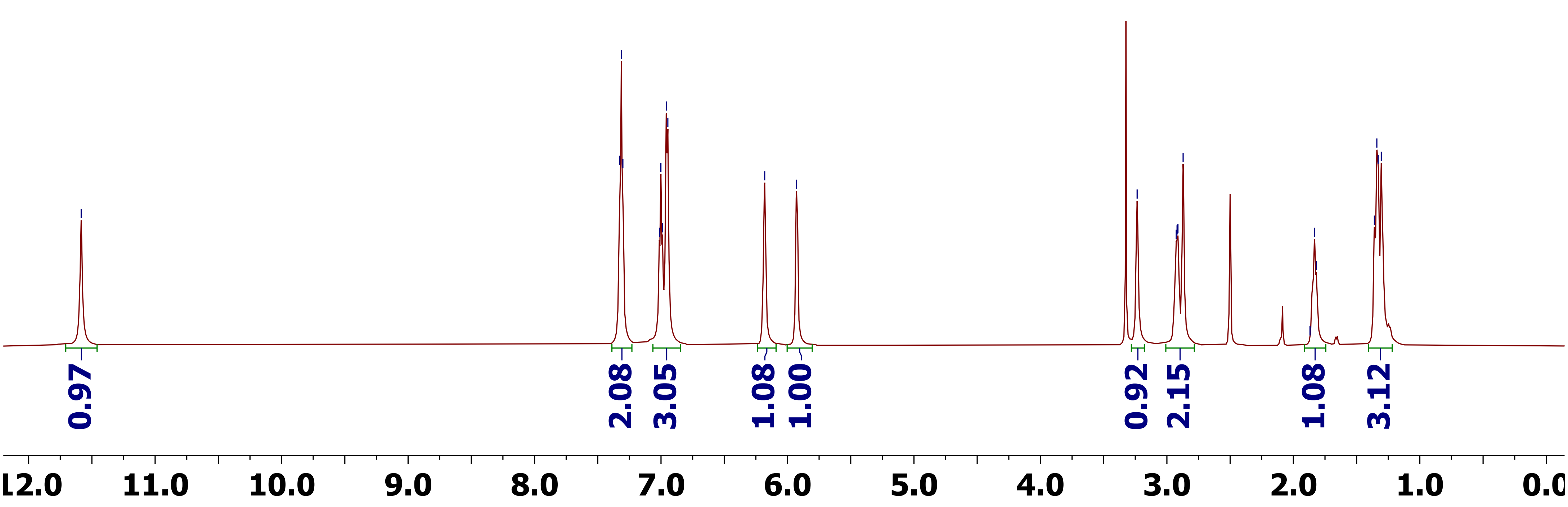

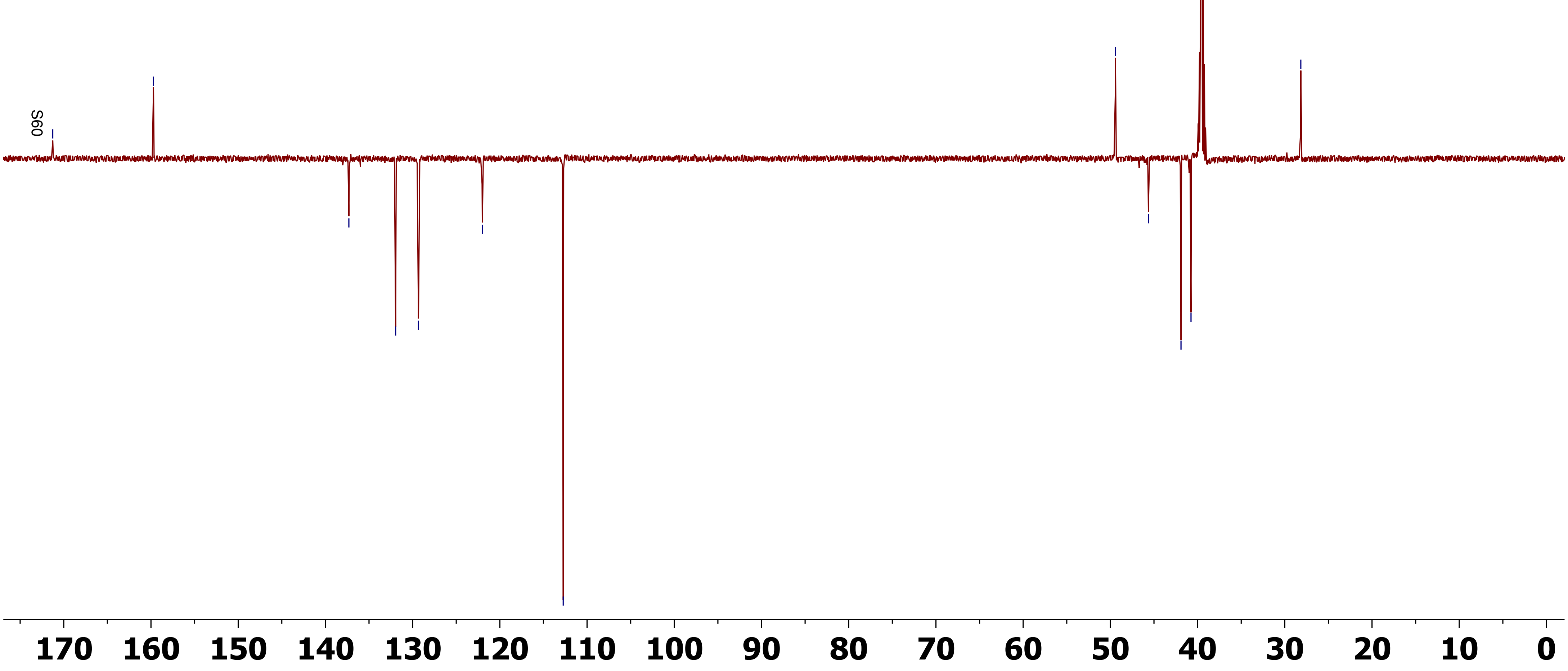

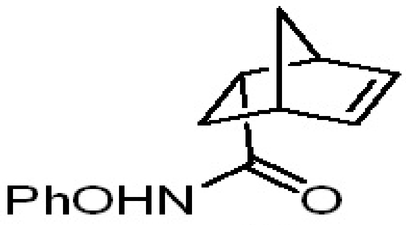

35

	Parameter	Value
1	Title	grd-1-278.5.fid
2	Solvent	DMSO
3	Temperature	300.0
4	Number of Scans	256
5	Receiver Gain	2050.0
6	Relaxation Delay	5.0000
7	Pulse Width	15.0000
8	Spectrometer Frequency	150.97
9	Nucleus	13C

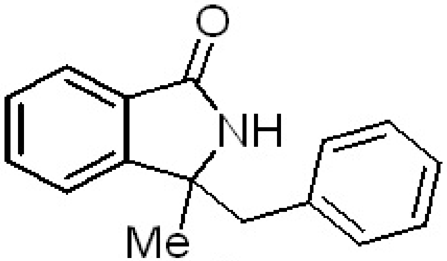

3

	Parameter	Value
1	Title	grd-1-142.1.fid
2	Solvent	CDCl3
3	Temperature	298.0
4	Number of Scans	16
5	Receiver Gain	101.0
6	Relaxation Delay	1.0000
7	Pulse Width	10.5000
8	Spectrometer Frequency	600.32
9	Nucleus	1H

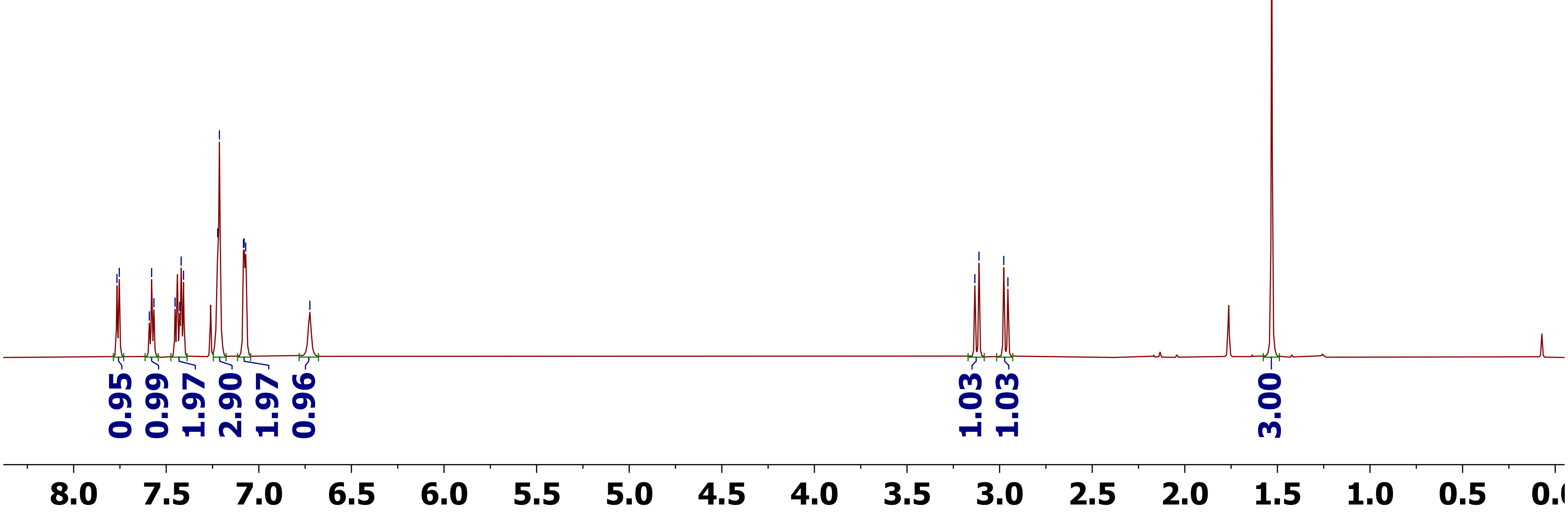

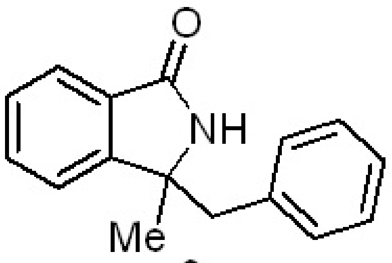

3

	Parameter	Value
1	Title	grd-1-142.2.fid
2	Solvent	CDCl3
3	Temperature	298.0
4	Number of Scans	256
5	Receiver Gain	2050.0
6	Relaxation Delay	5.0000
7	Pulse Width	10.6300
8	Spectrometer Frequency	150.97
9	Nucleus	13C

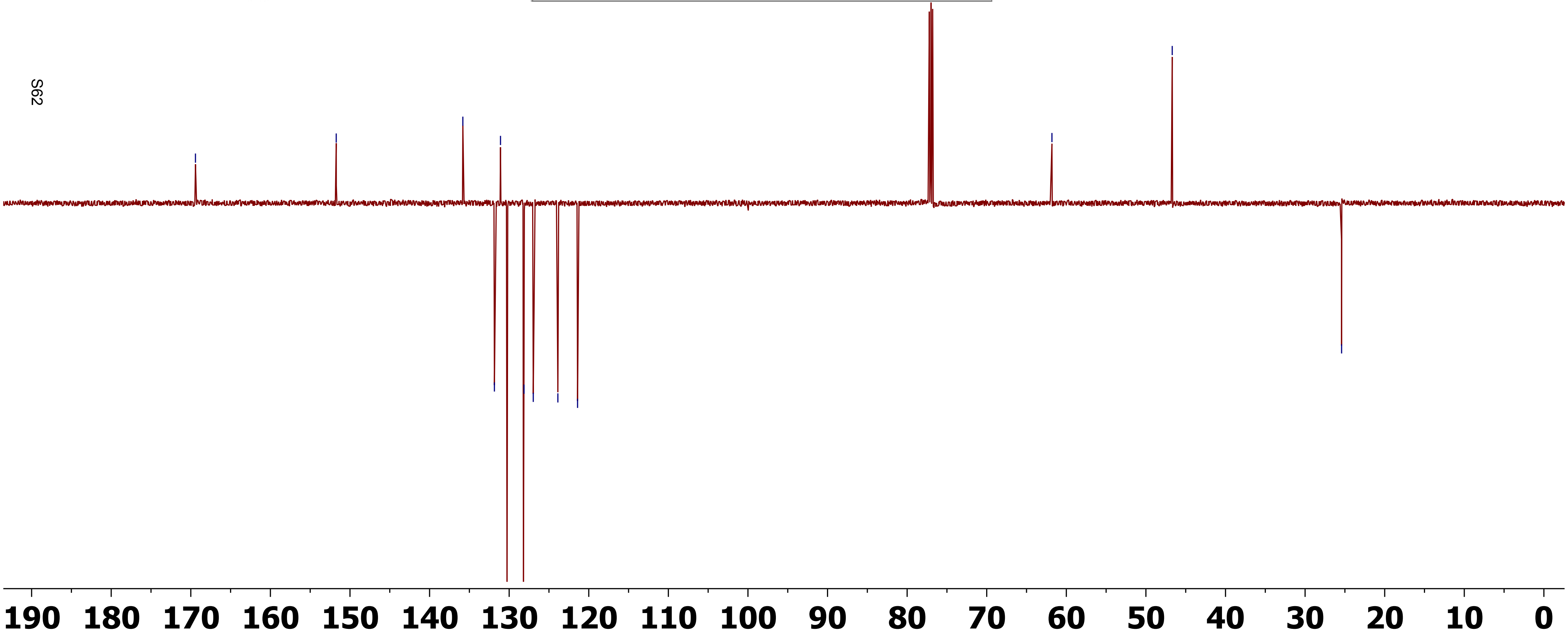

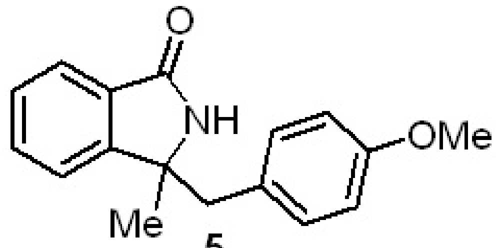

5

	Parameter	Value
1	Title	grd-1-154.1.1
2	Solvent	CDCl3
3	Temperature	298.0
4	Number of Scans	16
5	Receiver Gain	16.0
6	Relaxation Delay	1.0000
7	Pulse Width	10.5000
8	Spectrometer Frequency	600.32
9	Nucleus	1H

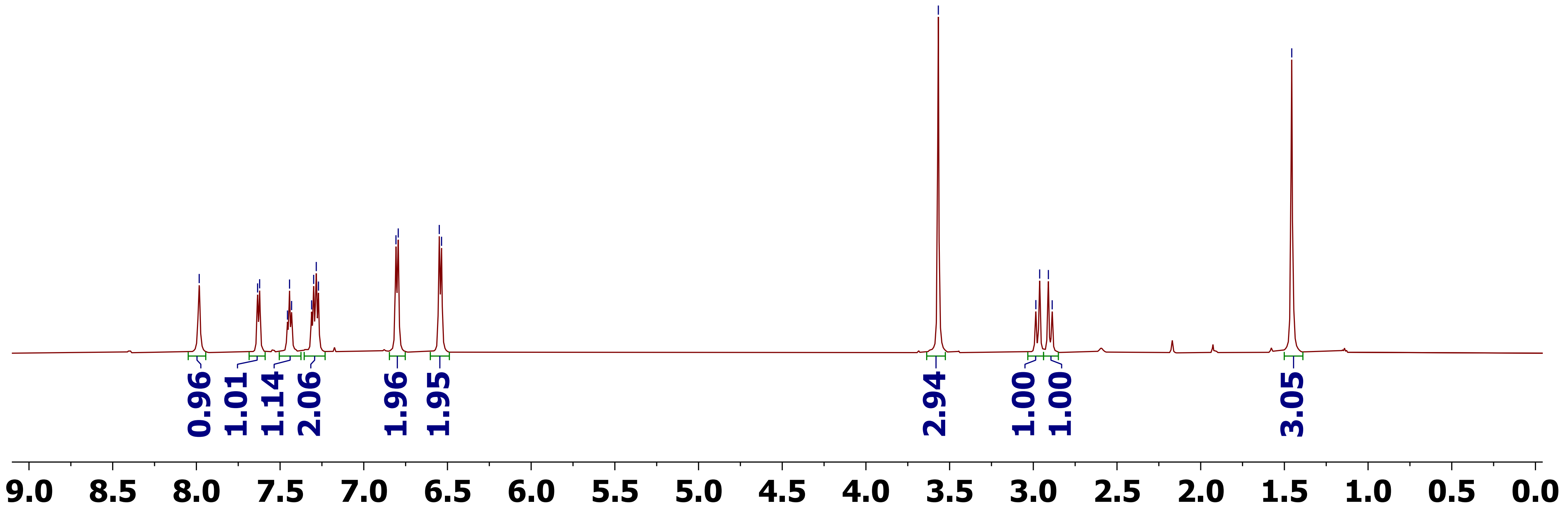

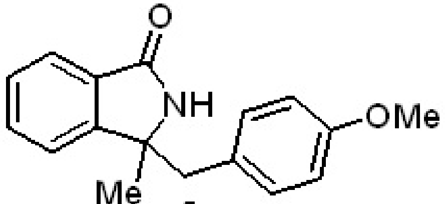

5

	Parameter	Value
1	Title	grd-1-154.2.1
2	Solvent	CDCl3
3	Temperature	298.0
4	Number of Scans	256
5	Receiver Gain	2050.0
6	Relaxation Delay	5.0000
7	Pulse Width	10.6300
8	Spectrometer Frequency	150.95
9	Nucleus	13C

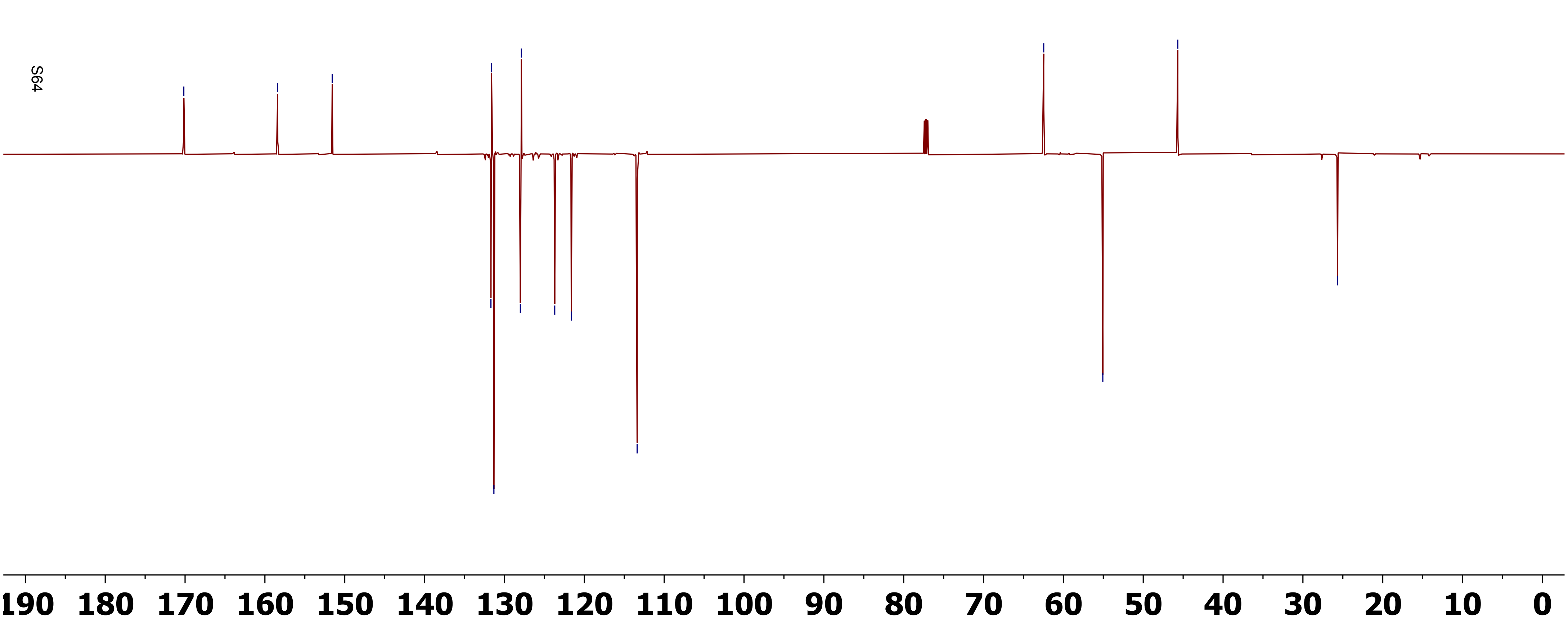

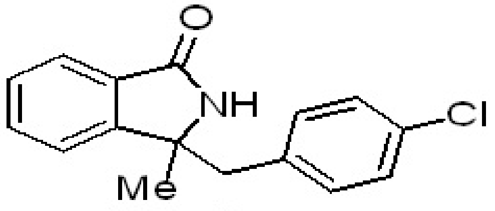

6

	Parameter	Value
1	Title	grd-1-158.2.fid
2	Solvent	CDCl3
3	Temperature	298.0
4	Number of Scans	16
5	Receiver Gain	40.3
6	Relaxation Delay	1.0000
7	Pulse Width	10.5000
8	Spectrometer Frequency	600.32
9	Nucleus	1H

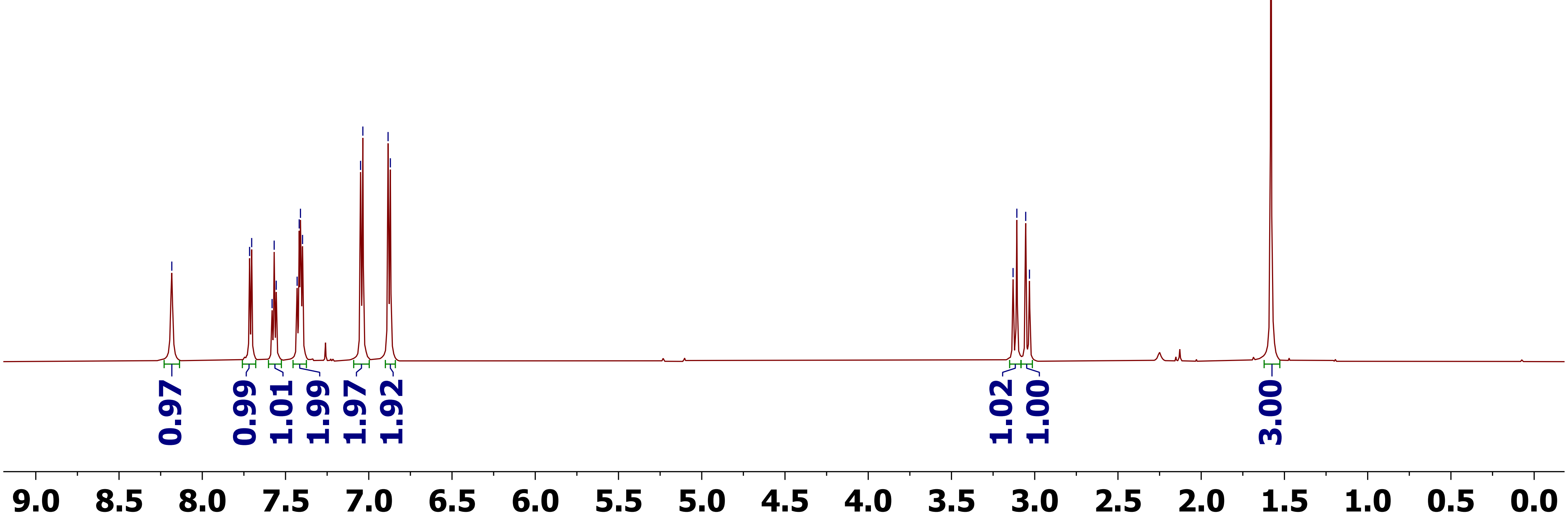

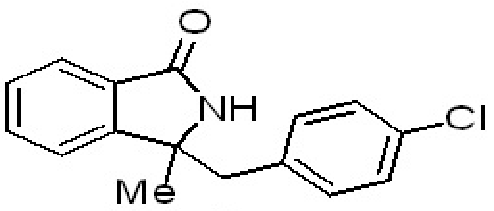

6

	Parameter	Value
1	Title	grd-1-158.3.fid
2	Solvent	CDCl3
3	Temperature	298.0
4	Number of Scans	256
5	Receiver Gain	2050.0
6	Relaxation Delay	5.0000
7	Pulse Width	10.6300
8	Spectrometer Frequency	150.97
9	Nucleus	13C

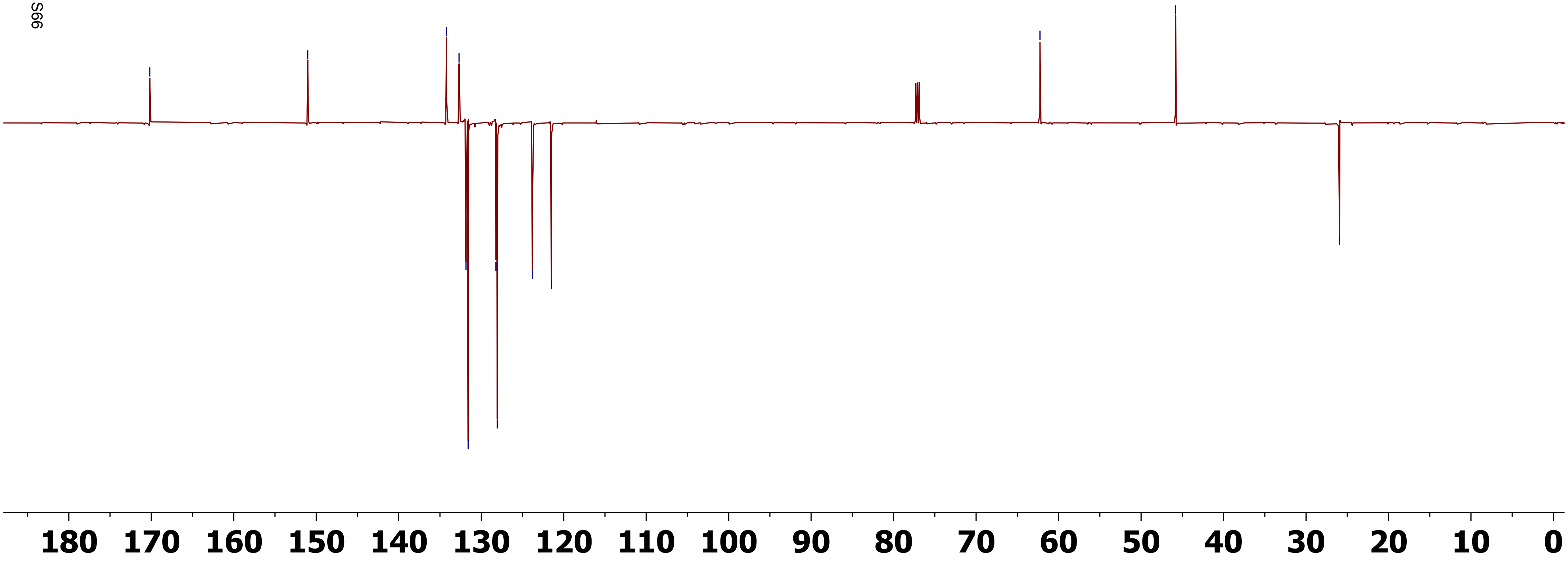

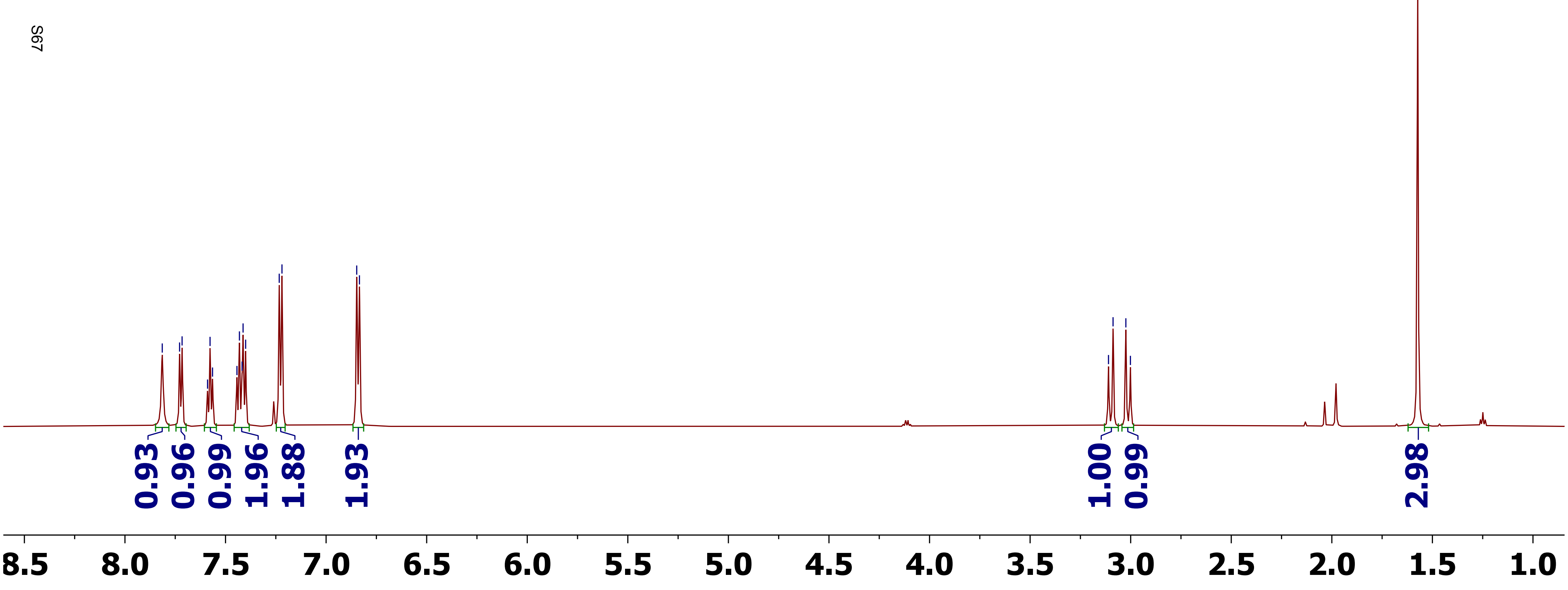

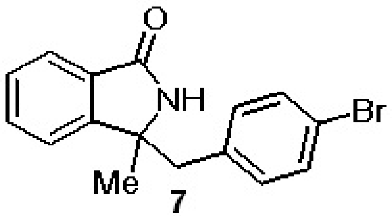

7

	Parameter	Value
1	Title	grd-1-145.1.fid
2	Solvent	CDCl3
3	Temperature	298.0
4	Number of Scans	16
5	Receiver Gain	57.0
6	Relaxation Delay	1.0000
7	Pulse Width	10.5000
8	Spectrometer Frequency	600.32
9	Nucleus	1H

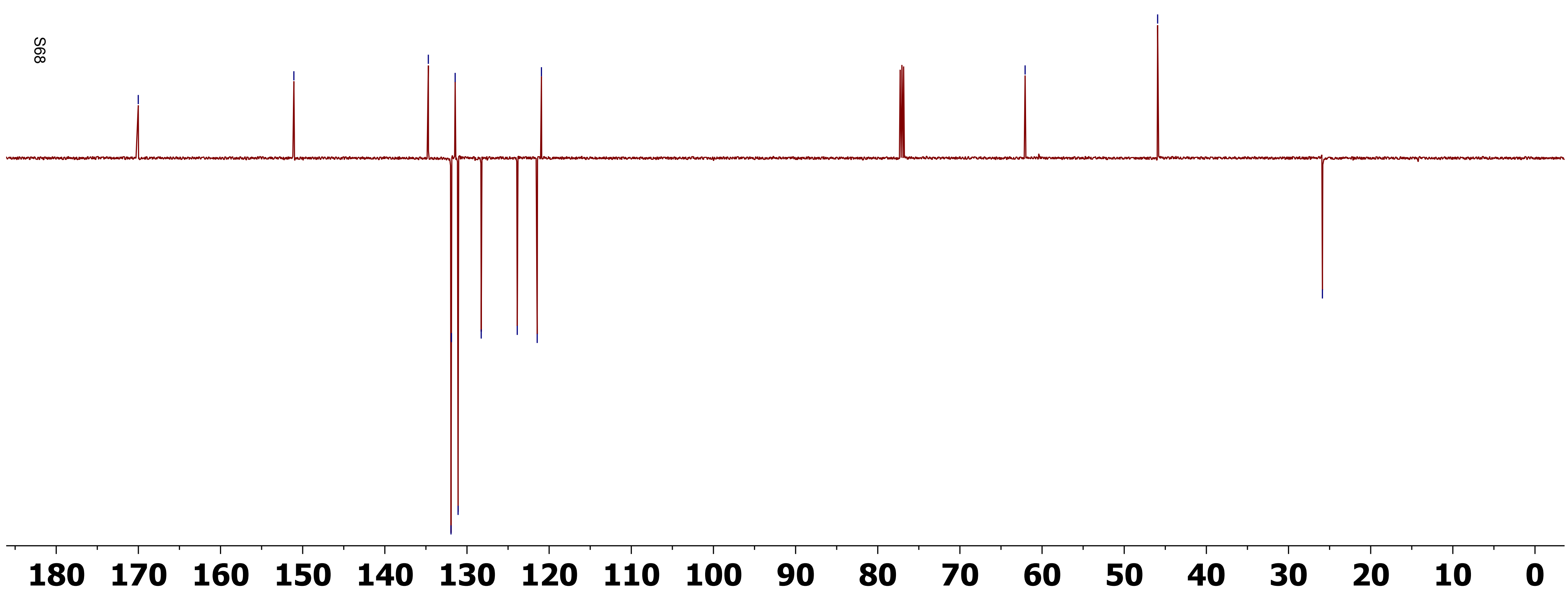

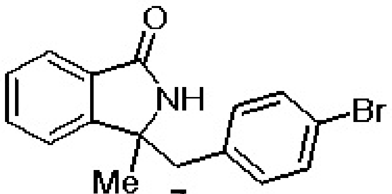

7

	Parameter	Value
1	Title	grd-1-145.2.fid
2	Solvent	CDCl3
3	Temperature	298.0
4	Number of Scans	256
5	Receiver Gain	2050.0
6	Relaxation Delay	5.0000
7	Pulse Width	10.6300
8	Spectrometer Frequency	150.97
9	Nucleus	13C

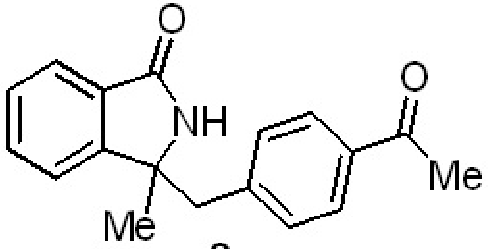

8

	Parameter	Value
1	Title	grd-2-134.1.1
2	Solvent	CDCl3
3	Temperature	298.2
4	Number of Scans	16
5	Receiver Gain	8.0
6	Relaxation Delay	1.0000
7	Pulse Width	15.0000
8	Spectrometer Frequency	400.13
9	Nucleus	1H

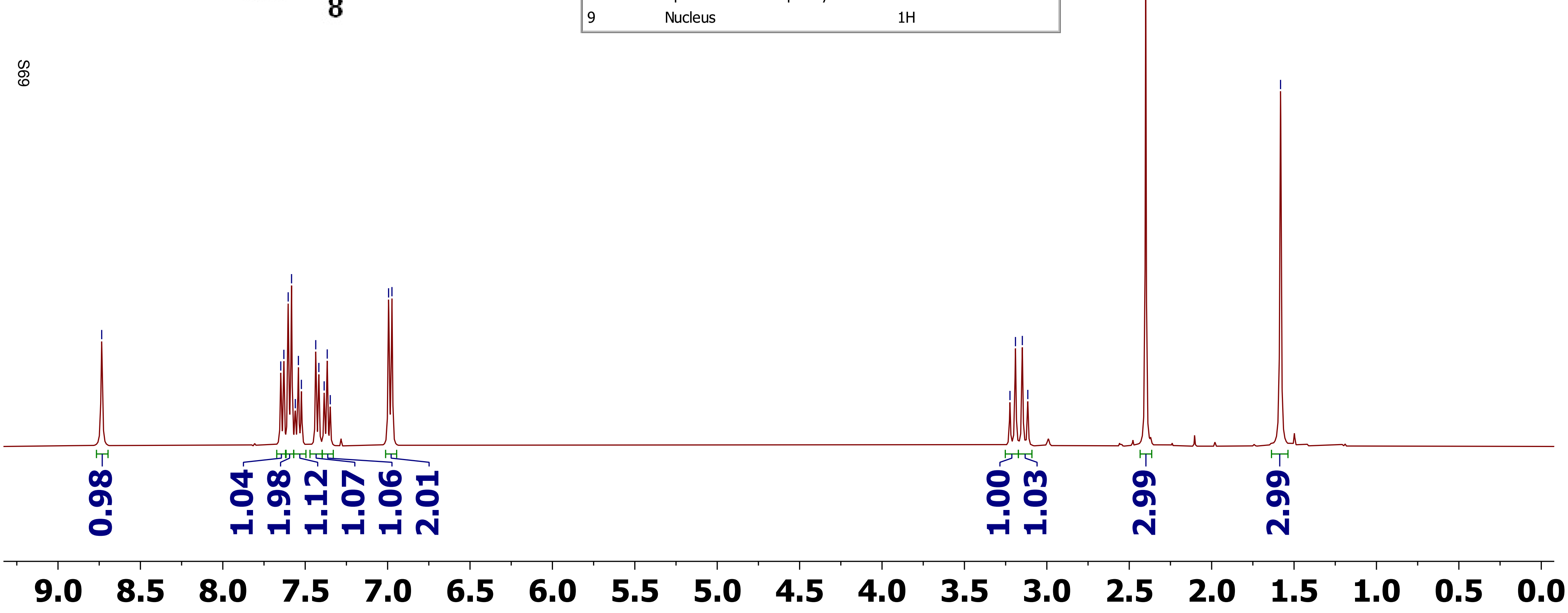

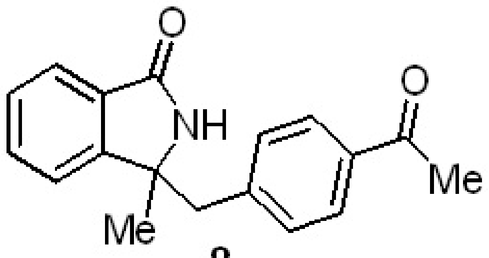

8

	Parameter	Value
1	Title	grd-2-134.2.1
2	Solvent	CDCl3
3	Temperature	298.2
4	Number of Scans	256
5	Receiver Gain	512.0
6	Relaxation Delay	3.0000
7	Pulse Width	10.7000
8	Spectrometer Frequency	100.61
9	Nucleus	13C

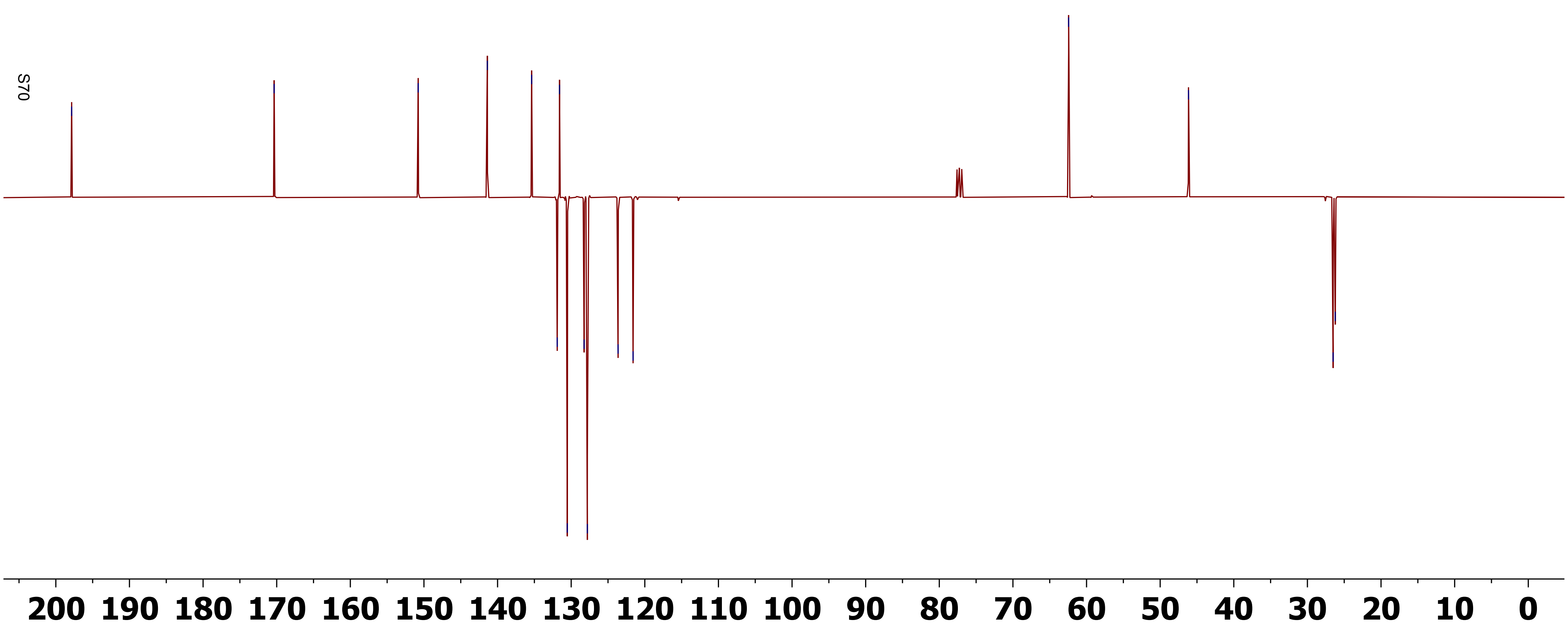

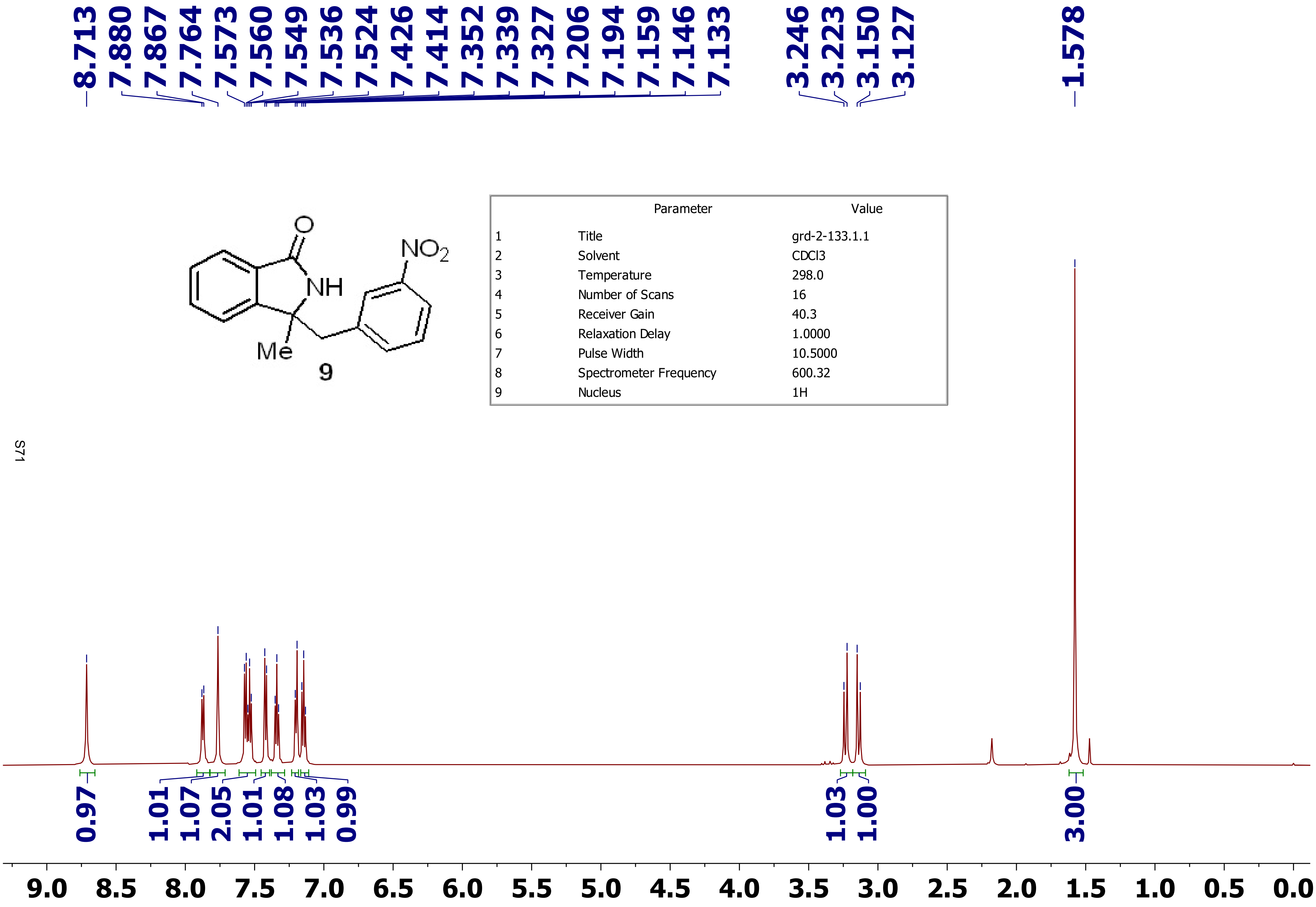

9

	Parameter	Value
1	Title	grd-2-133.1.1
2	Solvent	CDCl3
3	Temperature	298.0
4	Number of Scans	16
5	Receiver Gain	40.3
6	Relaxation Delay	1.0000
7	Pulse Width	10.5000
8	Spectrometer Frequency	600.32
9	Nucleus	1H

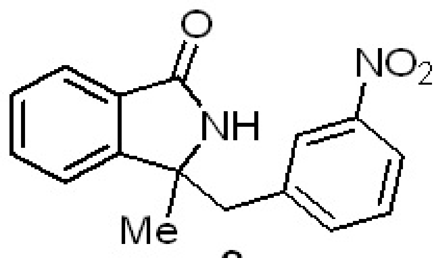

	Parameter	Value
1	Title	grd-2-133.2.1
2	Solvent	CDCl3
3	Temperature	298.0
4	Number of Scans	256
5	Receiver Gain	2050.0
6	Relaxation Delay	5.0000
7	Pulse Width	10.6300
8	Spectrometer Frequency	150.95
9	Nucleus	13C

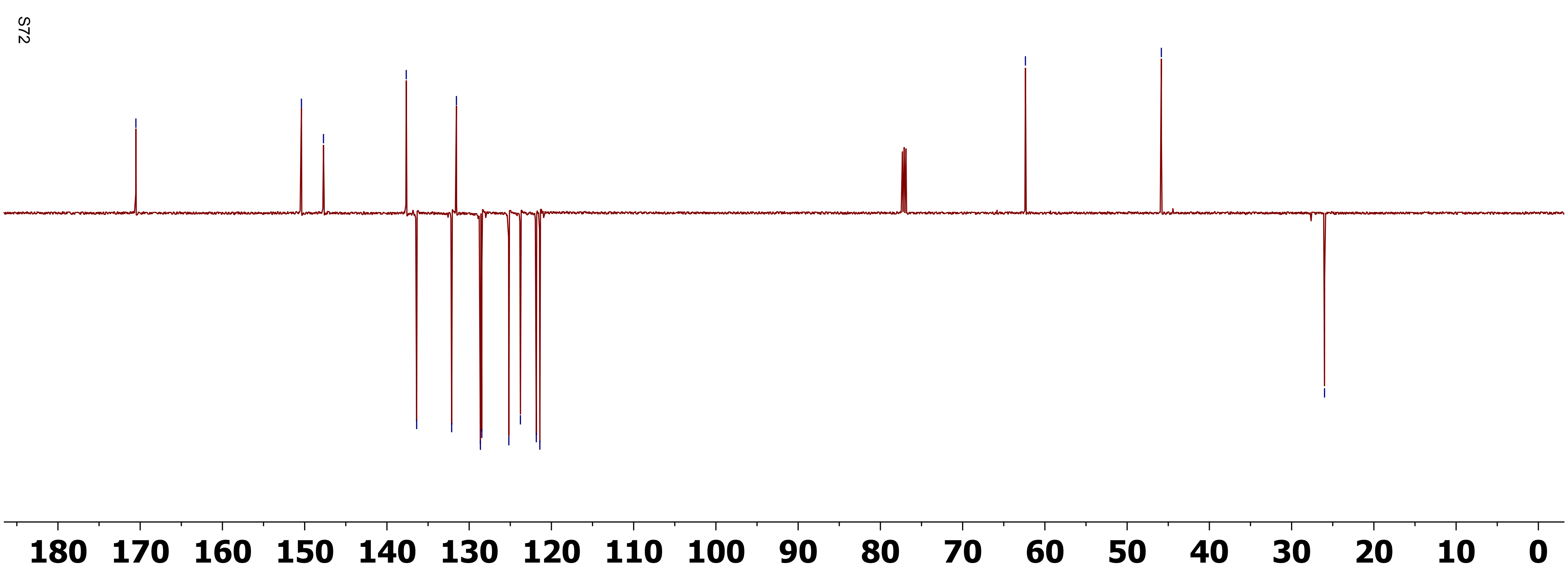

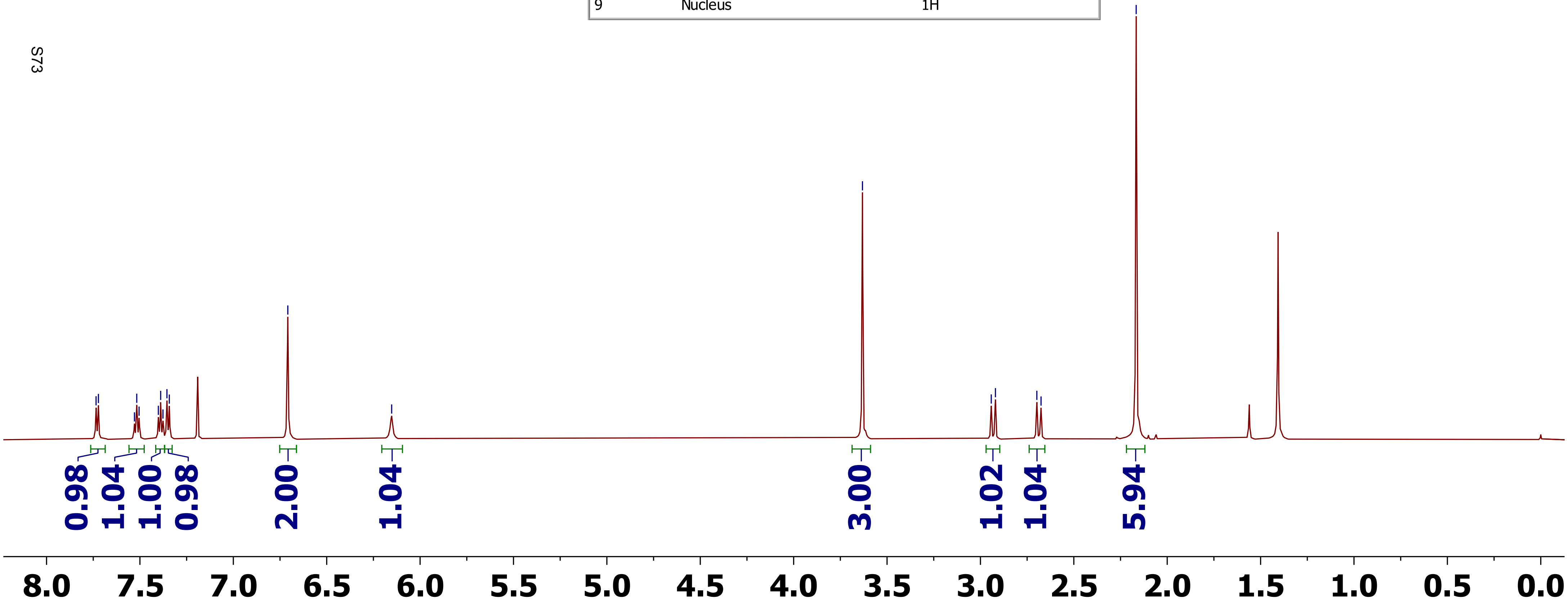

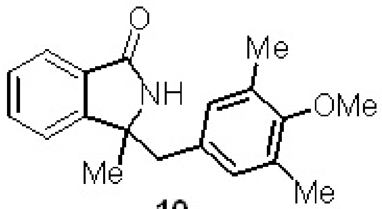

10

	Parameter	Value
1	Title	grd-1-176.8.fid
2	Solvent	CDCl3
3	Temperature	298.0
4	Number of Scans	16
5	Receiver Gain	128.0
6	Relaxation Delay	1.0000
7	Pulse Width	10.5000
8	Spectrometer Frequency	600.32
9	Nucleus	1H

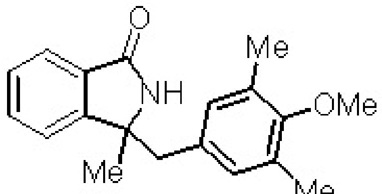

10

	Parameter	Value
1	Title	grd-1-176.7.fid
2	Solvent	CDCl3
3	Temperature	298.0
4	Number of Scans	256
5	Receiver Gain	2050.0
6	Relaxation Delay	5.0000
7	Pulse Width	10.6300
8	Spectrometer Frequency	150.97
9	Nucleus	13C

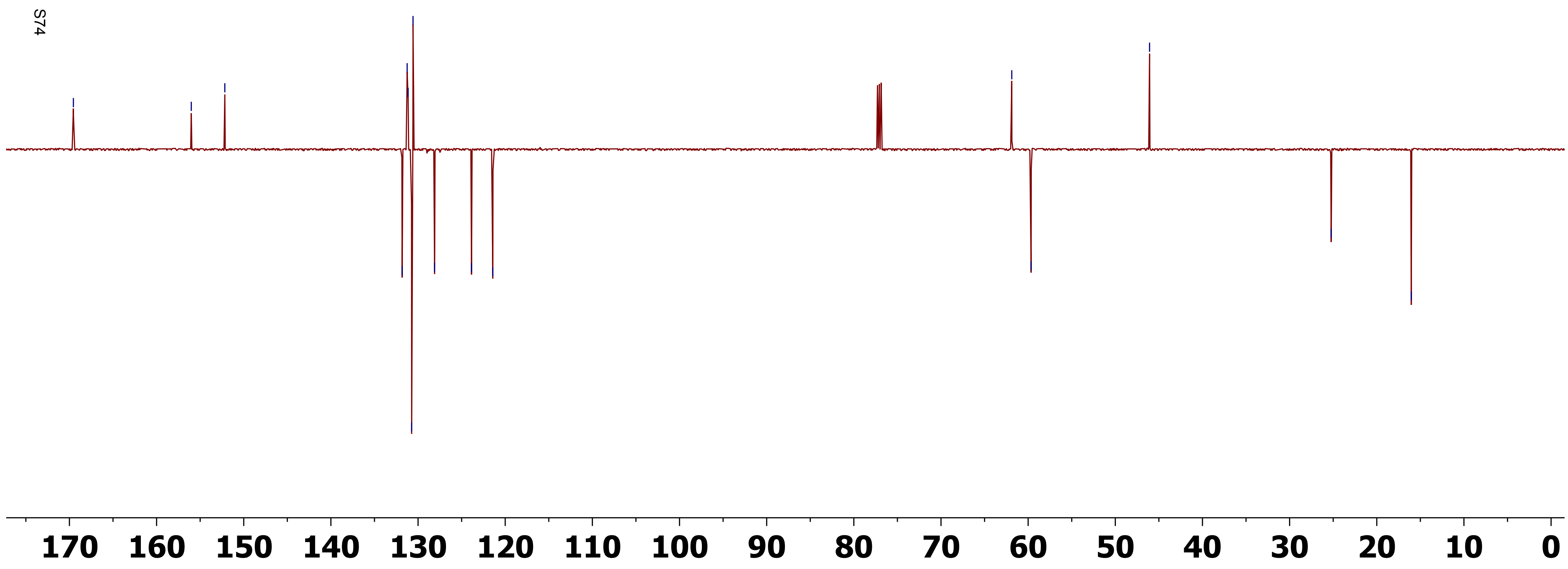

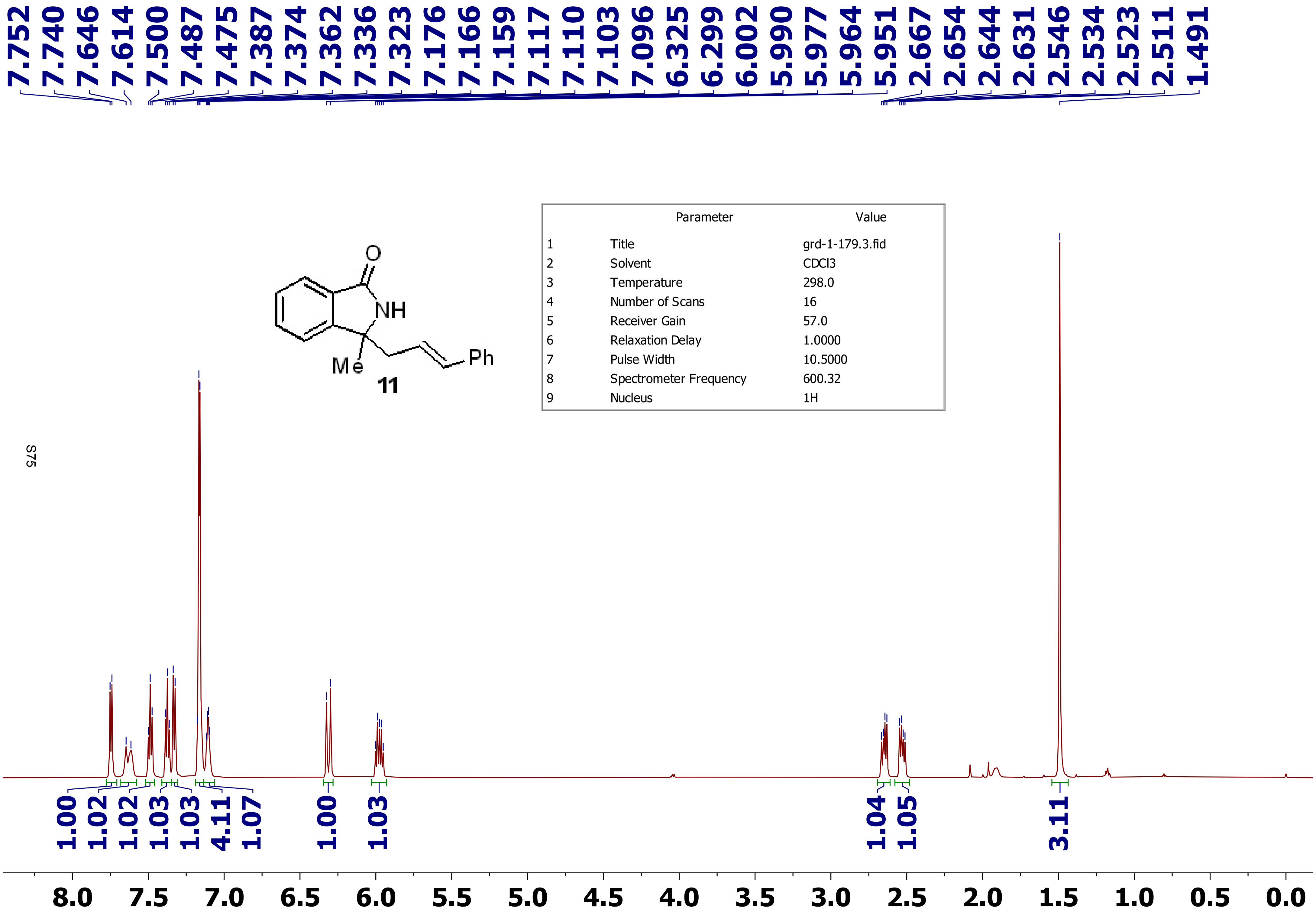

	Parameter	Value
1	Title	grd-1-179.3.fid
2	Solvent	CDCl3
3	Temperature	298.0
4	Number of Scans	16
5	Receiver Gain	57.0
6	Relaxation Delay	1.0000
7	Pulse Width	10.5000
8	Spectrometer Frequency	600.32
9	Nucleus	1H

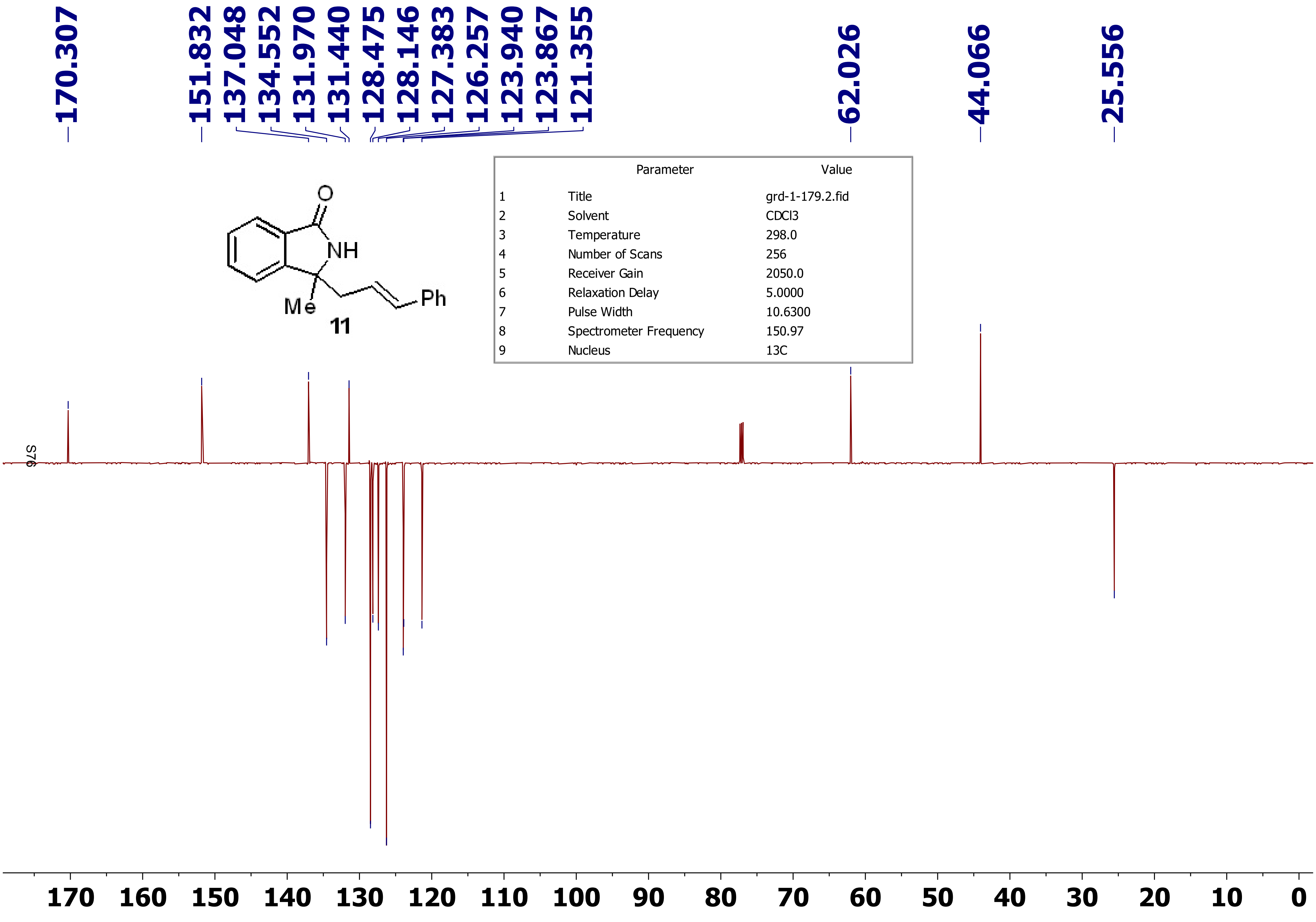

	Parameter	Value
1	Title	grd-1-179.2.fid
2	Solvent	CDCl3
3	Temperature	298.0
4	Number of Scans	256
5	Receiver Gain	2050.0
6	Relaxation Delay	5.0000
7	Pulse Width	10.6300
8	Spectrometer Frequency	150.97
9	Nucleus	13C

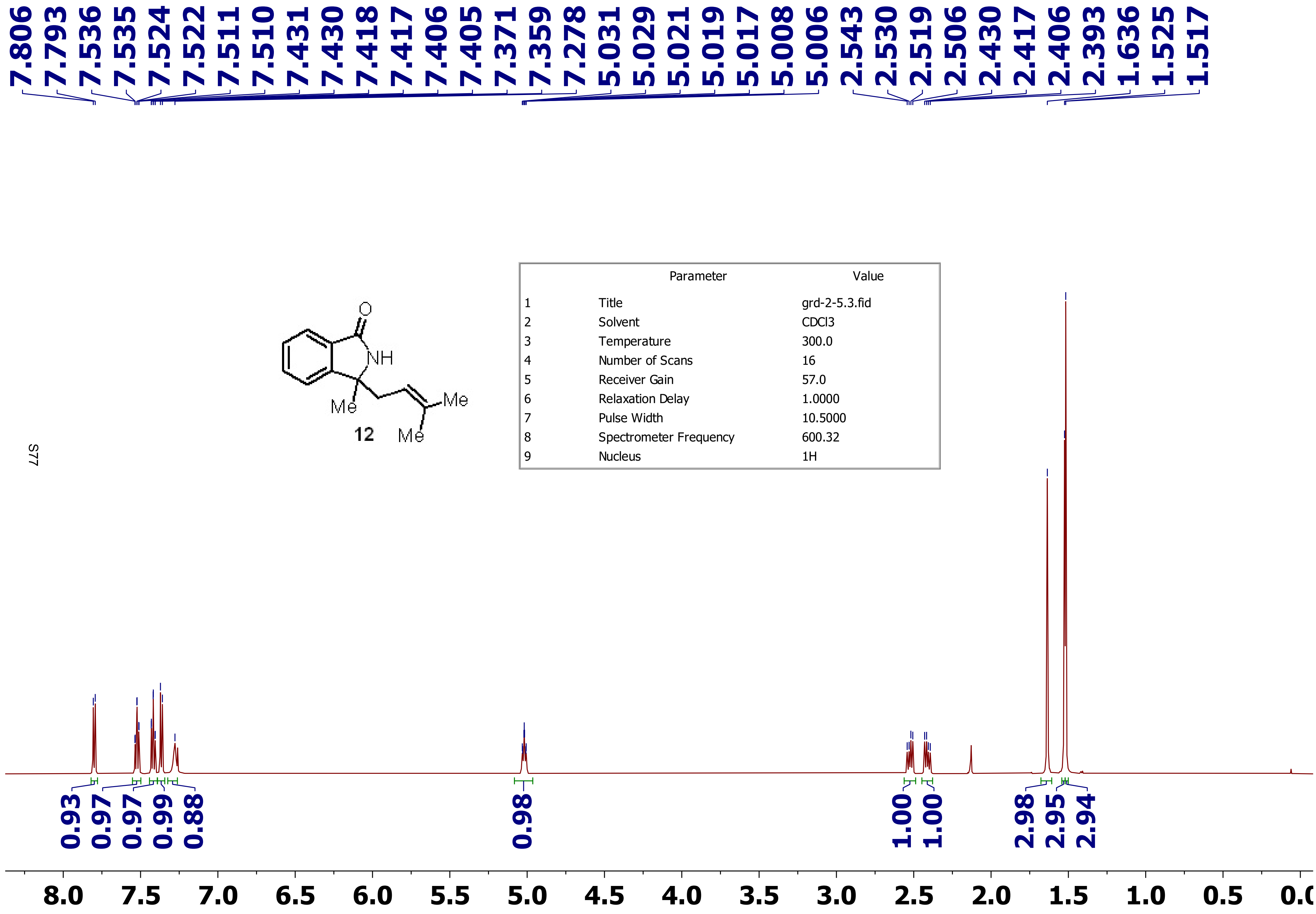

	Parameter	Value
1	Title	grd-2-5.3.fid
2	Solvent	CDCl3
3	Temperature	300.0
4	Number of Scans	16
5	Receiver Gain	57.0
6	Relaxation Delay	1.0000
7	Pulse Width	10.5000
8	Spectrometer Frequency	600.32
9	Nucleus	1H

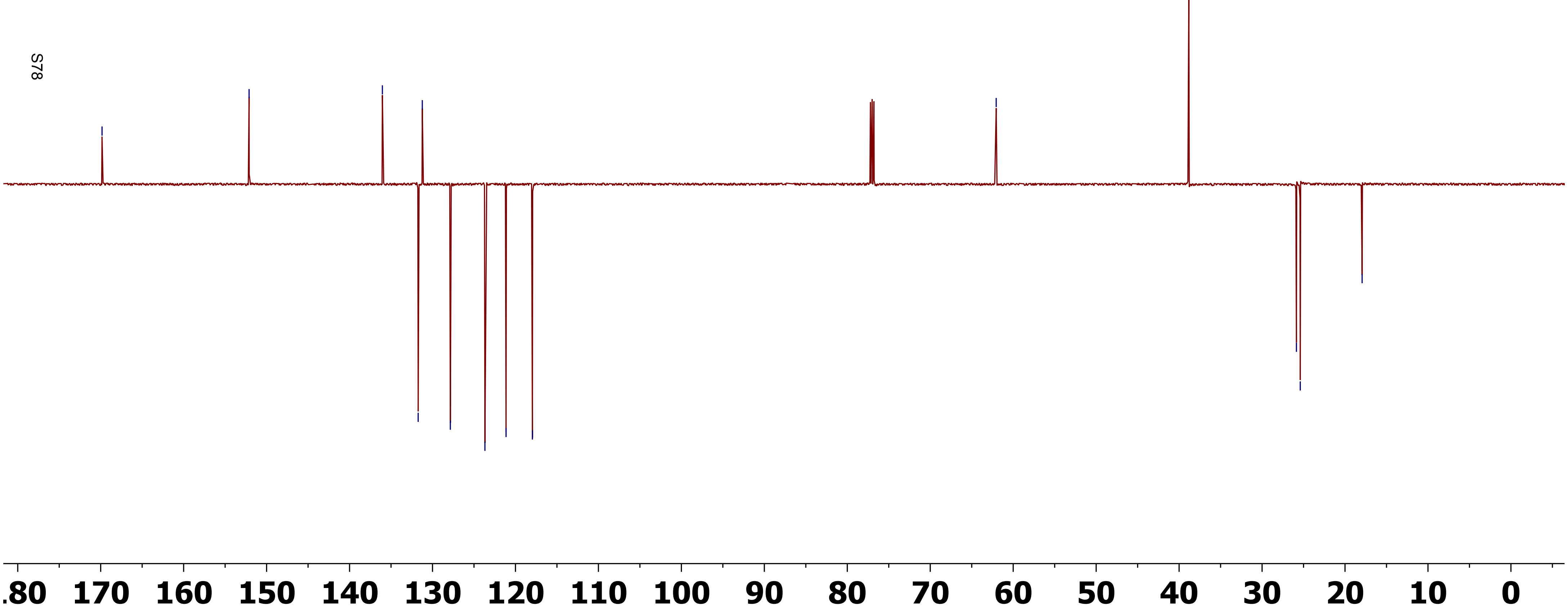

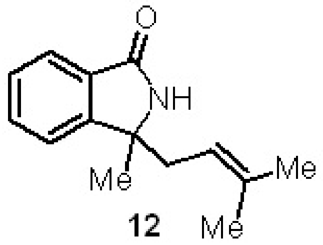

12

	Parameter	Value
1	Title	grd-2-5.4.fid
2	Solvent	CDCl3
3	Temperature	300.0
4	Number of Scans	256
5	Receiver Gain	2050.0
6	Relaxation Delay	5.0000
7	Pulse Width	10.6300
8	Spectrometer Frequency	150.97
9	Nucleus	13C

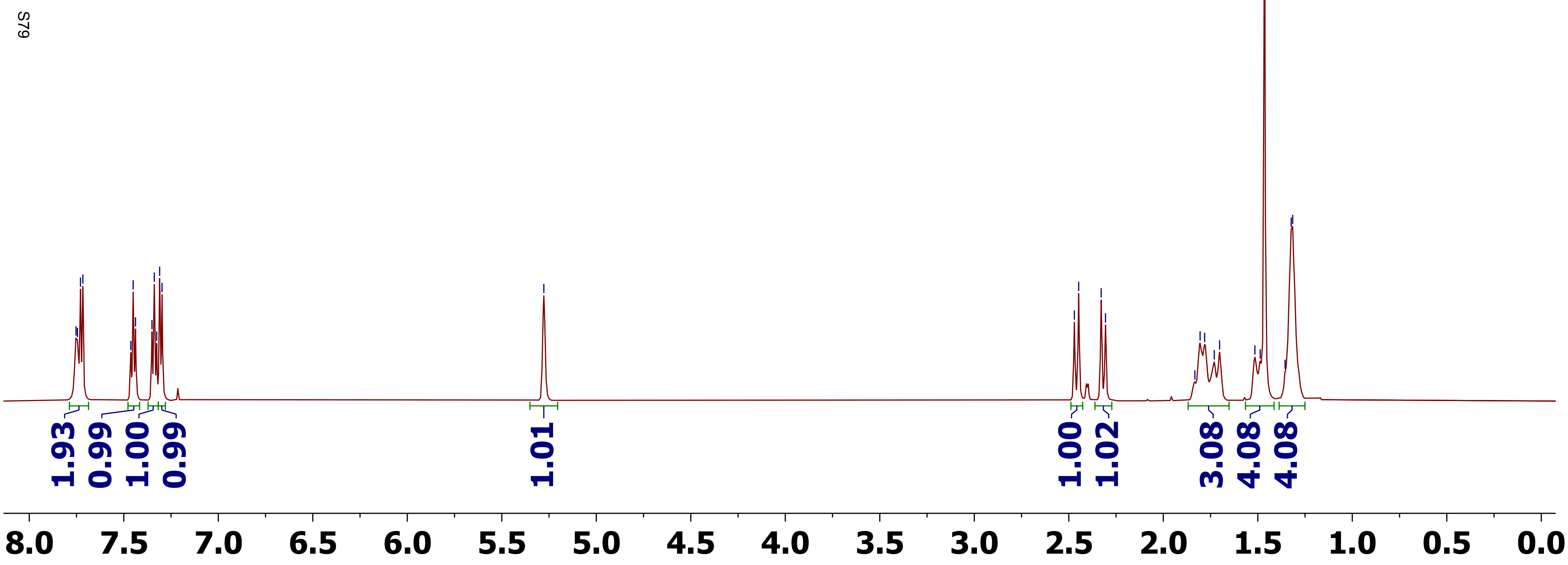

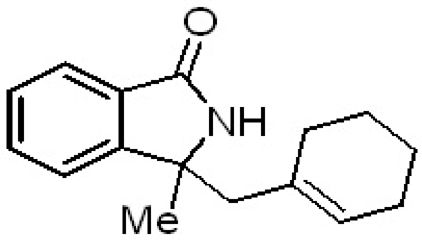

13

	Parameter	Value
1	Title	grd-2-124.3.1
2	Solvent	CDCl3
3	Temperature	298.0
4	Number of Scans	16
5	Receiver Gain	16.0
6	Relaxation Delay	1.0000
7	Pulse Width	10.5000
8	Spectrometer Frequency	600.32
9	Nucleus	1H

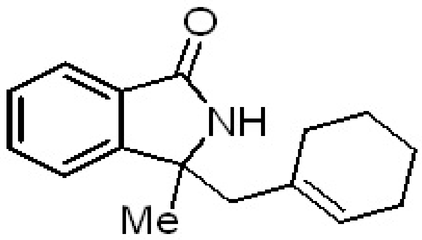

13

	Parameter	Value
1	Title	grd-2-124.4.1
2	Solvent	CDCl3
3	Temperature	298.0
4	Number of Scans	256
5	Receiver Gain	2050.0
6	Relaxation Delay	5.0000
7	Pulse Width	10.6300
8	Spectrometer Frequency	150.95
9	Nucleus	13C

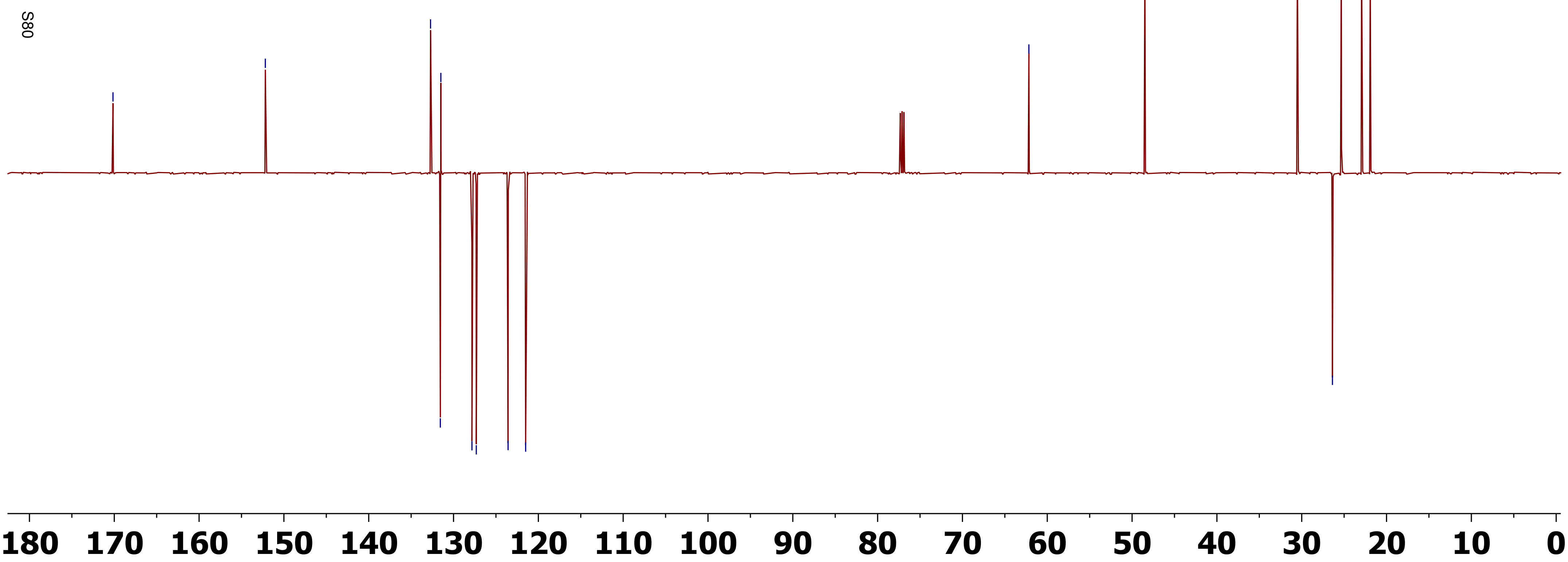

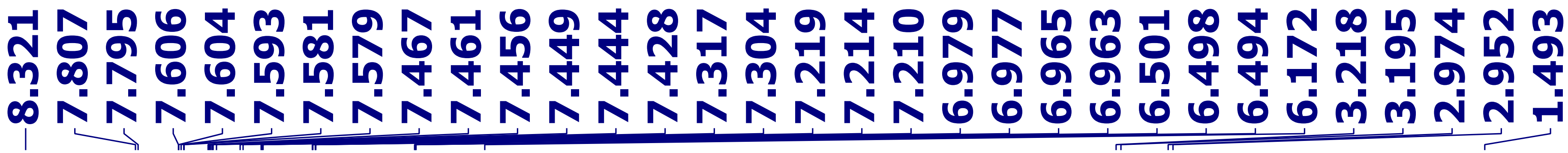

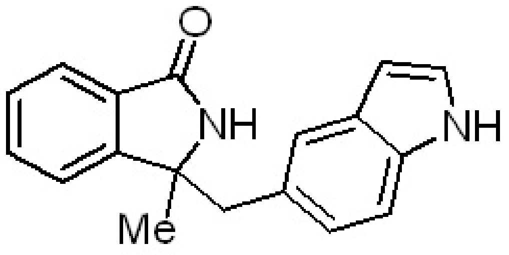

14

	Parameter	Value
1	Title	grd-2-95.2.fid
2	Solvent	CDCl3
3	Temperature	300.0
4	Number of Scans	16
5	Receiver Gain	144.0
6	Relaxation Delay	1.0000
7	Pulse Width	10.5000
8	Spectrometer Frequency	600.32
9	Nucleus	1H

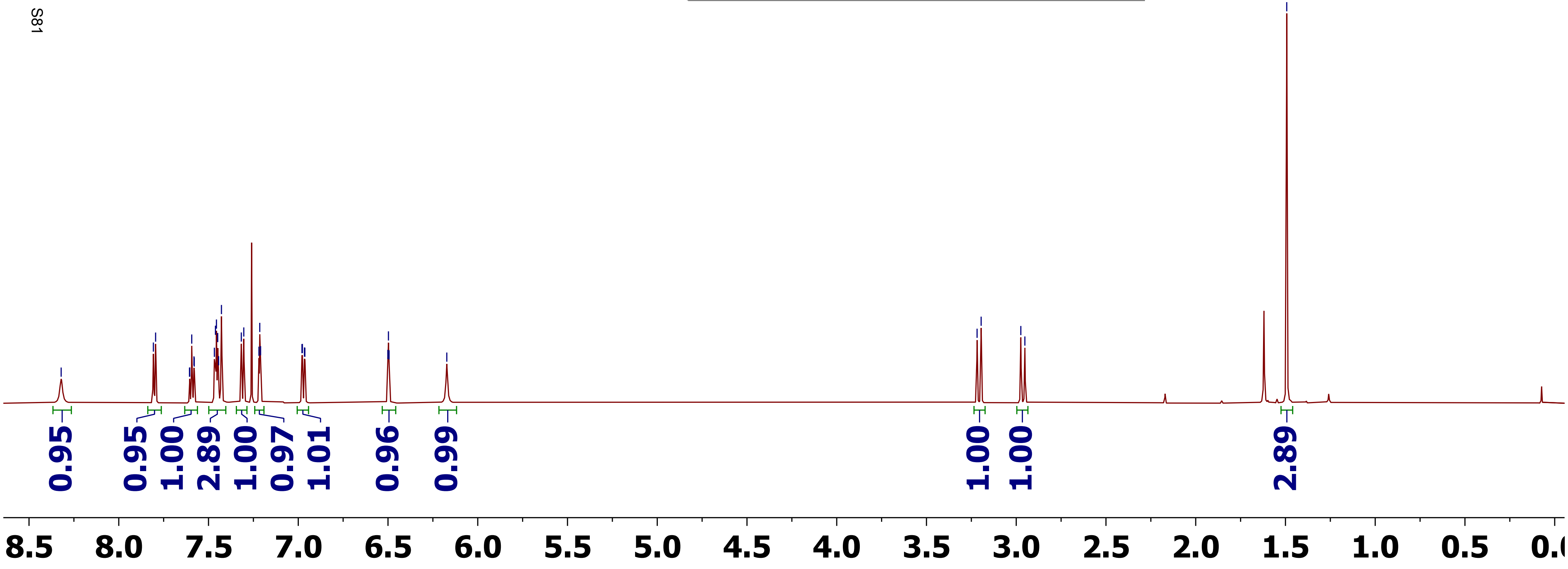

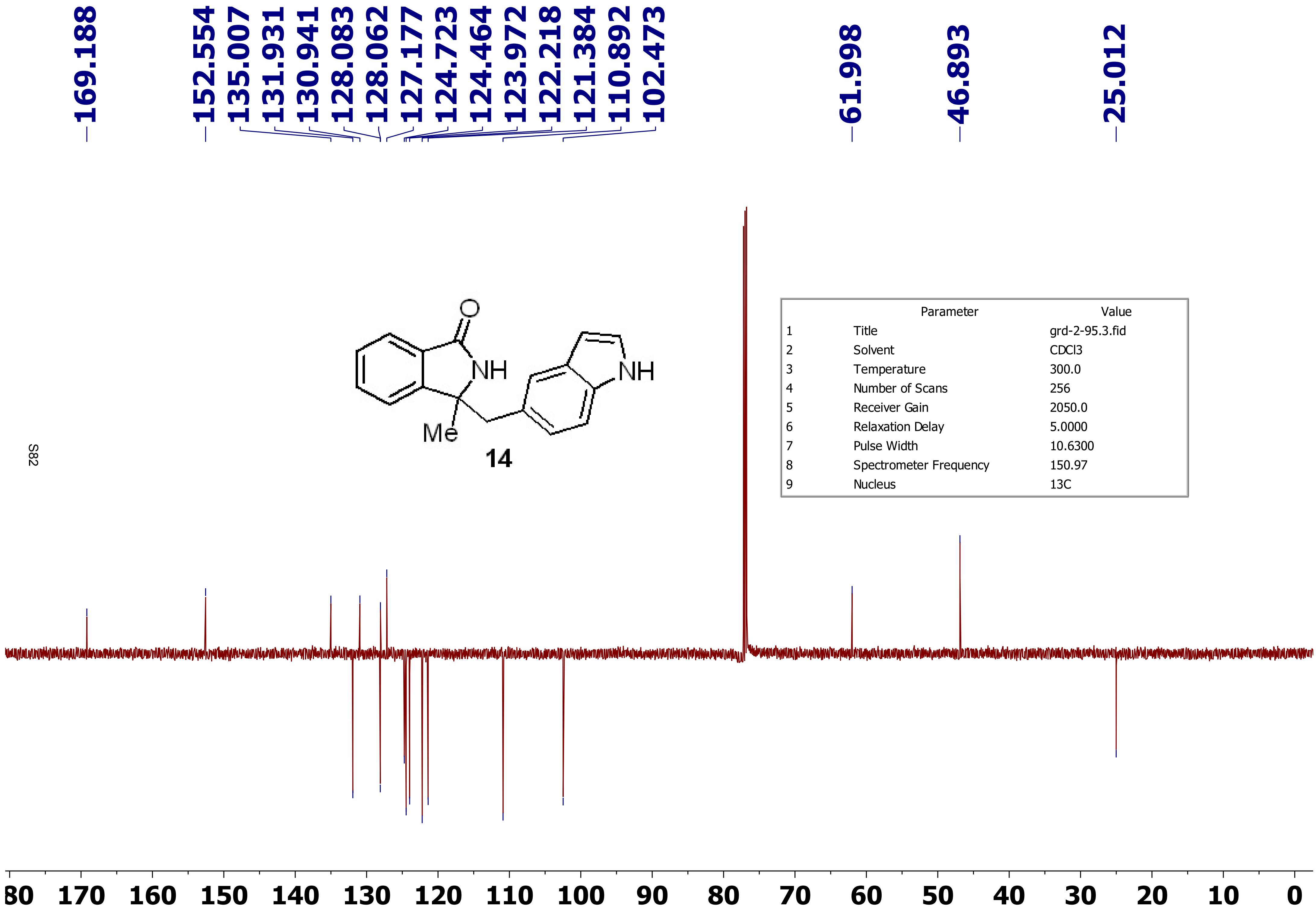

	Parameter	Value
1	Title	grd-2-95.3.fid
2	Solvent	CDCl3
3	Temperature	300.0
4	Number of Scans	256
5	Receiver Gain	2050.0
6	Relaxation Delay	5.0000
7	Pulse Width	10.6300
8	Spectrometer Frequency	150.97
9	Nucleus	13C

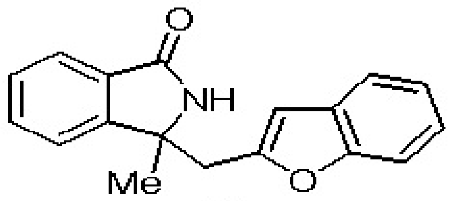

15

	Parameter	Value
1	Title	grd-2-101.5.1
2	Solvent	CDCl3
3	Temperature	298.0
4	Number of Scans	16
5	Receiver Gain	18.0
6	Relaxation Delay	1.0000
7	Pulse Width	10.5000
8	Spectrometer Frequency	600.32
9	Nucleus	1H

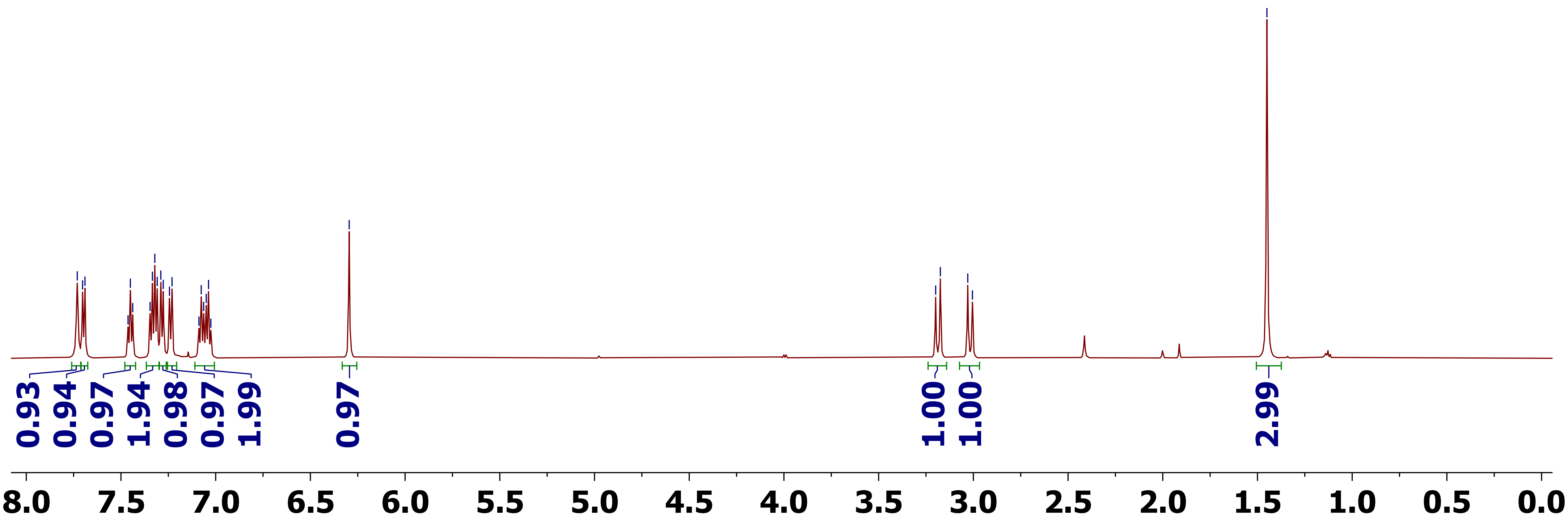

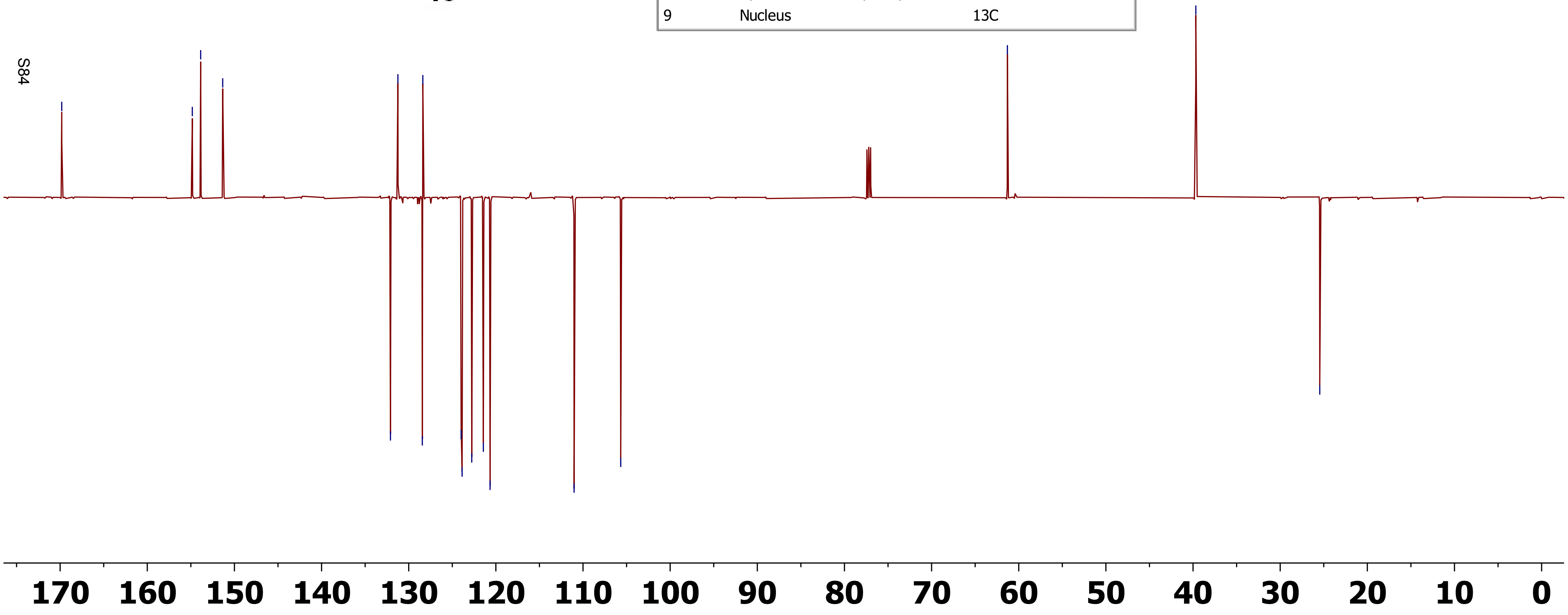

	Parameter	Value
1	Title	grd-2-101.6.1
2	Solvent	CDCl3
3	Temperature	298.0
4	Number of Scans	256
5	Receiver Gain	2050.0
6	Relaxation Delay	5.0000
7	Pulse Width	10.6300
8	Spectrometer Frequency	150.95
9	Nucleus	13C

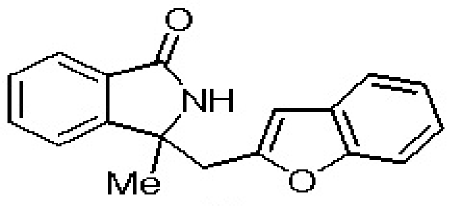

15

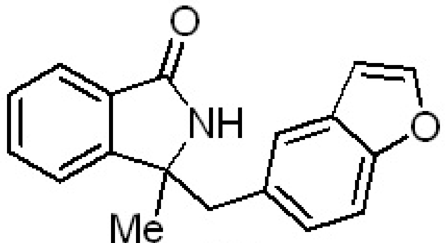

16

	Parameter	Value
1	Title	grd-2-102.3.1
2	Solvent	CDCl3
3	Temperature	298.0
4	Number of Scans	16
5	Receiver Gain	57.0
6	Relaxation Delay	1.0000
7	Pulse Width	10.5000
8	Spectrometer Frequency	600.32
9	Nucleus	1H

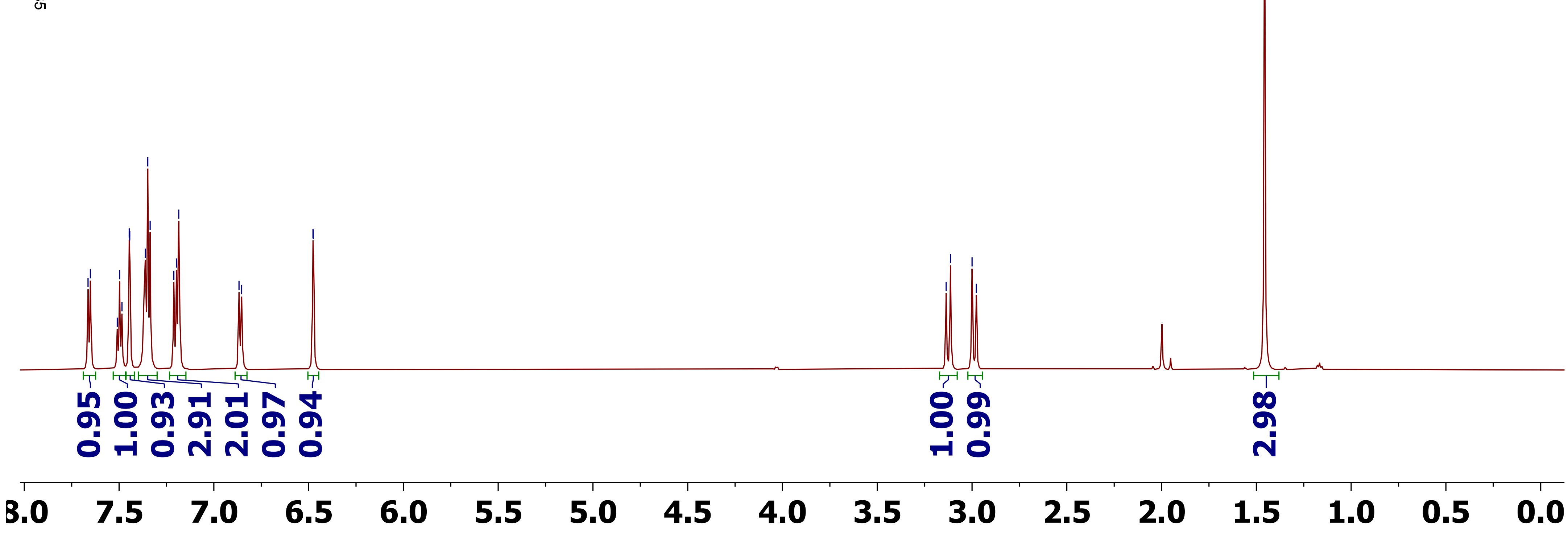

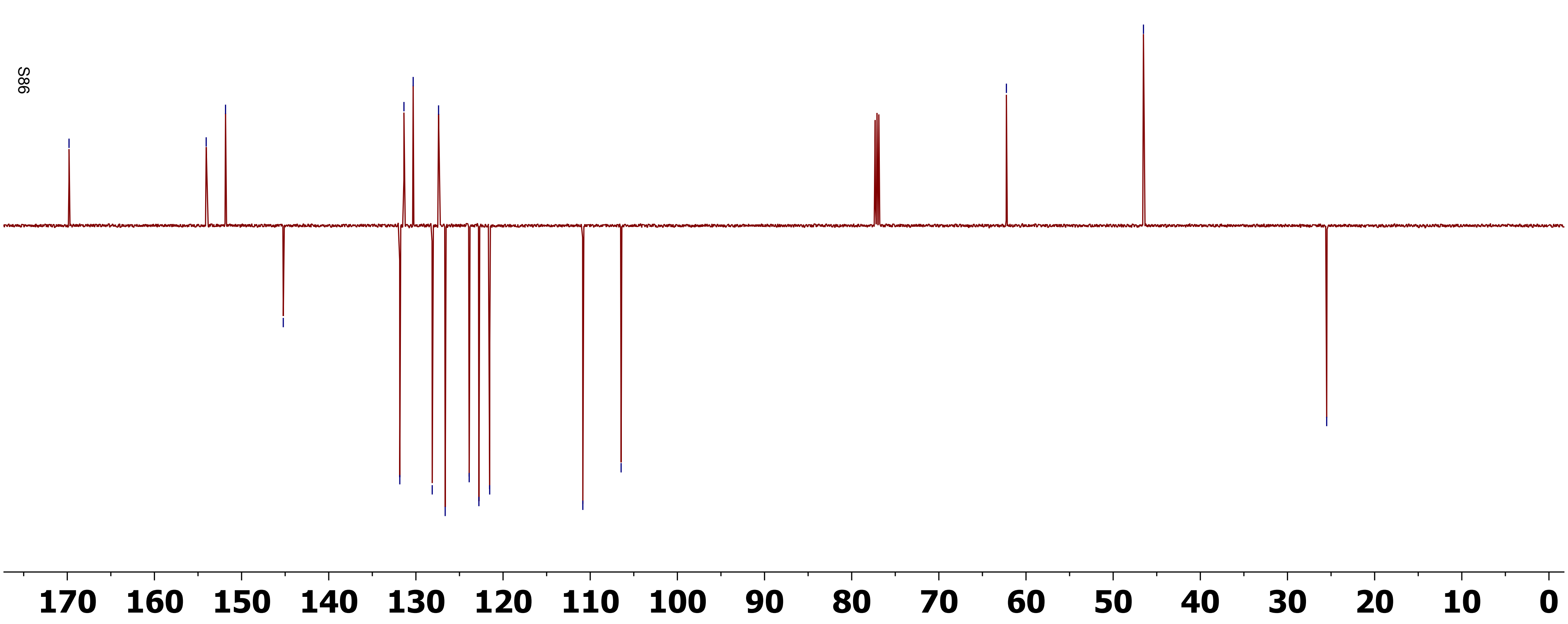

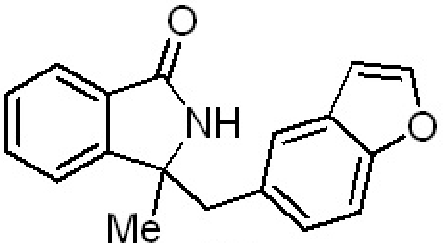

16

	Parameter	Value
1	Title	grd-2-102.4.1
2	Solvent	CDCl3
3	Temperature	298.0
4	Number of Scans	256
5	Receiver Gain	2050.0
6	Relaxation Delay	5.0000
7	Pulse Width	10.6300
8	Spectrometer Frequency	150.95
9	Nucleus	13C

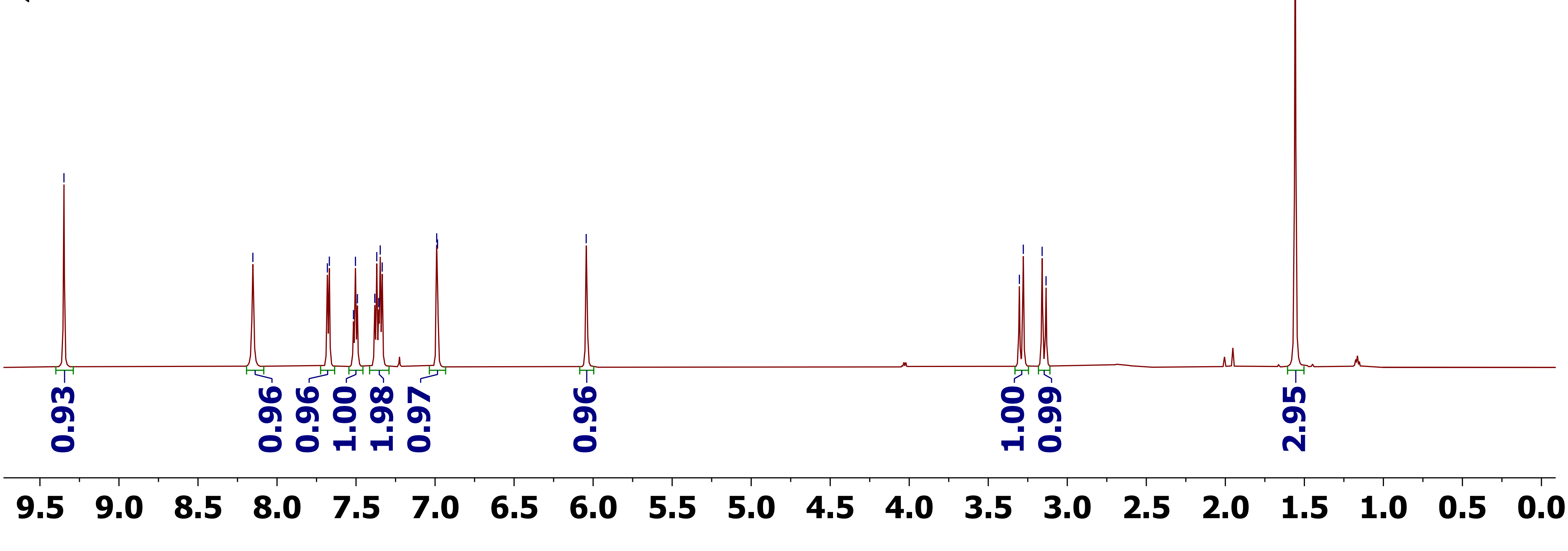

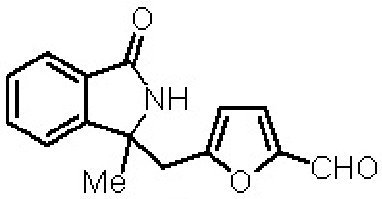

17

	Parameter	Value
1	Title	grd-2-153.1.1
2	Solvent	CDCl3
3	Temperature	297.1
4	Number of Scans	16
5	Receiver Gain	36.0
6	Relaxation Delay	1.0000
7	Pulse Width	10.5000
8	Spectrometer Frequency	600.32
9	Nucleus	1H

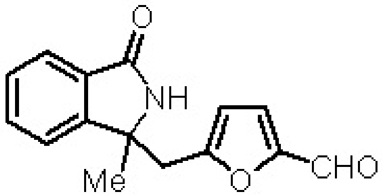

17

	Parameter	Value
1	Title	grd-2-153.2.1
2	Solvent	CDCl3
3	Temperature	299.4
4	Number of Scans	256
5	Receiver Gain	2050.0
6	Relaxation Delay	5.0000
7	Pulse Width	10.6300
8	Spectrometer Frequency	150.95
9	Nucleus	13C

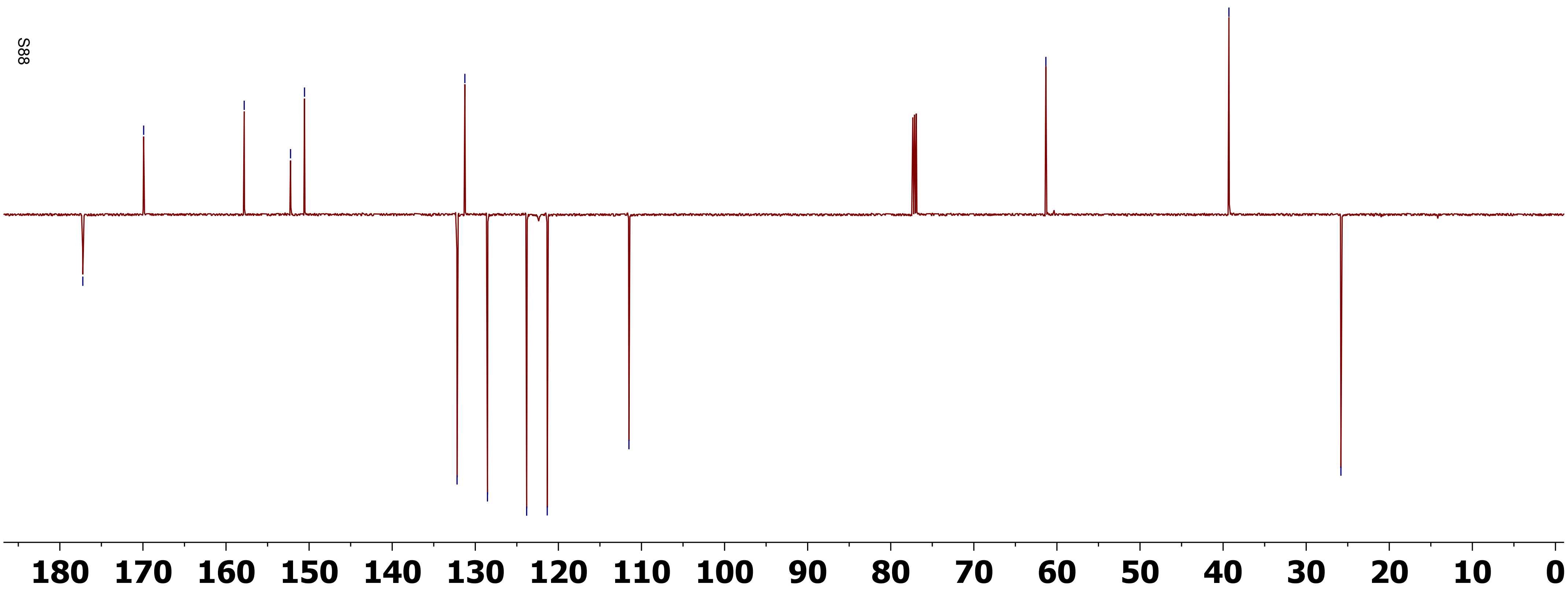

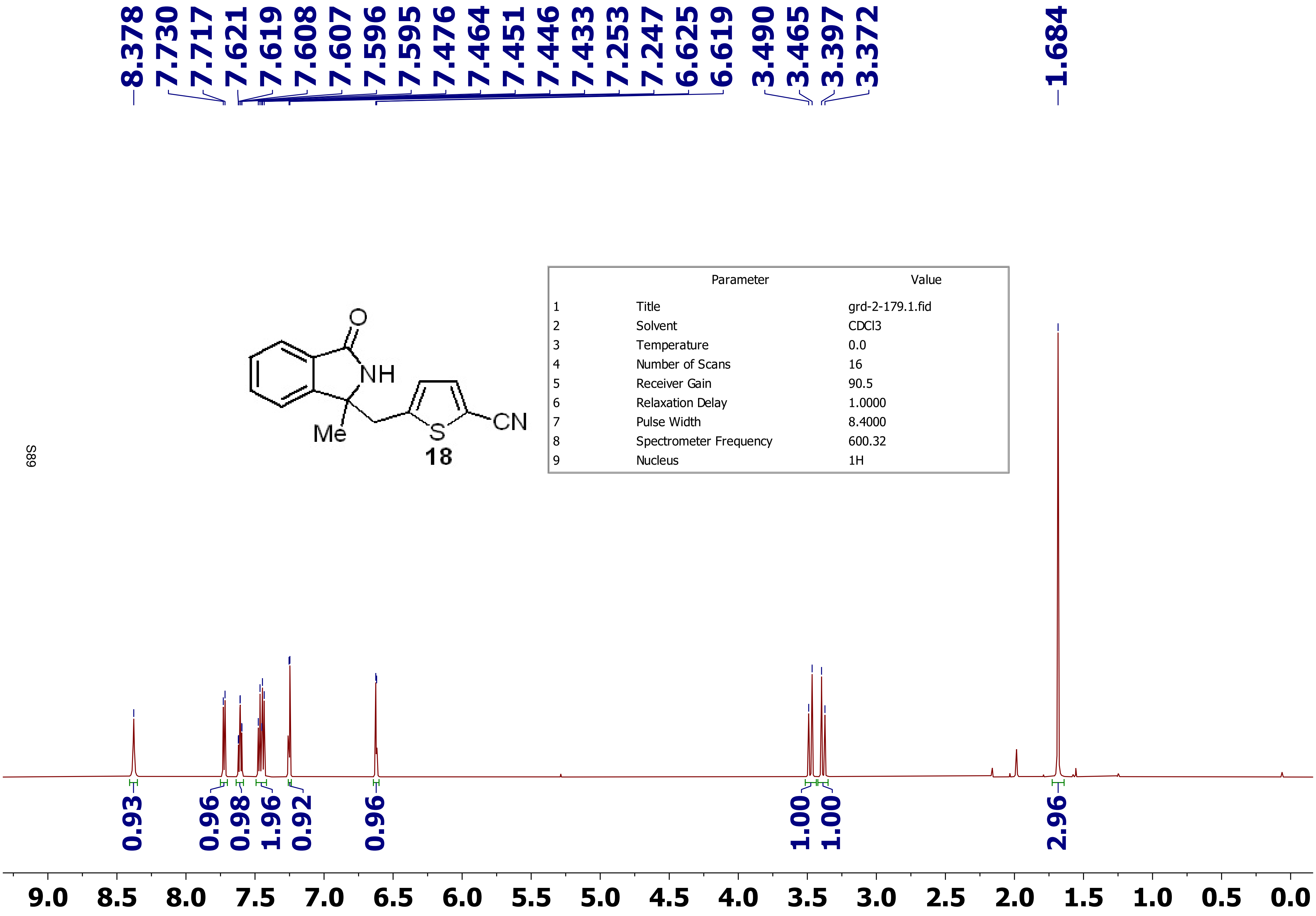

18

	Parameter	Value
1	Title	grd-2-179.1.fid
2	Solvent	CDCl3
3	Temperature	0.0
4	Number of Scans	16
5	Receiver Gain	90.5
6	Relaxation Delay	1.0000
7	Pulse Width	8.4000
8	Spectrometer Frequency	600.32
9	Nucleus	1H

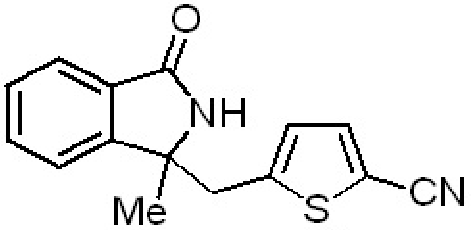

18

	Parameter	Value
1	Title	grd-2-179.2.fid
2	Solvent	CDCl3
3	Temperature	0.0
4	Number of Scans	256
5	Receiver Gain	2050.0
6	Relaxation Delay	5.0000
7	Pulse Width	15.0000
8	Spectrometer Frequency	150.97
9	Nucleus	13C

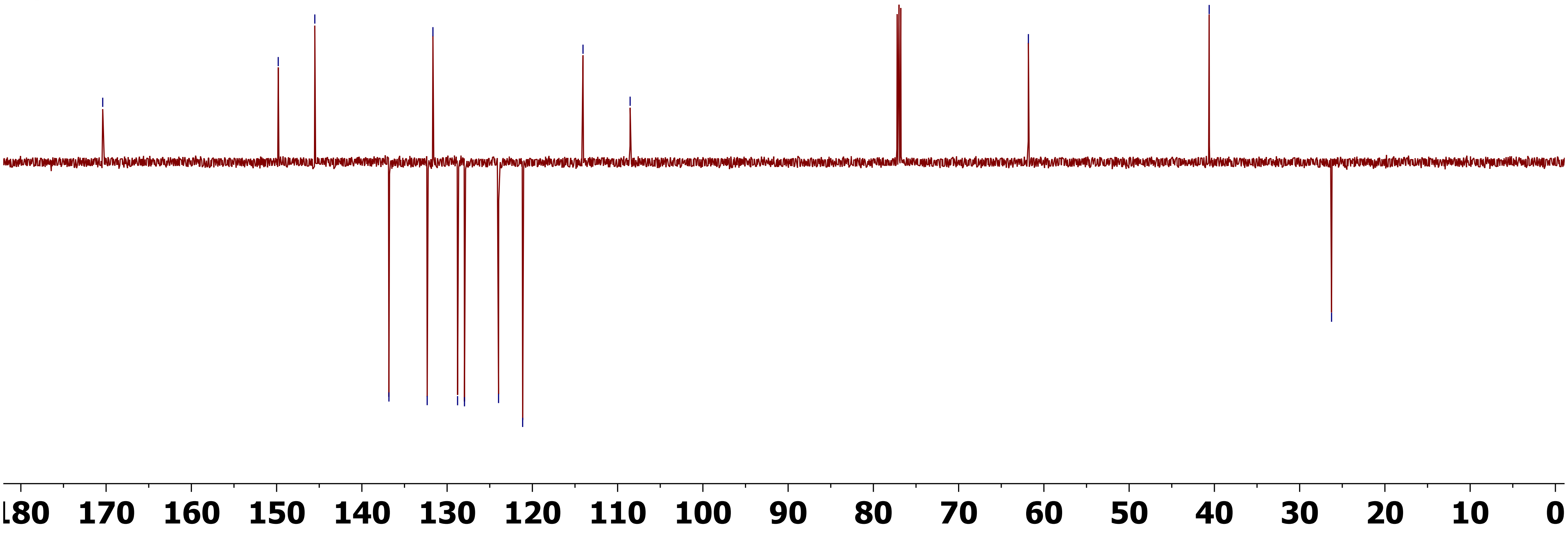

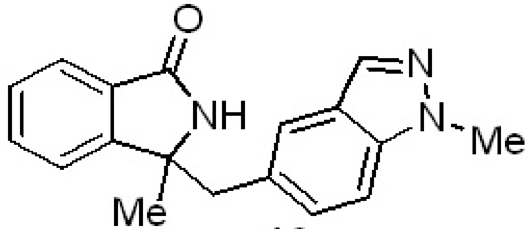

19

	Parameter	Value
1	Title	grd-2-168.3.1
2	Solvent	CDCl3
3	Temperature	298.0
4	Number of Scans	16
5	Receiver Gain	181.0
6	Relaxation Delay	1.0000
7	Pulse Width	10.5000
8	Spectrometer Frequency	600.32
9	Nucleus	1H

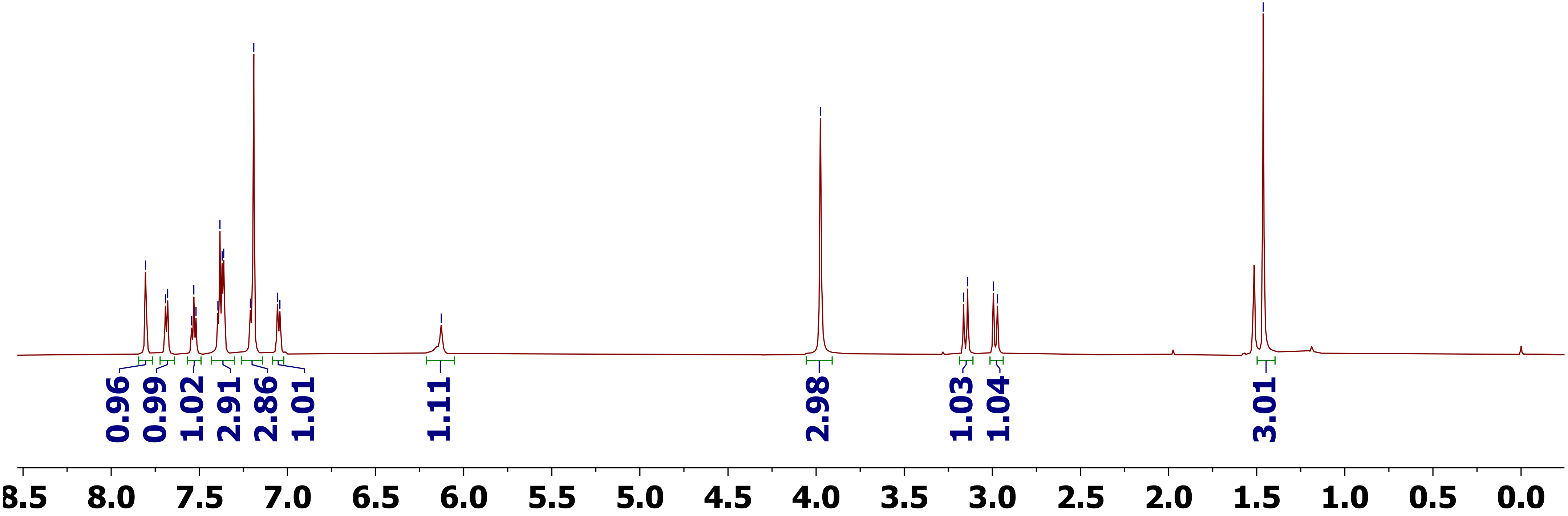

169.94
151.66
139.05
132.41
131.86
131.48
128.94
128.14
127.92
123.99
123.83
122.31
121.57
108.37
62.46
46.44
35.48
25.64

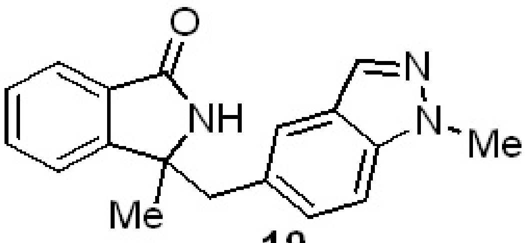

19

	Parameter	Value
1	Title	grd-2-168.2.1
2	Solvent	CDCl3
3	Temperature	298.0
4	Number of Scans	256
5	Receiver Gain	2050.0
6	Relaxation Delay	5.0000
7	Pulse Width	10.6300
8	Spectrometer Frequency	150.95
9	Nucleus	13C

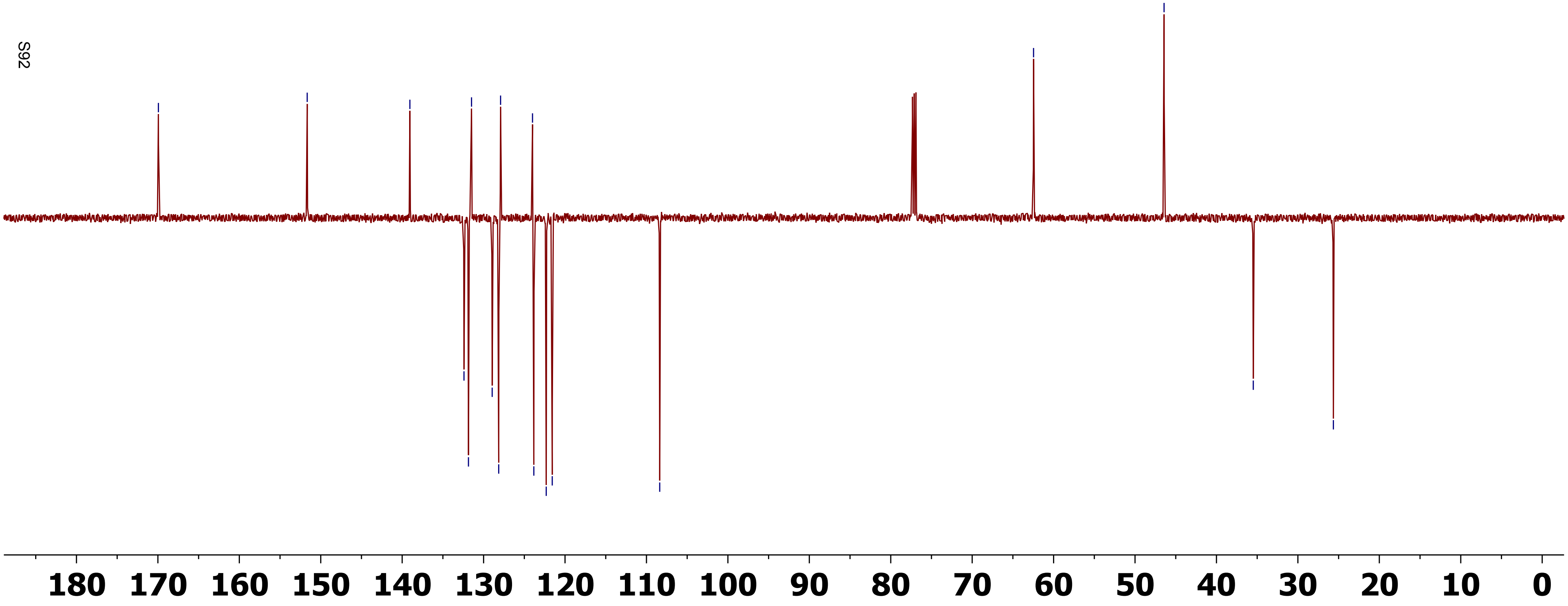

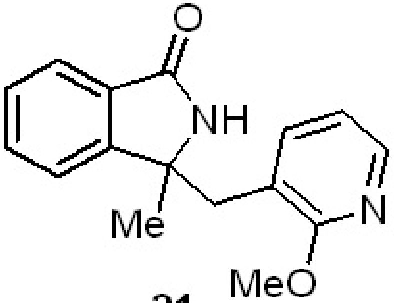

21

	Parameter	Value
1	Title	grd-2-162.1.1
2	Solvent	CDCl3
3	Temperature	298.0
4	Number of Scans	16
5	Receiver Gain	12.7
6	Relaxation Delay	1.0000
7	Pulse Width	10.5000
8	Spectrometer Frequency	600.32
9	Nucleus	1H

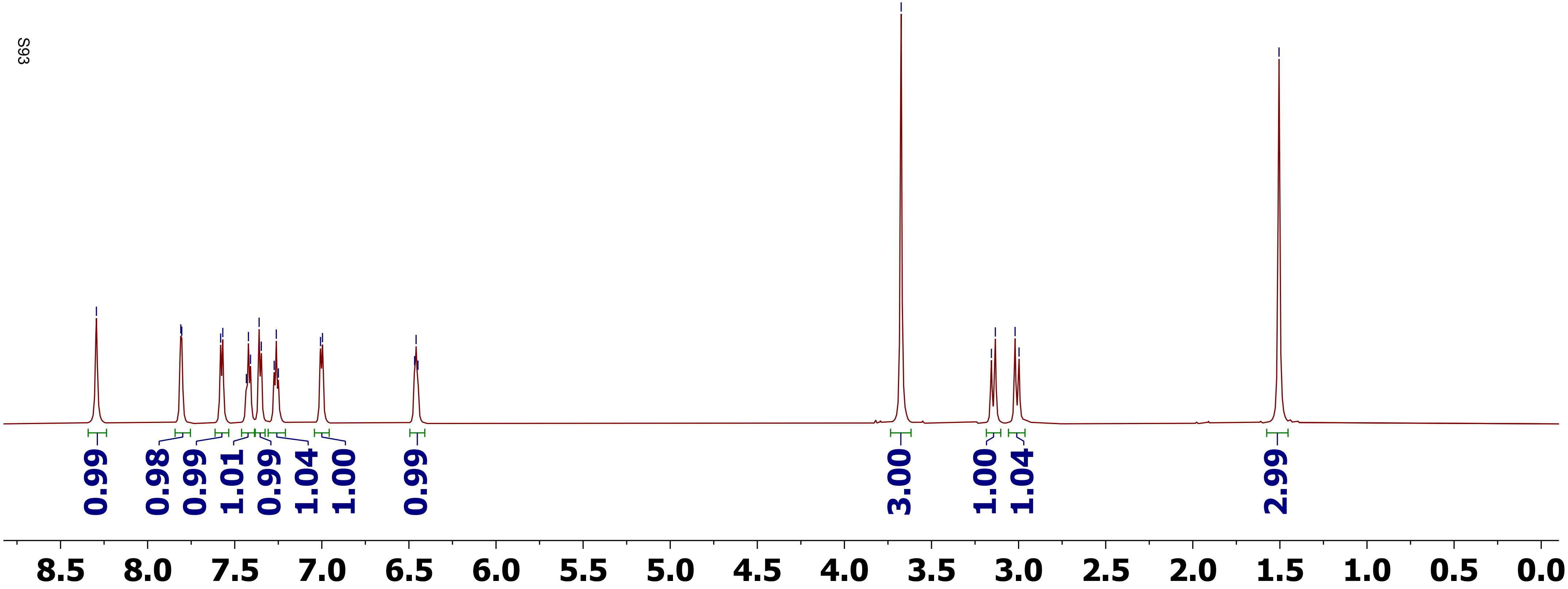

170.14 162.19 150.90 145.39 139.81 131.55 131.31 127.99 123.29 121.91 118.53 116.29 62.68 53.04 39.04 26.22

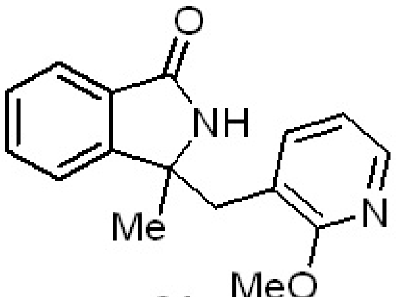

21

	Parameter	Value
1	Title	grd-2-162.2.1
2	Solvent	CDCl3
3	Temperature	298.0
4	Number of Scans	256
5	Receiver Gain	2050.0
6	Relaxation Delay	5.0000
7	Pulse Width	10.6300
8	Spectrometer Frequency	150.95
9	Nucleus	13C

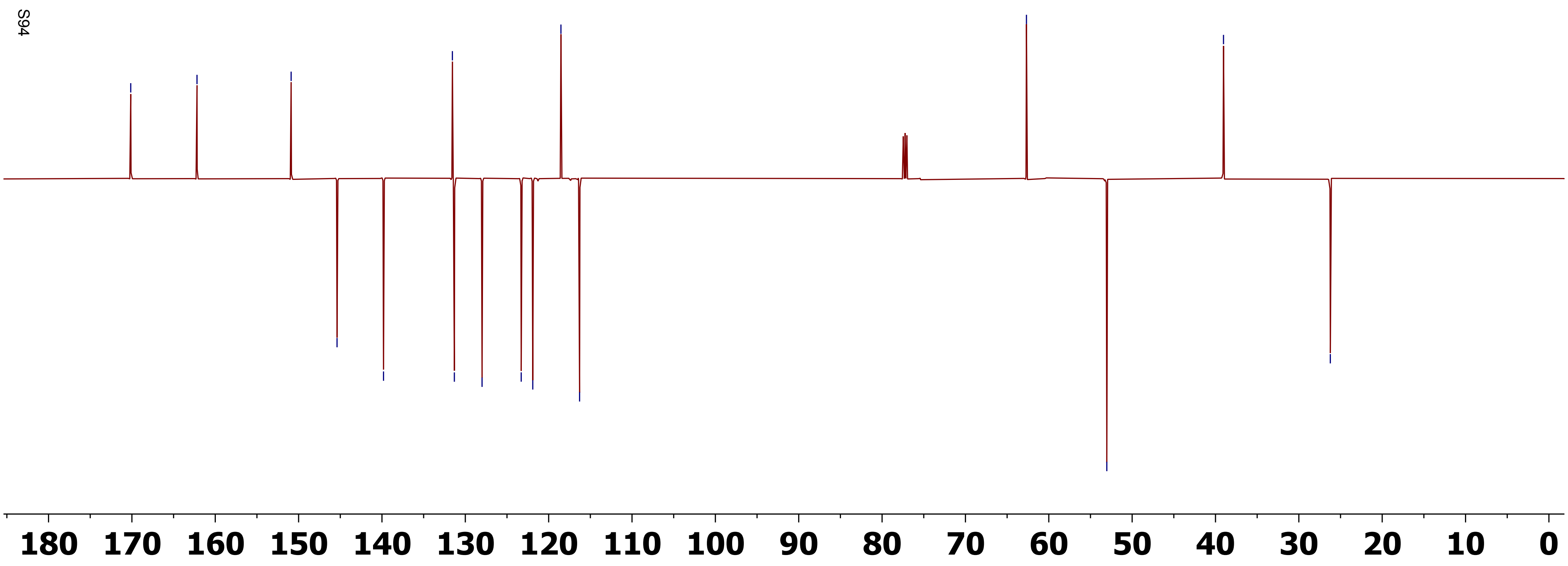

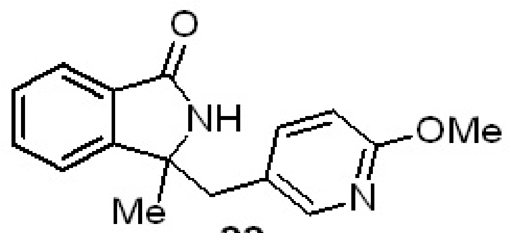

22

	Parameter	Value
1	Title	grd-2-161.4.1
2	Solvent	CDCl3
3	Temperature	298.0
4	Number of Scans	16
5	Receiver Gain	57.0
6	Relaxation Delay	1.0000
7	Pulse Width	10.5000
8	Spectrometer Frequency	600.32
9	Nucleus	1H

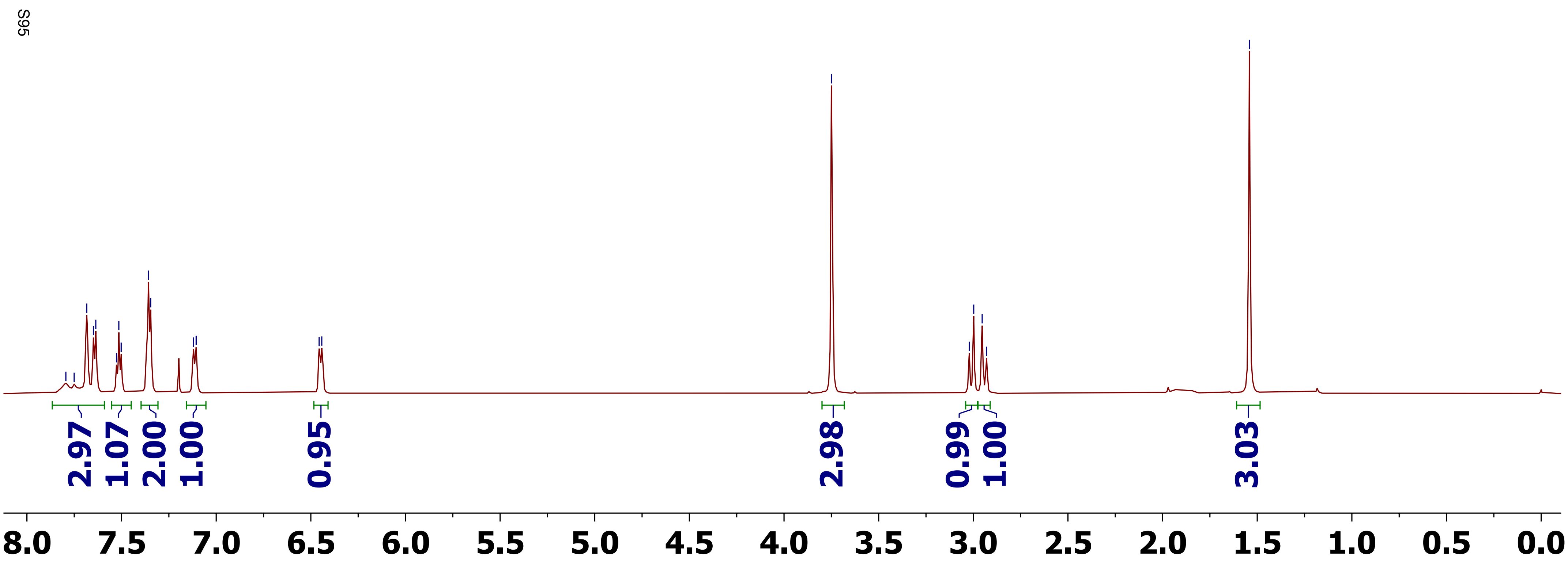

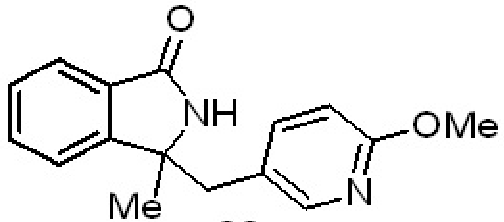

22

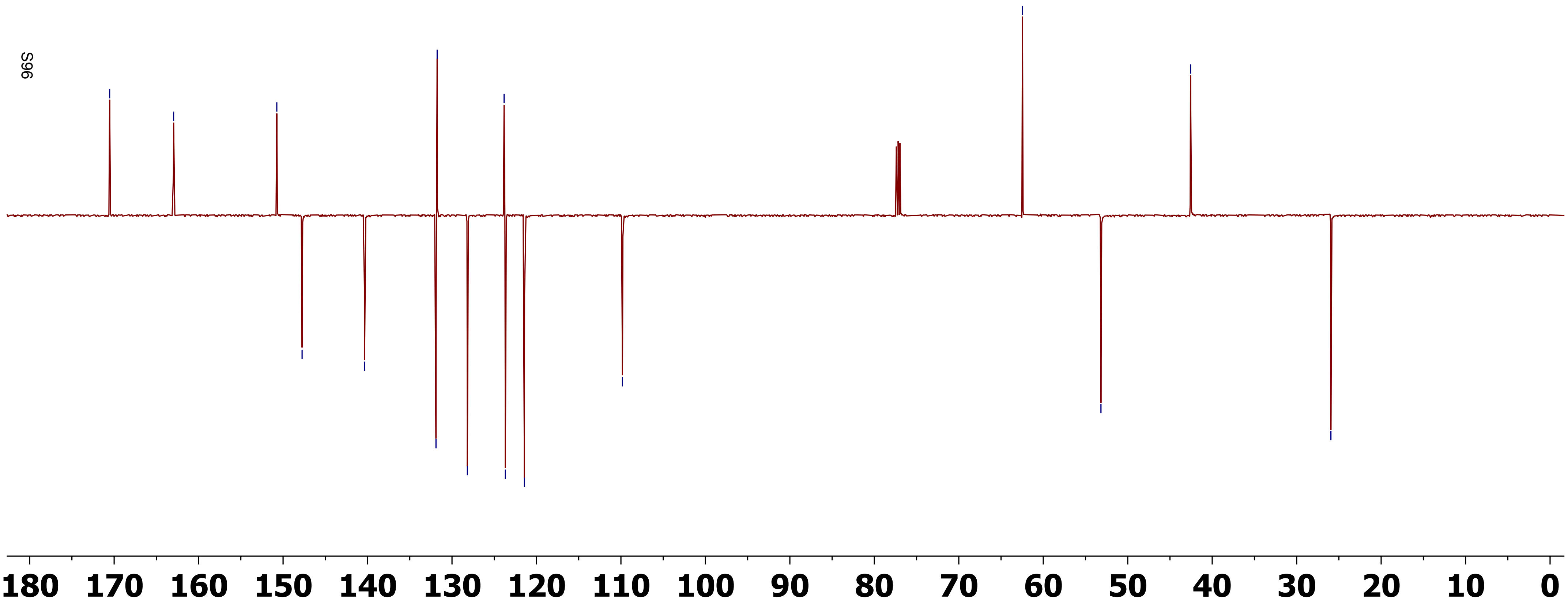

	Parameter	Value
1	Title	grd-2-161.2.1
2	Solvent	CDCl3
3	Temperature	298.0
4	Number of Scans	256
5	Receiver Gain	2050.0
6	Relaxation Delay	5.0000
7	Pulse Width	10.6300
8	Spectrometer Frequency	150.95
9	Nucleus	13C

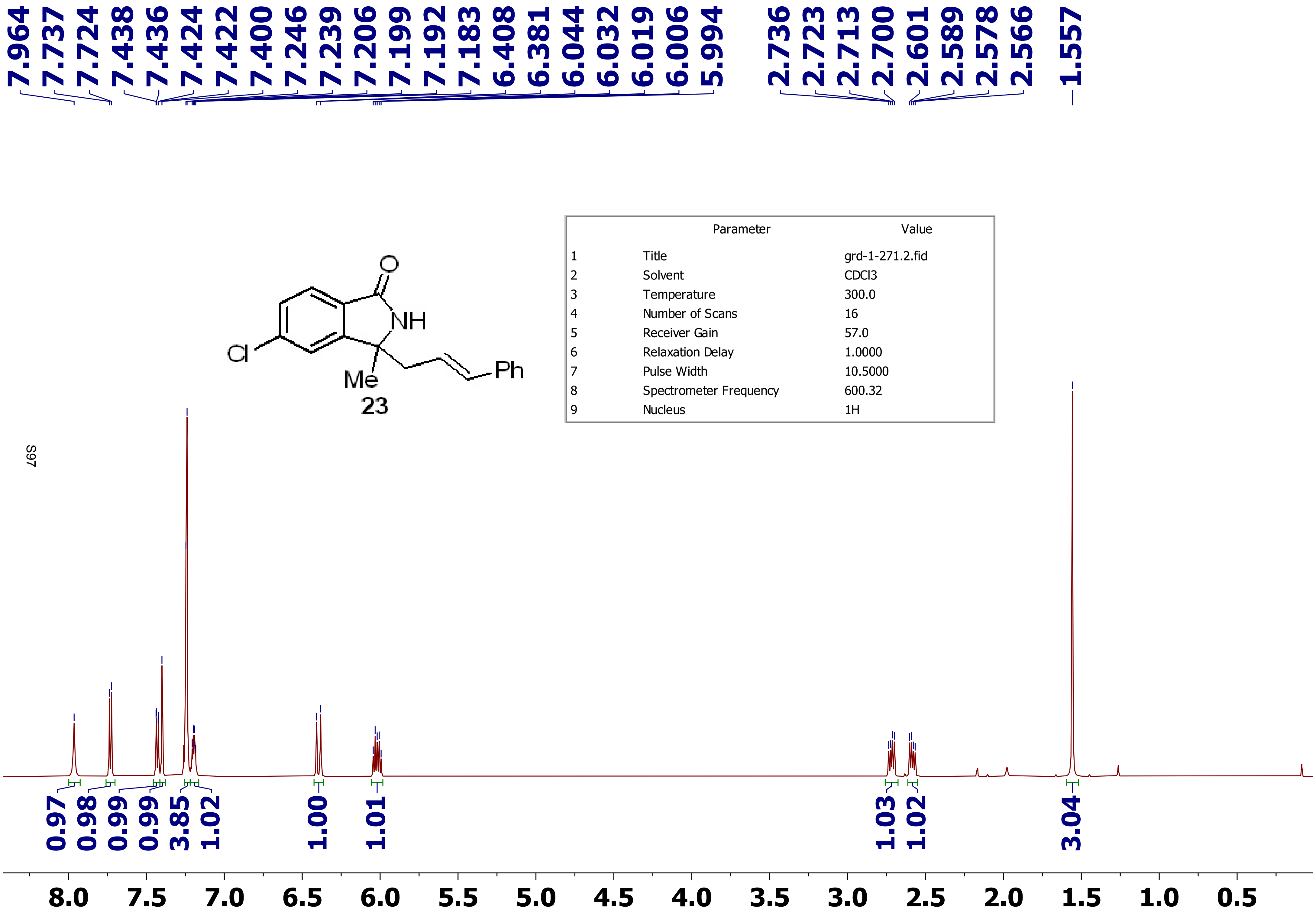

	Parameter	Value
1	Title	grd-1-271.2.fid
2	Solvent	CDCl3
3	Temperature	300.0
4	Number of Scans	16
5	Receiver Gain	57.0
6	Relaxation Delay	1.0000
7	Pulse Width	10.5000
8	Spectrometer Frequency	600.32
9	Nucleus	1H

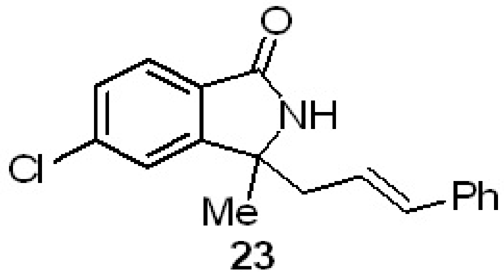

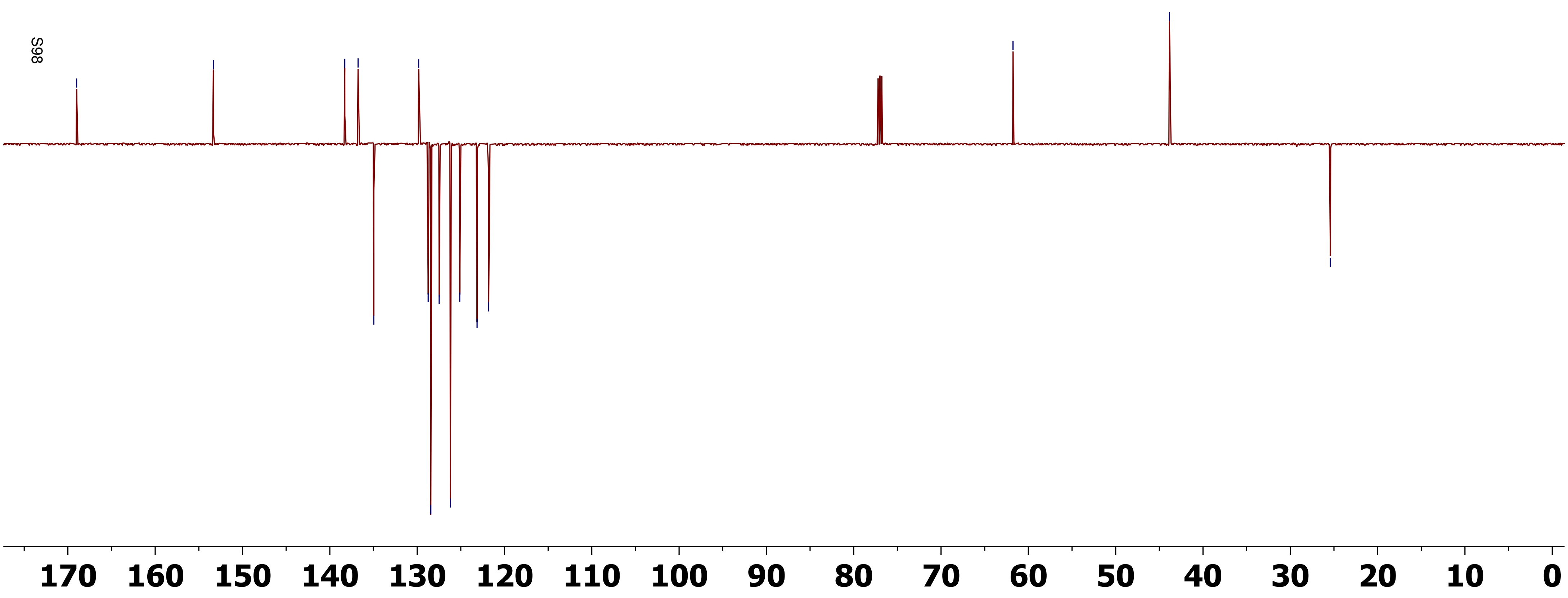

	Parameter	Value
1	Title	grd-1-271.3.fid
2	Solvent	CDCl3
3	Temperature	300.0
4	Number of Scans	256
5	Receiver Gain	2050.0
6	Relaxation Delay	5.0000
7	Pulse Width	10.6300
8	Spectrometer Frequency	150.97
9	Nucleus	13C

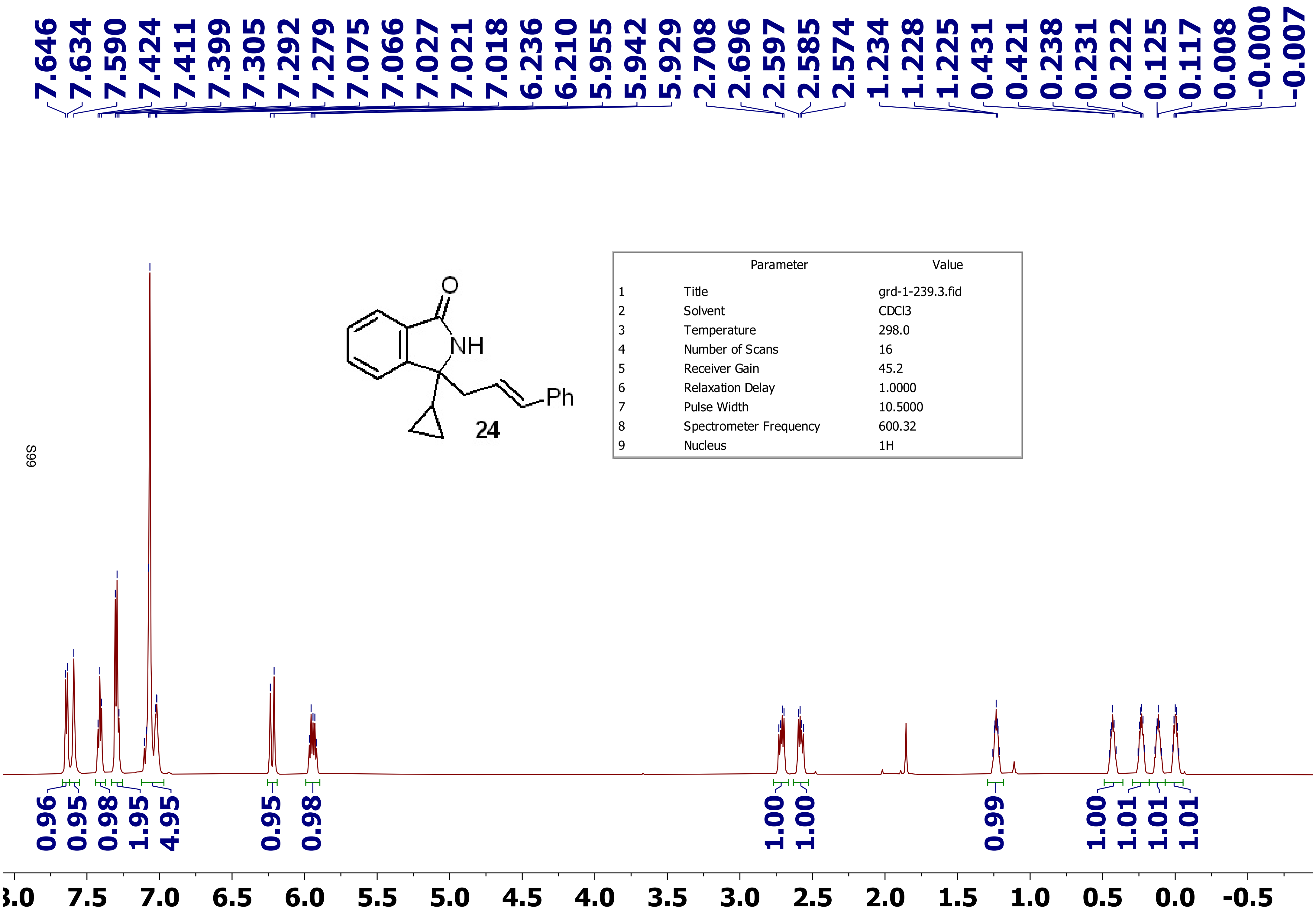

24

	Parameter	Value
1	Title	grd-1-239.3.fid
2	Solvent	CDCl3
3	Temperature	298.0
4	Number of Scans	16
5	Receiver Gain	45.2
6	Relaxation Delay	1.0000
7	Pulse Width	10.5000
8	Spectrometer Frequency	600.32
9	Nucleus	1H

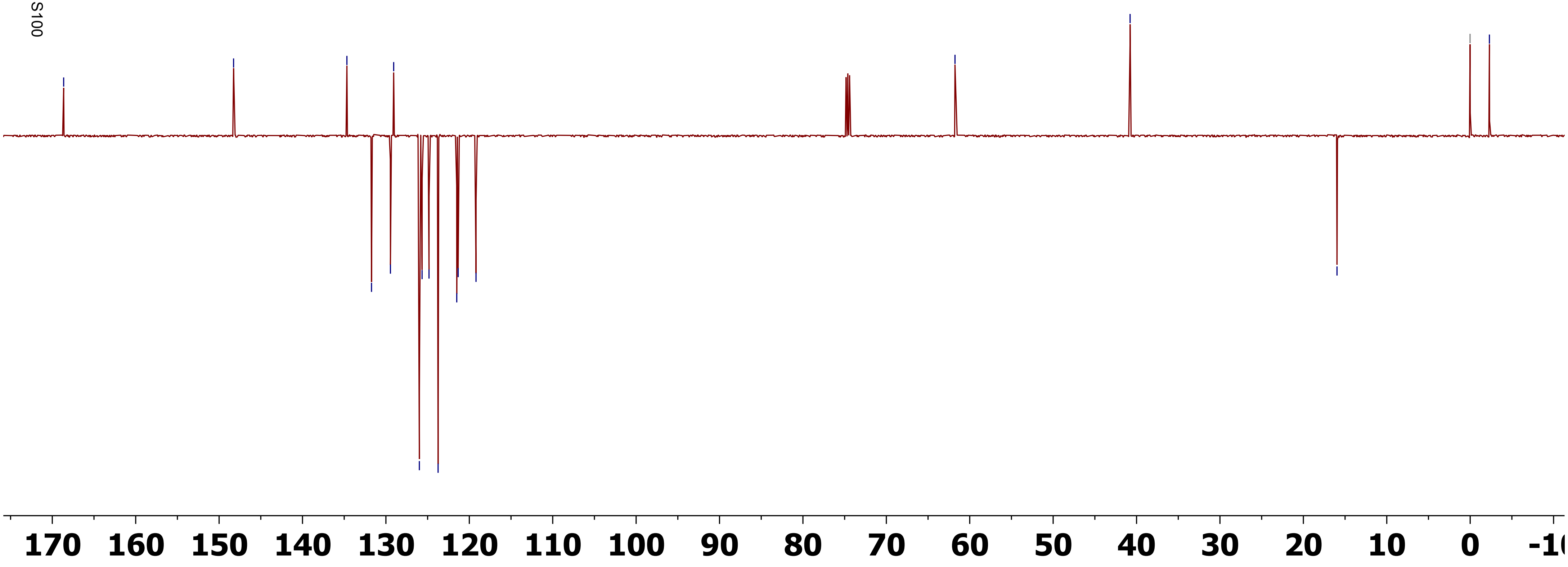

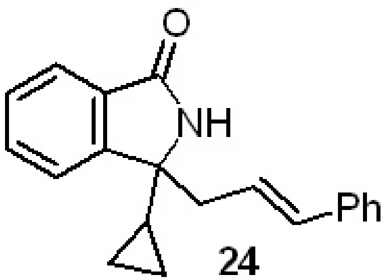

24

	Parameter	Value
1	Title	grd-1-239.4.fid
2	Solvent	CDCl3
3	Temperature	298.0
4	Number of Scans	256
5	Receiver Gain	2050.0
6	Relaxation Delay	5.0000
7	Pulse Width	10.6300
8	Spectrometer Frequency	150.97
9	Nucleus	13C

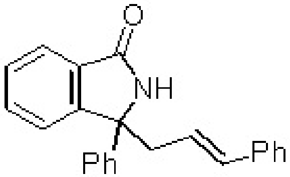

25

	Parameter	Value
1	Title	grd-1-213.1.fid
2	Solvent	CDCl3
3	Temperature	298.0
4	Number of Scans	16
5	Receiver Gain	57.0
6	Relaxation Delay	1.0000
7	Pulse Width	10.5000
8	Spectrometer Frequency	600.32
9	Nucleus	1H

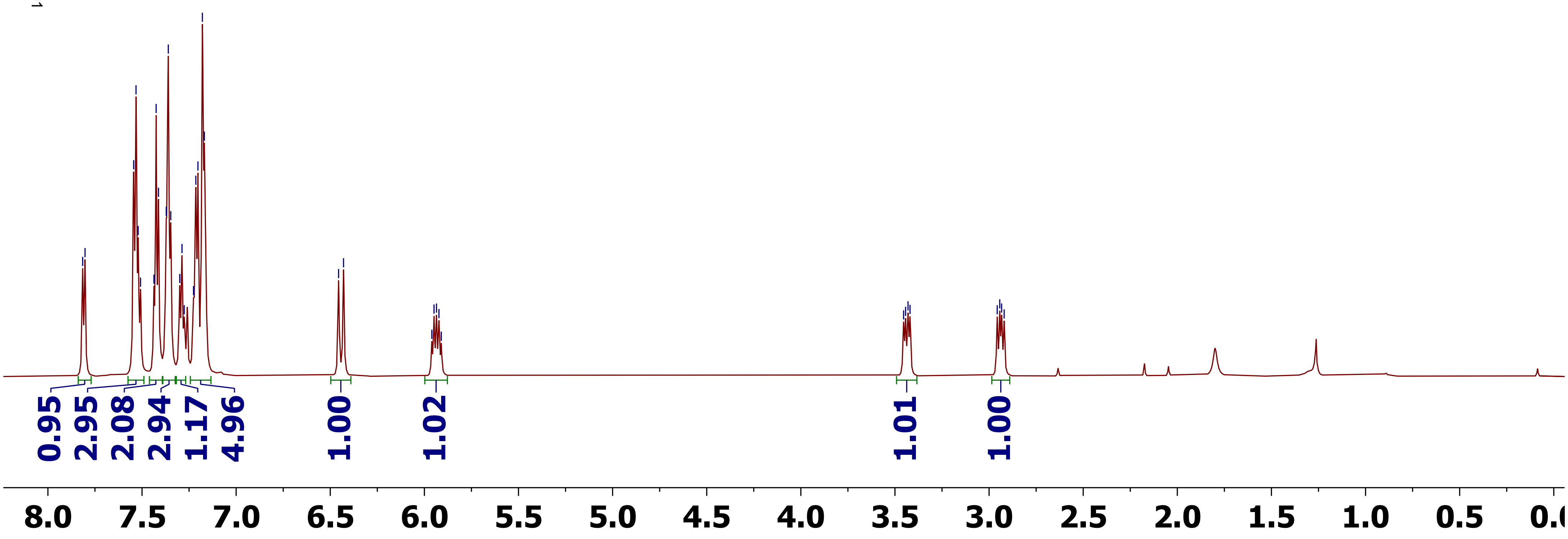

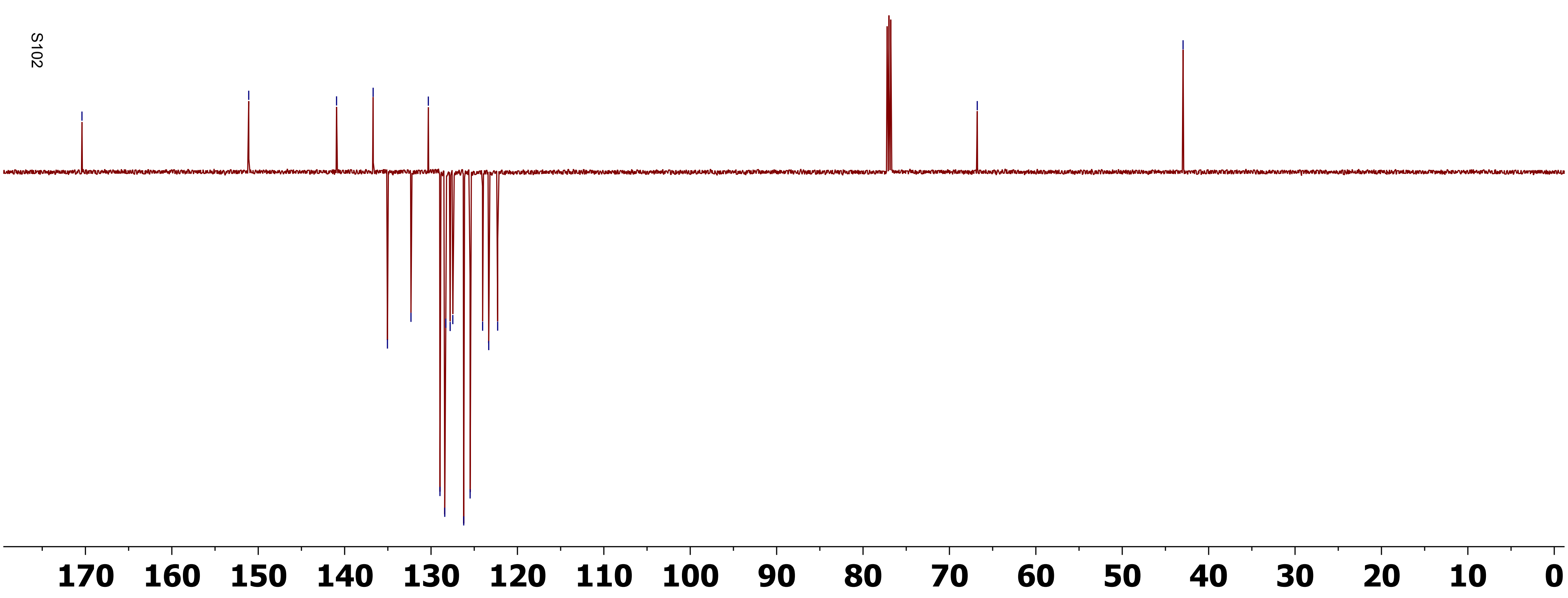

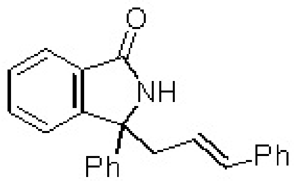

25

	Parameter	Value
1	Title	grd-1-213.2.fid
2	Solvent	CDCl3
3	Temperature	298.0
4	Number of Scans	256
5	Receiver Gain	2050.0
6	Relaxation Delay	5.0000
7	Pulse Width	10.6300
8	Spectrometer Frequency	150.97
9	Nucleus	13C

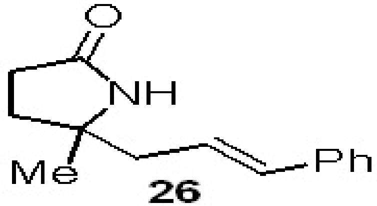

26

	Parameter	Value
1	Title	grd-1-192.2.fid
2	Solvent	CDCl3
3	Temperature	300.0
4	Number of Scans	16
5	Receiver Gain	32.0
6	Relaxation Delay	1.0000
7	Pulse Width	10.5000
8	Spectrometer Frequency	600.32
9	Nucleus	1H

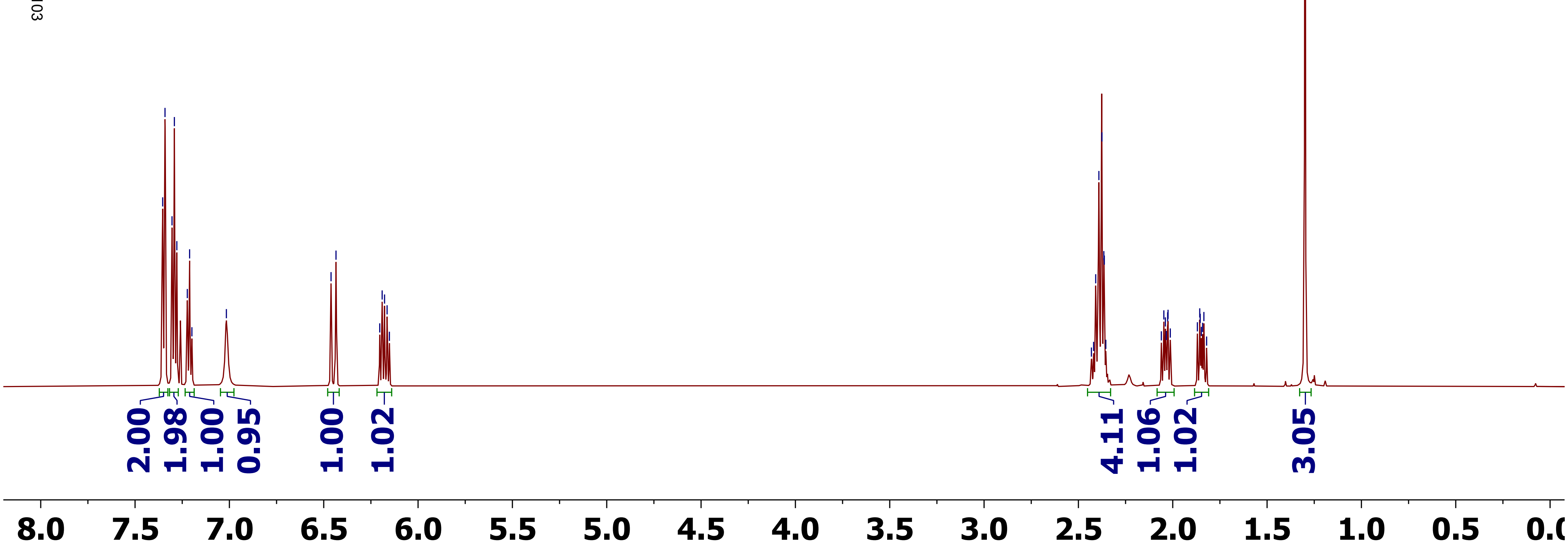

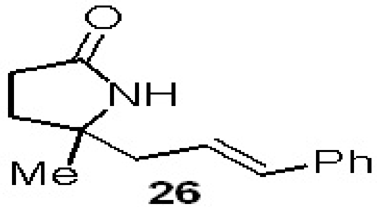

26

	Parameter	Value
1	Title	grd-1-192.3.fid
2	Solvent	CDCl3
3	Temperature	300.0
4	Number of Scans	256
5	Receiver Gain	2050.0
6	Relaxation Delay	5.0000
7	Pulse Width	10.6300
8	Spectrometer Frequency	150.97
9	Nucleus	13C

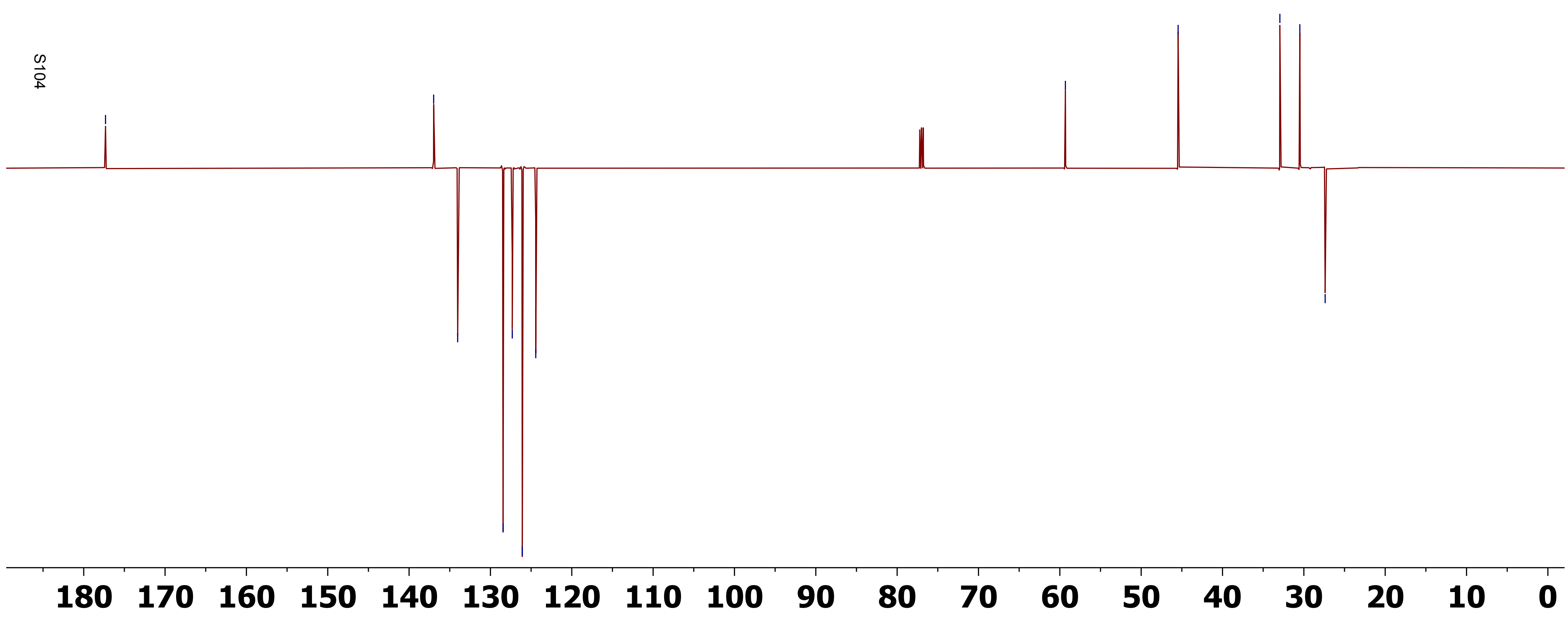

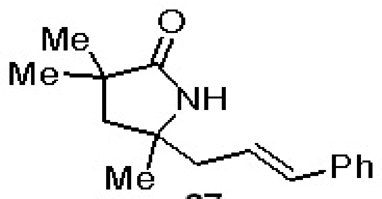

27

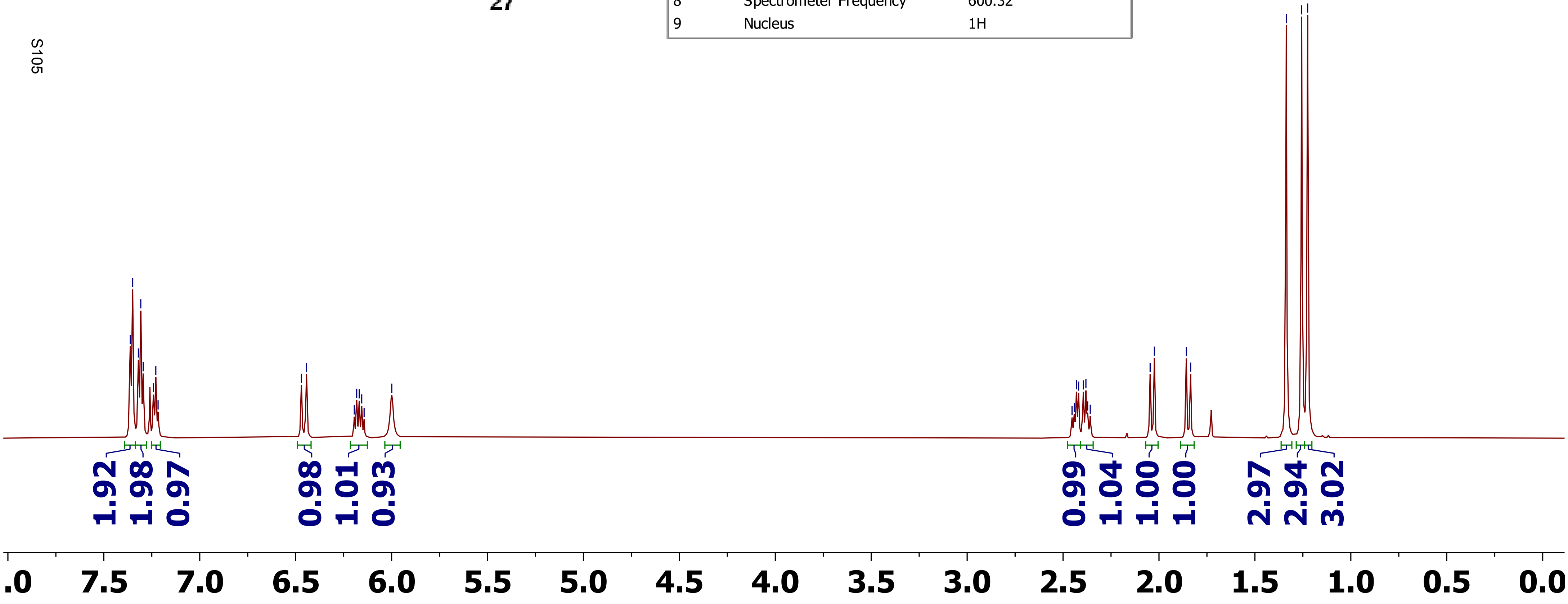

	Parameter	Value
1	Title	grd-1-206.4.fid
2	Solvent	CDCl3
3	Temperature	298.0
4	Number of Scans	16
5	Receiver Gain	57.0
6	Relaxation Delay	1.0000
7	Pulse Width	10.5000
8	Spectrometer Frequency	600.32
9	Nucleus	1H

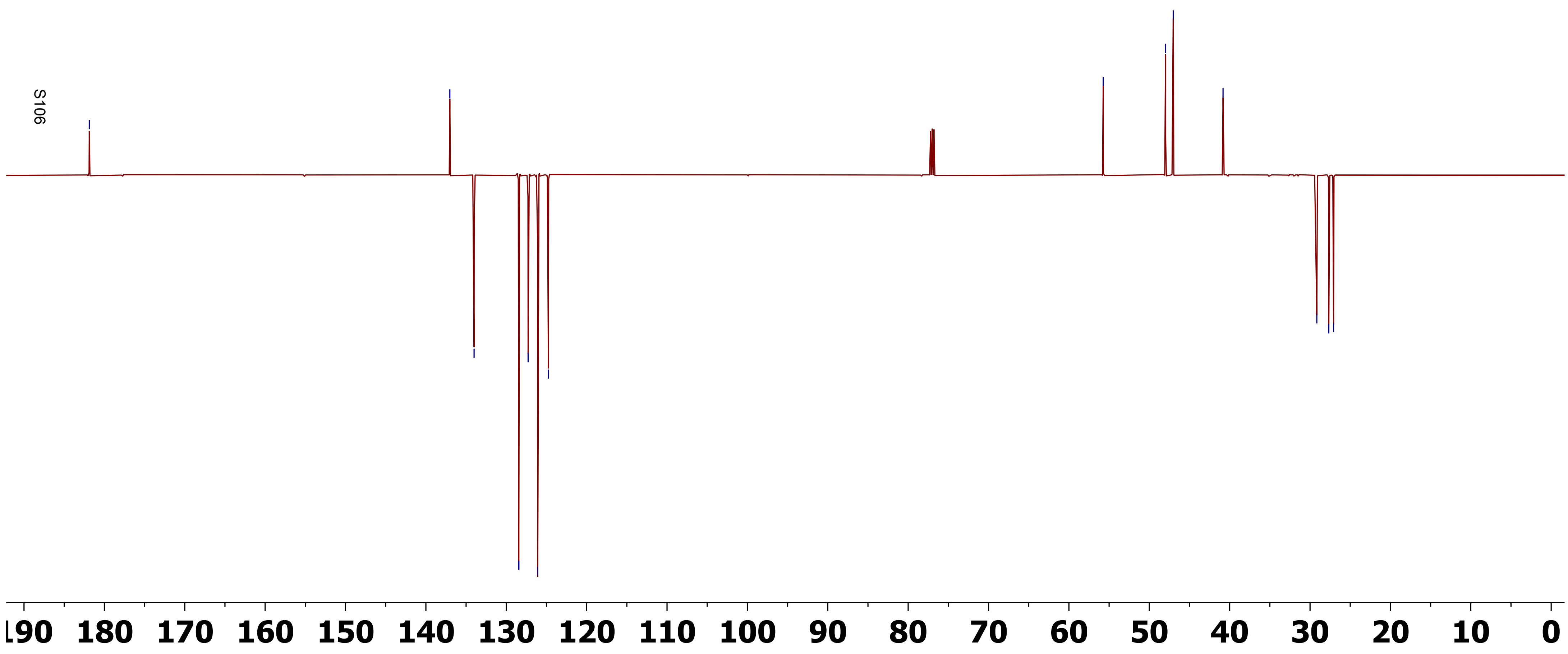

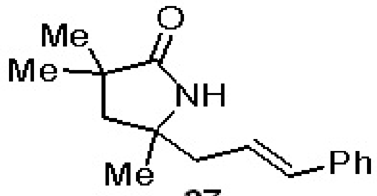

27

	Parameter	Value
1	Title	grd-1-206.3.fid
2	Solvent	CDCl3
3	Temperature	298.0
4	Number of Scans	256
5	Receiver Gain	2050.0
6	Relaxation Delay	5.0000
7	Pulse Width	10.6300
8	Spectrometer Frequency	150.97
9	Nucleus	13C

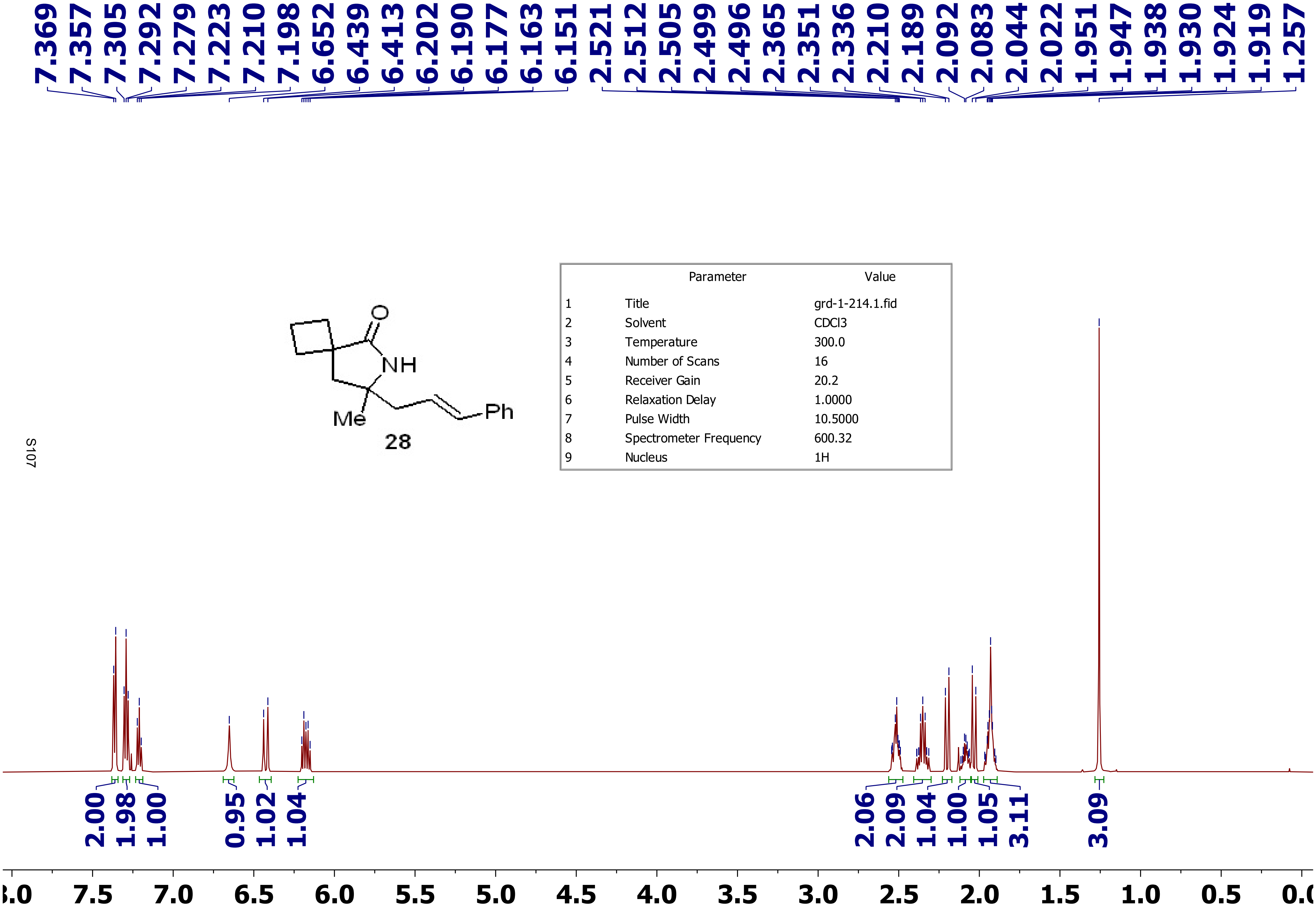

28

	Parameter	Value
1	Title	grd-1-214.1.fid
2	Solvent	CDCl3
3	Temperature	300.0
4	Number of Scans	16
5	Receiver Gain	20.2
6	Relaxation Delay	1.0000
7	Pulse Width	10.5000
8	Spectrometer Frequency	600.32
9	Nucleus	1H

	Parameter	Value
1	Title	grd-1-214.2.fid
2	Solvent	CDCl3
3	Temperature	300.0
4	Number of Scans	256
5	Receiver Gain	2050.0
6	Relaxation Delay	5.0000
7	Pulse Width	10.6300
8	Spectrometer Frequency	150.97
9	Nucleus	13C

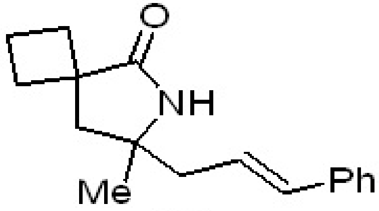

28

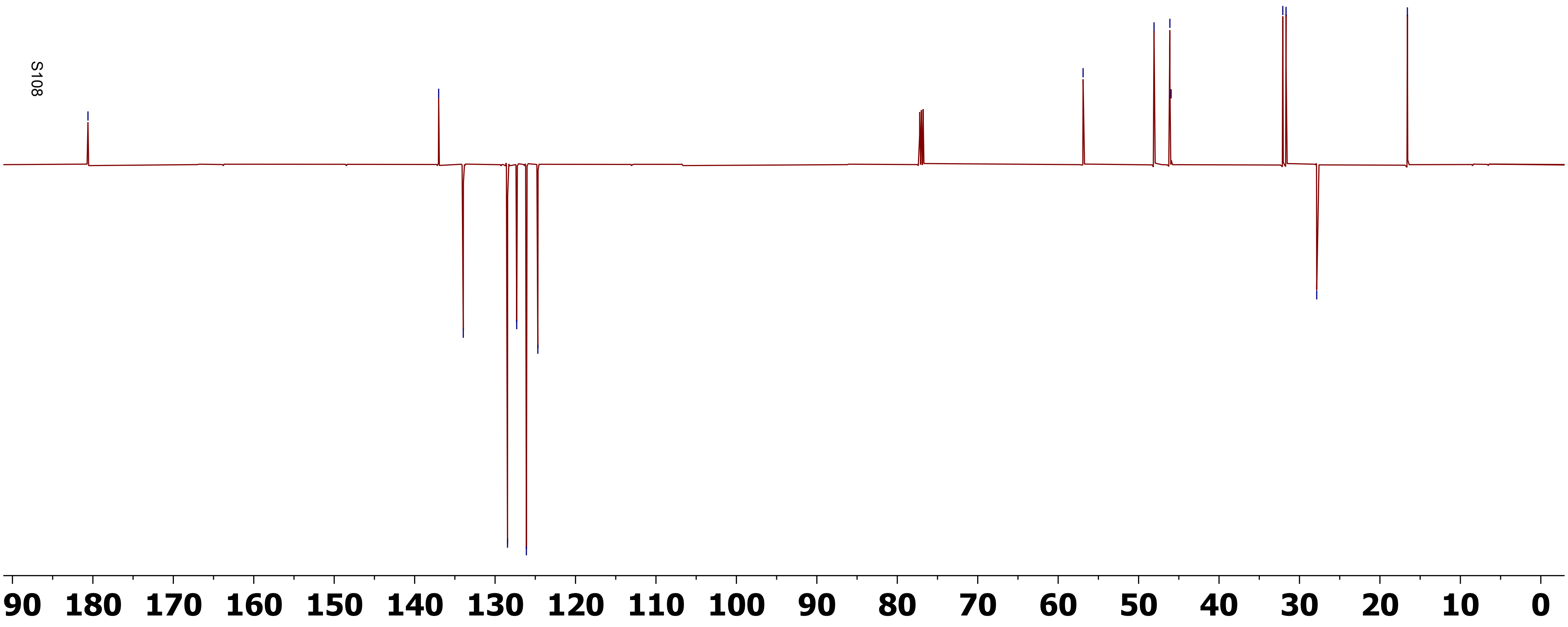

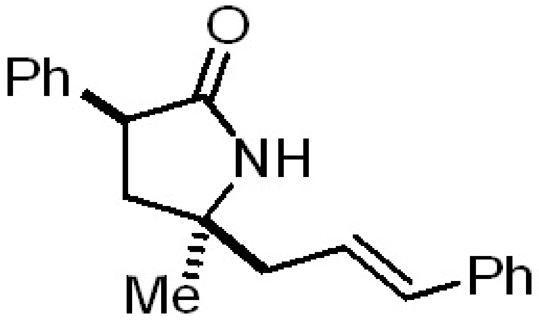

29

	Parameter	Value
1	Title	grd-1-208.1.fid
2	Solvent	CDCl3
3	Temperature	300.0
4	Number of Scans	16
5	Receiver Gain	12.7
6	Relaxation Delay	1.0000
7	Pulse Width	10.5000
8	Spectrometer Frequency	600.32
9	Nucleus	1H

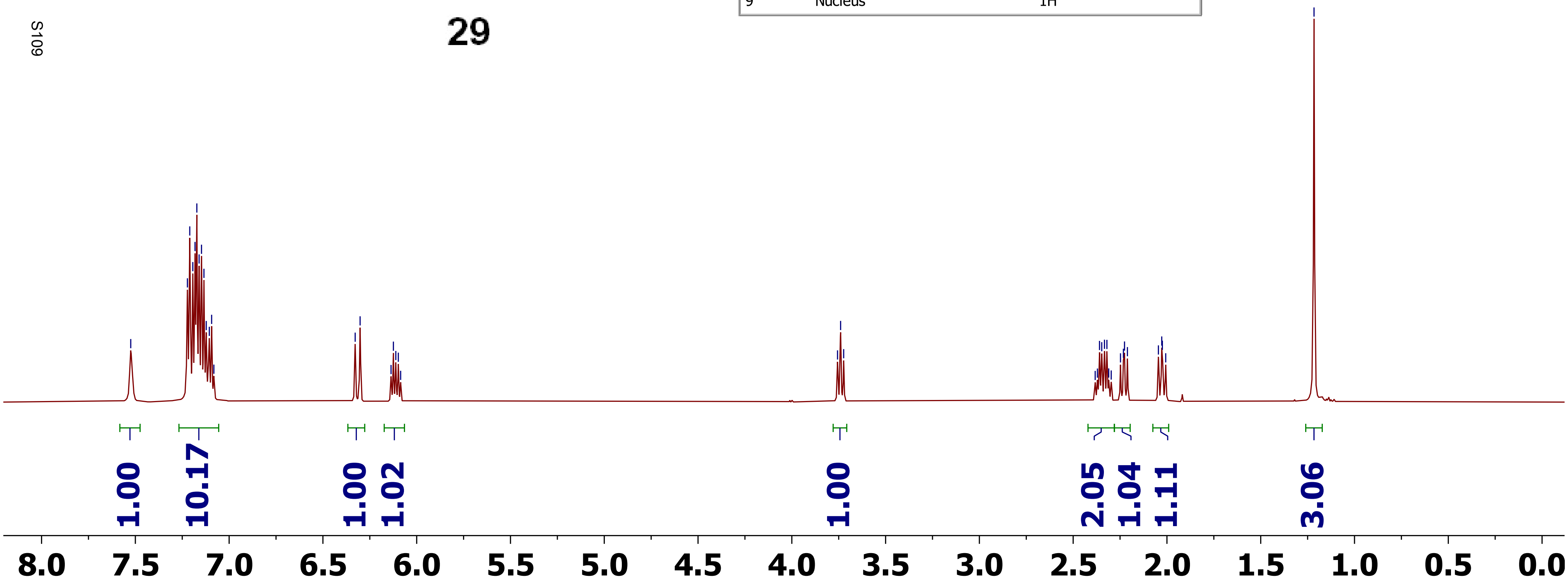

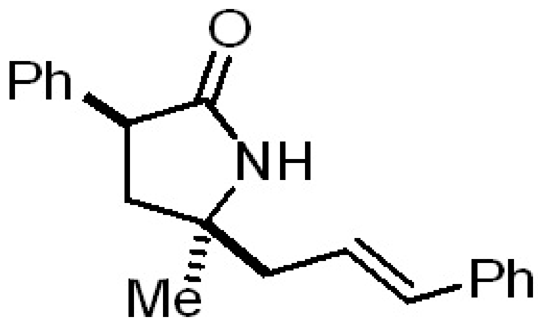

29

	Parameter	Value
1	Title	grd-1-208.2.fid
2	Solvent	CDCl3
3	Temperature	300.0
4	Number of Scans	256
5	Receiver Gain	2050.0
6	Relaxation Delay	5.0000
7	Pulse Width	10.6300
8	Spectrometer Frequency	150.97
9	Nucleus	13C

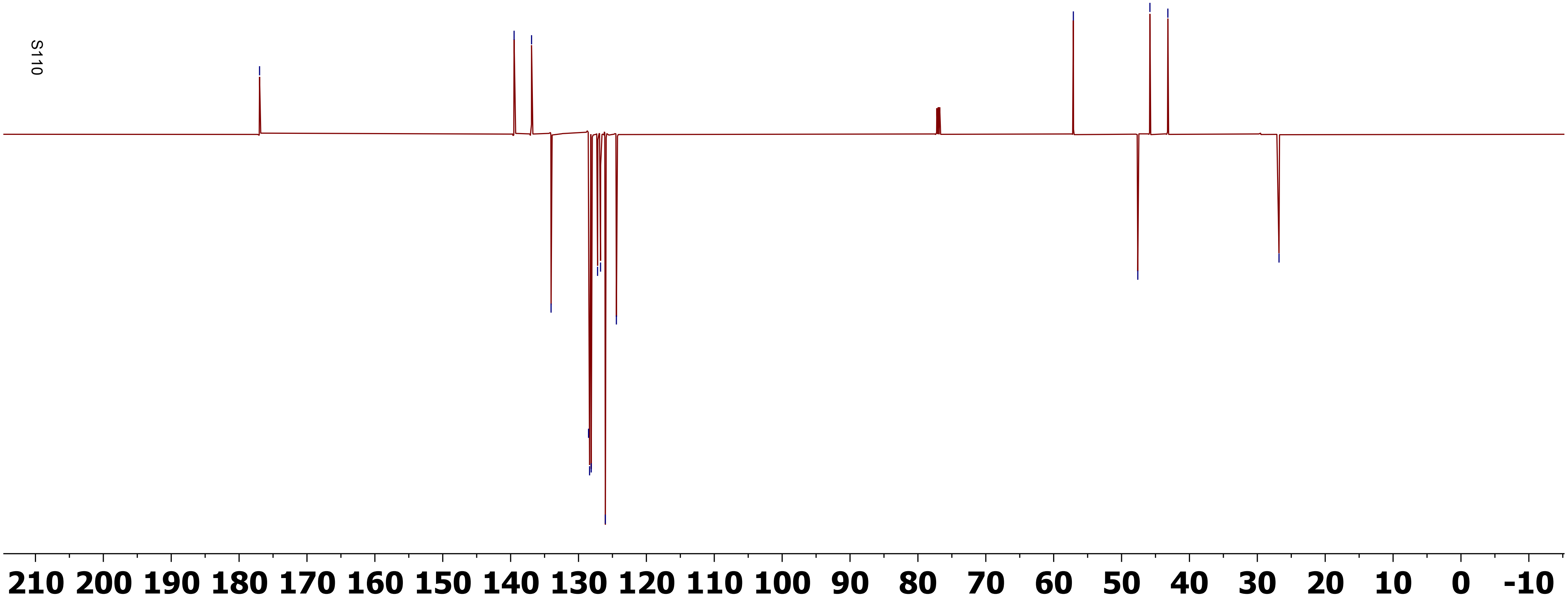

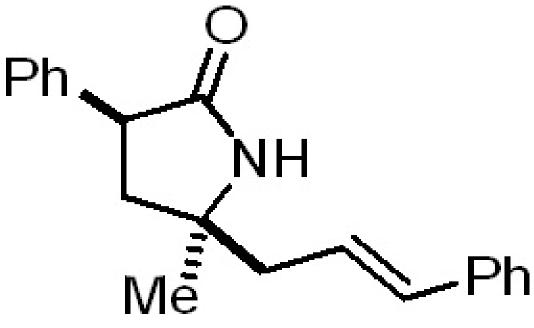

29

	Parameter	Value
1	Title	grd-1-208.6.fid
2	Solvent	CDCl3
3	Temperature	298.2
4	Number of Scans	64
5	Receiver Gain	18.0
6	Relaxation Delay	2.0000
7	Pulse Width	15.0000
8	Spectrometer Frequency	400.13
9	Nucleus	1H

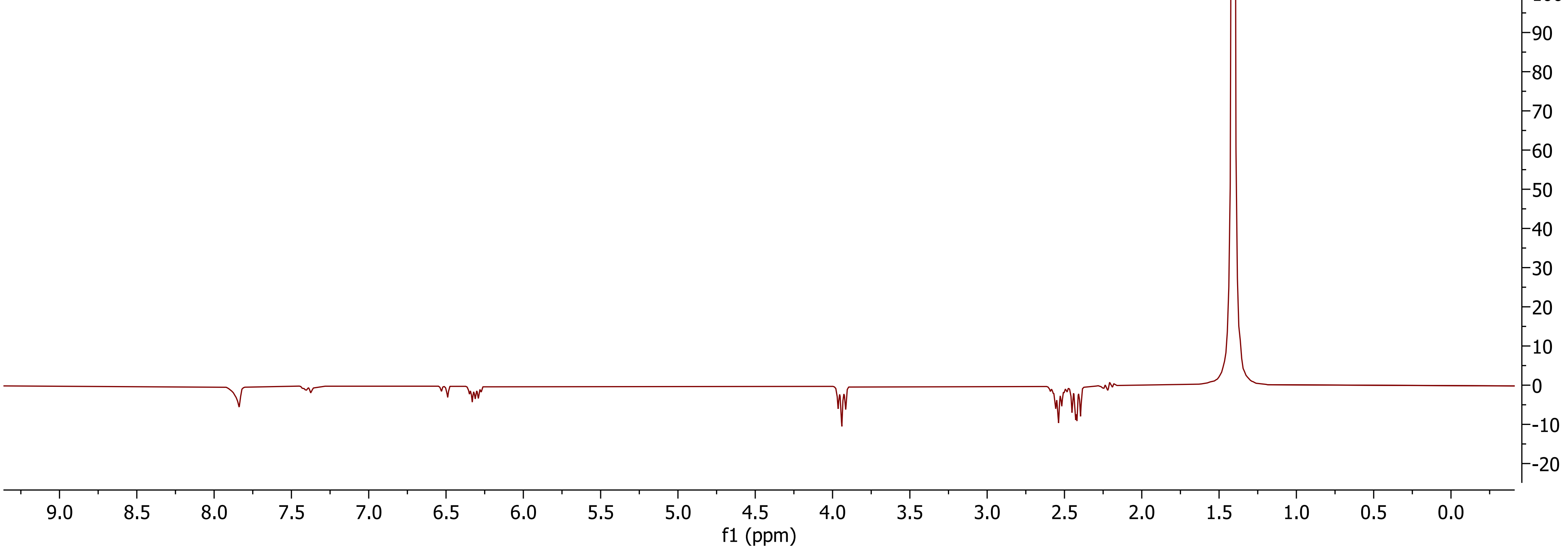

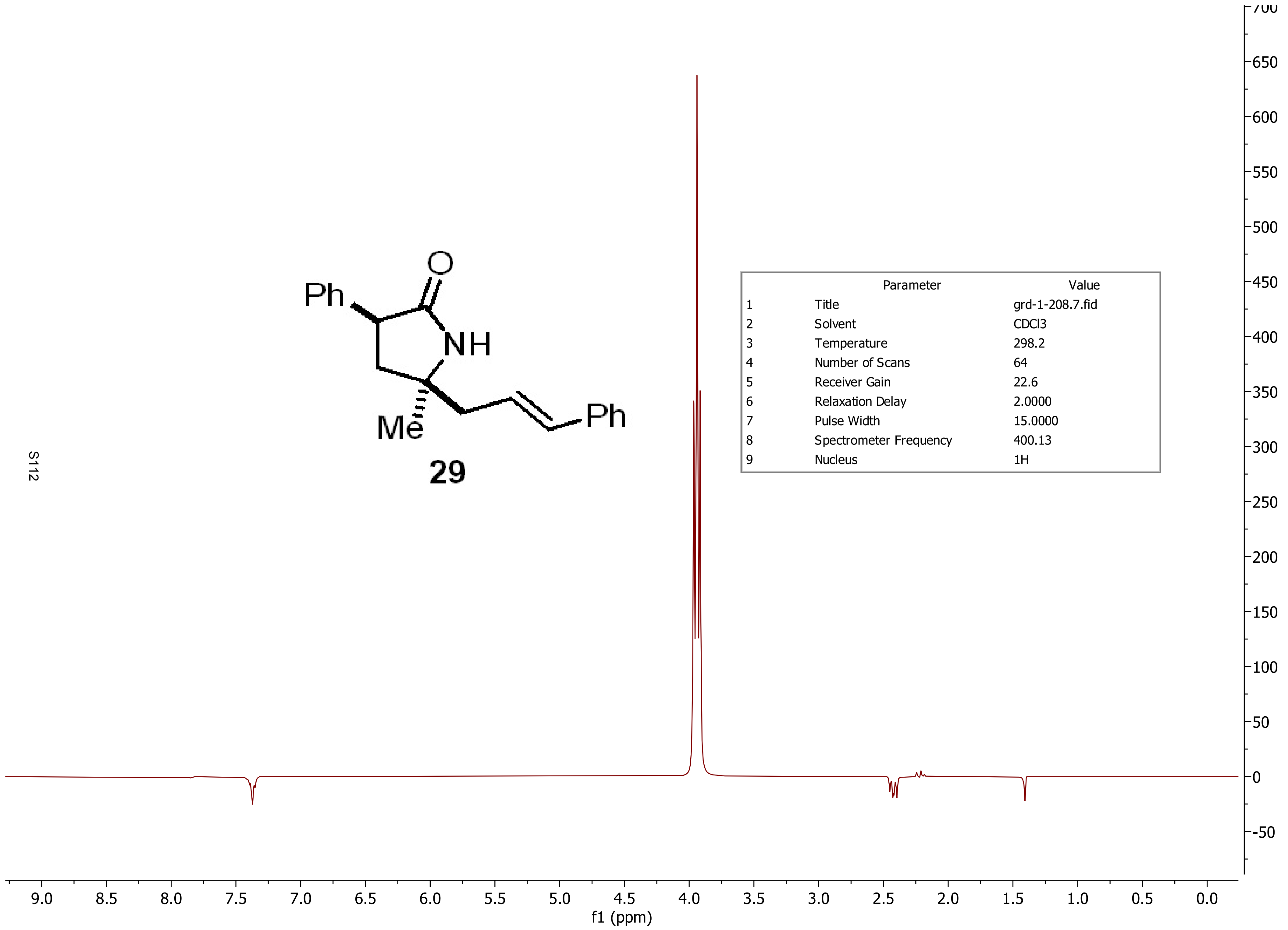

29

	Parameter	Value
1	Title	grd-1-208.7.fid
2	Solvent	CDCl3
3	Temperature	298.2
4	Number of Scans	64
5	Receiver Gain	22.6
6	Relaxation Delay	2.0000
7	Pulse Width	15.0000
8	Spectrometer Frequency	400.13
9	Nucleus	1H

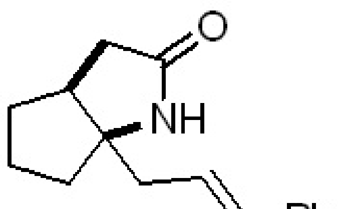

30

	Parameter	Value
1	Title	grd-2-135.3.1
2	Solvent	CDCl3
3	Temperature	298.0
4	Number of Scans	16
5	Receiver Gain	18.0
6	Relaxation Delay	1.0000
7	Pulse Width	10.5000
8	Spectrometer Frequency	600.32
9	Nucleus	1H

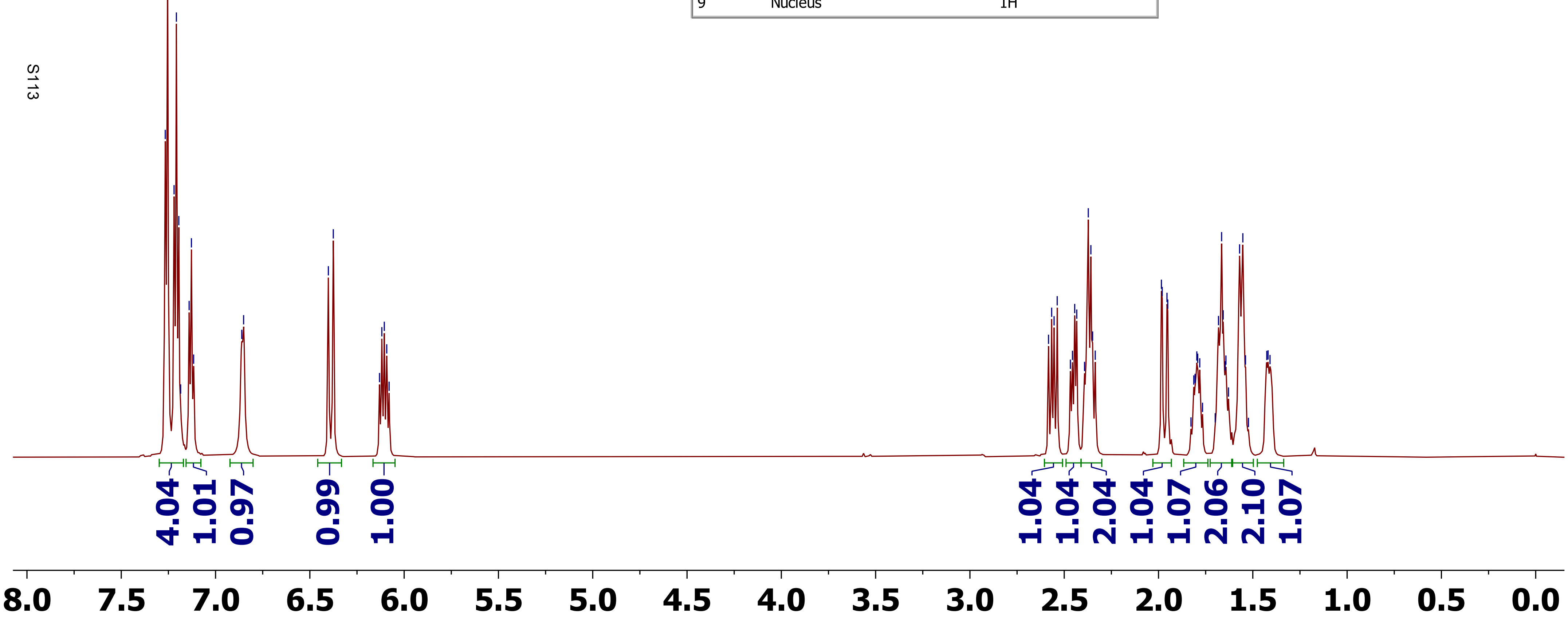

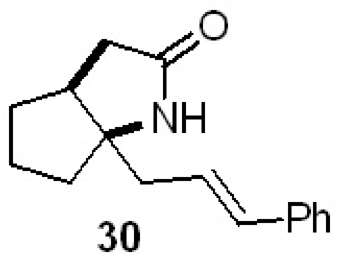

30

	Parameter	Value
1	Title	grd-2-135.4.1
2	Solvent	CDCl3
3	Temperature	298.0
4	Number of Scans	256
5	Receiver Gain	2050.0
6	Relaxation Delay	5.0000
7	Pulse Width	10.6300
8	Spectrometer Frequency	150.95
9	Nucleus	13C

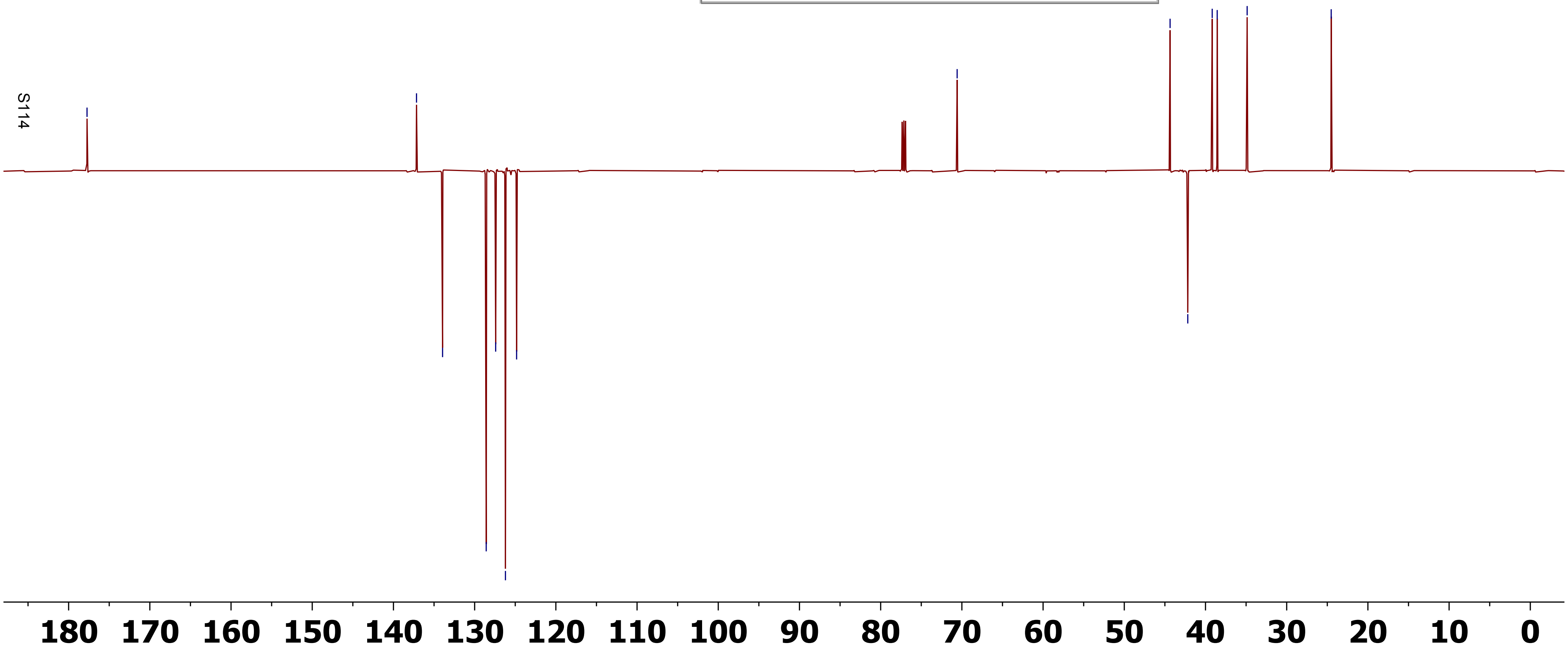

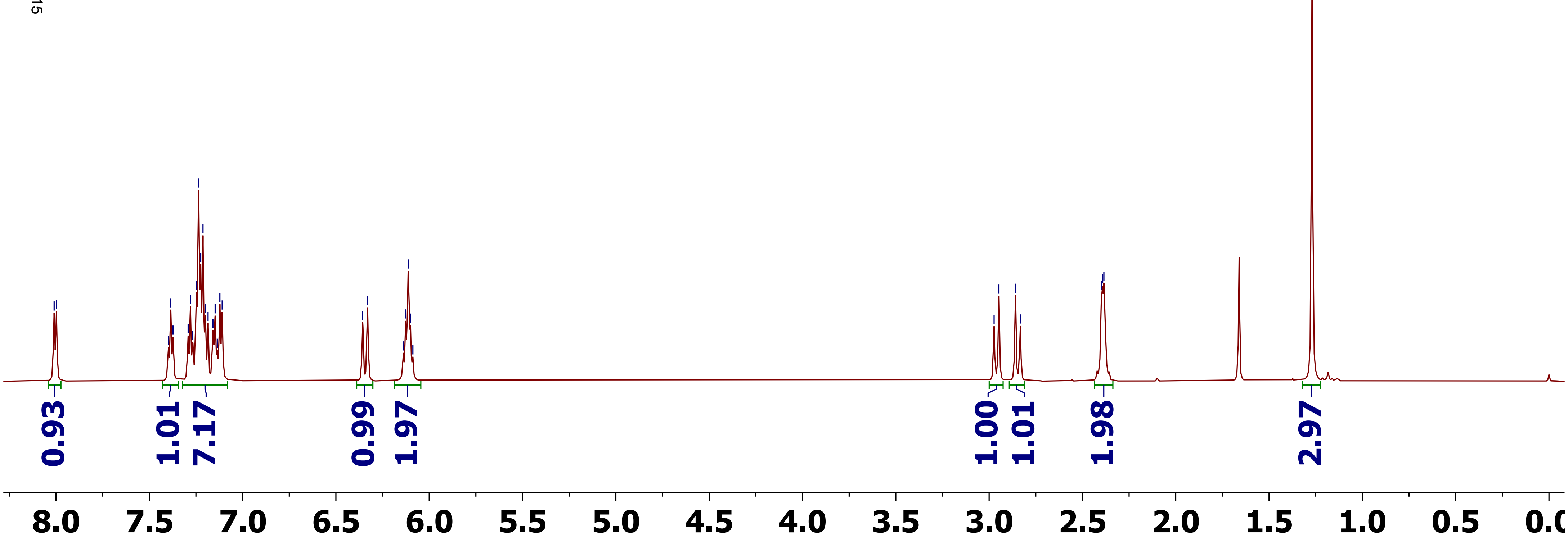

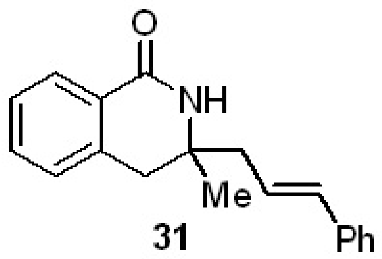

31

	Parameter	Value
1	Title	grd-1-238.3.fid
2	Solvent	CDCl3
3	Temperature	298.0
4	Number of Scans	16
5	Receiver Gain	90.5
6	Relaxation Delay	1.0000
7	Pulse Width	10.5000
8	Spectrometer Frequency	600.32
9	Nucleus	1H

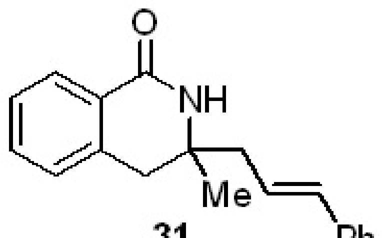

31

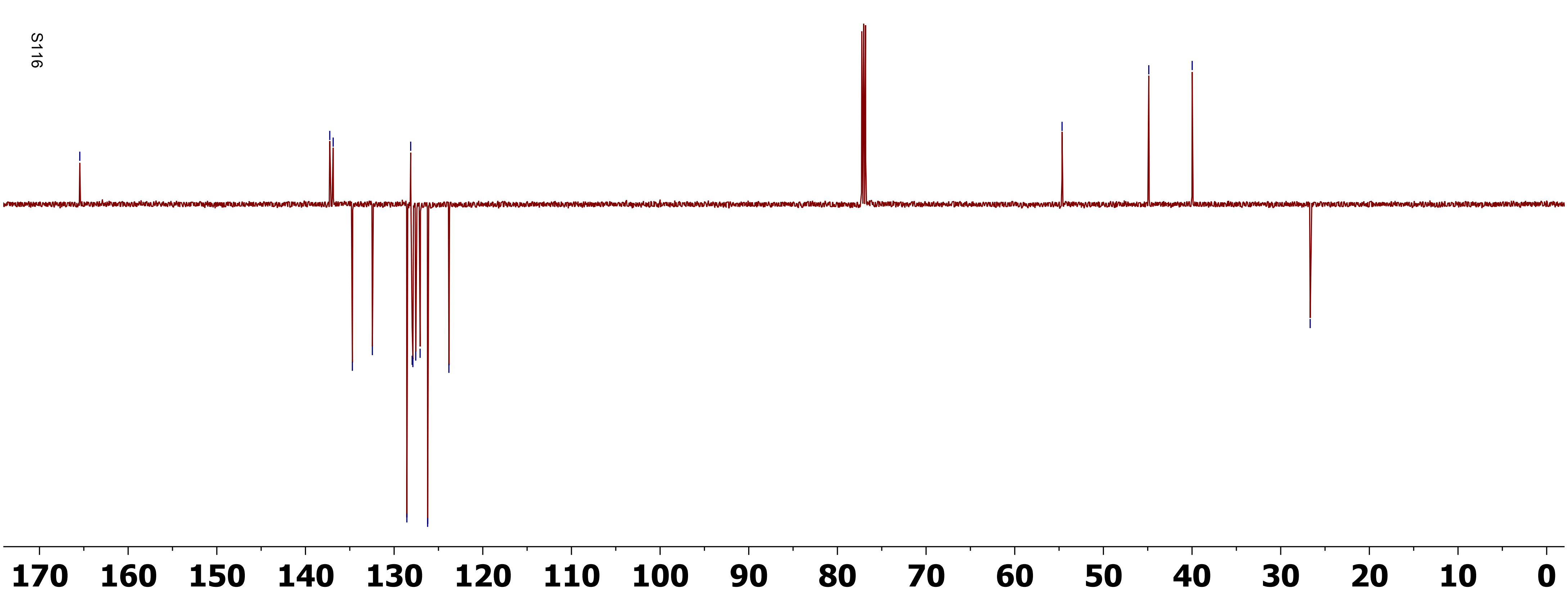

	Parameter	Value
1	Title	grd-1-238.4.fid
2	Solvent	CDCl3
3	Temperature	298.0
4	Number of Scans	256
5	Receiver Gain	2050.0
6	Relaxation Delay	5.0000
7	Pulse Width	10.6300
8	Spectrometer Frequency	150.97
9	Nucleus	13C

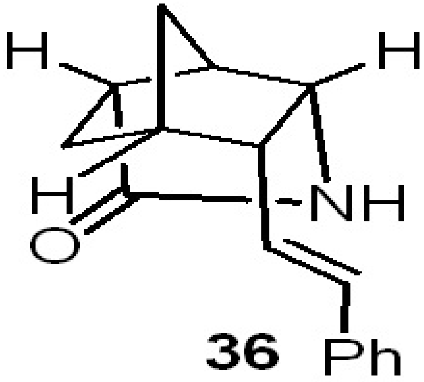

36

Parameter		Value
1	Title	grd-2-4.2.fid
2	Solvent	CDCl3
3	Temperature	300.0
4	Number of Scans	16
5	Receiver Gain	18.0
6	Relaxation Delay	1.0000
7	Pulse Width	10.6100
8	Spectrometer Frequency	600.32
9	Nucleus	1H

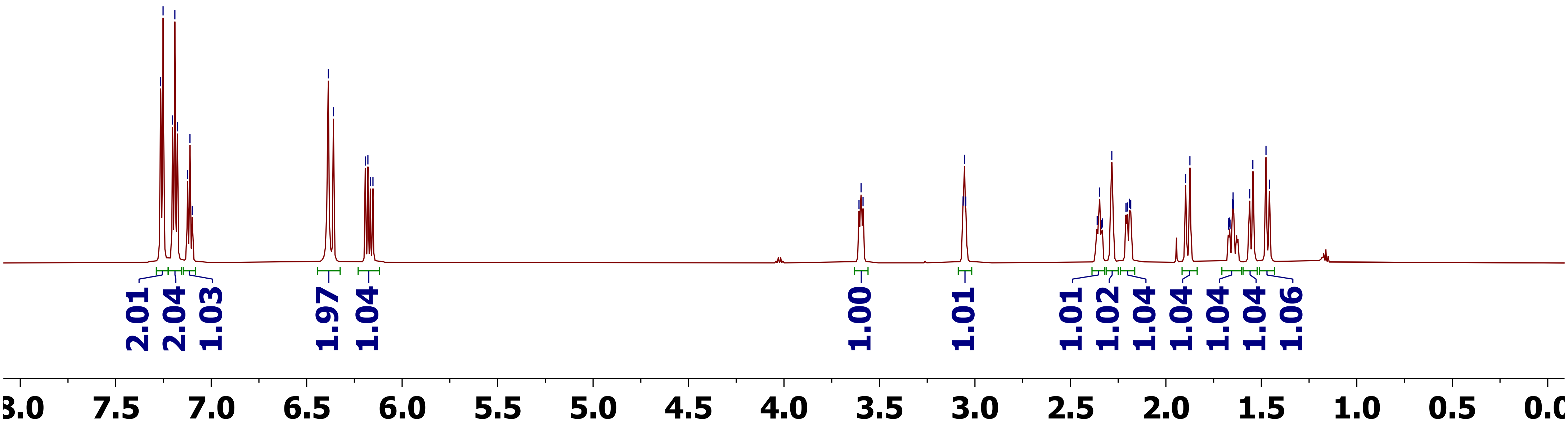

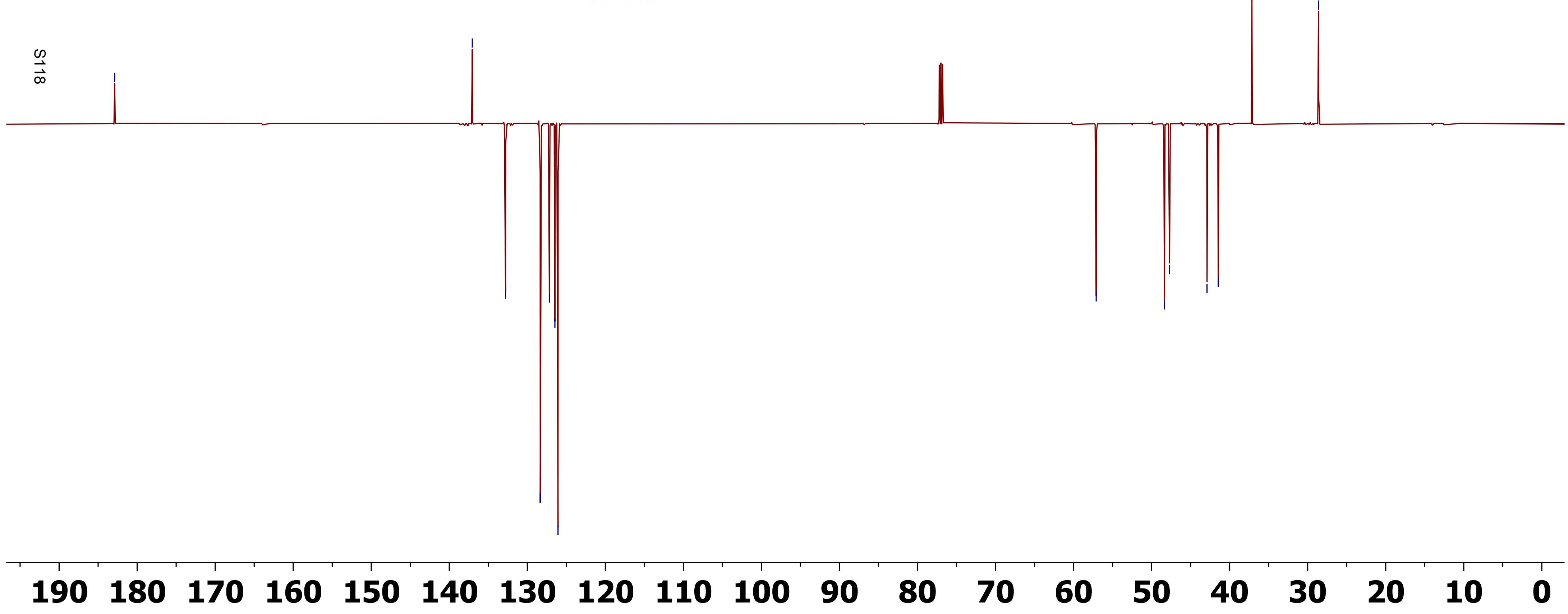

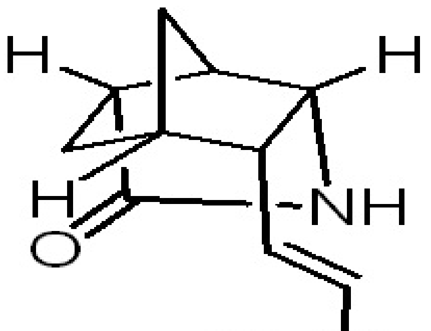

36

	Parameter	Value
1	Title	grd-2-4.3.fid
2	Solvent	CDCl3
3	Temperature	300.0
4	Number of Scans	256
5	Receiver Gain	2050.0
6	Relaxation Delay	5.0000
7	Pulse Width	15.0000
8	Spectrometer Frequency	150.97
9	Nucleus	13C

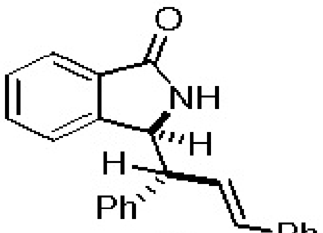

41

	Parameter	Value
1	Title	grd-1-240.2.fid
2	Solvent	CDCl3
3	Temperature	298.0
4	Number of Scans	16
5	Receiver Gain	57.0
6	Relaxation Delay	1.0000
7	Pulse Width	10.5000
8	Spectrometer Frequency	600.32
9	Nucleus	1H

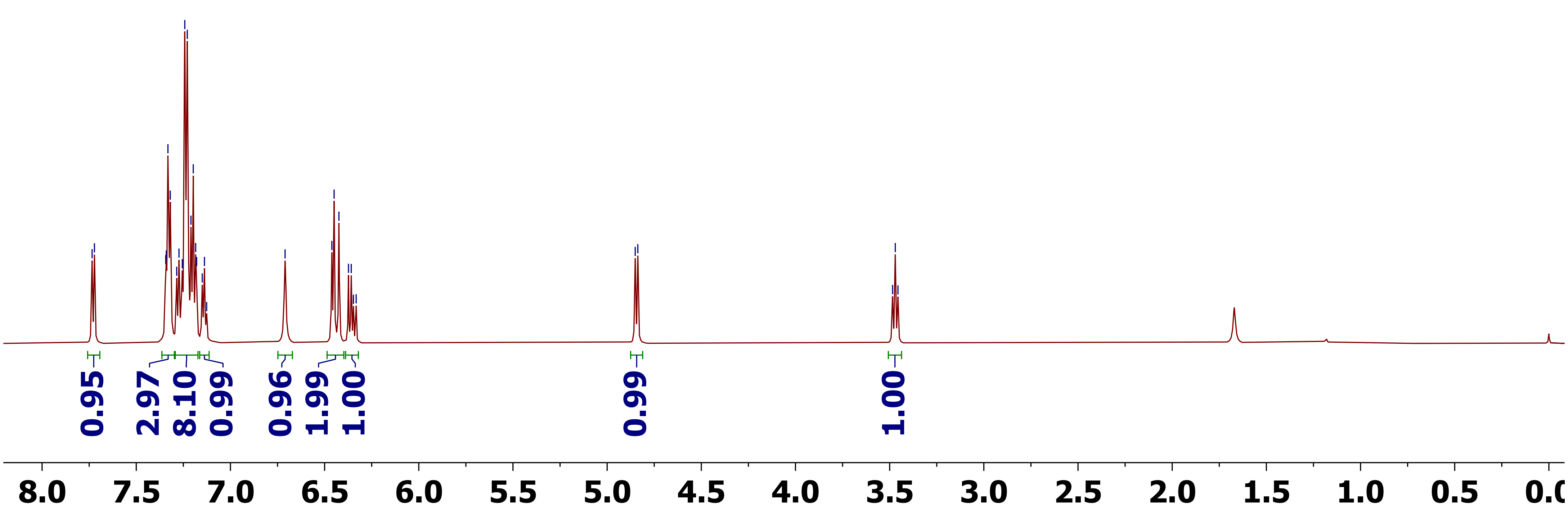

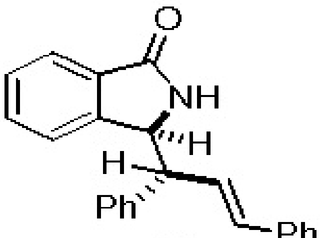

41

	Parameter	Value
1	Title	grd-1-240.3.fid
2	Solvent	CDCl3
3	Temperature	298.0
4	Number of Scans	256
5	Receiver Gain	2050.0
6	Relaxation Delay	5.0000
7	Pulse Width	10.6300
8	Spectrometer Frequency	150.97
9	Nucleus	13C

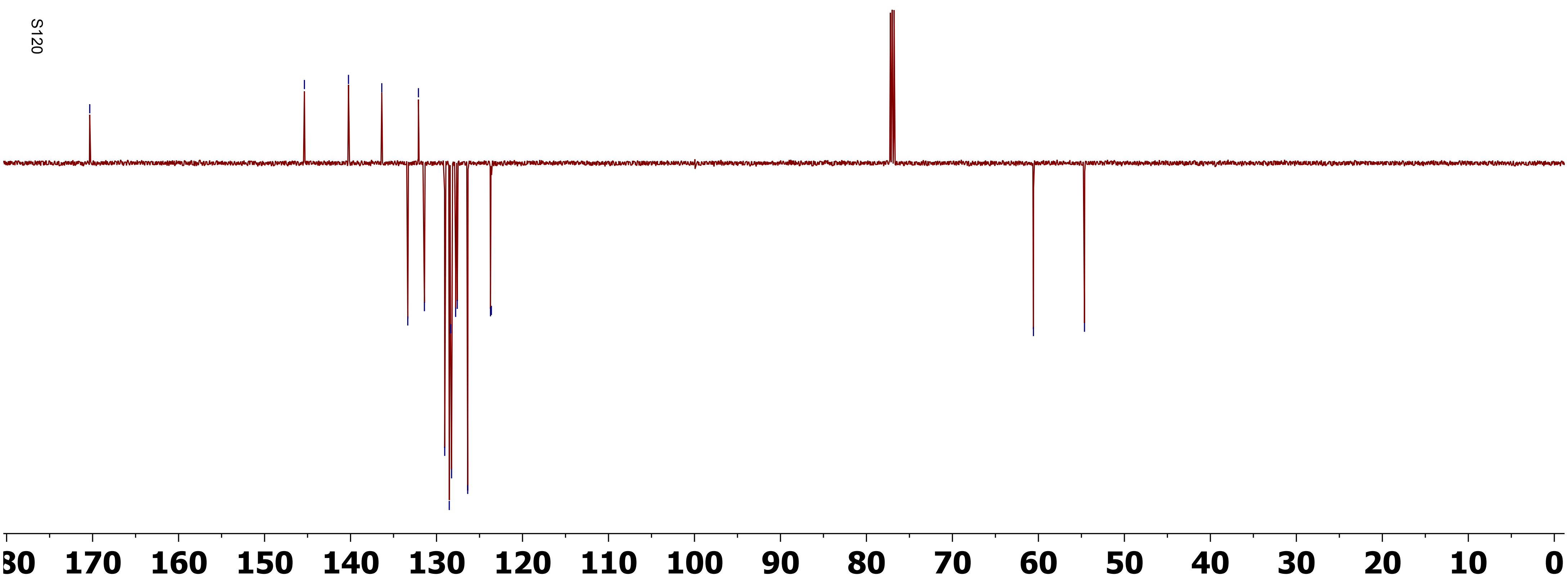

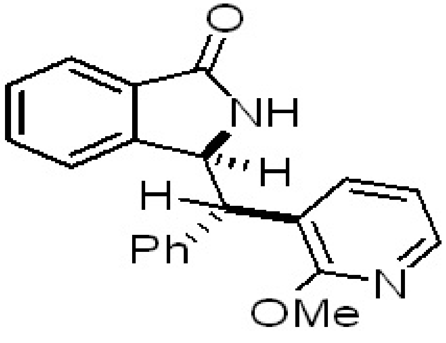

42

	Parameter	Value
1	Title	grd-2-167.5.1
2	Solvent	CDCl3
3	Temperature	298.0
4	Number of Scans	16
5	Receiver Gain	40.3
6	Relaxation Delay	1.0000
7	Pulse Width	10.5000
8	Spectrometer Frequency	600.32
9	Nucleus	1H

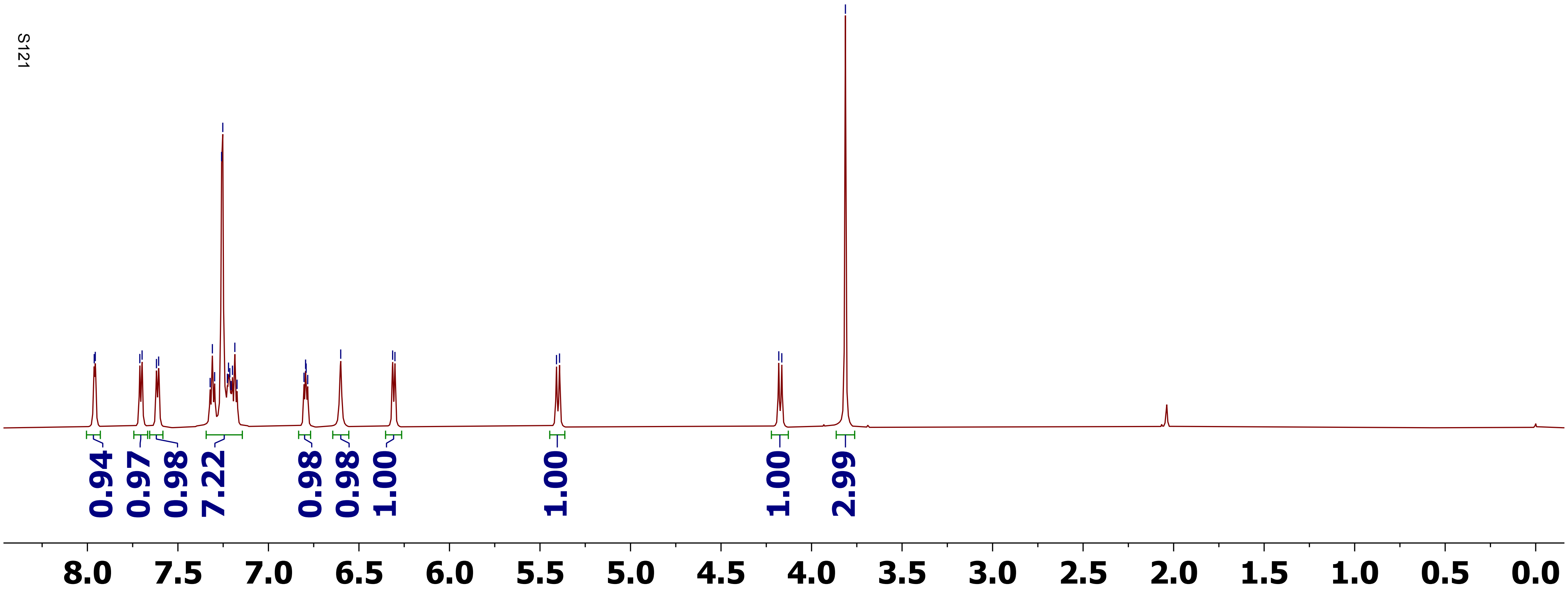

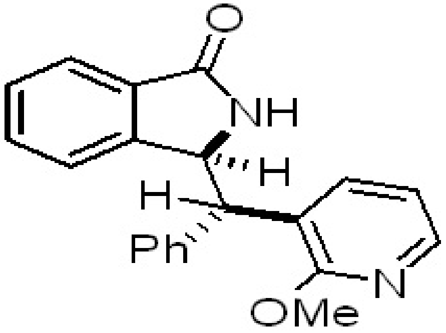

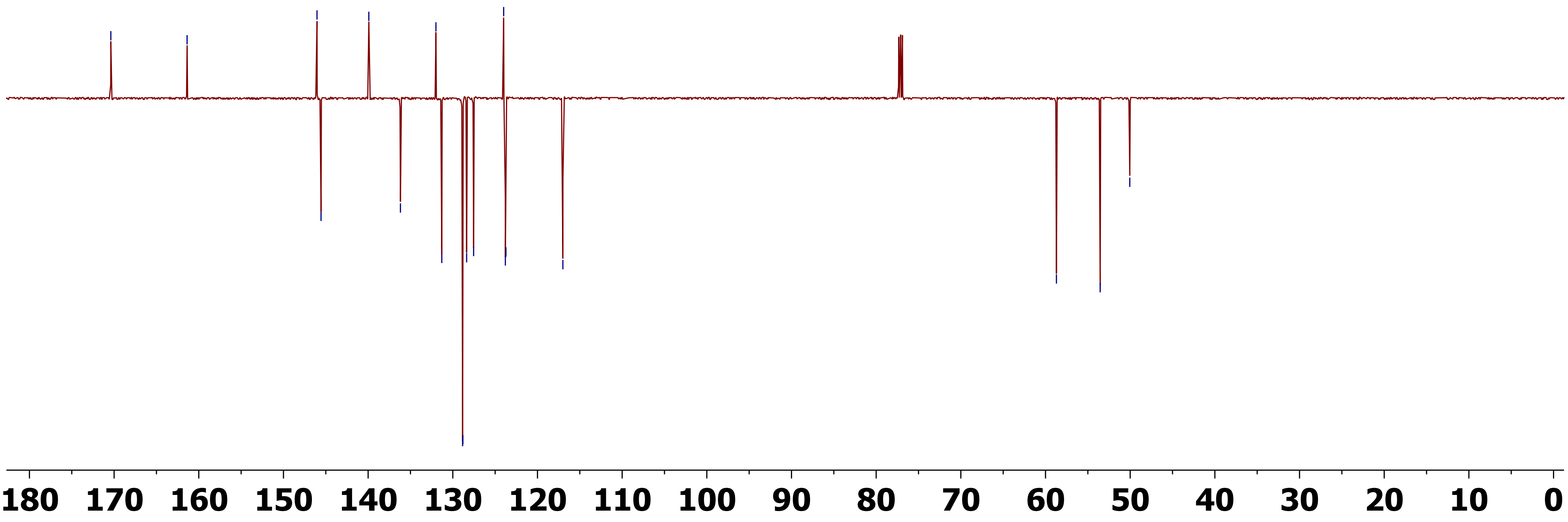

42

	Parameter	Value
1	Title	grd-2-167.6.1
2	Solvent	CDCl3
3	Temperature	298.0
4	Number of Scans	256
5	Receiver Gain	2050.0
6	Relaxation Delay	5.0000
7	Pulse Width	10.6300
8	Spectrometer Frequency	150.95
9	Nucleus	13C

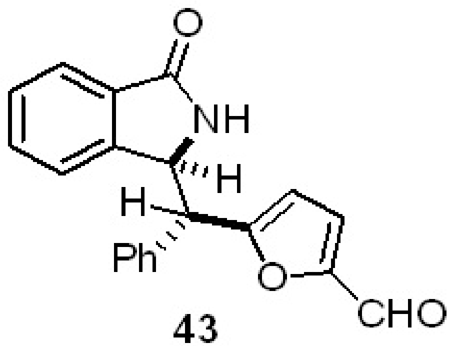

43

	Parameter	Value
1	Title	grd-2-172.3.1
2	Solvent	CDCl3
3	Temperature	298.0
4	Number of Scans	16
5	Receiver Gain	57.0
6	Relaxation Delay	1.0000
7	Pulse Width	10.5000
8	Spectrometer Frequency	600.32
9	Nucleus	1H

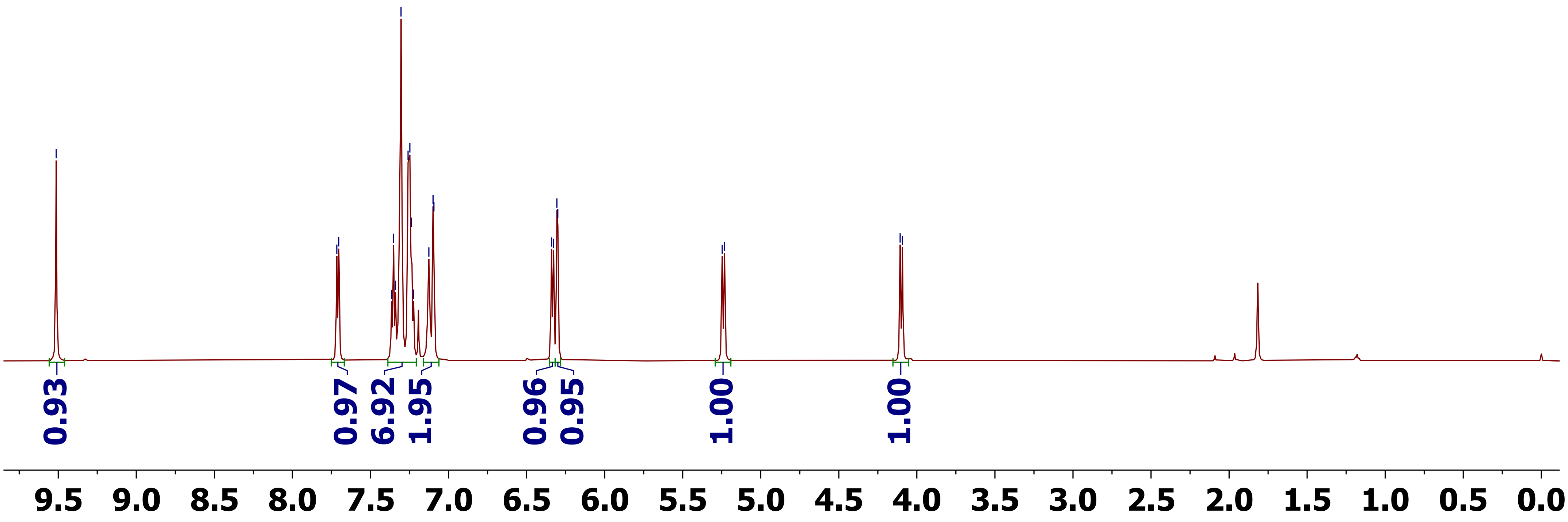

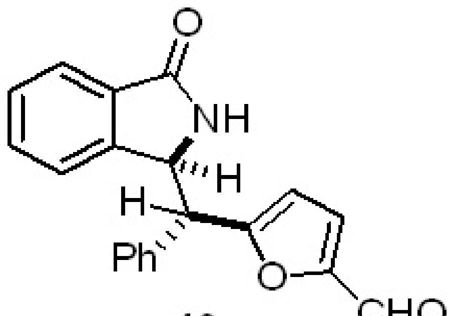

43

	Parameter	Value
1	Title	grd-2-172.2.1
2	Solvent	CDCl3
3	Temperature	298.0
4	Number of Scans	256
5	Receiver Gain	2050.0
6	Relaxation Delay	5.0000
7	Pulse Width	10.6300
8	Spectrometer Frequency	150.95
9	Nucleus	13C

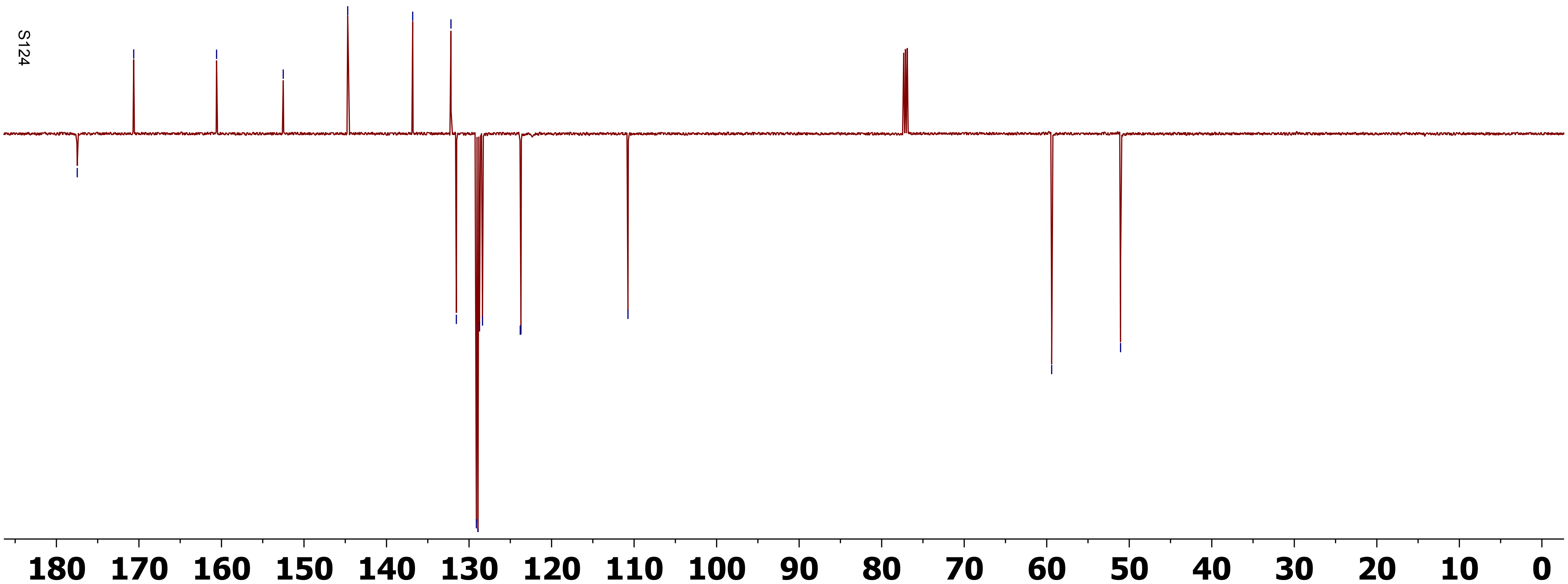

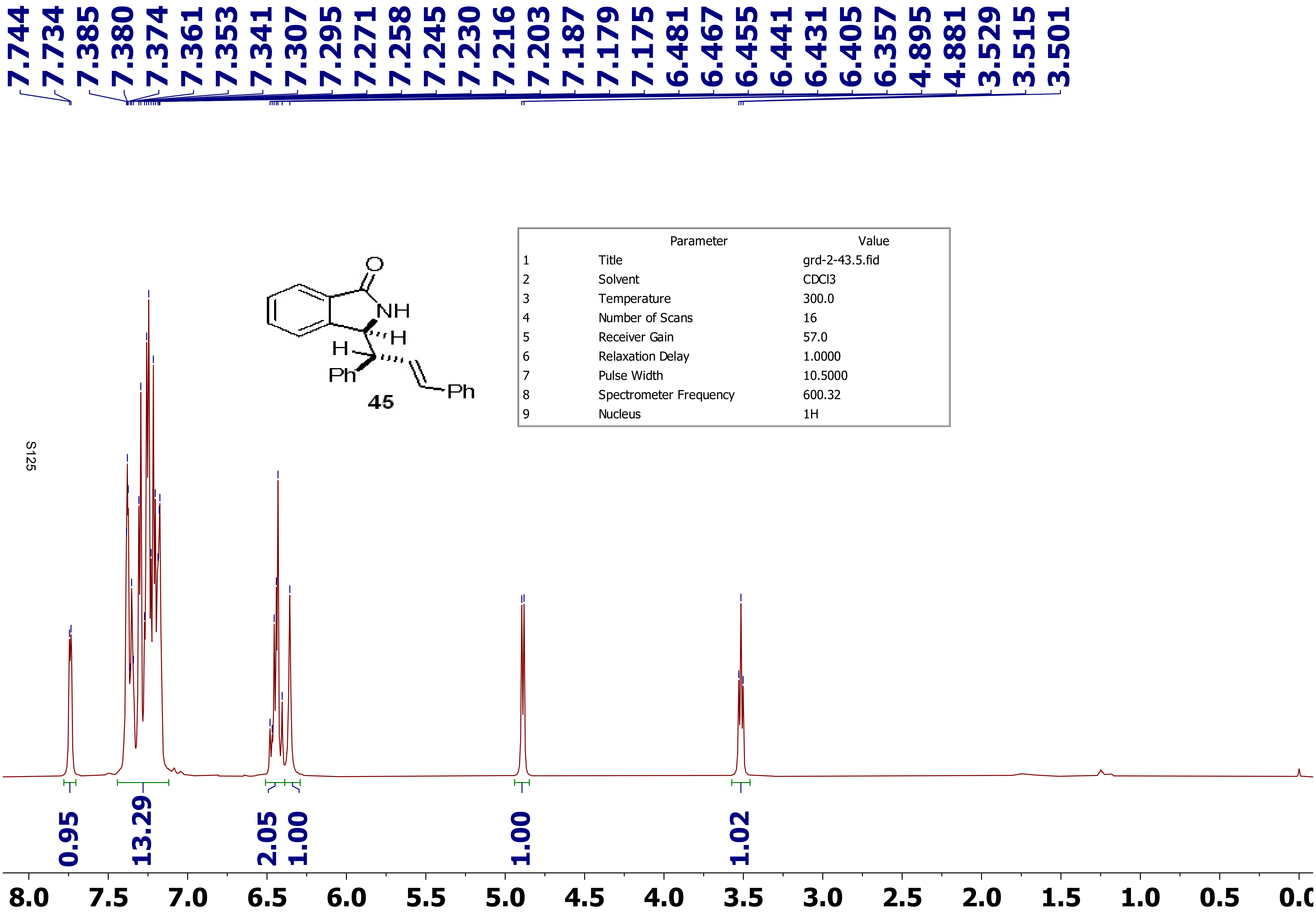

	Parameter	Value
1	Title	grd-2-43.5.fid
2	Solvent	CDCl3
3	Temperature	300.0
4	Number of Scans	16
5	Receiver Gain	57.0
6	Relaxation Delay	1.0000
7	Pulse Width	10.5000
8	Spectrometer Frequency	600.32
9	Nucleus	1H

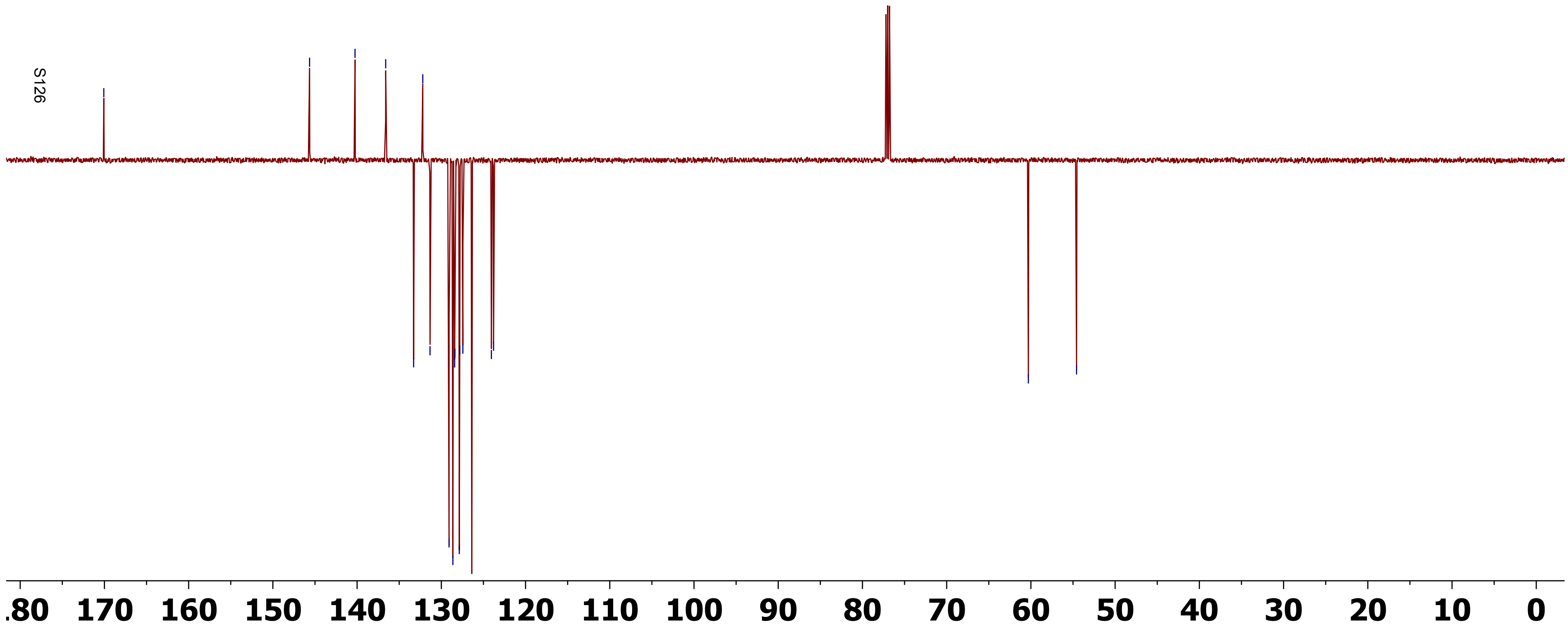

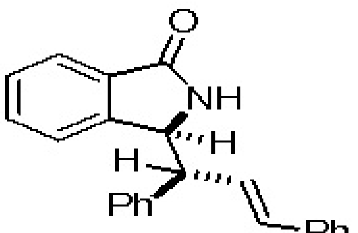

45

	Parameter	Value
1	Title	grd-2-43.6.fid
2	Solvent	CDCl3
3	Temperature	300.0
4	Number of Scans	256
5	Receiver Gain	2050.0
6	Relaxation Delay	5.0000
7	Pulse Width	10.6300
8	Spectrometer Frequency	150.97
9	Nucleus	13C

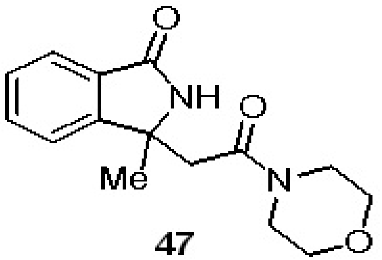

47

	Parameter	Value
1	Title	grd-2-266-pure.1.fid
2	Solvent	CDCl3
3	Temperature	300.0
4	Number of Scans	16
5	Receiver Gain	18.0
6	Relaxation Delay	1.0000
7	Pulse Width	10.5000
8	Spectrometer Frequency	600.32
9	Nucleus	1H

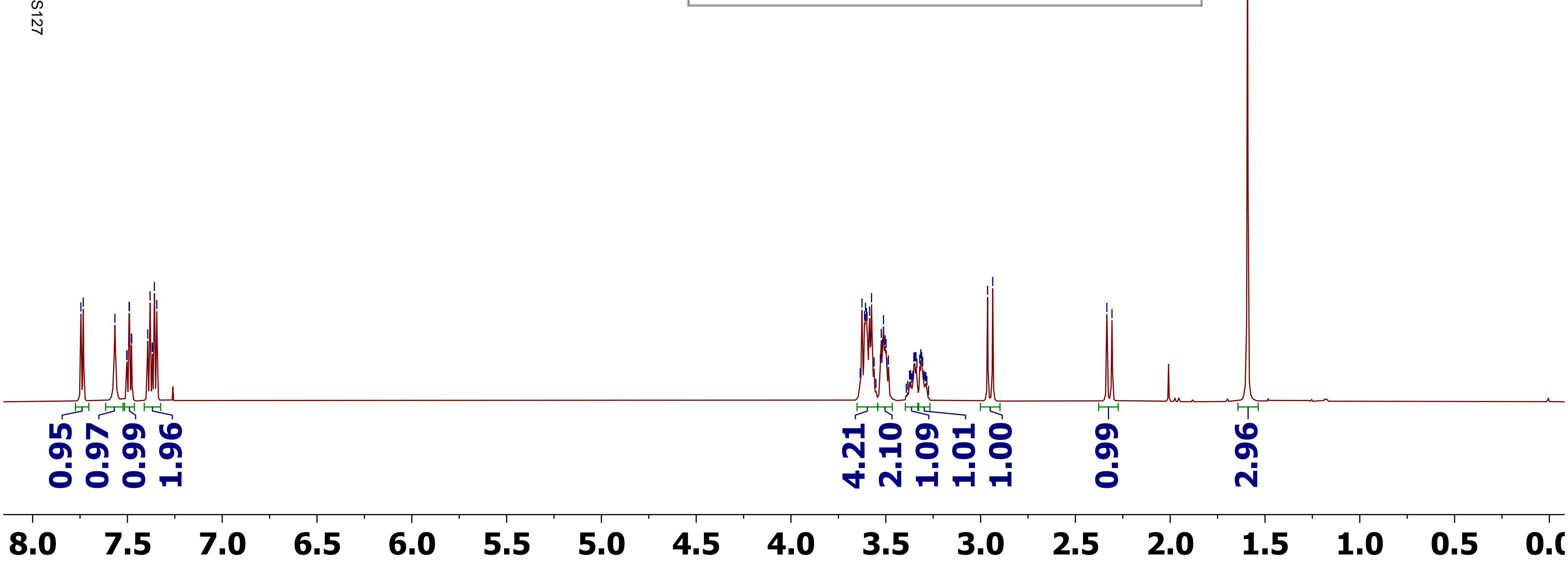

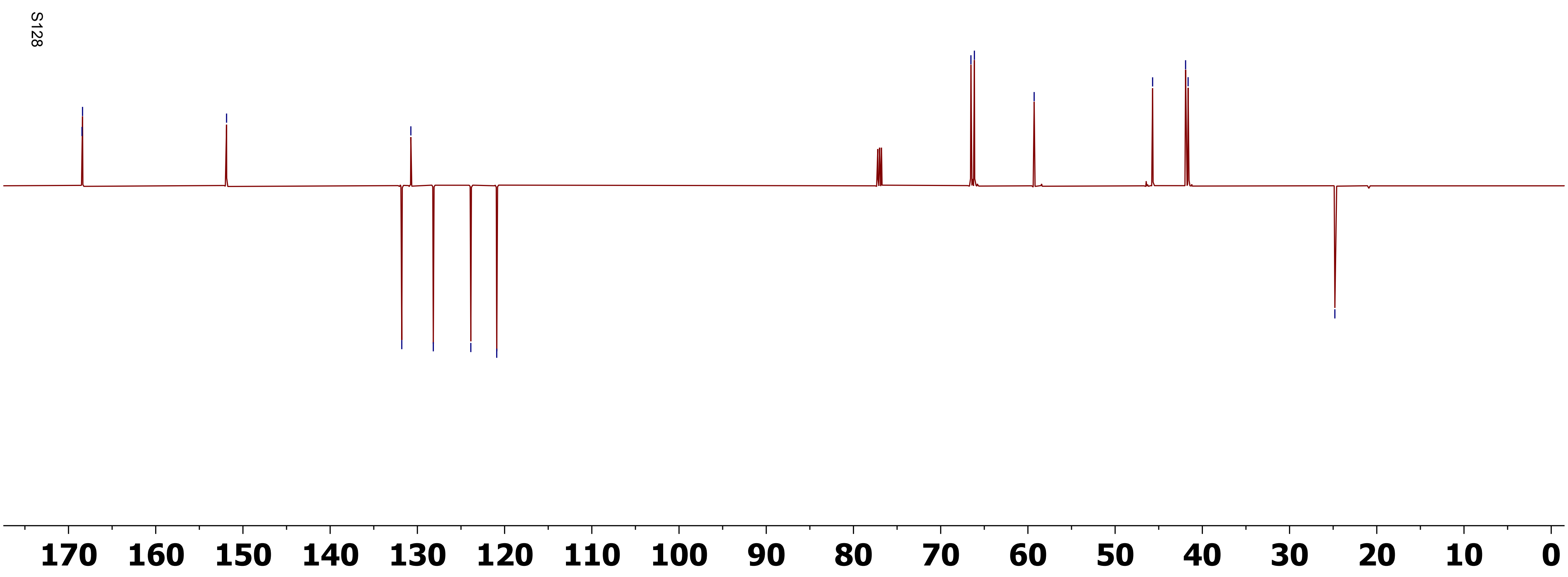

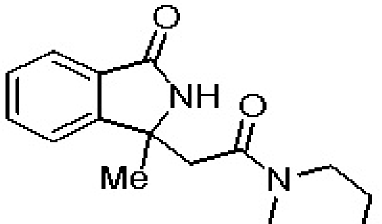

47

	Parameter	Value
1	Title	grd-2-266-pure.2.fid
2	Solvent	CDCl3
3	Temperature	300.0
4	Number of Scans	256
5	Receiver Gain	2050.0
6	Relaxation Delay	5.0000
7	Pulse Width	10.6300
8	Spectrometer Frequency	150.97
9	Nucleus	13C

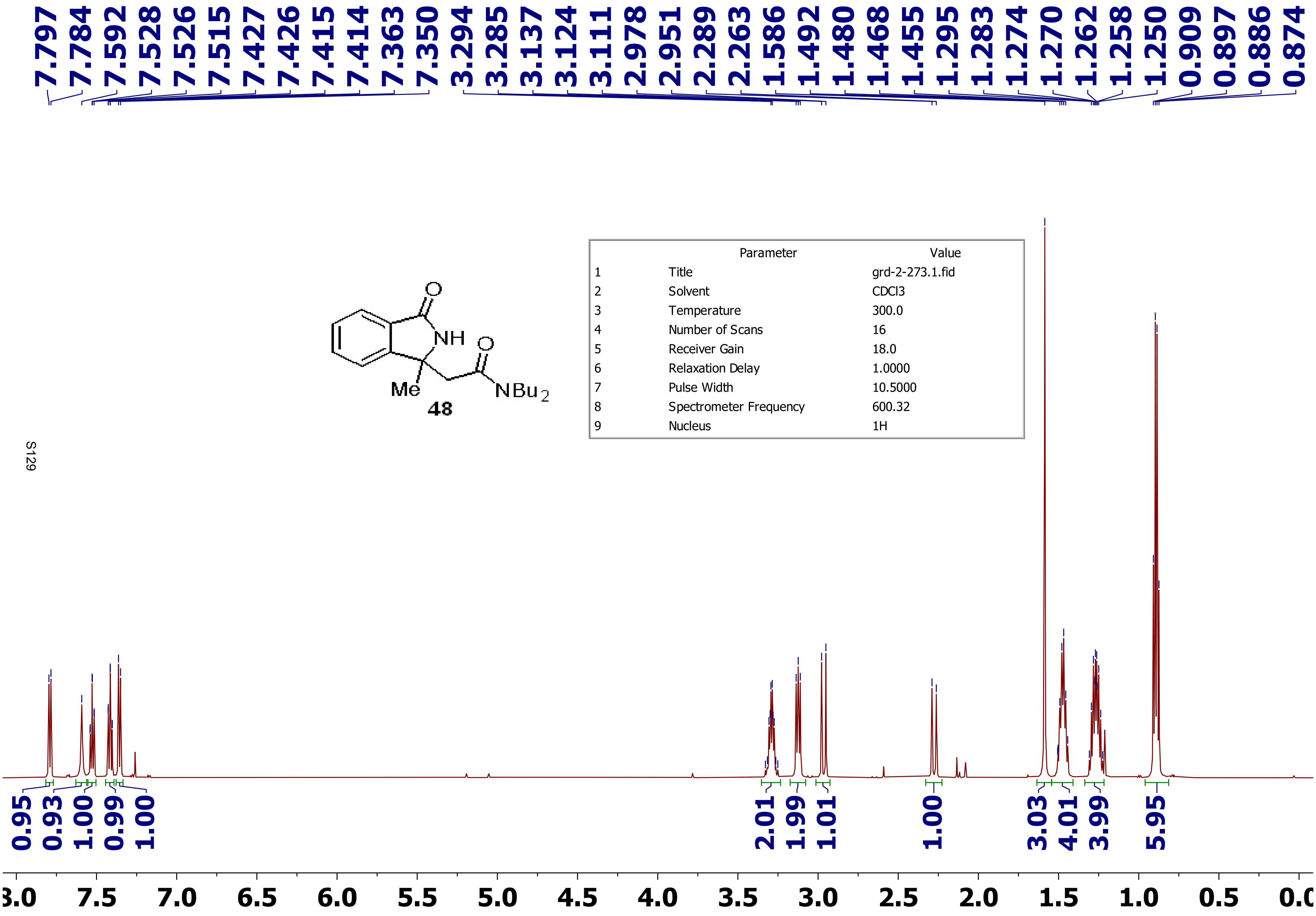

48

	Parameter	Value
1	Title	grd-2-273.1.fid
2	Solvent	CDCl3
3	Temperature	300.0
4	Number of Scans	16
5	Receiver Gain	18.0
6	Relaxation Delay	1.0000
7	Pulse Width	10.5000
8	Spectrometer Frequency	600.32
9	Nucleus	1H

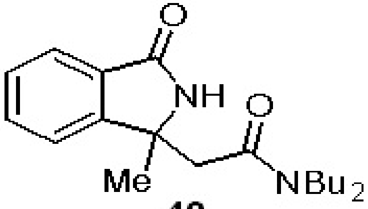

48

	Parameter	Value
1	Title	grd-2-273.2.fid
2	Solvent	CDCl3
3	Temperature	300.0
4	Number of Scans	256
5	Receiver Gain	2050.0
6	Relaxation Delay	5.0000
7	Pulse Width	10.6300
8	Spectrometer Frequency	150.97
9	Nucleus	13C

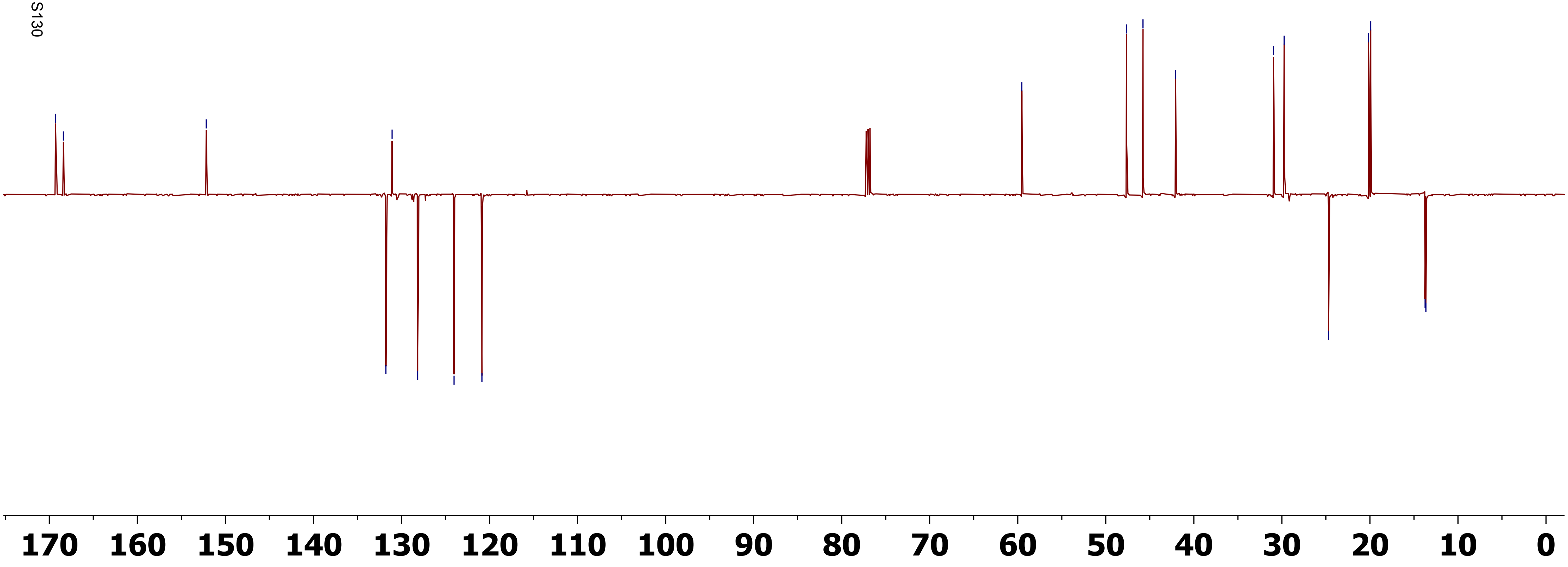

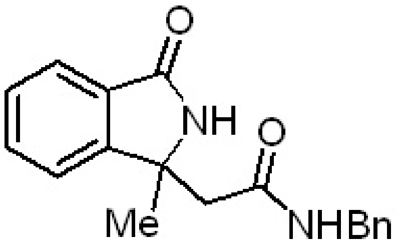

49

	Parameter	Value
1	Title	grd-2-272.5.fid
2	Solvent	CDCl3
3	Temperature	300.0
4	Number of Scans	16
5	Receiver Gain	57.0
6	Relaxation Delay	1.0000
7	Pulse Width	10.5000
8	Spectrometer Frequency	600.32
9	Nucleus	1H

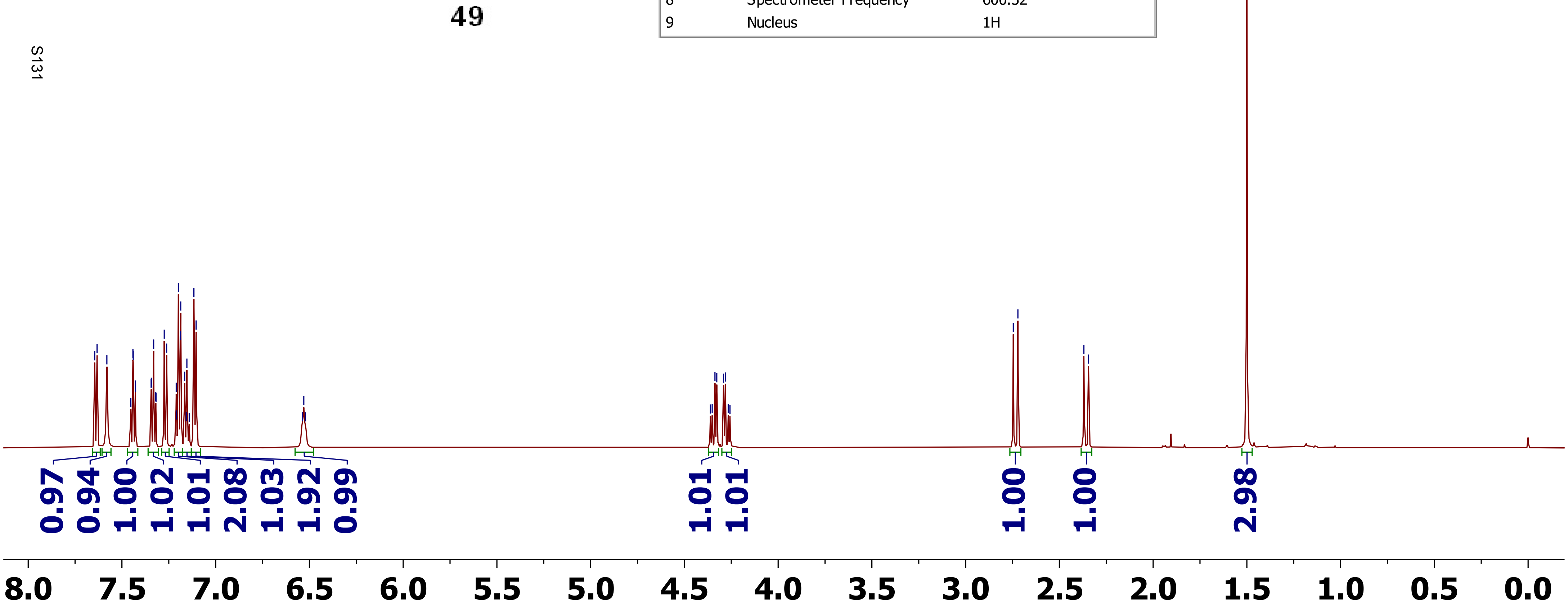

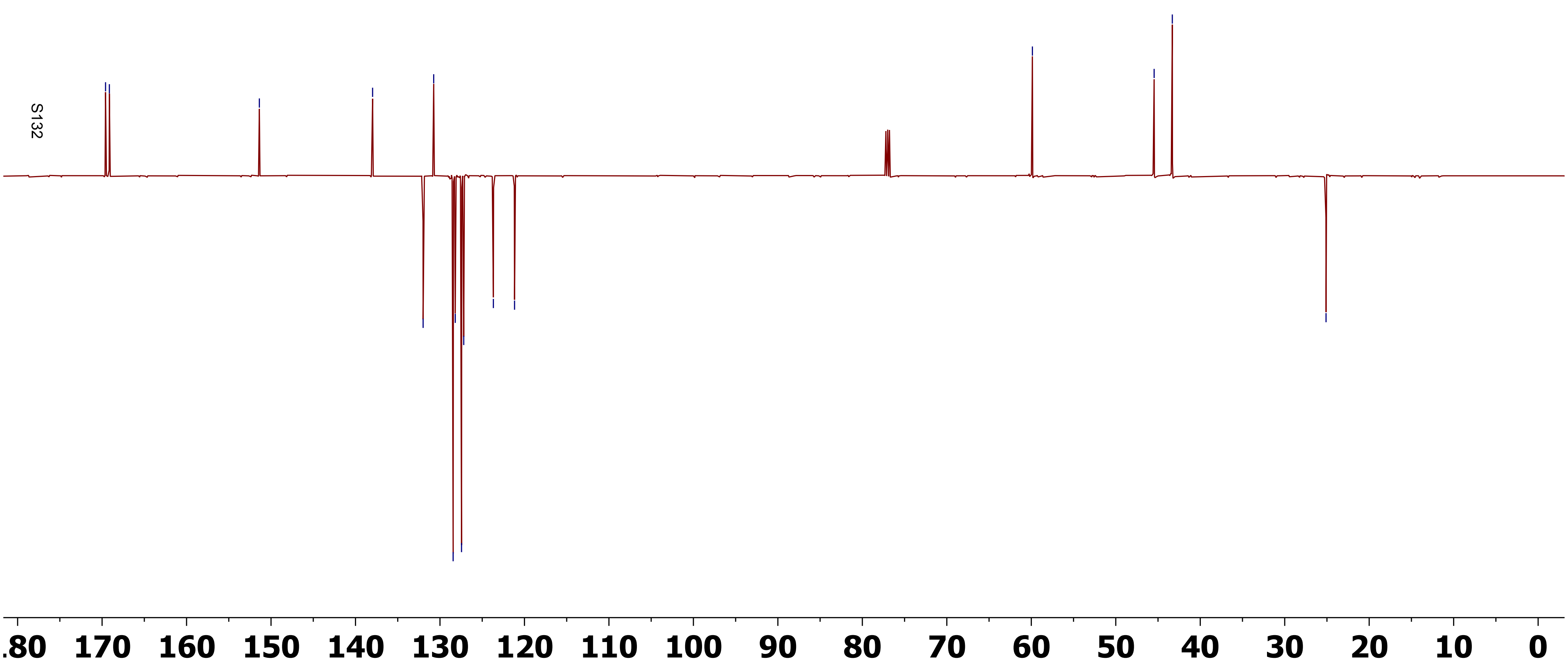

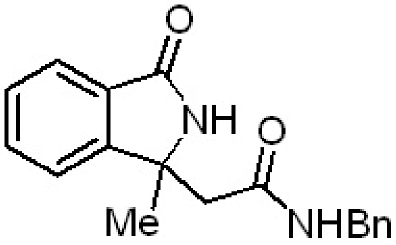

49

	Parameter	Value
1	Title	grd-2-272.4.fid
2	Solvent	CDCl3
3	Temperature	300.0
4	Number of Scans	256
5	Receiver Gain	2050.0
6	Relaxation Delay	5.0000
7	Pulse Width	10.6300
8	Spectrometer Frequency	150.97
9	Nucleus	13C

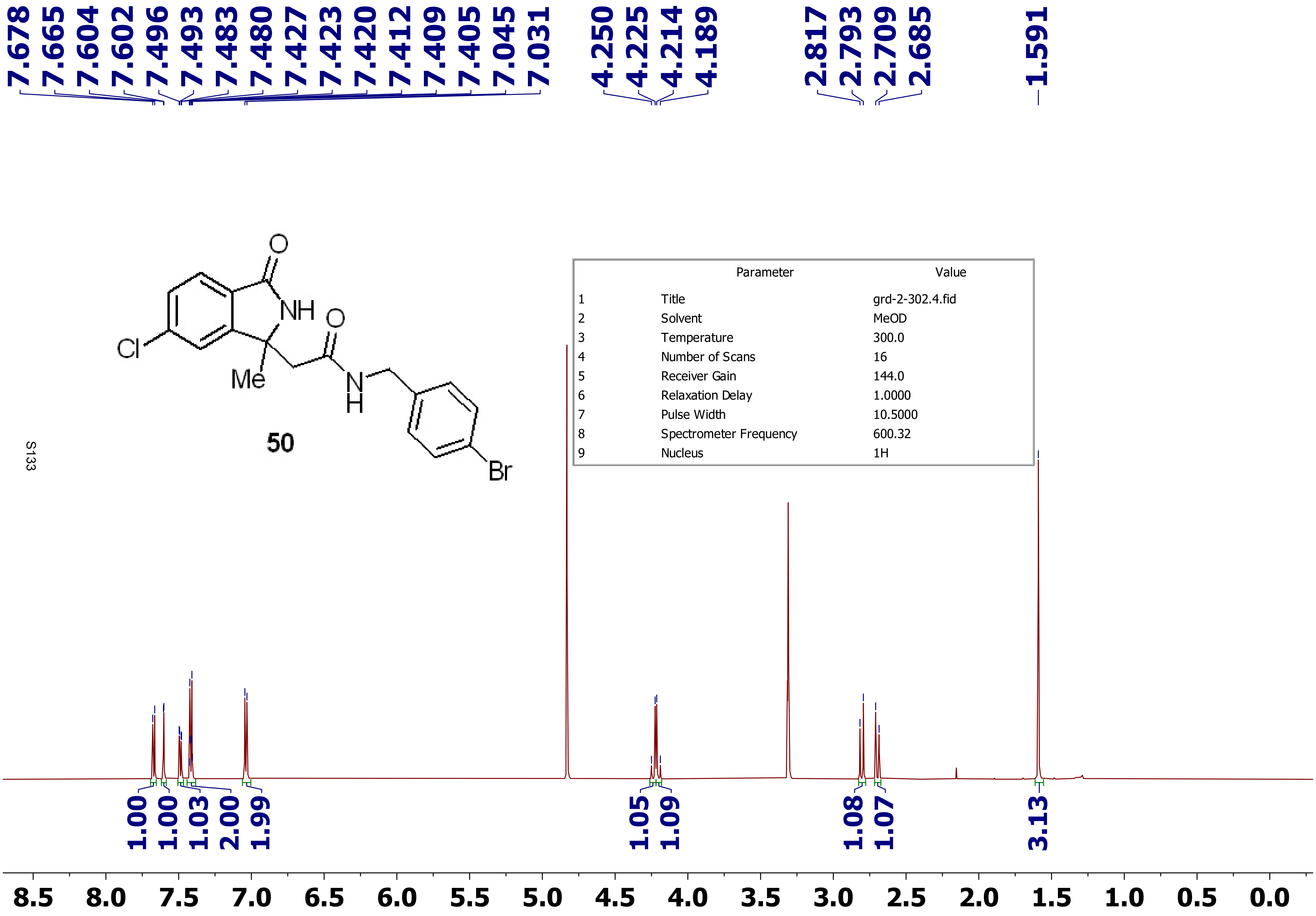

50

	Parameter	Value
1	Title	grd-2-302.4.fid
2	Solvent	MeOD
3	Temperature	300.0
4	Number of Scans	16
5	Receiver Gain	144.0
6	Relaxation Delay	1.0000
7	Pulse Width	10.5000
8	Spectrometer Frequency	600.32
9	Nucleus	1H

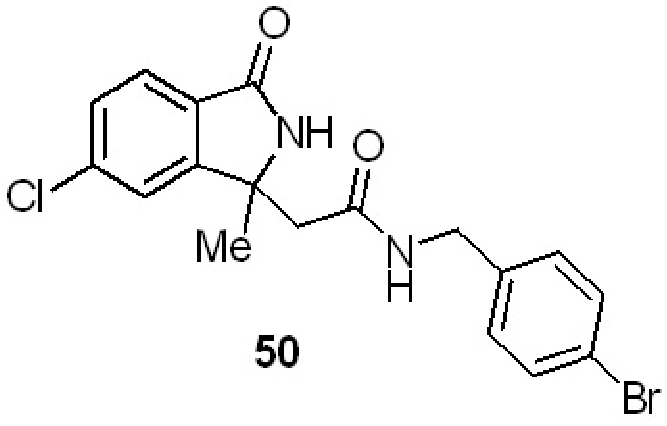

50

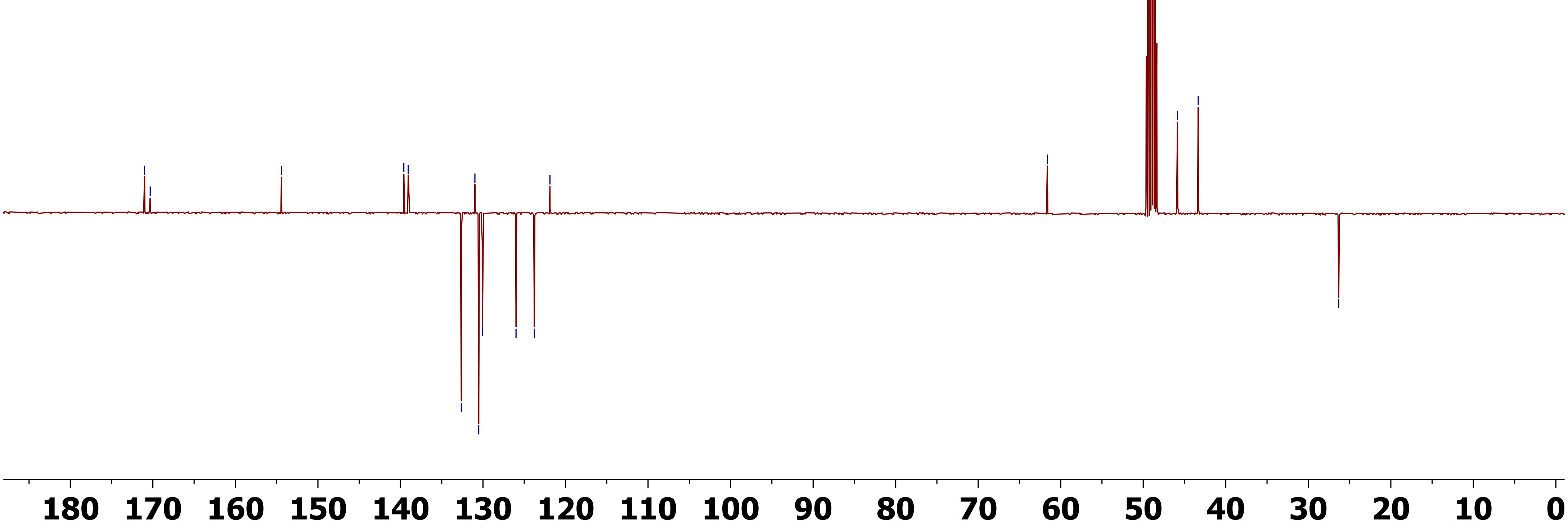

	Parameter	Value
1	Title	grd-2-302-pure.2.fid
2	Solvent	MeOD
3	Temperature	298.2
4	Number of Scans	1024
5	Receiver Gain	512.0
6	Relaxation Delay	3.0000
7	Pulse Width	10.7000
8	Spectrometer Frequency	100.62
9	Nucleus	13C

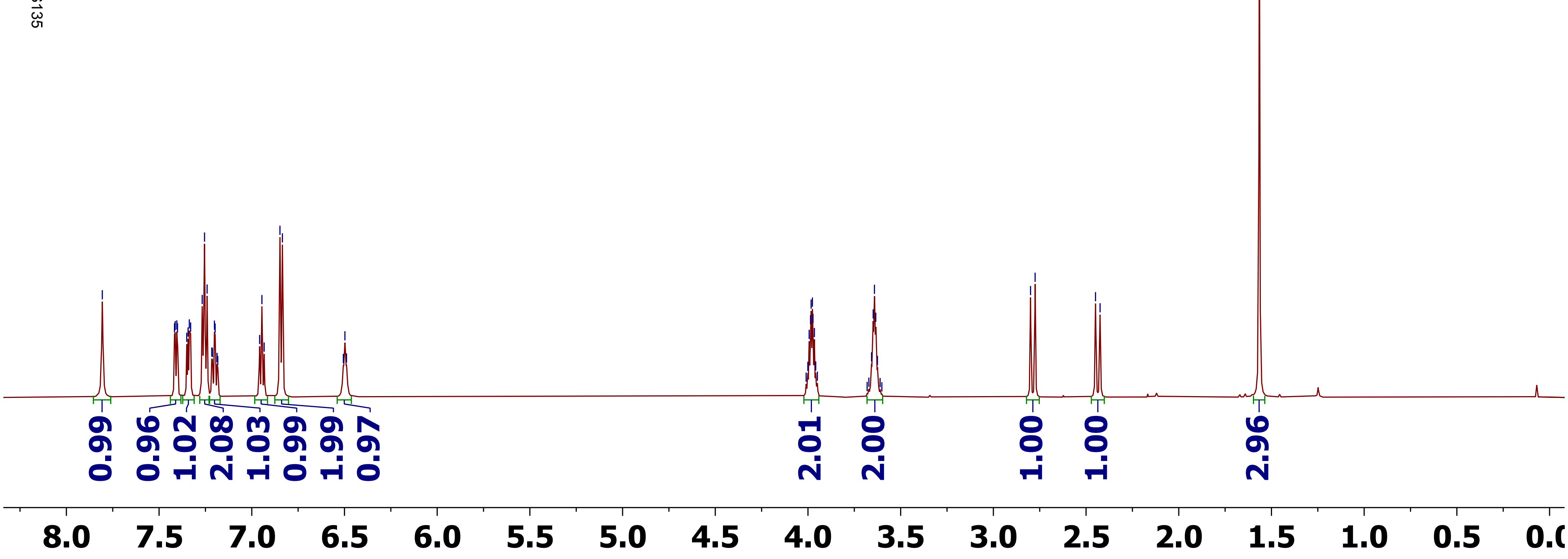

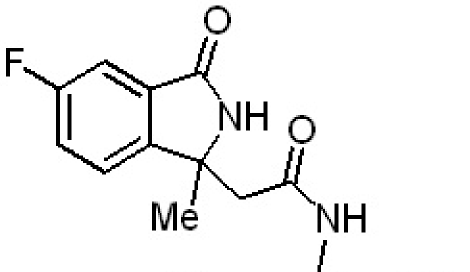

51

	Parameter	Value
1	Title	grd-2-298.5.fid
2	Solvent	CDCl3
3	Temperature	297.3
4	Number of Scans	16
5	Receiver Gain	57.0
6	Relaxation Delay	1.0000
7	Pulse Width	10.5000
8	Spectrometer Frequency	600.32
9	Nucleus	1H

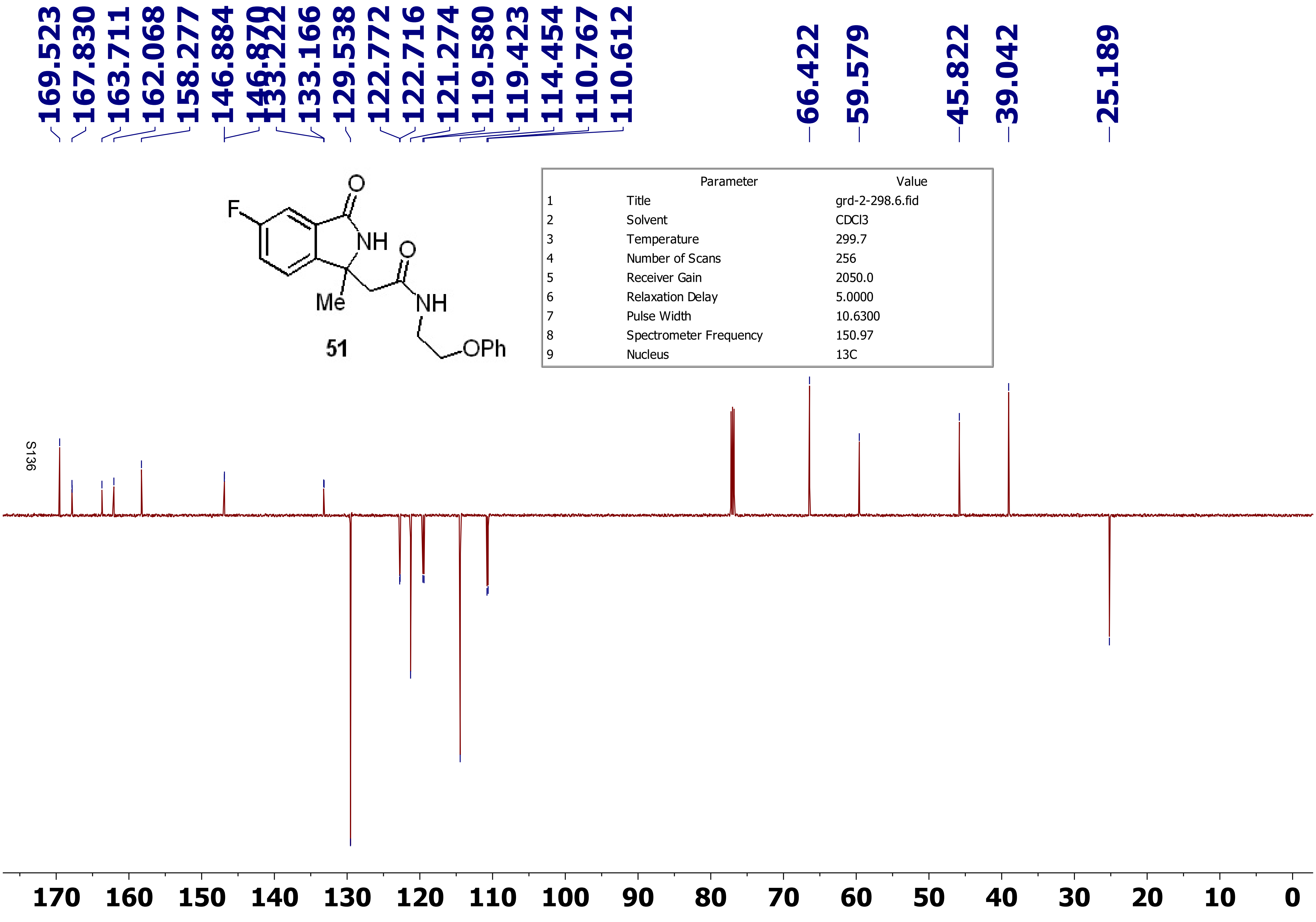

51

	Parameter	Value
1	Title	grd-2-298.6.fid
2	Solvent	CDCl3
3	Temperature	299.7
4	Number of Scans	256
5	Receiver Gain	2050.0
6	Relaxation Delay	5.0000
7	Pulse Width	10.6300
8	Spectrometer Frequency	150.97
9	Nucleus	13C

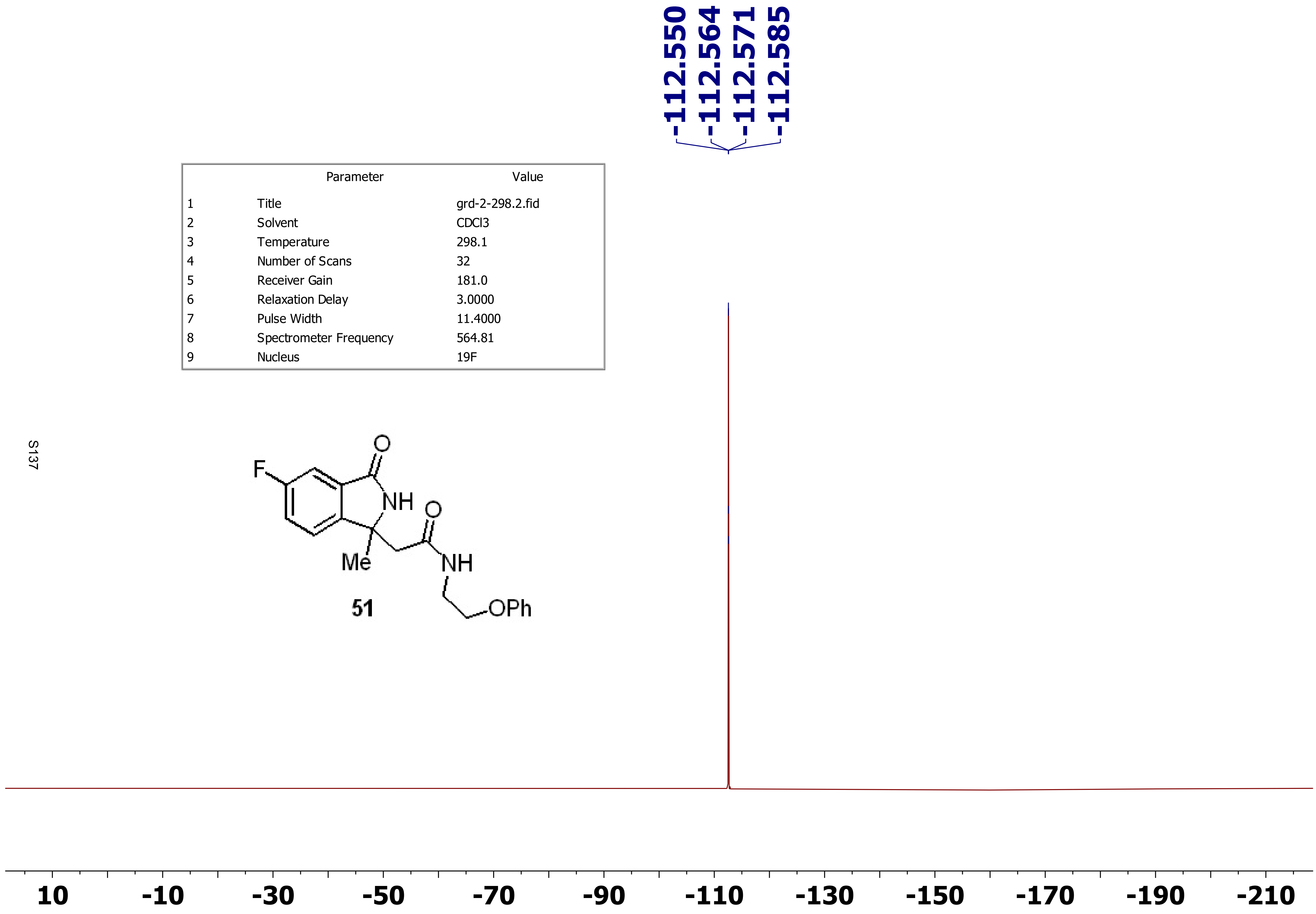

	Parameter	Value
1	Title	grd-2-298.2.fid
2	Solvent	CDCl3
3	Temperature	298.1
4	Number of Scans	32
5	Receiver Gain	181.0
6	Relaxation Delay	3.0000
7	Pulse Width	11.4000
8	Spectrometer Frequency	564.81
9	Nucleus	19F

51

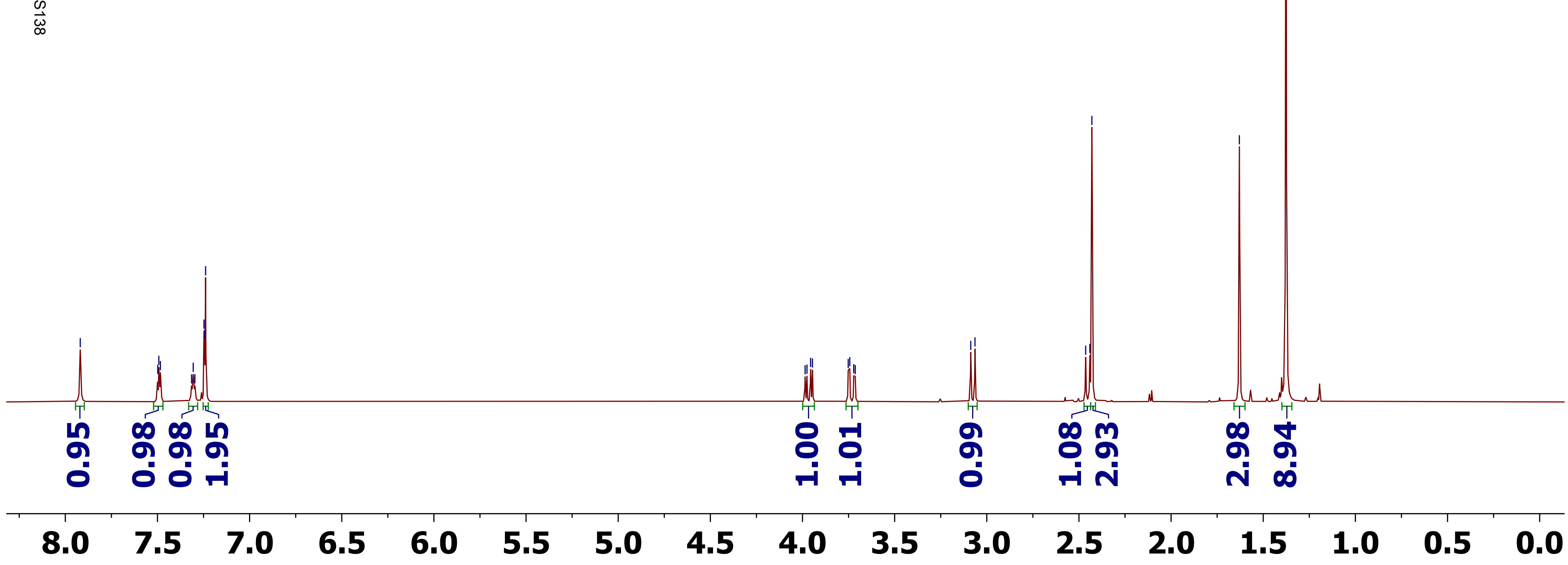

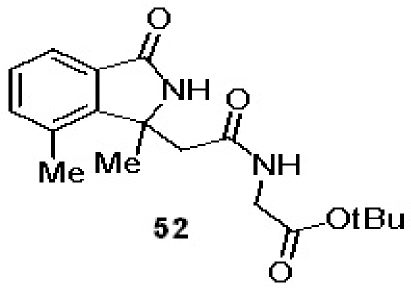

52

	Parameter	Value
1	Title	grd-2-299.1.fid
2	Solvent	CDCl3
3	Temperature	297.9
4	Number of Scans	16
5	Receiver Gain	18.0
6	Relaxation Delay	1.0000
7	Pulse Width	10.5000
8	Spectrometer Frequency	600.32
9	Nucleus	1H

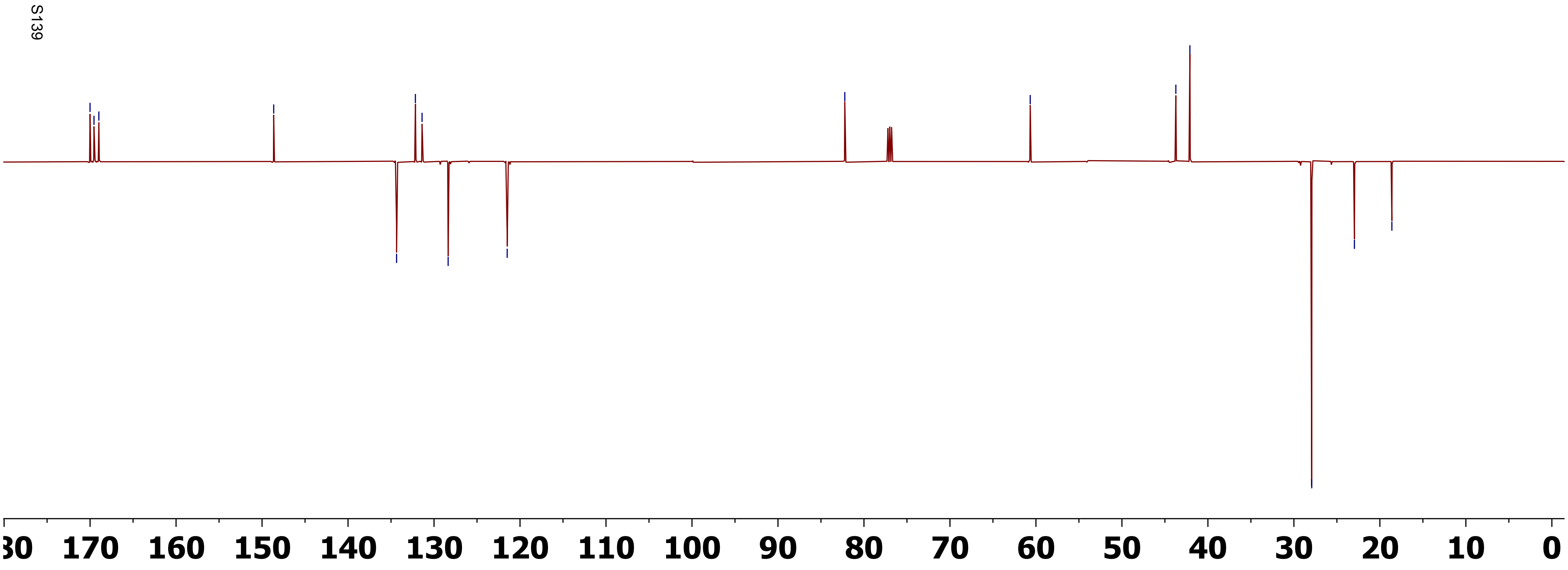

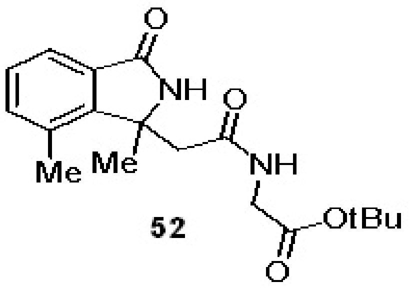

52

	Parameter	Value
1	Title	grd-2-299.2.fid
2	Solvent	CDCl3
3	Temperature	300.3
4	Number of Scans	256
5	Receiver Gain	2050.0
6	Relaxation Delay	5.0000
7	Pulse Width	10.6300
8	Spectrometer Frequency	150.97
9	Nucleus	13C

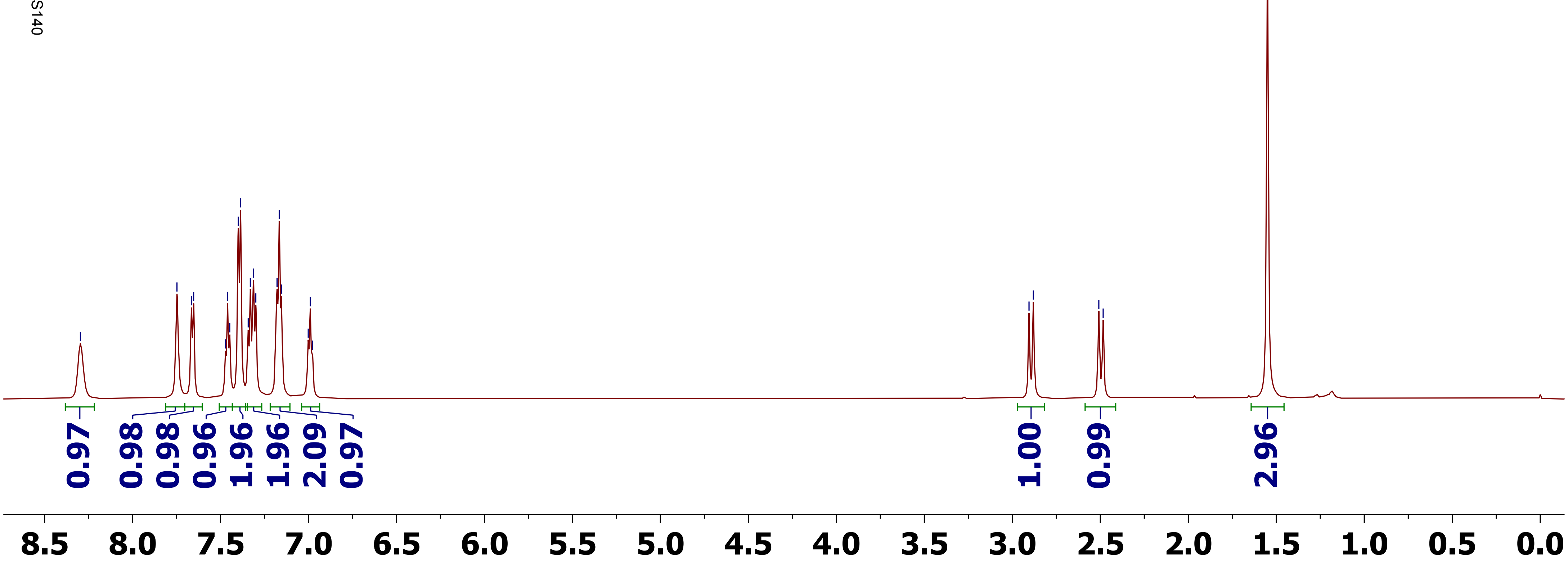

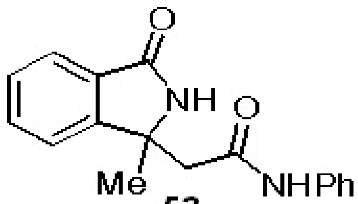

53

	Parameter	Value
1	Title	grd-2-275.2.fid
2	Solvent	CDCl3
3	Temperature	300.0
4	Number of Scans	16
5	Receiver Gain	57.0
6	Relaxation Delay	1.0000
7	Pulse Width	10.5000
8	Spectrometer Frequency	600.32
9	Nucleus	1H

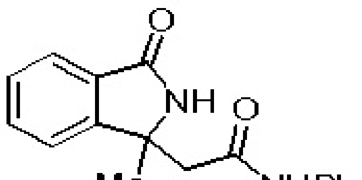

53

	Parameter	Value
1	Title	grd-2-275.4.fid
2	Solvent	CDCl3
3	Temperature	300.0
4	Number of Scans	256
5	Receiver Gain	2050.0
6	Relaxation Delay	5.0000
7	Pulse Width	10.6300
8	Spectrometer Frequency	150.97
9	Nucleus	13C

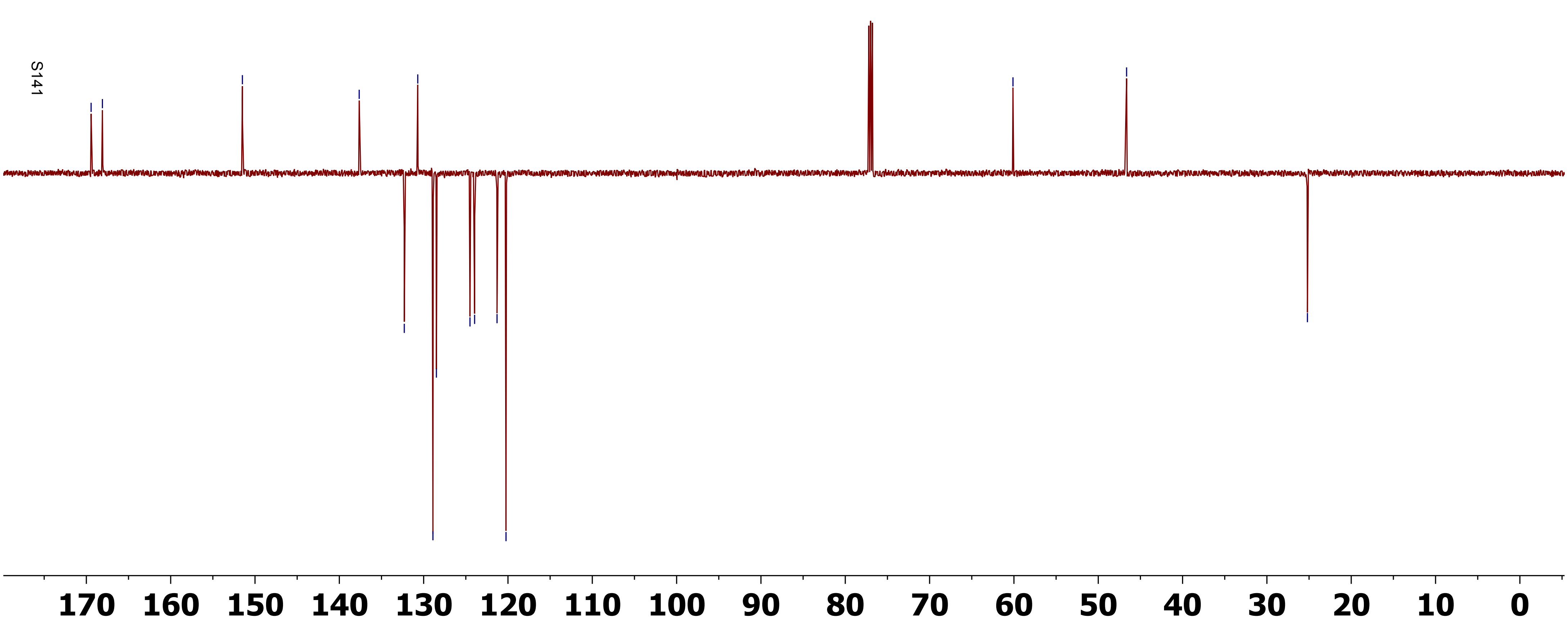

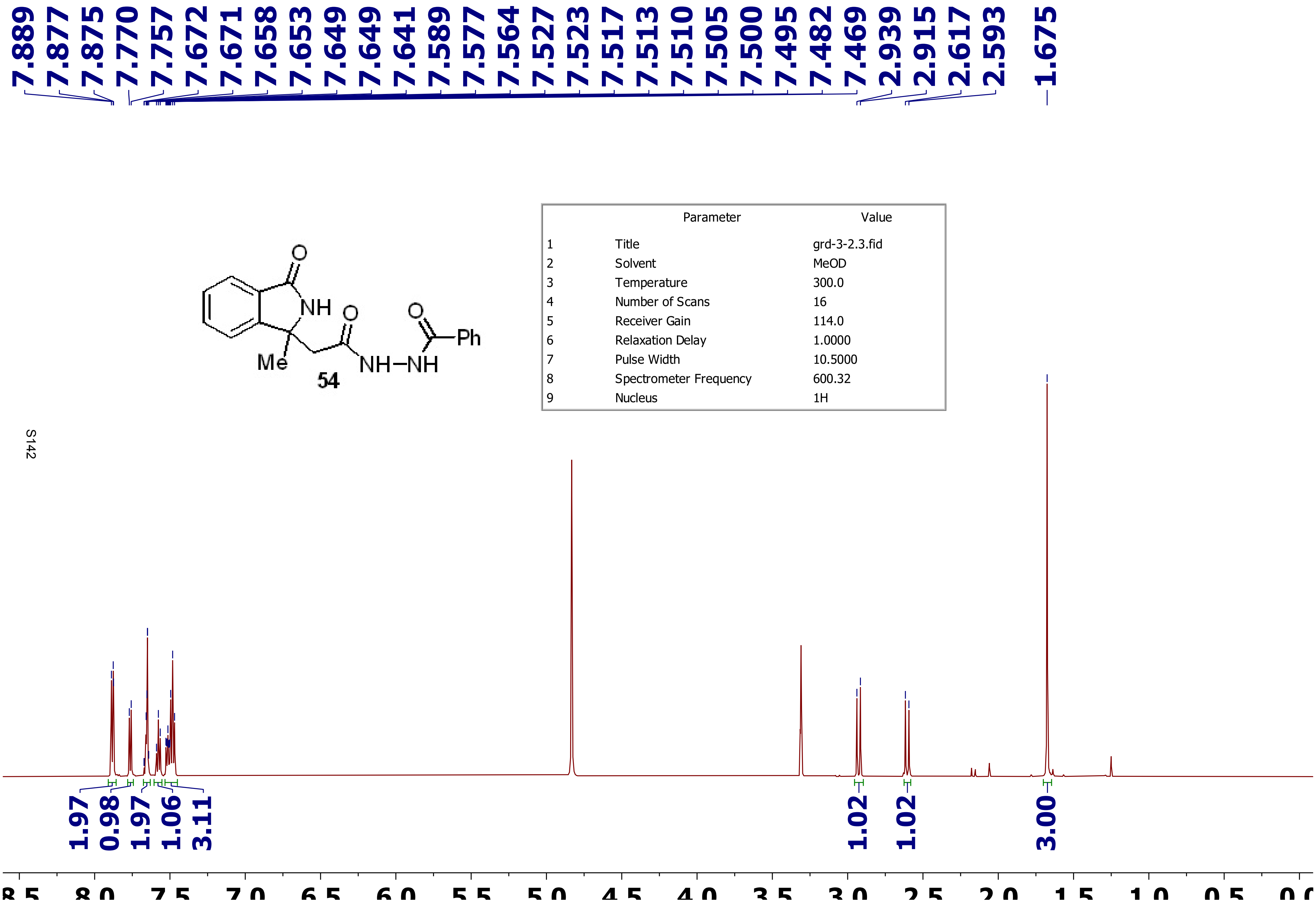

54

	Parameter	Value
1	Title	grd-3-2.3.fid
2	Solvent	MeOD
3	Temperature	300.0
4	Number of Scans	16
5	Receiver Gain	114.0
6	Relaxation Delay	1.0000
7	Pulse Width	10.5000
8	Spectrometer Frequency	600.32
9	Nucleus	1H

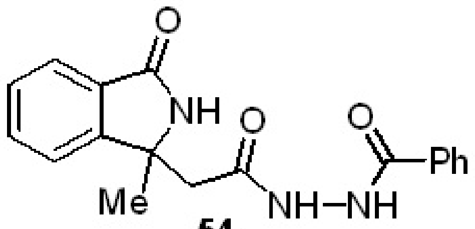

54

	Parameter	Value
1	Title	grd-3-2-pure.2.fid
2	Solvent	MeOD
3	Temperature	298.2
4	Number of Scans	1024
5	Receiver Gain	512.0
6	Relaxation Delay	3.0000
7	Pulse Width	10.7000
8	Spectrometer Frequency	100.62
9	Nucleus	13C

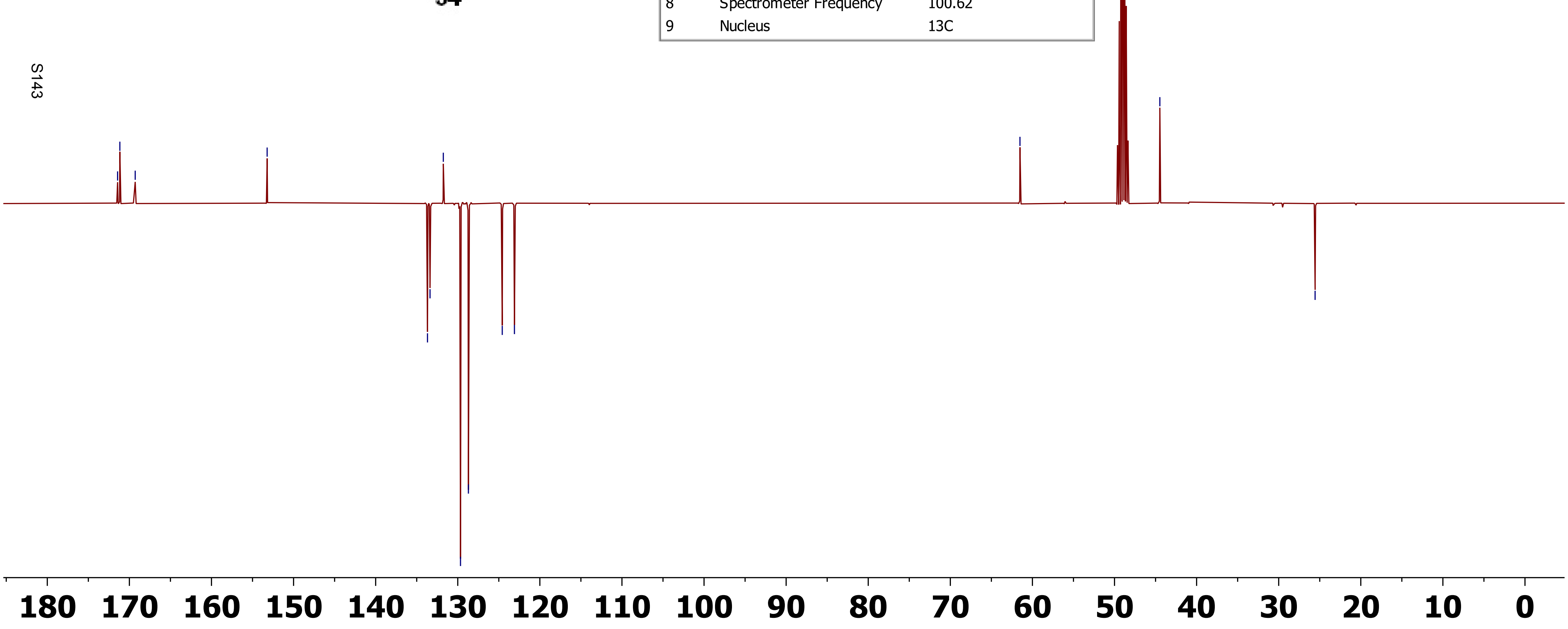

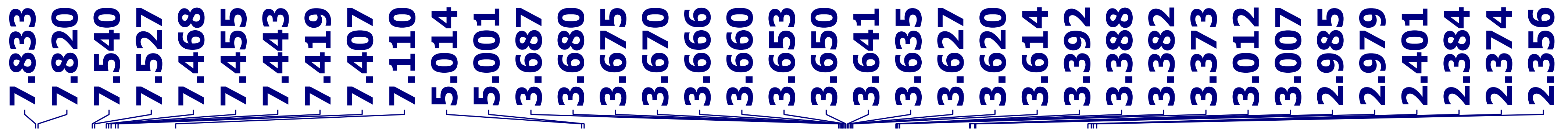

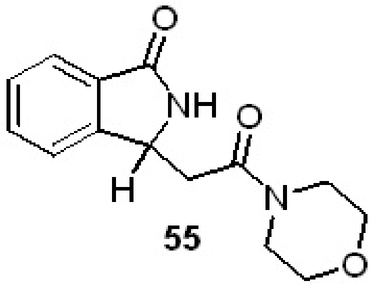

55

	Parameter	Value
1	Title	grd-2-283.5.fid
2	Solvent	CDCl3
3	Temperature	297.3
4	Number of Scans	16
5	Receiver Gain	57.0
6	Relaxation Delay	1.0000
7	Pulse Width	10.5000
8	Spectrometer Frequency	600.32
9	Nucleus	1H

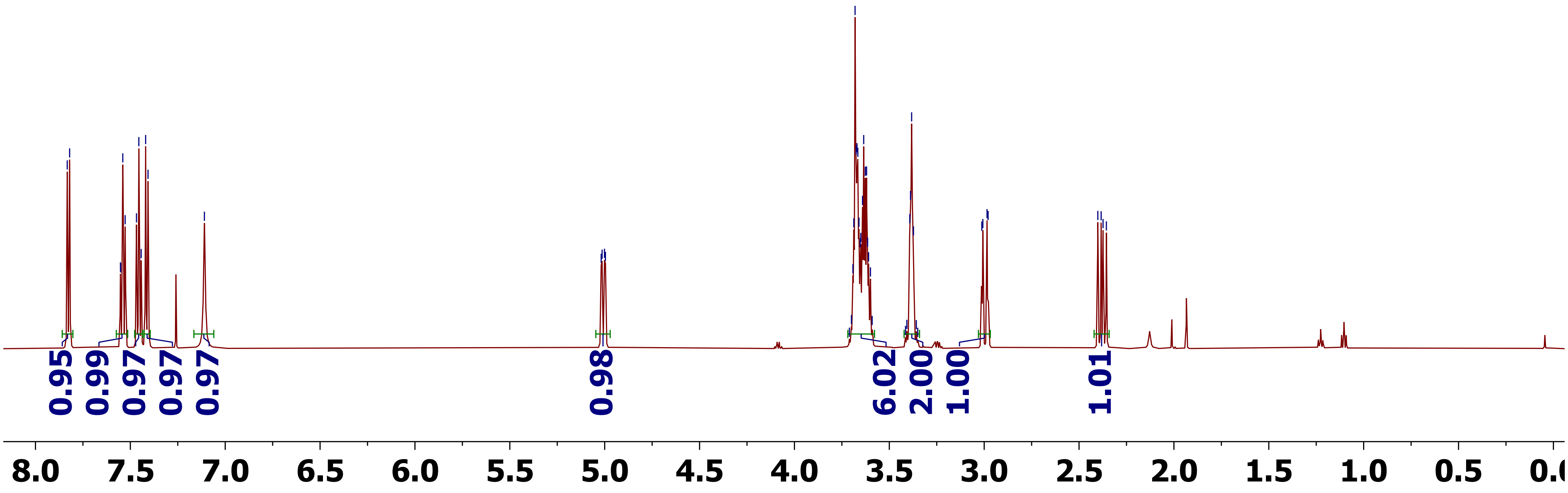

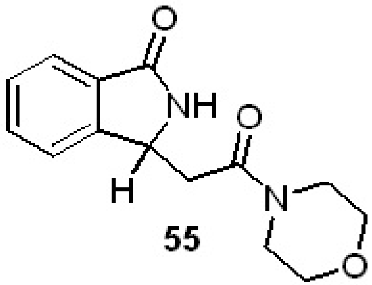

55

	Parameter	Value
1	Title	grd-2-283.6.fid
2	Solvent	CDCl3
3	Temperature	299.8
4	Number of Scans	256
5	Receiver Gain	2050.0
6	Relaxation Delay	5.0000
7	Pulse Width	10.6300
8	Spectrometer Frequency	150.97
9	Nucleus	13C

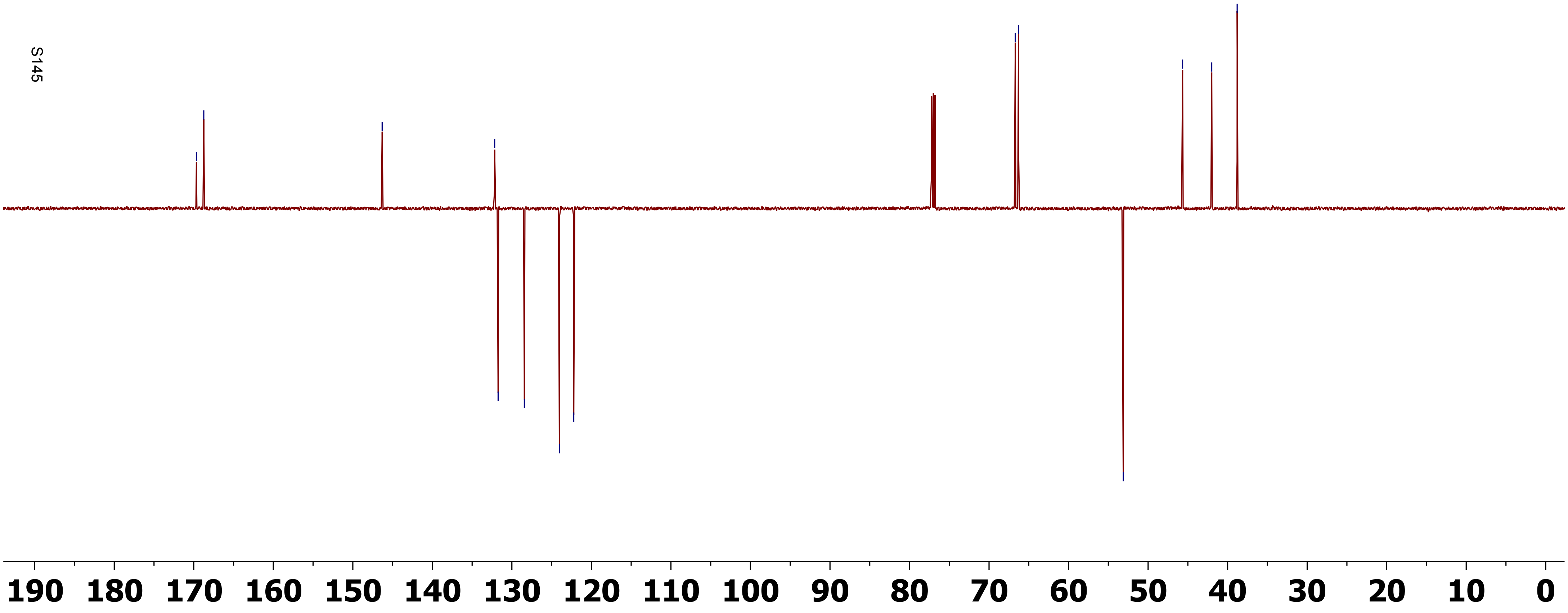

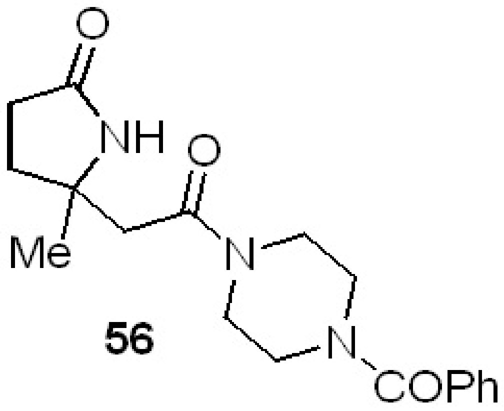

56

	Parameter	Value
1	Title	grd-2-285.3.fid
2	Solvent	CDCl3
3	Temperature	300.0
4	Number of Scans	16
5	Receiver Gain	40.3
6	Relaxation Delay	1.0000
7	Pulse Width	10.5000
8	Spectrometer Frequency	600.32
9	Nucleus	1H

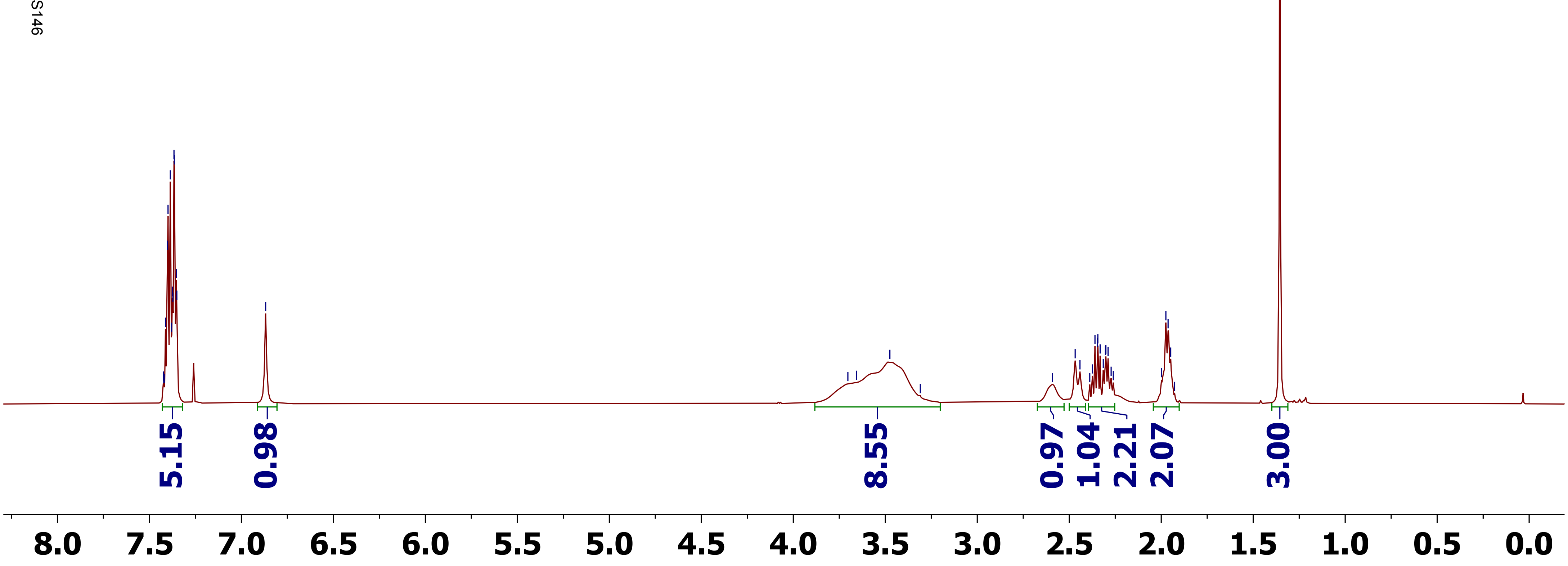

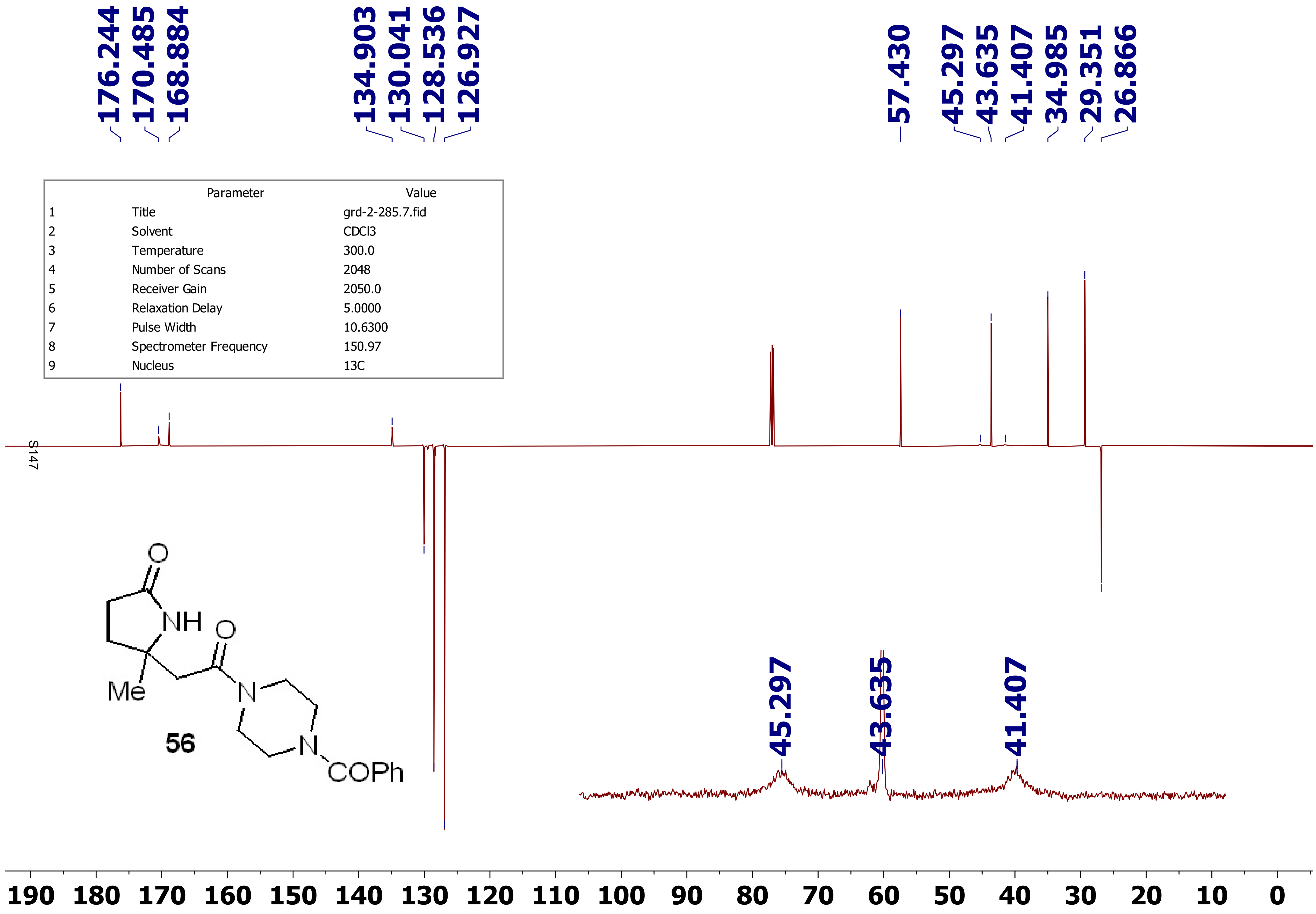

	Parameter	Value
1	Title	grd-2-285.7.fid
2	Solvent	CDCl3
3	Temperature	300.0
4	Number of Scans	2048
5	Receiver Gain	2050.0
6	Relaxation Delay	5.0000
7	Pulse Width	10.6300
8	Spectrometer Frequency	150.97
9	Nucleus	13C

56

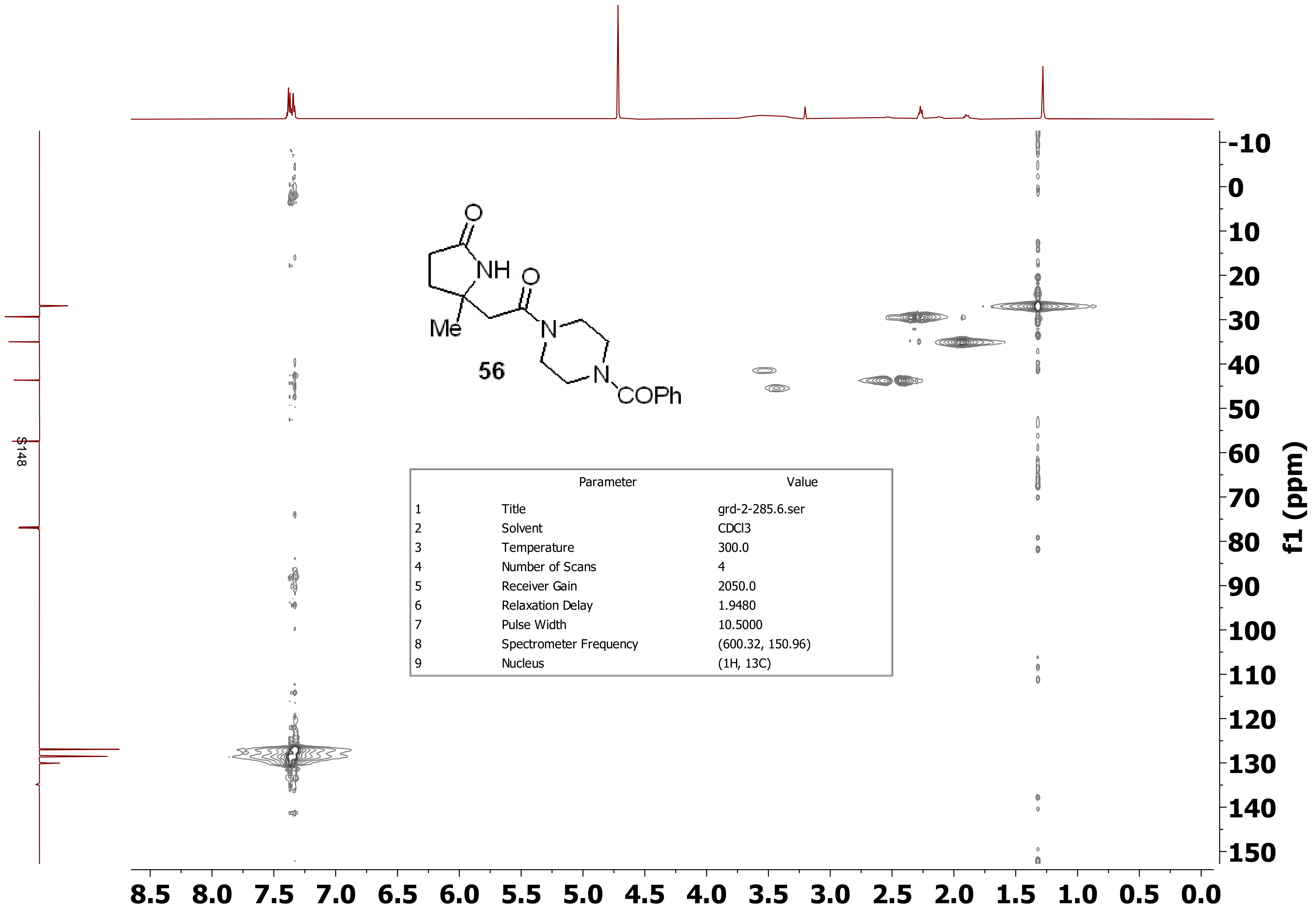

	Parameter	Value
1	Title	grd-2-285.6.ser
2	Solvent	CDCl3
3	Temperature	300.0
4	Number of Scans	4
5	Receiver Gain	2050.0
6	Relaxation Delay	1.9480
7	Pulse Width	10.5000
8	Spectrometer Frequency	(600.32, 150.96)
9	Nucleus	(1H, 13C)

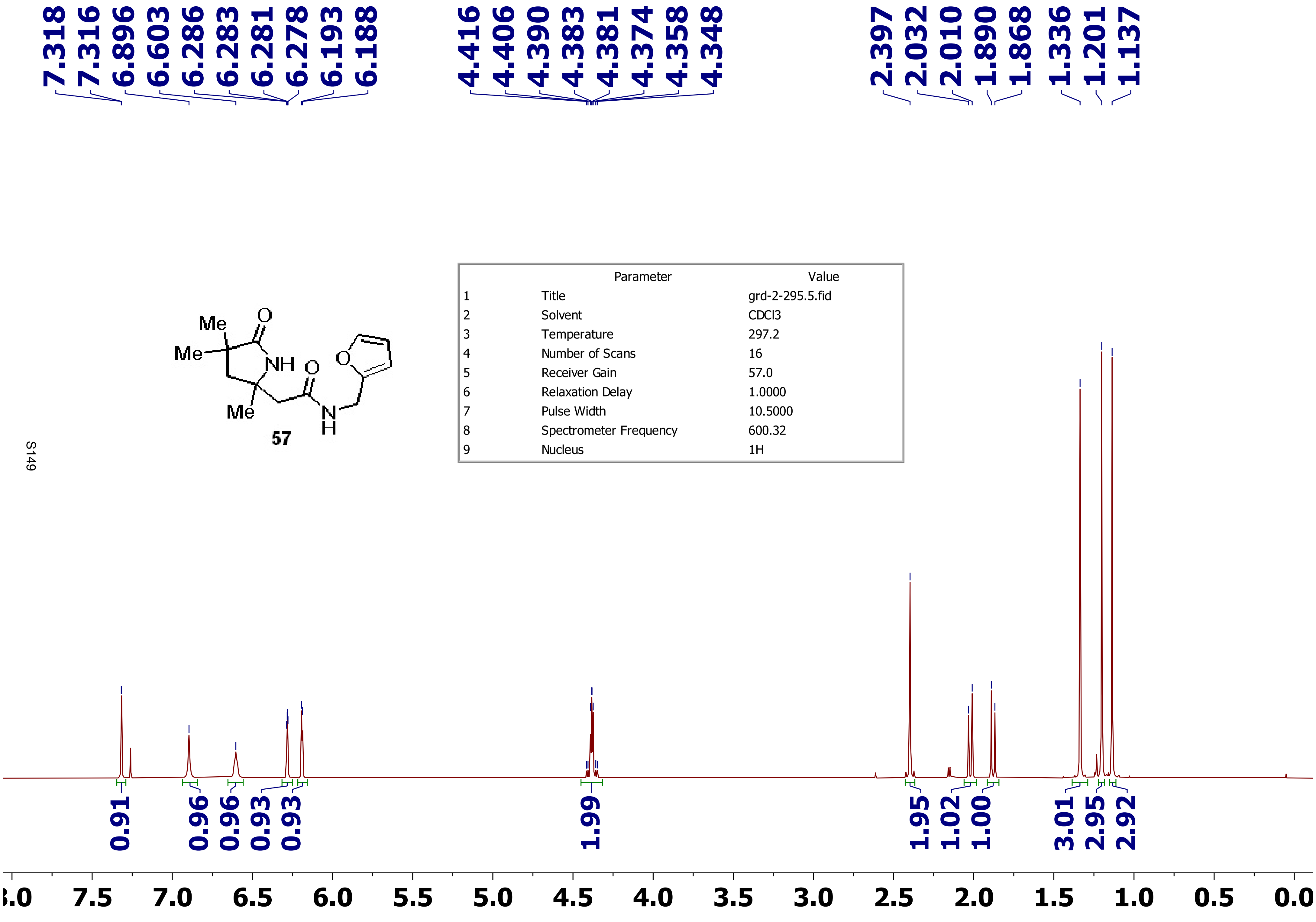

57

	Parameter	Value
1	Title	grd-2-295.5.fid
2	Solvent	CDCl3
3	Temperature	297.2
4	Number of Scans	16
5	Receiver Gain	57.0
6	Relaxation Delay	1.0000
7	Pulse Width	10.5000
8	Spectrometer Frequency	600.32
9	Nucleus	1H

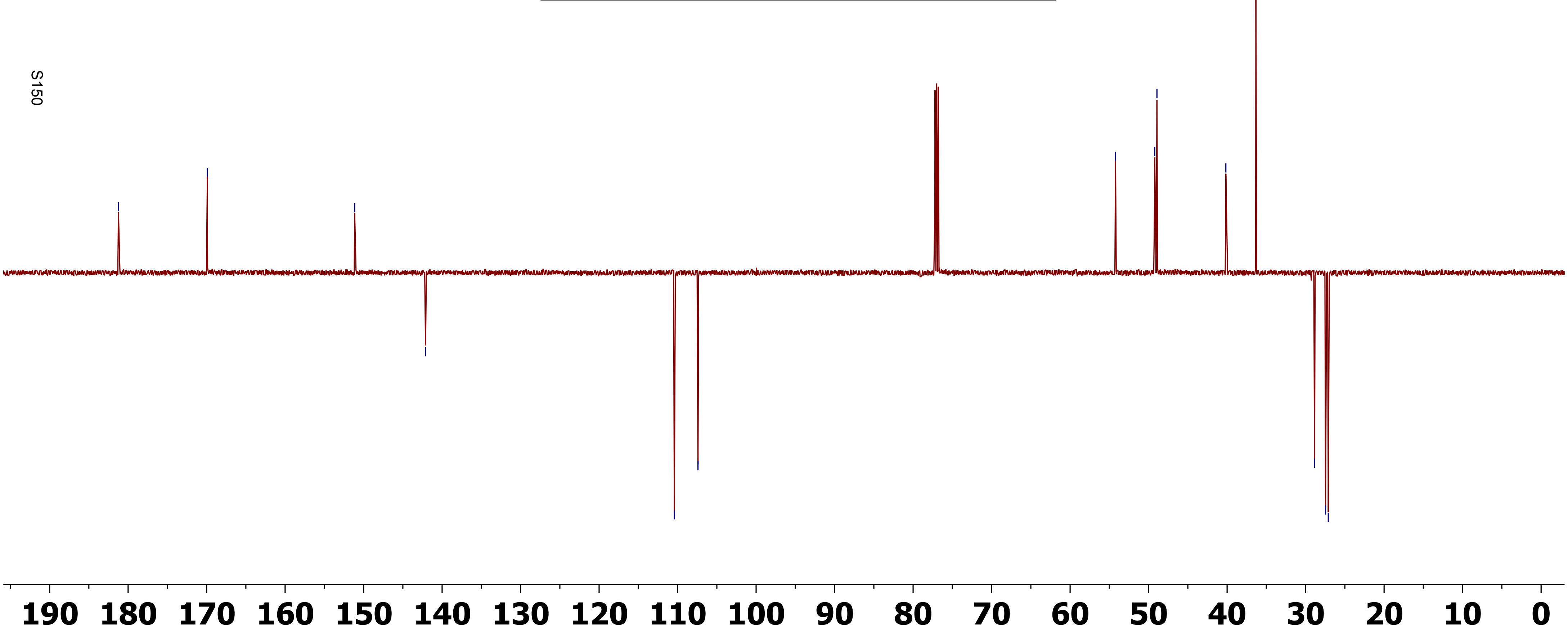

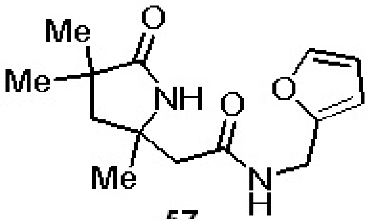

57

	Parameter	Value
1	Title	grd-2-295.6.fid
2	Solvent	CDCl3
3	Temperature	299.7
4	Number of Scans	256
5	Receiver Gain	2050.0
6	Relaxation Delay	5.0000
7	Pulse Width	10.6300
8	Spectrometer Frequency	150.97
9	Nucleus	13C

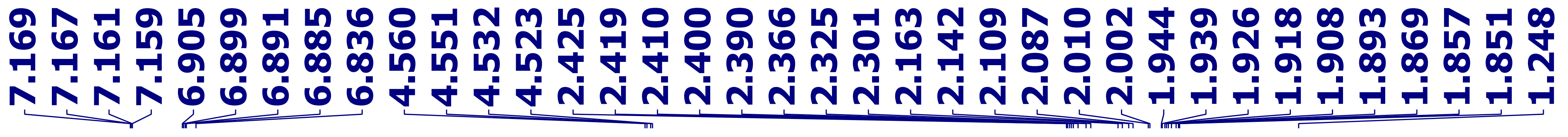
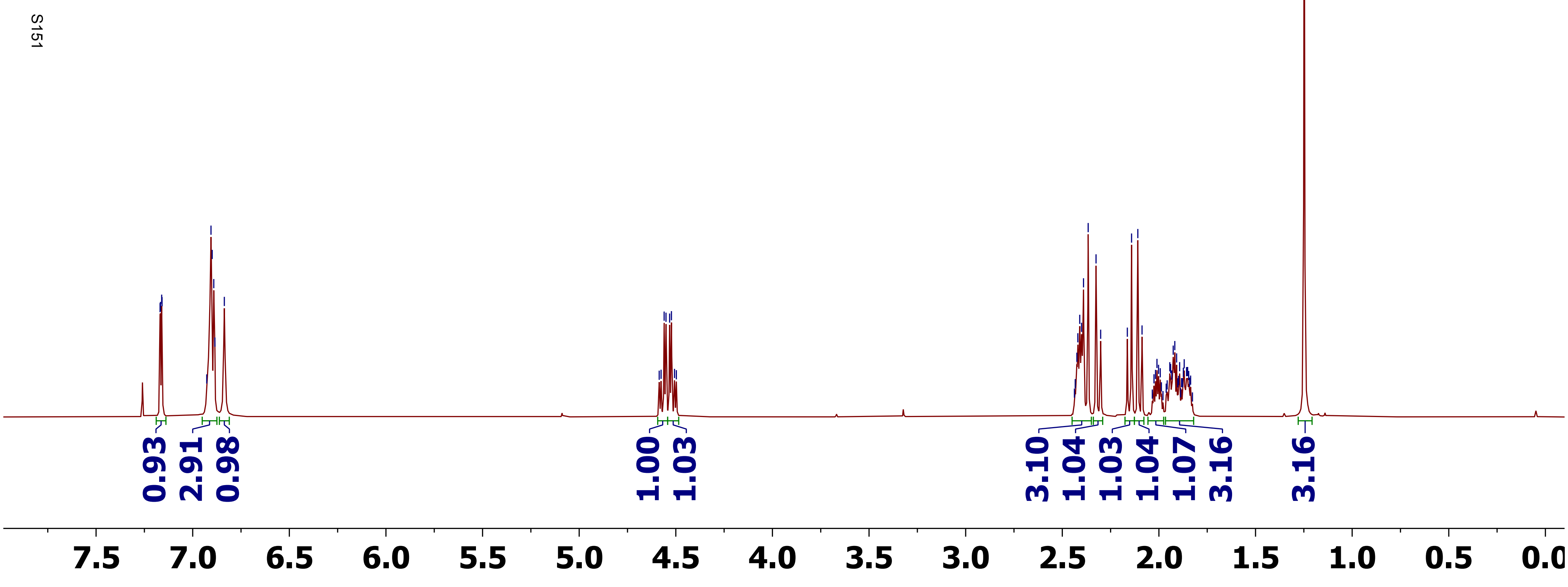

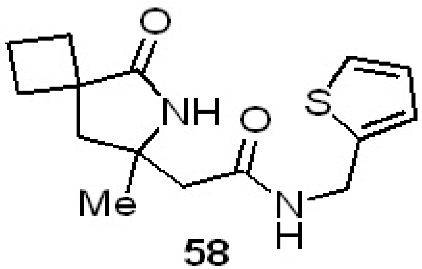

58

	Parameter	Value
1	Title	grd-2-296.2.fid
2	Solvent	CDCl3
3	Temperature	297.2
4	Number of Scans	16
5	Receiver Gain	40.3
6	Relaxation Delay	1.0000
7	Pulse Width	10.5000
8	Spectrometer Frequency	600.32
9	Nucleus	1H

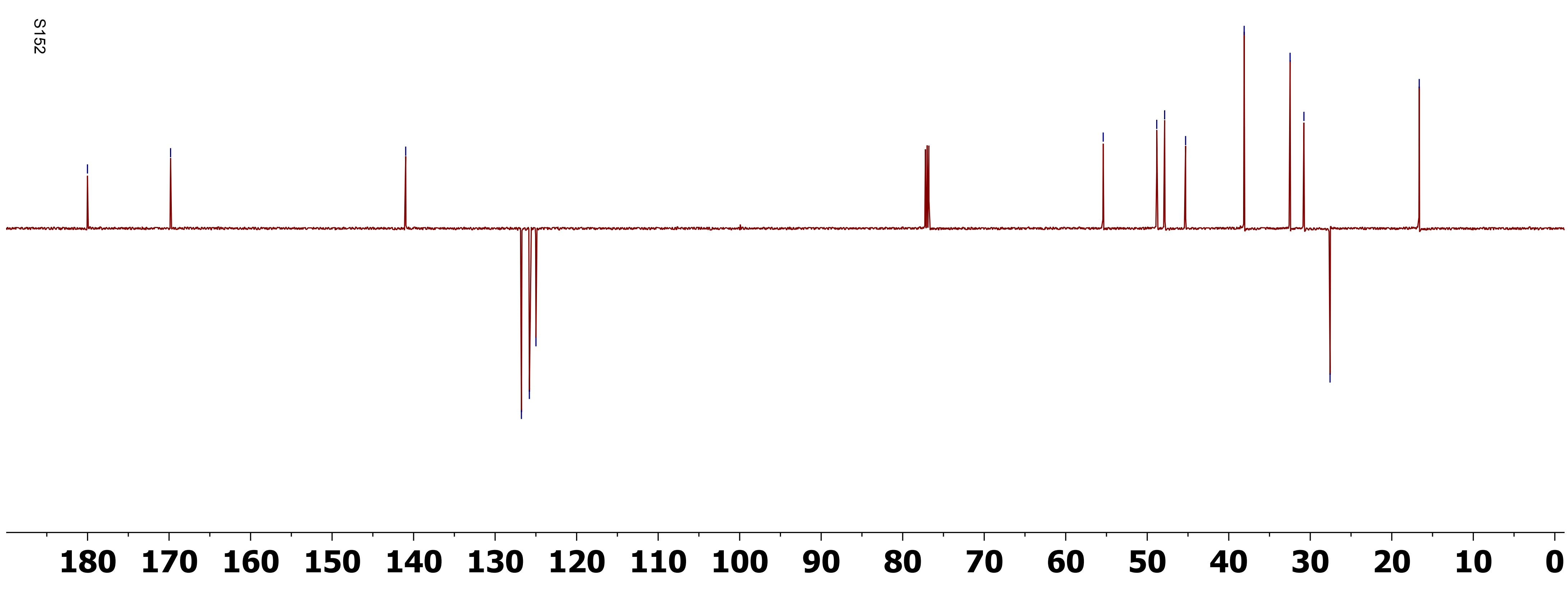

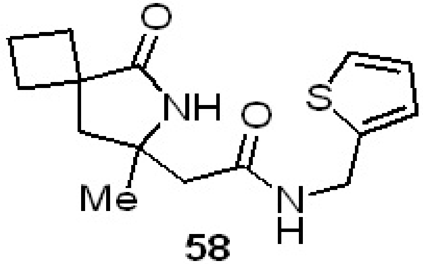

58

	Parameter	Value
1	Title	grd-2-296.3.fid
2	Solvent	CDCl3
3	Temperature	299.7
4	Number of Scans	256
5	Receiver Gain	2050.0
6	Relaxation Delay	5.0000
7	Pulse Width	10.6300
8	Spectrometer Frequency	150.97
9	Nucleus	13C

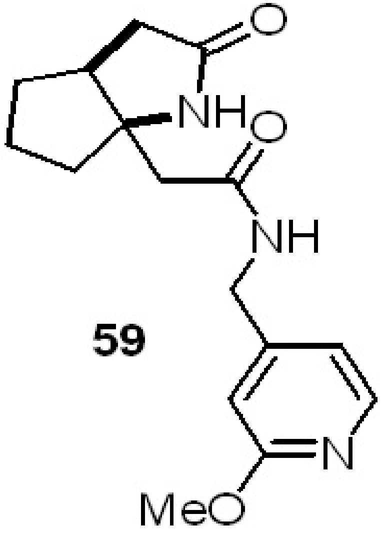

59

	Parameter	Value
1	Title	grd-3-3.4.fid
2	Solvent	CDCl3
3	Temperature	297.4
4	Number of Scans	16
5	Receiver Gain	32.0
6	Relaxation Delay	1.0000
7	Pulse Width	10.5000
8	Spectrometer Frequency	600.32
9	Nucleus	1H

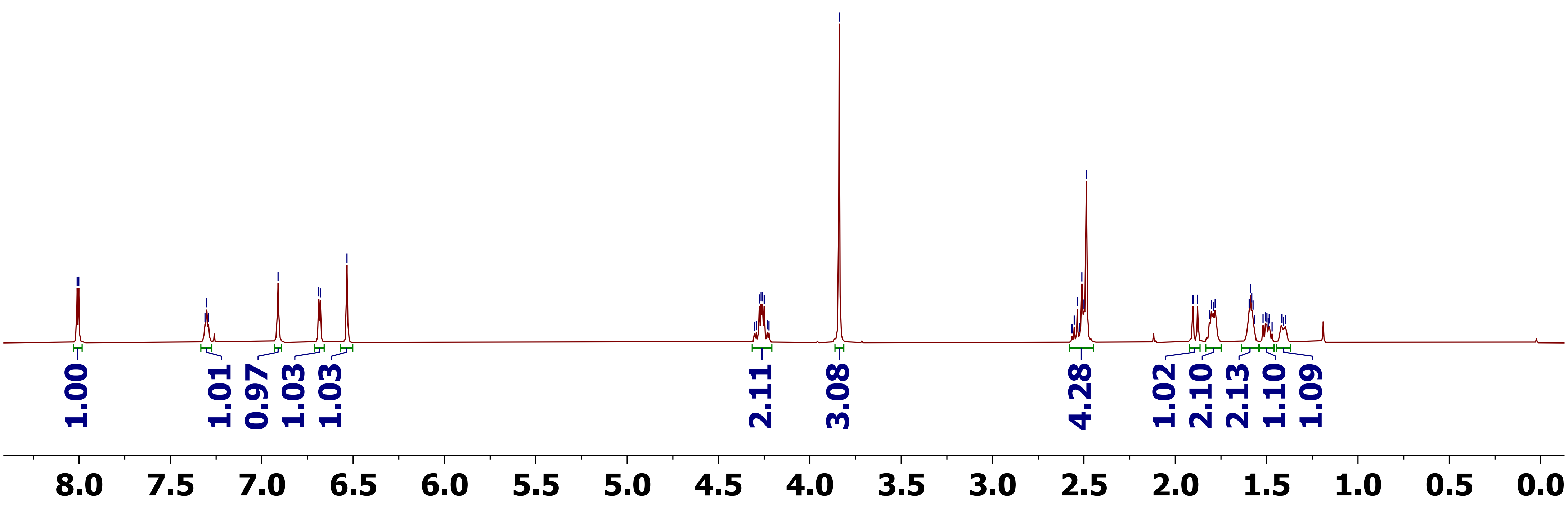

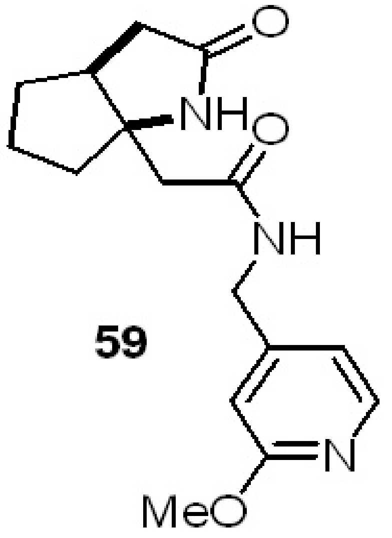

59

	Parameter	Value
1	Title	grd-3-3.5.fid
2	Solvent	CDCl3
3	Temperature	299.8
4	Number of Scans	256
5	Receiver Gain	2050.0
6	Relaxation Delay	5.0000
7	Pulse Width	10.6300
8	Spectrometer Frequency	150.97
9	Nucleus	13C

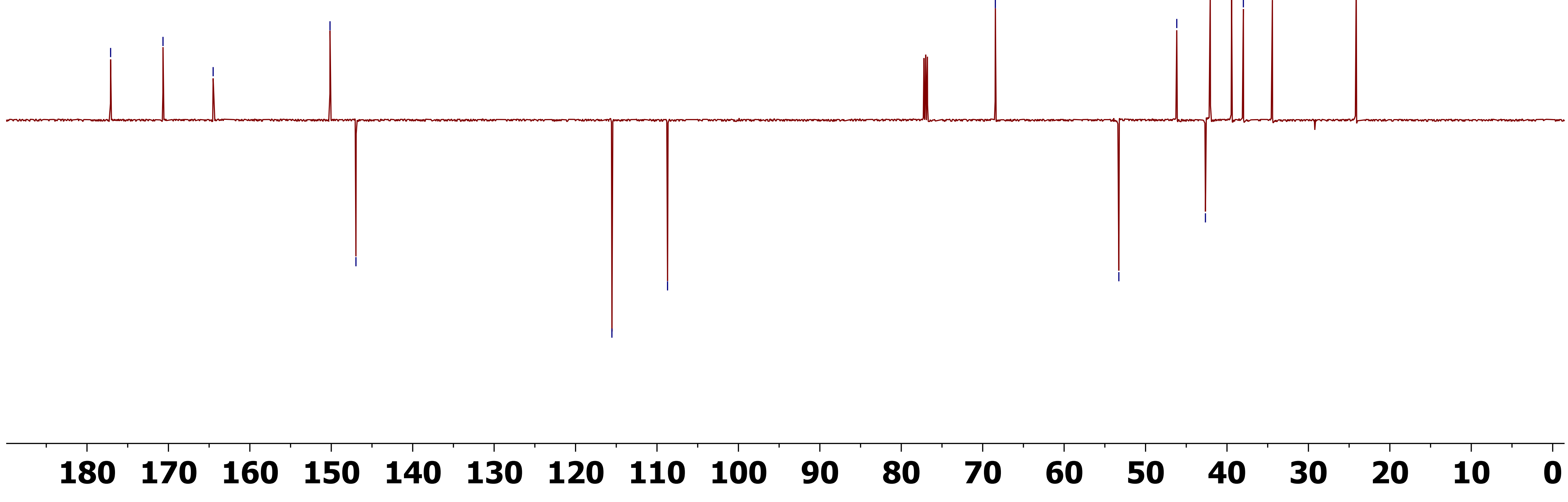

Supplement: SC-012-D1SC02075G-s001 [file SC-012-D1SC02075G-s001.pdf]
